# Cobalt Catalyst Determines Regioselectivity in Ring Opening of Epoxides with Aryl Halides

**DOI:** 10.1021/jacs.1c00659

**Published:** 2021-06-03

**Authors:** Aleksandra Potrząsaj, Mateusz Musiejuk, Wojciech Chaładaj, Maciej Giedyk, Dorota Gryko

**Affiliations:** Institute of Organic Chemistry, Polish Academy of Sciences, Kasprzaka 44/52, 01-224 Warsaw, Poland

## Abstract

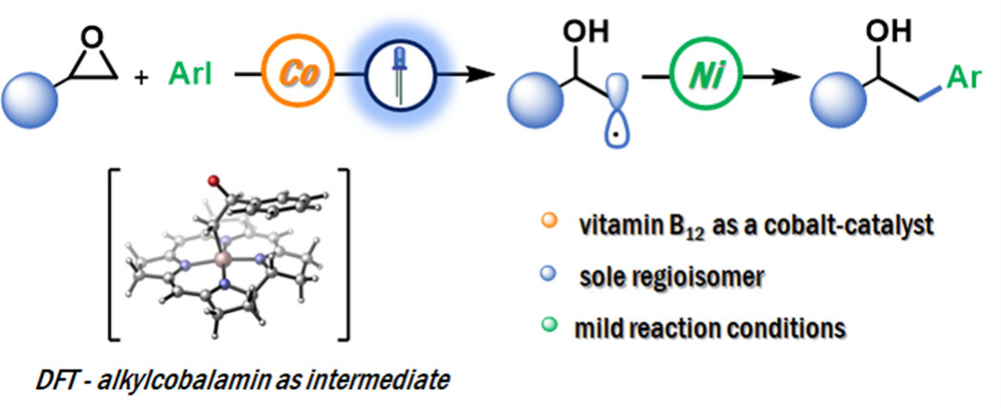

Ring-opening of epoxides furnishing either linear or branched products belongs to the group of classic transformations in organic synthesis. However, the regioselective cross-electrophile coupling of aryl epoxides with aryl halides still represents a key challenge. Herein, we report that the vitamin B_12_/Ni dual-catalytic system allows for the selective synthesis of linear products under blue-light irradiation, thus complementing methodologies that give access to branched alcohols. Experimental and theoretical studies corroborate the proposed mechanism involving alkylcobalamin as an intermediate in this reaction.

## Introduction

**Scheme 1:**
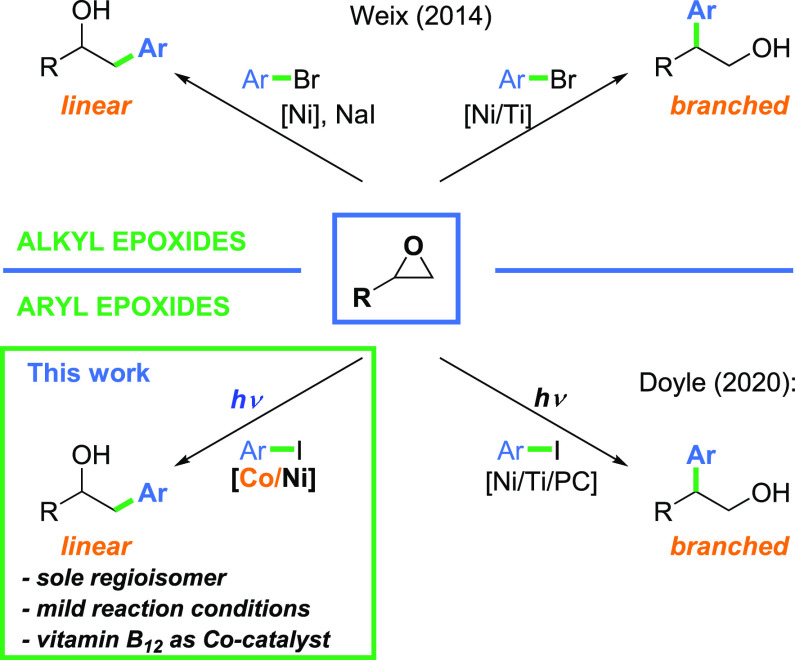
Regioselective Nickel-Catalyzed Cross-Electrophile Coupling of Epoxides with Aryl Halides

Driven by high demand for sustainable and efficient reactions, the discovery of selective reactivity patterns remains a key challenge. As epoxides are crucial building blocks in the synthesis of nonsymmetrical alcohols, their regioselective reactions have been intensively studied.^1−3^ In particular, considerable attention has been recently devoted to the utilization of epoxides in cross-electrophile couplings leading, in general, to regioisomeric (linear and branched) products (Scheme 1).^4−7^ It has been shown that the innately electrophilic epoxides can be transformed into radicals and, as such, be involved in a transition-metal-catalytic cycle.^7,8^ Depending on reaction conditions, the initial nucleophilic attack occurs predominantly at either the terminal or internal carbon atom.

In 2014, Weix and co-workers developed a nickel-catalyzed, regiodivergent cross-electrophile coupling of epoxides with various halides and triflates.^6^ For aliphatic epoxides (Scheme 1, upper part), the regioselectivity of the ring-opening step depends on the cocatalyst used. Sodium iodide promotes the formation of a linear product. The nucleophilic attack of the iodide anion at the less substituted carbon atom affords iodohydrin, which in turn undergoes reduction and Ni-catalyzed coupling with an electrophile. On the other hand, in the presence of a titanocene cocatalyst secondary alkyl radicals are generated, facilitating the formation of branched products.^9,10^*Aryl epoxides, however, react predominantly at the benzylic position, regardless of the conditions employed*. A similar reactivity pattern has been recently reported by the Doyle group, who used organic iodides and the Ti/Ni/photoredox catalytic system in ring-opening reactions of three major classes of epoxides, namely, aryl, aliphatic, and bicyclic (Scheme 1, lower part).^11^ By changing a nickel complex, the authors were able to transform aliphatic epoxides into linear products, while aryl epoxides selectively formed branched ones. Despite the enormous importance of these contributions, *the synthesis of linear products from aryl epoxides via cross-electrophile coupling still represents an unsolved challenge*.

Our recent work on the alkylation of strained molecules showed that cobalt catalysis opens the path to a polarity-reversal strategy for radical couplings.^12^ We questioned whether it would be possible to adapt this methodology to achieve selective reactions of epoxides. Herein, we disclose that the nucleophilicity of Co(I) species along with sterically restricted side chains allows generating C-radicals from epoxides in a selective manner and engage them in Ni-catalyzed cross-coupling.

## Results and Discussion

### Design of the Catalytic System

**Scheme 2:**
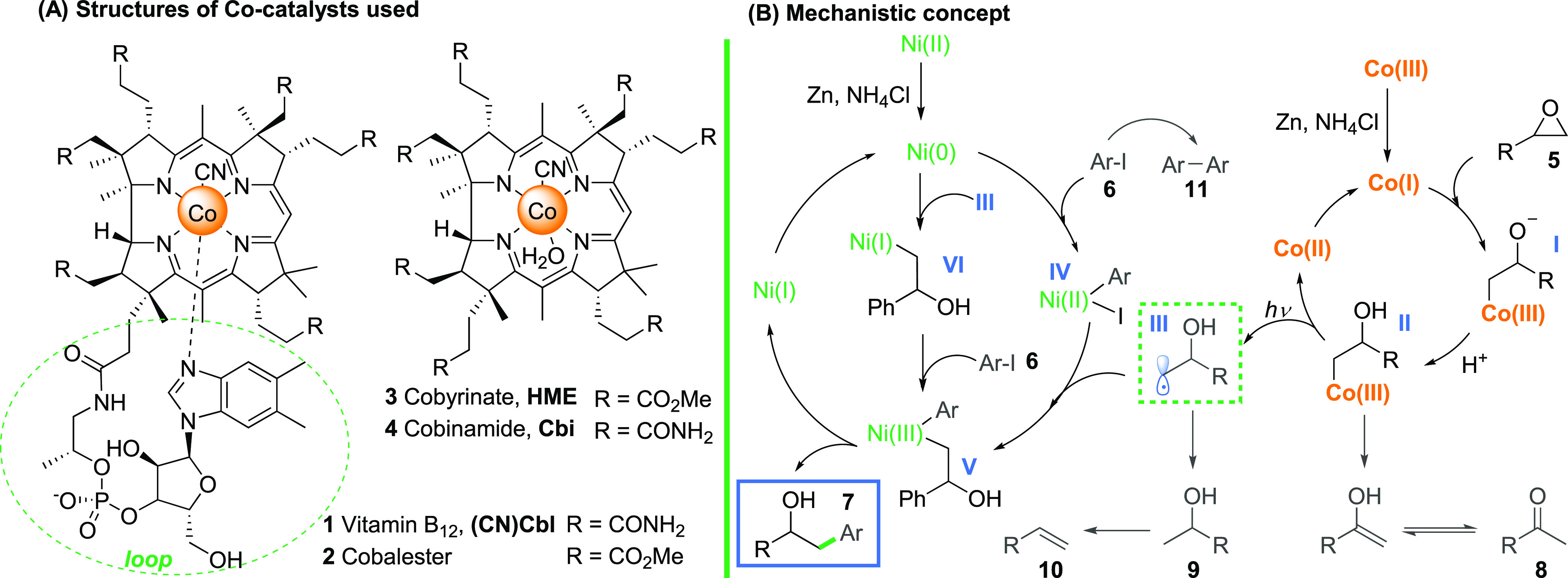
(A) Structures of Cocatalysts: Vitamin B_12_ and Derivatives; (B) Proposed Mechanistic Concept

Vitamin B_12_ (**1**, cobalamin) is a natural cobalt complex of remarkable stability and high biological importance.^13−15^ Due to the unique ability to form light-sensitive cobalt–carbon bonds, vitamin B_12_ (**1**) and its hydrophobic and amphiphilic derivatives **2** and **3** (Scheme 2A)^16−21^ have also been adopted for synthetic chemistry and used as redox mediators for the generation of various radicals.^22,23^ We assumed that a nucleophilic Co(I) complex that forms upon the reduction of cobalamin should open electrophilic epoxides, generating alkyl cobalamins (Scheme 2B). Such intermediates, upon light irradiation, undergo the homolytic Co–C bond cleavage to give alkyl radicals, which can be engaged in a number of both radical reactions and transition-metal-catalyzed cross-couplings. *Importantly, from the viewpoint of regioselective design, a bulky vitamin B*_*12*_*catalyst should attack an epoxide from the less sterically hindered side*. This kinetic factor may prevail over the high thermodynamic preference for stabilized benzyl radicals and thus allow the selective formation of primary radicals of type **III**.

**Figure 1:**
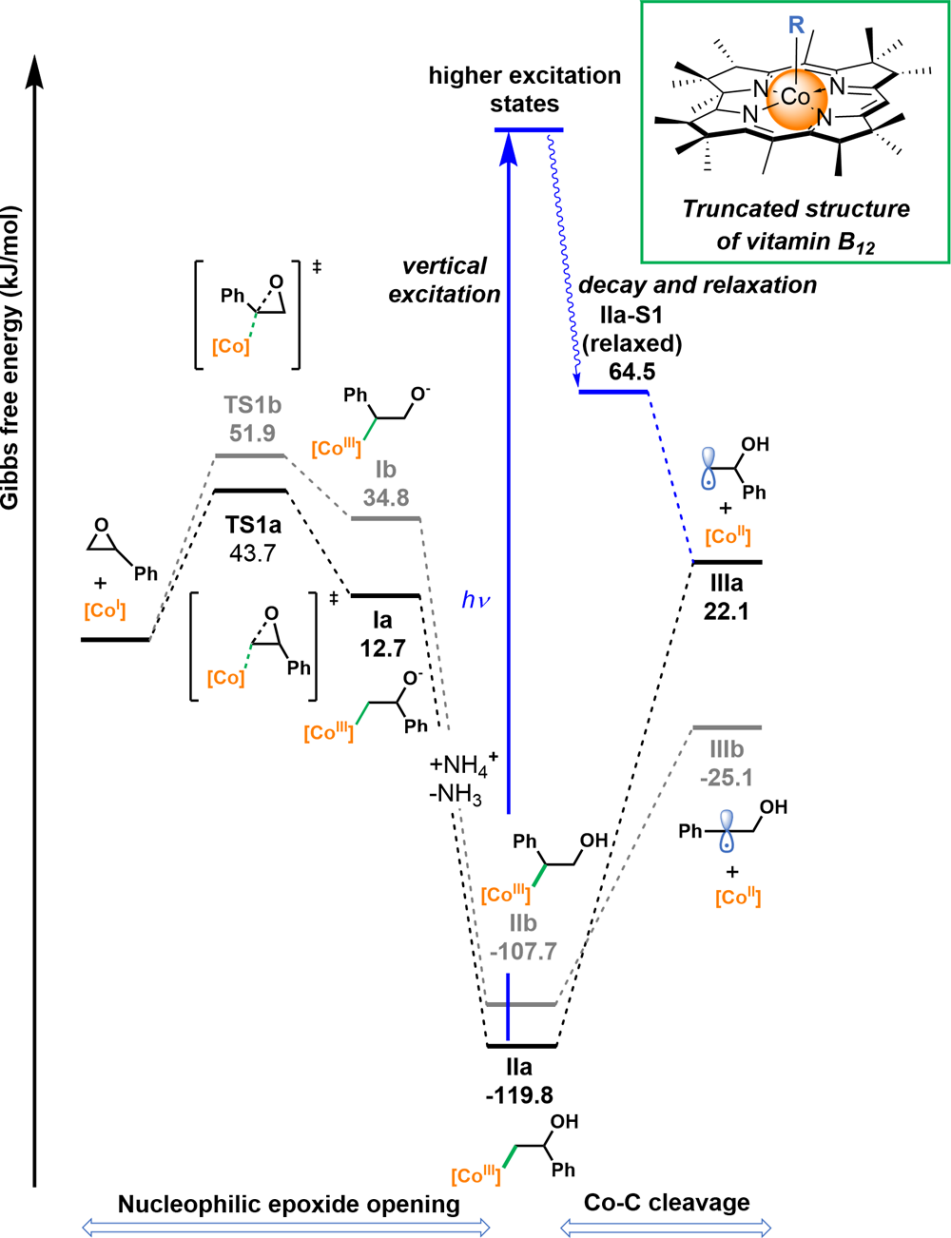
Calculated Gibbs free energy profile for the reaction of styrene oxide with the Co(I)-corrin complex.

To examine our hypothesis, in the first instance, we theoretically investigated the possible formation of alkyl radicals via a sequence of the epoxide ring opening with reduced vitamin B_12_ (Co(I) complex) followed by homolytic cleavage of the Co–C bond in alkyl cobalamin **II**. DFT calculations were performed with Gaussian 16.^24^ Geometry optimizations were computed at the BP86/6-31G(d) level of theory with the D3 version of Grimme’s empirical dispersion correction and solvation (acetone) with the SMD model. Frequency analysis was performed at the same level to provide correction to thermodynamic functions and confirm the nature of optimized structures (minima and transition states featured zero or one imaginary frequency, respectively). Single-point energies were computed at the BP86/6-311++G(2df,p) level of theory with the D3 version of Grimme’s empirical dispersion correction and solvation (acetone) with the SMD model. Several hybrid and long-range corrected functionals were tested for the model reaction (see Supporting Information (SI) for details). BP86 was, however, selected for further studies due to good performance reported for both ground and exited state calculations of cobalamin systems.^25−30^ We performed calculations approximating the structure of vitamin B_12_ (**1**) with a Co-corrin complex bearing 15 methyl groups, reflecting the substitution pattern at the periphery of the macrocyclic ring (Figure 1).

The calculated Gibbs free energy profile for the benchmark reaction of styrene oxide (**5a**) with the Co-corrin complex is depicted in Figure 1. Two paths, involving the nucleophilic attack on either side of the epoxide, were considered. In line with our assumptions, the ring opening of the epoxide with the nucleophilic Co(I) complex should proceed at the less hindered terminus with a 43.7 kJ/mol barrier, accessible even under mild conditions (black path). The barrier for the analogous reaction at the more hindered side is ∼8 kJ/mol higher (gray path). Sterically driven differences in the reactivity might be even more pronounced for native vitamin B_12_ or its derivatives compared to the selected model, due to presence of more sizable substituents at the corrin ring. Then, the resulting Co(III) complex (**I**) is protonated, providing intermediate **II**. As expected for alkyl cobalamins, the Co–C(sp^3^) bond in **IIa** and **IIb** is relatively weak and quite vulnerable to homolytic cleavage toward alkyl radical **IIIa** or **IIIb** and a Co(II) complex (Δ*G* = 141.9 and 82.6 kJ/mol, respectively). In particular, **IIa** could undergo Co–C photodissociation, presumably through the mechanism proposed by Kozlowski, involving generation of the singlet radical pair from the first electronically excited state (S1).^31−33^

The lowest singlet (**IIa-S1**) vertically excited states of intermediate **IIa** were found at 2.20 eV (212.5 kJ/mol, TD BP86-D3/6-311++G(2df,p)), while the relaxed S1 state lies 28.2 kJ/mol lower and features elongation of the Co–C bond by 0.22 Å. Noticeably, due to the preference for the nucleophilic attack at the less hindered side of the epoxide, the above-described path (black) should provide access to a 2-hydroxy-2-phenyl ethyl radical (**IIIa**), even though isomeric benzyl radical **IIIb** is thermodynamically more stable by 47.2 kJ/mol.

**Scheme 3:**
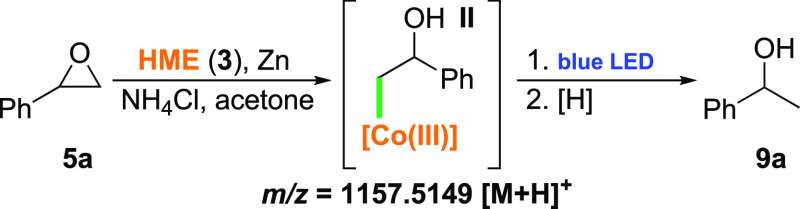
Vitamin B_12_-Catalyzed Ring Opening of Epoxide **5**

To support theoretical studies, the reductive photochemical ring opening of styrene oxide (**5a**) in the presence of a hydrophobic vitamin B_12_ derivative, HME (**3**), was performed (Scheme 3). The selected Co complex **3** allows convenient monitoring of reactive intermediates by ESI mass spectrometry due to its tendency to undergo facile ionization.

Indeed, the formation of intermediate alkyl-cobalt(III) complex **II** was observed by HR-MS (*m*/*z* = 1157.5149 [M + H]^+^, see the SI, Section 6.2), which is in good agreement with previous reports by Scheffold^34−38^ and Rusling,^39^ who used vitamin B_12_ (**1**) for isomerization of symmetrical epoxides to allyl alcohols. Satisfyingly, alcohol **9a** with a −OH group at the benzylic position formed just after 30 min. These results corroborate the proposed mechanistic concept in which vitamin B_12_ opens the aromatic epoxide from the less hindered side at the thermodynamic expense of forming the less stable radical **III** in the subsequent light-induced cleavage step.

Knowing that B_12_ catalysis can be merged with metal-catalyzed reactions,^12^ we next evaluated the feasibility of incorporating the generated alkyl radicals in the Ni catalytic cycle. Adding electrophilic aryl halides should enable cross-electrophile coupling and thus provide a convenient method for the carbon–carbon bond formation.^40^ The plausible mechanism for the reaction of epoxides with aryl halides in the presence of the B_12_/Ni catalytic system based on literature reports is outlined in Scheme 2B.^7,41−43^ The coupling requires the cooperation of both transition metal complexes (Co and Ni) that are activated by Zn/NH_4_Cl.^44,45^ The oxidative addition of aryl halide to Ni(0) produces aryl nickel(II) species **IV**, which undergoes subsequent alkylation with radical **III** and generates intermediate **V**. Alternatively, the same Ni(III) species can originate from the interception of alkyl radical **III** by Ni(0), preceding the oxidative addition, as has been recently proposed by Molander and Kozlowski.^46^ Both these possible pathways are followed by irreversible reductive elimination, leading to the regioselective formation of a linear product. Cobalt(II) and nickel(I) complexes are regenerated to Co(I) and Ni(0) with Zn, thereby closing the cycles.

**Scheme 4:**
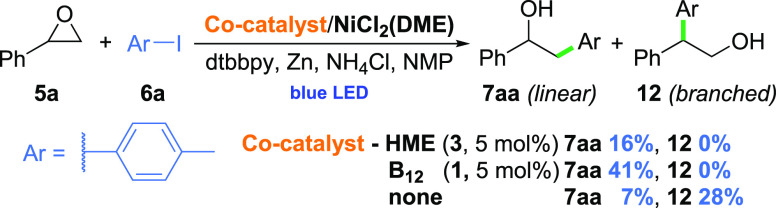
Proof-of-Concept Experiments Conditions: epoxide (**5a**, 0.2 mmol), aryl halide (**6a**, 1.5 equiv), NiCl_2_(DME) (20 mol %), Zn (3 equiv), NH_4_Cl (3 equiv), dtbbpy (40 mol %), dry NMP (*c* = 0.1 M), blue LED (single diode, 10 W), 30 min.

Styrene oxide (**5a**), when subjected to the reaction with *p*-iodotoluene (**6a**) in the presence of HME (**3**) and NiCl_2_(DME), generated desired linear product **7aa** as a single regioisomer in 16% yield (Scheme 4). The replacement of HME (**3**) with native vitamin B_12_ increased the yield up to 41%. Noteworthy, the reaction without any cobalt complex added not only was lower-yielding but also led to a mixture of two regioisomers with a predominance of the branched product. *This result clearly shows the decisive influence of the cobalt cocatalysis on the selectivity of this transformation*. Control experiments confirmed the dual-catalytic and light-induced nature of the process, while the addition of a radical trap (TEMPO) supported its radical character (see SI).

Noteworthy, the reaction without any cobalt complex added not only was lower-yielding but also led to a mixture of two regioisomers with a predominance of the branched product. *This result clearly shows the decisive influence of the cobalt cocatalysis on the selectivity of this transformation*. Control experiments confirmed the dual-catalytic and light-induced nature of the process, while the addition of a radical trap (TEMPO) supported its radical character (see SI).

### Optimization

Next, we turned our attention toward the synthetic utility of the developed method. The reaction was optimized with respect to cobalt and nickel catalysts, solvent, ligand, and reducing system, providing the desired product **7aa** in 60% yield (Table 1, entry 1).

**Table 1:**
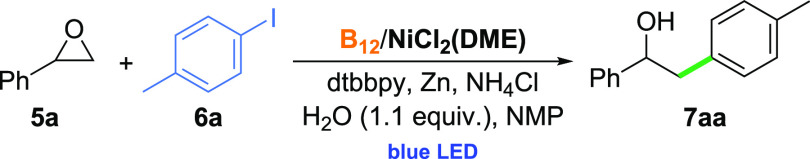
Optimization Studies of the Cross-Electrophile Ring Opening of Epoxides^a^

entry	deviation from the standard conditions	yield (%) **7aa**^a^
1	none	60
2	HME instead of B_12_	57
3	Co(acac)_3_ instead of B_12_	5
4	CoCl_2_ instead of B_12_	7
5	Co(dmgH)_2_Cl(py) instead of B_12_	8
6	Co(dmgH)_2_^i^Pr(py) instead of B_12_	11
7^b^	Mn instead of Zn	30
8	NiCl_2_ instead of NiCl_2_(DME)	36
9	Ni(acac)_2_ instead of NiCl_2_(DME)	33
10	Ni(OTf)_2_ instead of NiCl_2_(DME)	39
11	1,10-phenanthroline instead of dtbbpy	24
12	terpyridine instead of dtbbpy	13
13	no water added	53

a Conditions: epoxide (**5**, 0.2 mmol), aryl halide (**7**, 1.5 equiv), B_12_ (5 mol %), NiCl_2_(DME) (20 mol %), Zn (1.5 equiv), NH_4_Cl (3 equiv), dtbbpy (40 mol %), H_2_O (1.1 equiv), dry NMP (*c* = 0.1 M), time 30 min, blue LED (single diode, 10 W) (for more details see SI).

b Mn (1.5 equiv), TMSCl (0.2 equiv), dmgH = dimethylglyoxime, dtbbpy = 4,4′-di-*tert*-butylbipyridine.

We found that the addition of water (1.1 equiv) improved the yield of the reaction, while kinetic studies allowed us to determine the optimal reaction time (30 min, for details, see SI). The use of hydrophobic HME (**3**) instead of the parent vitamin B_12_ had little impact on the optimized model reaction (entry 2), while other commonly utilized cobalt complexes (Co(acac)_3_, CoCl_2_) led to a decrease in the yield of alcohol **7aa** (entries 3, 4). We have also examined cobalt dimethylglyooximate (dmg) complexes, which have been used by Pattenden^47^ and Morandi^48^ in regioselective cobalt-catalyzed coupling of aliphatic epoxides with alkenes. In our system, however, both catalysts afforded the desired product **7aa** only in low yields (entries 5, 6). Evaluation of reducing agents ruled out manganese or tetrakis(dimethylamino)ethylene (TDAE) as an efficient alternative to the Zn/NH_4_Cl system (entry 7). It also allowed establishing the optimal ratio of the two components at the 1.5 equiv: 3 equiv level. The reaction outcome did not improve in the presence of NiCl_2_, Ni(acac)_2_, or Ni(OTf)_2_ as well as other ligands (entries 8–12). Finally, various solvents were tested (for more details, see SI), but NMP with the addition of water (1.1 equiv) assured the highest yield (entry 13).

Detailed analysis of the reaction mixture revealed the formation of byproducts aside from desired product **7aa** under the optimized conditions (Scheme 2B). Acetophenone (**8a**, a side-product originating from epoxide **5a**) formed in 5% yield presumably via β-hydride elimination, while styrene (**10a**) is obtained in 30% yield from intermediate alcohol **9**.^49^ Finally, the reductive elimination in the nickel cycle may account for the observed small amount of biphenyl **11**.^50−52^ In order to gain more insight into the reaction mechanism, we carried out the reaction with enantioenriched styrene oxide (**5a**) under the optimized conditions. The expected coupling product **7aa** was obtained without any erosion of the stereocenter, which further supports the premise of the formation of the radical at the terminal position.

### Substrate Scope

**Scheme 5:**
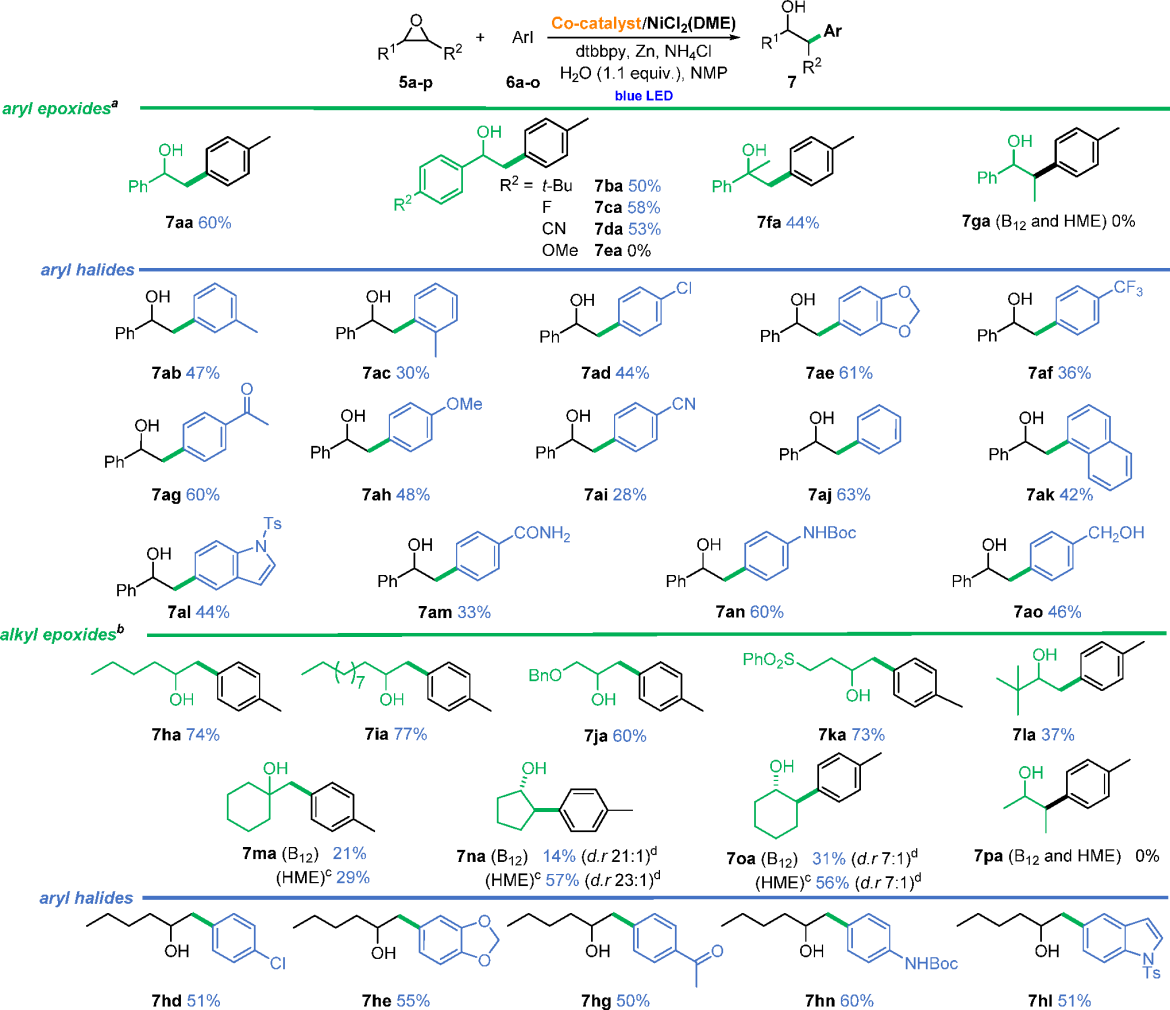
Vitamin B_12_-Catalyzed Ring-Opening Cross-Electrophile Coupling of (a) Aryl Epoxides and (b) Alkyl Epoxides^,^ Conditions: epoxide (0.2 mmol), aryl halide (1.5 equiv), B_12_ (5 mol %), NiCl_2_(DME) (20 mol %), Zn (1.5 equiv), NH_4_Cl (3 equiv), dtbbpy (40 mol %), H_2_O (1.1 equiv), dry NMP (*c* = 0.1 M), blue LED (single diode, 10 W), 30 min (for more details see SI). Blue LED (single diode, 3 W), 16 h. HME (5 mol %), acetone (*c* = 0.1 M), blue LED (single diode, 3 W), 16 h. Determined by GC.

With the optimized conditions in hand, we explored the scope and limitations of the developed method (Scheme 5).

Monosubstituted aryl epoxides **5a-f**, bearing both electron-withdrawing and electron-donating substituents, are, in general, well-tolerated and give corresponding products **7aa**–**fa** in 50–58% yields. However, 2-(4-methoxyphenyl)oxirane (**5e**) does not afford the desired product, as it decomposes rapidly under the present conditions. For disubstituted epoxides, the substitution pattern determines their reactivity. 1,1-Disubsituted epoxide **5f** leads to product **7fa** in 44% yield, while 1,2-disubstituted epoxide **5g** remains unreactive. As far as aryl halides are concerned, under standard conditions, both electron-donating and electron-withdrawing substituents are well tolerated, giving desired products **7ab**–**ao** in good to moderate yield (28–63%). Substitution at the 3- or 4-position of an aryl halide does not affect the reaction. In contrast, the more hindered halide, 2-iodotoluene (**6c**), undergoes coupling with styrene oxide (**5a**) in reduced reaction yield (compare **7aa**, **7ab**, and **7ac**). Although vitamin B_12_ exhibits exquisite reactivity in dehalogenation reactions,^11^ which often precludes the use of halogenated substrates, in our conditions product **7ad** forms in 44% yield. Importantly from the standpoint of possible further functionalizations, other functional groups (hydroxyl, carbonyl, protected amine) remain unaffected. Moreover, the representative heteroaryl halide, 5-iodo-(4-methylphenylsulfonyl)indole (**6l**), proves to be a viable substrate in the studied reaction without any further optimization needed. The developed method is also suitable for epoxides with aliphatic substituents (Scheme 5). The chain length does not impact the transformation’s outcome; the reaction with 1,2-epoxyhexane (**5h**) and 1,2-epoxydodecane (**5i**) gives products in 74% and 77% yield, respectively. We also found that aliphatic epoxide **5j**, possessing a protected primary hydroxyl group, could be converted into secondary alcohol **7ja** in 60% yield. The reaction with 4-(phenylsulfonyl)-1,2-epoxybutane (**5k**) gives corresponding product **7ka** in 73% yield. The potential use of aziridines as substrates was also investigated under the developed conditions, but only low yields of the respective products were obtained (see SI). Further studies on extending our methodology to other classes of heterocycles are currently ongoing in our laboratory.

Subsequently, the scope of aryl halides for the reaction with 1,2-epoxyhexane (**5h**) was explored. Substrates with both types of substituents—electron-rich and electron-deficient—on the aromatic ring afford the corresponding products **7hd**–**ol** in satisfactory yields. The *N*-Boc-protected amine, alkoxy, and carbonyl functionalities are well tolerated. The reaction with 1-chloro-4-iodobenzene (**6d**) leads to anticipated alcohol **7hd** in 51% yield. Similar to the reaction with aryl epoxides, indole-derived halide **6l** proved also a competent substrate, affording 1-(1-tosyl-1*H*-indol-5-yl)hexan-2-ol (**7hl**).

Compared to monosubstituted substrates, bicyclic epoxide **5o** was converted to the desired coupling product **7oa** with a significantly lower yield.^5^ Therefore, to gain a better understanding of how the reaction conditions affect the cross-electrophile coupling of disubstituted epoxides with aryl halides, additional experimental and theoretical studies were performed.

The use of hydrophobic analogue **3** instead of vitamin B_12_ (**1**) does not bring any substantial improvement (Table 2, entries 1, 2). However, with the simultaneous replacement of NMP with acetone, a 2-fold increase in the yield of **7oa** was observed (entry 3). A similar trend was also present for bicyclic epoxide **5n** and 1-oxaspiro[2.5]octane (**5m**), which provide considerably higher yields of desired alcohols **7na** and **7oa** in the presence of the HME/acetone system compared to vitamin B_12_/NMP. The main feature by which the studied Co catalysts differ is the presence/absence of the so-called “nucleotide loop” (the axial ligand located at the α face of the corrin ring with a 5′,6′-dimethylbenzimidazol (DMB) moiety) in addition to the replacement of amide into ester groups. Halpern et al. reported that in methyl malonyl-coenzyme A rearrangement switching between base-on and base-off forms of (CN)Cbl (**1**) changes the strength of the Co–C bond and hence the rate of its homolytic cleavage.^53^ To assess if the presence of this structural element impacts opening of bicyclic epoxides, we used cobalester **2** as a Co complex. This catalyst bears a nucleotide loop in its structure, but unlike parent vitamin B_12_, it dissolves well in both NMP and acetone, allowing for a direct comparison. A decisive solvent’s dependence was observed, with acetone assuring a higher yield than NMP (entries 4, 5), which corroborates the sole influence of the reaction medium. Likewise, the reaction catalyzed by cobinamide **4** (amide groups, no nucleotide loop) in NMP gives similar results to reactions catalyzed by other B_12_ derivatives in this solvent (entry 6).

**Table 2:**
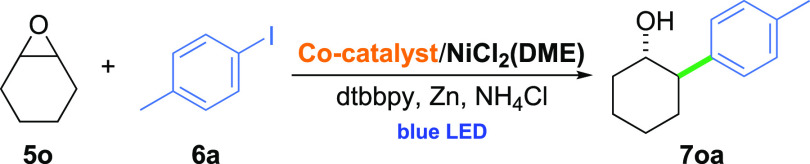
Influence of the Co Complex Structure on the Opening of Bicyclic Epoxides^a^

entry	solvent	catalyst		yield (%) **7oa**
1	NMP	B_12_		31
		CONH_2_	loop	
2	NMP	HME		26
		CO_2_Me	no loop	
3	**acetone**	HME^12^		**56**
		**CO**_**2**_**Me**	**no loop**	
4	acetone	cobalester^17^		55
		CO_2_Me	loop	
5	NMP	cobalester		32
		CO_2_Me	loop	
6	NMP	cobinamide^54^		35
		CONH_2_	no loop	

a Conditions: epoxide (**5o**, 0.2 mmol), aryl halide (**6a**, 1.5 equiv), Co catalyst (5 mol %), NiCl_2_(DME) (20 mol %), Zn (1.5 equiv), NH_4_Cl (3.0 equiv), dtbbpy (40 mol %), solvent (*c* = 0.1 M), blue LED (single diode, 3 W), 16 h.

**Chart 1:**
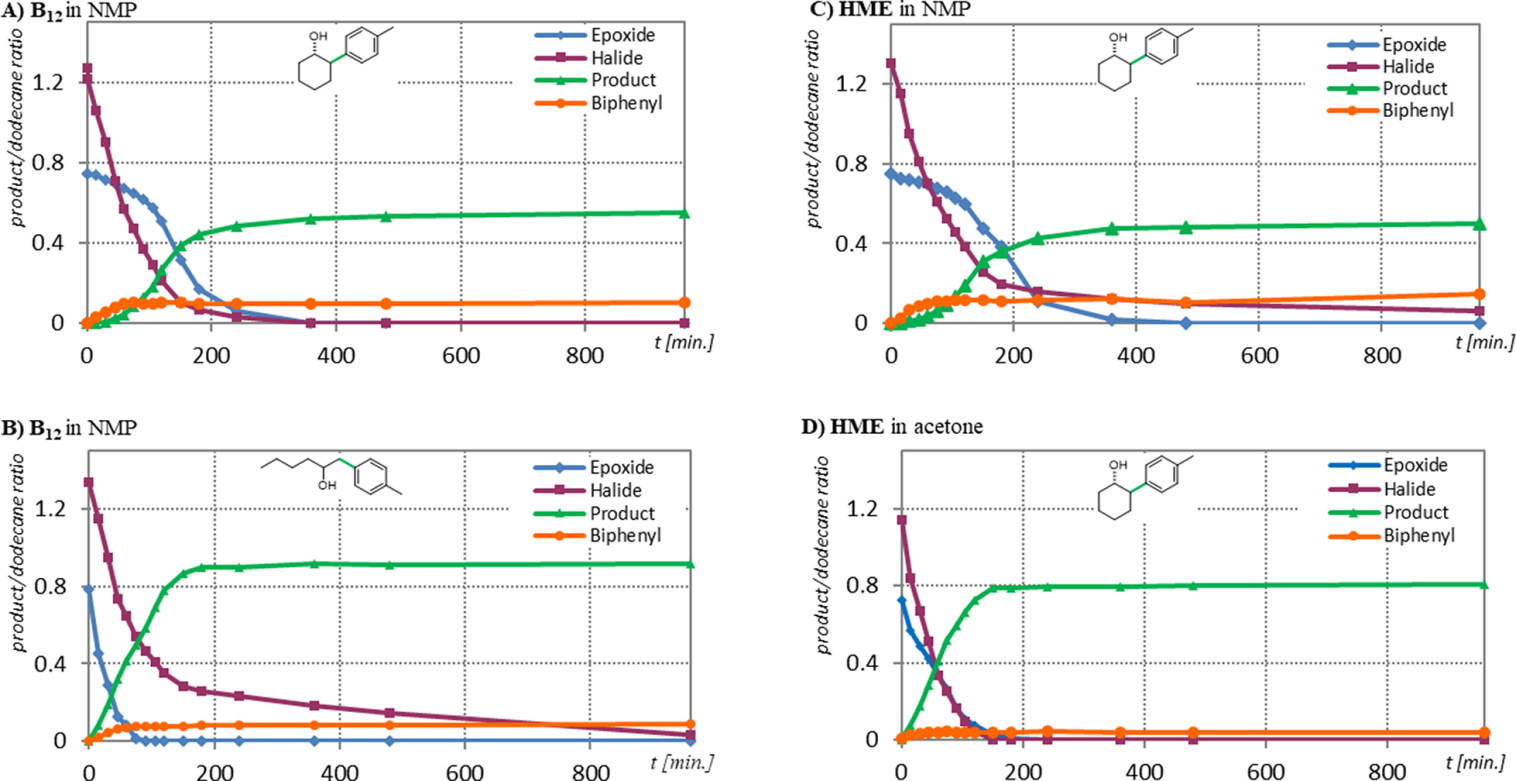
Kinetic Profile of the Opening of Bicyclic Epoxides (**6o**) (A, C, D) and Aliphatic Epoxide **6h** (B)a^,^b

Performed kinetic studies contributed to a better understanding of the observed differences. The rate of the bicyclic epoxide (**5o**) ring opening was found to vary significantly depending on the conditions applied (Chart 1). It takes 6 h to fully convert epoxide **5o** in both vitamin B_12_- and HME-catalyzed reactions as long as NMP is used as a solvent (compare fields A and C). On the other hand, the reaction in acetone provides full conversion in less than 3 h, which, presumably, translates to greater availability of alkyl radicals at a particular time (compare fields C and D).

a Conditions: epoxide (**5**, 0.2 mmol), aryl halide (**6a**, 1.5 equiv), Co catalyst (5 mol %), NiCl_2_(DME) (20 mol %), Zn (1.5 equiv), NH_4_Cl (3.0 equiv), dtbbpy (40 mol %), solvent (*c* = 0.1 M), blue LED (single diode, 3 W), 16 h, dodecane as an internal standard. b Measurements at *t* = 0 min refer to concentrations of compounds before mixing two solutions; see SI.

The reactivity of aryl iodide toward a Ni catalyst is assumed to be at a similar level, regardless of the conditions applied. Therefore, in NMP an insufficient concentration of alkyl radicals derived from bicyclic epoxides may promote Ni-catalyzed homocoupling of aryl iodide **6a**, an unproductive pathway, leading to biphenyl (**11a**) (fields A and C).^50,52^ This side-product is observed only in the presence of the Ni complex. The higher reactivity of monosubstituted aliphatic epoxide **5h** is reflected by its faster conversion as compared to bicyclic epoxide **5o** (compare fields A and B). In their case, the catalyst and the solvent do not affect the reaction; yields are almost identical (74%) in both cases.

**Figure 2:**
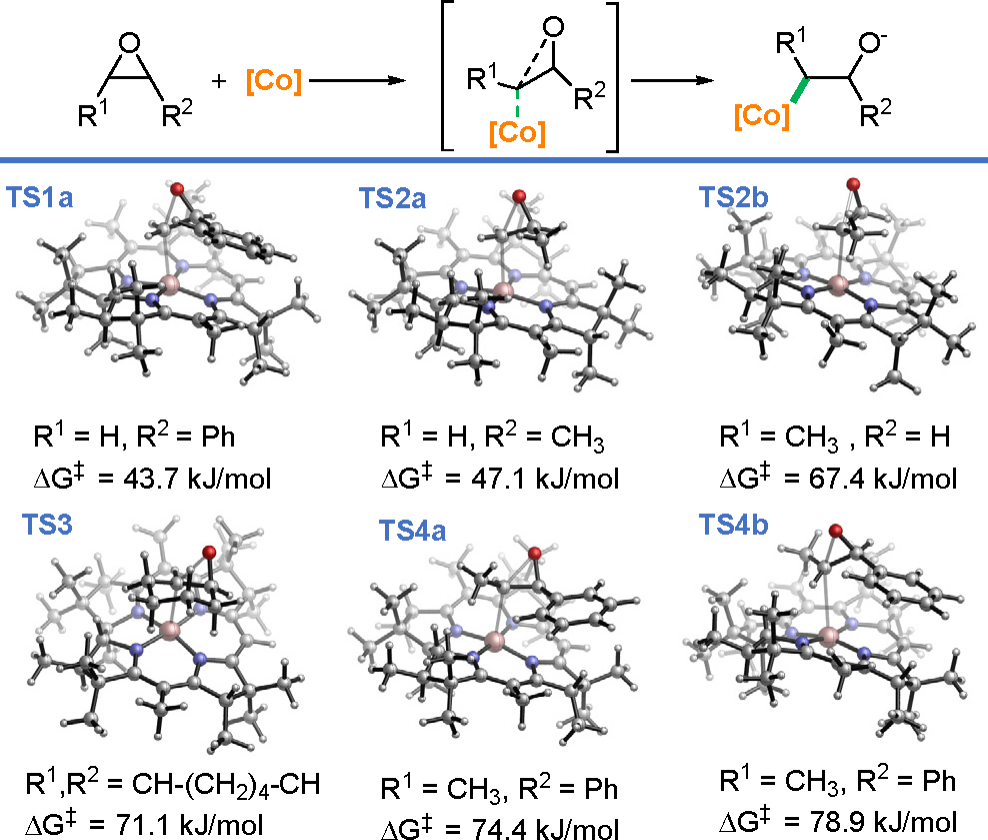
Gibbs free energy barriers for the opening of epoxides with the Co(I)-corrin complex calculated at the BP86-D3/6-311++G(2df,p)/SMD(acetone)//BP86-D3/6-31G(d)/SMD(acetone) level of theory.

The observed reactivity pattern corresponds well with the calculated barriers for the nucleophilic opening of the epoxides with the Co(I)-corrin complex (Figure 2). In general, the Gibbs free energy of activation for the reaction of aryl- and alkyl-monosubstituted epoxides (**TS1a** and **TS2a**, 43.7 and 47.1 kJ/mol for Ph- and Me-substituted, respectively) is smaller than for more sterically demanding 1,2-disubstituted epoxides (**TS3** and **TS4**, >70 kJ/mol). Nevertheless, bicyclic substrates **5n**,**o** provide desired products **7na** and **7oa** in good yields, while epoxide **5g**, for which the activation barrier is ∼3 kJ/mol higher, remains unreactive (**TS3** versus **TS4a**) regardless of the *Z*/*E* configuration of the epoxide (**TS4a** vs **TS4b**, Δ*G*^⧧^ = 74.4 vs 78.9 kJ/mol). Additionally, the observed regioselectivity is well reflected by the energetically favored attack of the Co(I)-corrin on the less hindered side on propylene oxide (a model used for an alkyl epoxide, **TS2a** vs **TS2b**, Δ*G*^⧧^ = 47.1 vs 67.4 kJ/mol).

## Conclusions

We have developed a highly regioselective, Co/Ni-catalyzed ring-opening reaction of epoxides with aryl halides. The scope of our method has been demonstrated in a broad range of aliphatic and aromatic epoxides. Gratifyingly, these include cyclic and disubstituted epoxides even though the Gibbs free energy of activation for their reactions are higher than for alkyl- and aryl-monosubstituted substrates. Due to the mild reaction conditions, a wide range of functional groups is well tolerated.

Only the cooperation of vitamin B_12_ as a Co catalyst with Ni catalysis assures high regioselectivity of the cross-electrophile coupling. The crucial ring opening by the Co(I) complex occurs from the less hindered side, leading to linear products.

This new methodology complements the existing approaches providing access to a diverse array of substituted alcohols, which are valuable feedstock chemicals in synthetic and medicinal chemistry. Consequently, it closes the gap in the synthesis of linear and branched alcohols via cross-electrophile coupling; they are now accessible from both alkyl and aryl epoxides.

## Supporting Information Available

Experimental details and procedures, optimization studies, mechanistic experiments, DFT, and spectral data for all new compounds (PDF)
