# Supplementary material for: Cobalt Catalyst Determines Regioselectivity in Ring Opening of Epoxides with Aryl Halides
Source: J Am Chem Soc. 2021 Jun 3;143(25):9368–76. doi: 10.1021/jacs.1c00659 (PMC8297733; doi:10.1021/jacs.1c00659)

Supporting Information

Cobalt Catalyst Determines Regioselectivity in Ring-opening of Epoxides with Aryl Halides

Aleksandra Potrząsaj, Mateusz Musiejuk, Wojciech Chaładaj,

Maciej Giedyk*, Dorota Gryko*

Institute of Organic Chemistry Polish Academy of Science
Kasprzaka 44/52, 01-224 Warsaw, Poland

e-mail: dorota.gryko@icho.edu.pl

Table of Contents

1. General Information **4**
2. Setup for photoreactions **5**
3. Full Optimization of the Reaction Parameters **6**
3.1 Background experiments 6
3.2 The influence of light on the model reaction 6
3.3 Optimization of a solvent for HME (**3**) – catalyzed reaction 6
3.4 Screening of cobalt catalysts 7
3.5 Optimization of a solvent for B_{12} (**1**) – catalyzed reaction 8
3.6 B_{12} – catalyst loading 8
3.7 The influence of Zn and NH_4Cl amounts 8
3.8 Screening of Ni – catalysts 9
3.9 Screening of ligands 9
3.10 Optimization of the substrates ratio 10
3.11 Concentration of styrene oxide (5a) 10
3.12 The influence of water on the model reaction 10
4. General Procedures **11**
4A General Procedure for opening aryl epoxides 11
4B General Procedure for opening aliphatic epoxides 11
4C General Procedure for opening cyclic epoxides catalyzed by B_{12} 11
4D General Procedure for opening cyclic epoxides catalyzed by HME 12
4.1 General procedure - notes 12
5. Scope and characterization of new compounds – epoxides and aziridines **13**
6. Mechanistic consideration **30**
6.1 Proposed mechanism 30
6.2 Mass Spectrometry studies 30
6.3 Studies of regioselectivity 31
6.4 Stereoselectivity studies 34
6.5 DFT Calculations 35
6.6 Kinetic studies for model reaction 82
6.7 Reactions in deuterated solvents 82
7. References **85**
8. NMR Spectra **88**
1-phenyl-2-(4-tolyl)ethan-1-ol (**7aa**) 88
1-(4-(*tert*-butyl)phenyl)-2-(4-tolyl)ethan-1-ol (**7ba**) 89
1-(4-fluorophenyl)-2-(4-tolyl)ethan-1-ol (**7ca**) 90
4-(1-hydroxy-2-(4-tolyl)ethyl)benzonitrile (**7da**) 92
2-phenyl-1-(4-tolyl)propan-2-ol (**7fa**) 93
1-phenyl-2-(3-tolyl)ethan-1-ol (**7ab**) 94
1-phenyl-2-(2-tolyl)ethan-1-ol (**7ac**) 95
2-(4-chlorophenyl)-1-phenylethan-1-ol (**7ad**) 96
2-(benzo[*d*][1,3]dioxol-5-yl)-1-phenylethan-1-ol (**7ae**) 97
1-phenyl-2-(4-(trifluoromethyl)phenyl)ethan-1-ol (**7af**) 98
1-(4-(2-hydroxy-2-phenylethyl)phenyl)ethan-1-one (**7ag**) 100
2-(4-methoxyphenyl)-1-phenylethan-1-ol (**7ah**) 101
4-(2-hydroxy-2-phenylethyl)benzonitrile (**7ai**) 102
1,2-diphenylethan-1-ol (**7aj**) 103
2-(naphthalen-1-yl)-1-phenylethan-1-ol (**7ak**) 104
1-phenyl-2-(1-tosyl-1*H*-indol-5-yl)ethan-1-ol (**7al**) 105
4-(2-hydroxy-2-phenylethyl)benzamide (**7am**) 106
tert-butyl (4-(2-hydroxy-2-phenylethyl)phenyl)carbamate (**7an**) 107

2-(4-(hydroxymethyl)phenyl)-1-phenylethan-1-ol (**7ao**) 108
1-(4-tolyl)hexan-2-ol (**7ha**) 109
1-(4-tolyl)dodecan-2-ol (**7ia**) 110
1-(benzyloxy)-3-(4-tolyl)propan-2-ol (**7ja**) 111
4-(phenylsulfonyl)-1-(4-tolyl)butan-2-ol (**7ka**) 112
3,3-dimethyl-1-(4-tolyl)butan-2-ol (**7la**) 113
1-(4-methylbenzyl)cyclohexan-1-ol (**7ma**) 114
(1*S*,2*R*)-2-(4-tolyl)cyclopentan-1-ol (**7na**) 115
(1*S*,2*R*)-2-(4-tolyl)cyclohexan-1-ol (**7oa**) 116
1-(4-chlorophenyl)hexan-2-ol (**7hd**) 117
1-(benzo[*d*][1,3]dioxol-5-yl)hexan-2-ol (**7he**) 118
1-(4-(2-hydroxyhexyl)phenyl)ethan-1-one (**7hg**) 119
tert-butyl (4-(2-hydroxyhexyl)phenyl)carbamate (**7hn**) 120
1-(1-tosyl-1*H*-indol-5-yl)hexan-2-ol (**7hl**) 121
4-methyl-*N*-(2-phenyl-(*p*-tolyl)ethyl)benzenosulphonamide (**S23**) and 4-methyl-*N*-(1-phenyl-2-(*p*-tolyl)ethyl)benzenosulfonamide (**S24**) 122
4-methyl-*N*-(2-(*p*-tolyl)cyclopentyl)benzenosulfonamide (**S25**) 123
4-methyl-*N*-(1-(*p*-tolyl)hexan-2-yl)benzenosulfonamide (**S26**) 124

1. General Information

General Procedures. Unless otherwise noted, reactions were performed without the exclusion of air or moisture. All the photochemical reactions were performed in 10 mL glassy vials sealed with aluminum caps containing a rubber septa. Reactions were monitored by gas chromatography (GC, specification below) or thin-layer chromatography (TLC) on Merck silica gel (GF254, 0.20 mm thickness), visualizing with UV-light, potassium permanganate ($KMnO_4$), or ceric ammonium molybdate (CAM)/Hanessian's stain. Colum chromatography was performed using Merck silica gel 60 (230-400 mesh). GC yields were calibrated using dodecane as an internal standard.

Materials. Commercial reagents and solvents were purchased from Sigma-Aldrich, Acros Organics, Alfa Aesar, Fluorochem, and TCI, and used as received unless otherwise noted. Dry solvents: dimethyl sulfoxide (DMSO), dichloromethane (CH_2Cl_2), tetrahydrofuran (THF), acetonitrile (CH_3CN) were taken from *Solvent Purification System* (SPS). Deuterated solvents ($CDCl_3$, $(CD_3)_2CO$ and MeOD) were purchased from Eurisotop. Amidine ligands (**S18**)[1], 5-iodo-1-(4-methylphenylsulfonyl)indole (**6l**)[2], 4-*tert*-butylphenylethylene oxide (**5b**)[3], 4-cyano-butylphenylethylene oxide (**5d**)[4], 4-(phenylsulfonyl)-1,2-epoxybutane (**5k**)[5], 1-oxaspiro(2.5)octane (**5m**)[6], *N*-(*p*-tolylsulfonyl)-2-phenylaziridine (**S20**)[7], N-tosyl-6-azabicyclo[3.1.0]hexane (**S21**)[7], 2-butyl-*N*-tosylaziridine (**S22**)[7] and catalysts: $NiCl_2$(dtbbpy)[8], (CN)(H_2O)Cby(OMe)$_7$ (**3**),[9] Co(dmgH)$_2$py^{i}Pr (**S6**)[10] were synthesized according to literature procedures.

Before the reaction, zinc was activated by the following method: a) washing with 10% HCl, b) grinding, c) washing with H_2O, EtOH, and Et_2O, d) drying in a vacuum.

Instrumentation.

- NMR Spectroscopy: ^{1}H and ^{13}C NMR spectra were recorded at 25 °C on a Bruker 400 MHz, 500 MHz or Varian 600 MHz instrument with TMS as an internal standard. NMR chemical shifts are reported in ppm and referenced to the residual solvent peak of $CDCl_3$ (7.26 ppm - ^{1}H NMR and 77.16 ppm - ^{13}C NMR). Multiplicities are indicated by singlet (s), doublet (d), triplet (t), quartet (q), multiplet (m) and broad (br). Coupling constants (*J*) are reported in Hertz. All data analysis was performed using MestReNova software package.

- GC/MS Chromatography: GC-MS analyses were performed using Shimadzu GCMS-QP2010 SE gas chromatograph with FID detector and Zebron ZB 5MSi column.

- Elemental Analysis: Elemental analysis (N, H, C, S) were performed on PERKIN-ELMER 240 Elemental Analyzer.

- High Resolution Mass Spectrometry: High-resolution mass spectra (HRMS) were recorded on a Waters AutoSpec Premier instrument using electron ionization (EI) or a Waters SYNAPT G2-S HDMS instrument using electrospray ionization (ESI) with time of flight detector (TOF).

- Low Resolution Mass Spectrometry: Low-resolution mass spectra (LRMS) were recorded on an Applied Biosystems API 365 mass spectrometer using electrospray ionization (ESI) technique.

- Melting points: Melting points were recorded on a Marienfeld MPM-H2 melting point apparatus and are uncorrected.

- High-performance liquid chromatography (HPLC): High-performance liquid chromatography (HPLC) was performed on Daicel Chiralpak ID-H column (250 mm x 4.6 mm inside diameter) using a mixture of AcOEt in hexane (10%) as a mobile phase.

2. Setup for photoreactions

Reactions were performed in three different types of photoreactors (Figure 2.1)

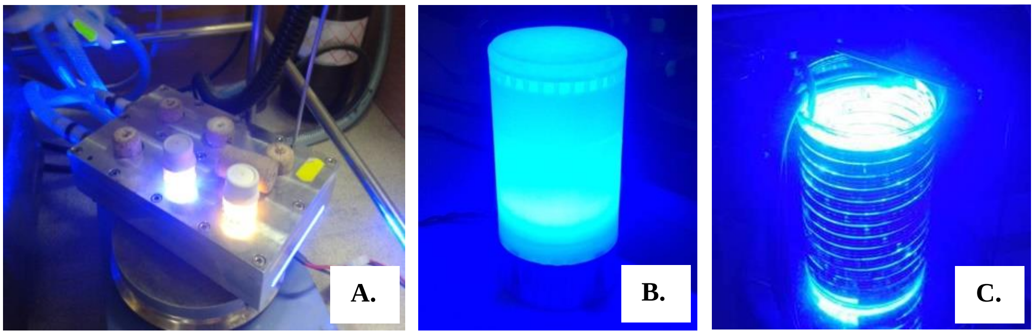

Figure 2.1 Photograph of a photoreactors

Characteristic of photoreactors:

A. Blue LED; single diode 3 W, controlled by mini chiller set up at 33 ˚C, reactions in 10 mL vials;

B. Blue LED; single diode 10 W, fan cooling, reactions in 10 mL vials;

C. Blue LED; LED tape 9 W, fan cooling, reactions in a test tubes.

3. Full Optimization of the Reaction Parameters

Model reaction:

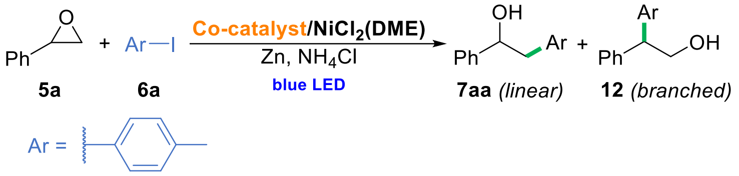

Reaction conditions: styrene oxide (**5a**) (0.2 mmol, 1 equiv.), 4-iodotoluene (**6a**) (1.5 equiv.), Zn (3 equiv.), NH_4Cl (3 equiv.), B_{12} (**1**) (5 mol%), $NiCl_2$(DME) (20 mol%), dtbbpy (40 mol%), water (1.1 equiv.), dry NMP (c = 0.1 M), blue LED, 30 min.

3.1 Background experiments

Entry	Deviation from the Standard Conditions	Yield of **7aa** [%]
**1**	**none**	**60**
2	No Co-cat., No NH_4Cl	7 and 28[a]
3	No Co-cat., No Ni-cat., No ligand	0
4	No Ni-cat., No ligand	0
5	No light	<10%
6[b]	No light, 50 °C	26
7[b]	No light, 80 °C	28
8	Air atmosphere	product not observed

Reaction conditions: styrene oxide (**5a**) (0.2 mmol, 1 equiv.), 4-iodotoluene (**6a**) (1.5 equiv.), Zn (3 equiv.), NH_4Cl (3 equiv.), B_{12} (**1**) (5 mol%), $NiCl_2$(DME) (20 mol%), dtbbpy (40 mol%), water (1.1 equiv.), dry NMP (c = 0.1 M), blue LED, 30 min. [a] - product branched formed – **12,** [b] – reaction time (16 h)

3.2 The influence of light on the model reaction

Entry	Light	Yield of **7aa** [%]
1	Violet LEDs (tape)	23
2	White LEDs (tape)	16
3	Green LEDs (tape)	24
4	Blue LED (single diode, 3 W)	30
**5**	**Blue LED** (single diode, 10 W)	**38**

Reaction conditions: styrene oxide (**5a**) (0.2 mmol, 1 equiv.), 4-iodotoluene (**6a**) (1.5 equiv.), Zn (3 equiv.), NH_4Cl (3 equiv.), HME (**3**) (5 mol%), $NiCl_2$(DME) (20 mol%), dtbbpy (40 mol%), acetone (2 mL), 16 h

3.3 Optimization of a solvent for HME (3) – catalyzed reaction

Entry	Solvent	Yield of **7aa** [%]
1	CH_3CN	32
2	DMSO	7
3	Methanol	19
**4**	**Aceton**	**38**
5	DMF	21
6	THF	27

Reaction conditions: styrene oxide (**5a**) (0.2 mmol, 1 equiv.), 4-iodotoluene (**6a**) (1.5 equiv.), Zn (3 equiv.), NH_4Cl (3 equiv.), HME (**3**) (5 mol%), $NiCl_2$(DME) (20 mol%), dtbbpy (40 mol%), solvent (2 mL), Blue LED, 16 h

3.4 Screening of the cobalt catalysts

Entry	Catalyst	Yield of **7aa** [%]
1	3	38
2	S1	10
**3**[c]	**1**	**42**
4	2	31
5	S5	5
6	S2	7
7	S3	8
8	S4	7
9	S6	11

Reaction conditions: styrene oxide (**5a**) (0.2 mmol), 4-iodotoluene (**6a**) (1.5 equiv.), Zn (3 eq.), NH_4Cl (3 equiv.), Co-catalyst (5 mol%), $NiCl_2$(DME) (20 mol%), dtbbpy (40 mol%), Acetone (2 mL), Blue LEDs, 16 h
[c]Conditions: styrene oxide (**5a**) (0.2 mmol, 1 equiv.), 4-iodotoluene (**6a**) (1.5 equiv.), Zn (3 equiv.), NH_4Cl (3 equiv.), $NiCl_2$(DME) (20 mol%), dtbbpy (40 mol%), dry NMP (2 mL), Blue LED, 30 min.

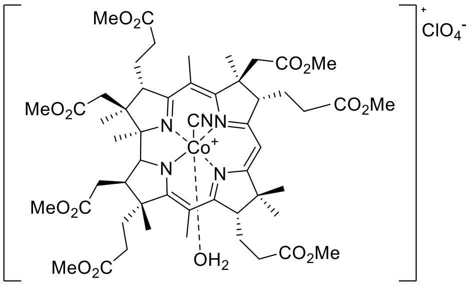

Aqua(cyano)heptamethyl cobyrinate (3)

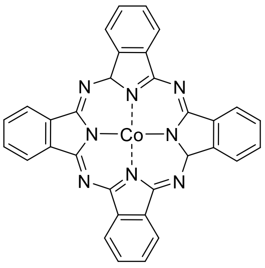

Cobalt(II) phthalocyanine (S1)

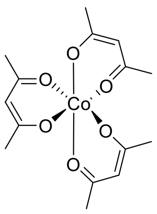

Cobalt(III) acetyloacetonate (S4)

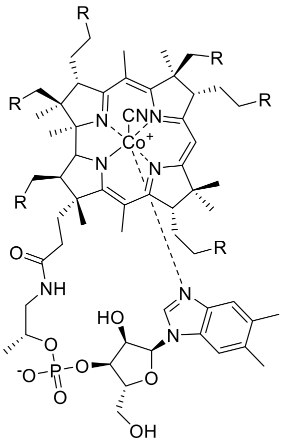

Vitamin B_{12} (1), R = $CONH_2$
Cobalester (2), R = CO_2Me

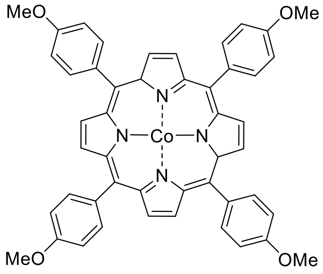

5,10,15,20-Tetrakis(4-methoxyphenyl)-21*H*,23*H*-porphine cobalt (II) (S2)

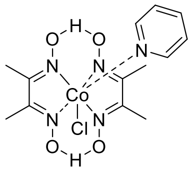

Chloro(pyridine)bis (dimethylglyoximato)cobalt(III) (S3)

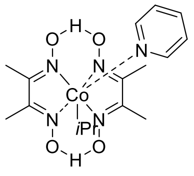

***i*-Propyl(pyridine)bis (dimethylglyoximato)cobalt(III) (S6)**

$CoCl_2$
Cobalt(II) chloride (S5)

3.5 Optimization of a solvent for B_{12} (1) – catalyzed reaction

Entry	Solvent	Yield of **7aa** [%]
**1**	**NMP**	**42**
2	Methanol	28
3	DMA	6
4	DMF	20
5	Acetone:Methanol 1:1	27
6	THF:H_2O (1:1)	4

Reaction conditions: styrene oxide (**5a**) (0.2 mmol), 4-iodotoluene (**6a**) (1.5 equiv.), Zn (3 equiv.), NH_4Cl (3 equiv.), B_{12} (**1**) (5 mol%), $NiCl_2$(DME) (20 mol%), dtbbpy (40 mol%), solvent (2mL), Blue LED, 30 min.

3.6 B_{12} – catalyst loading

Entry	Catalyst loading [%]	Yield of **7aa** [%]
1	2.5	33
**2**	**5**	**42**
3	7.5	40
4	10	38

Reaction conditions: styrene oxide (**5a**) (0.2 mmol), 4-iodotoluene (**6a**) (1.5 equiv.), Zn (3 equiv.), NH_4Cl (3 equiv.), B_{12} (**1**), $NiCl_2$(DME) (20 mol%), dtbbpy (40 mol%), dry NMP (2 mL), Blue LED, 30 min.

3.7 The influence of Zn and NH_4Cl amounts

Entry	Zn (equiv.)	NH_4Cl (equiv.)	Yield of **7aa** [%]
1	3	3	42
2	3	1	34
3	6	3	22
4	3	5	20
5	3	4	31
6	3	1.5	42
7	3	2	46
**8**	**1.5**	**3**	**53**
9	1	3	32
10	1	2	30

Reaction conditions: styrene oxide (**5a**) (0.2 mmol), 4-iodotoluene (**6a**) (1.5 equiv.), B_{12} (**1**) (5mol%), $NiCl_2$(DME) (20 mol%), dtbbpy (40 mol%), dry NMP (2 mL), Blue LED, 30 min.

3.8 Screening of Ni – catalysts

Entry	Catalyst	Catalyst loading [mol%]	Yield of **7aa** [%]
**1**	**$NiCl_2$(DME)**	**20**	**53**
2	$NiBr_2$(DME)	20	43
3	$NiCl_2$	20	36
4	$NiBr_2$	20	43
5	NiI_2	20	40
6	$Ni(TMHD)_2$	20	20
7	$Ni(acac)_2$	20	33
8	$Ni(OAc)_2 \cdot 4H_2O$	20	31
9	$Ni(OTf)_2$	20	39
10[d]	$NiCl_2$(dtbbpy)	20	45
11[d]	$NiCl_2$(dtbbpy)	10	46
12	$NiCl_2$(DME)	10	37
13	$NiCl_2$(DME)	40	50

Reaction conditions: styrene oxide (**5a**) (0.2 mmol, 1 equiv.), 4-iodotoluene (**6a**) (1.5 equiv.), B_{12} (**1**) (5mol%), Zn (1.5 equiv.), NH_4Cl (3 equiv.), dtbbpy (40 mol%), dry NMP (2 mL), Blue LED, 30 min.; [c] – without ligand

3.9 Screening of ligands

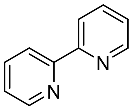

S7 30%

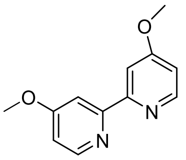

S8 52%

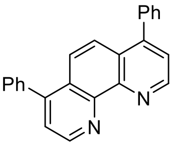

S9 34%

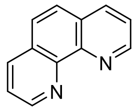

S10 24%

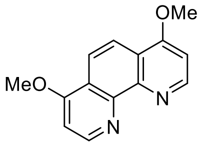

S11 38%

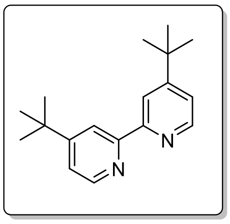

S12 53%

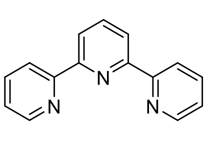

S13 13 %

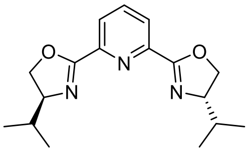

S14 0%

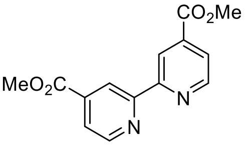

S15 26%

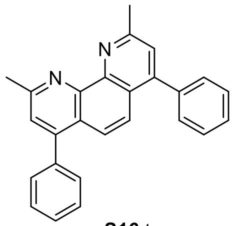

S16 traces

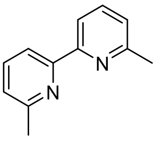

S17 traces

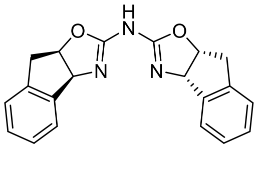

S18 0%

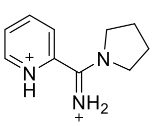

S19 41%

The amount of the ligand added:

Entry	Ligand [mol%]	Yield of **7aa** [%]
1	10	39
2	15	41
3	20	36
4	30	37
**5**	**40**	**53**
6	50	39

Reaction conditions: styrene oxide (**5a**) (0.2 mmol, 1eq.), 4-iodotoluene (**6a**) (1.5 eq.), B_{12} (**1**) (5mol%), Zn (1.5 equiv.), NH_4Cl (3 equiv.) $NiCl_2$(DME) (20 mol%), dry NMP (2 mL), Blue LED, 30 min

3.10 Optimization of the substrates ratio

Entry	**5a** (equiv.)	**6a** (equiv.)	Yield of **7aa** [%]
**1**	**1**	**1.5**	**53**
2	1.5	1	43
3	1	1	40
4	1	2	36
5	2	1	45
6	1	3	42

Reaction conditions: styrene oxide (**5a**) (0.2 mmol), 4-iodotoluene (**6a**), B_{12} (**1**) (5mol%), Zn (1.5 equiv.), NH_4Cl (3 equiv.) $NiCl_2$(DME) (20 mol%), dtbbpy (40 mol%), dry NMP (2 mL), Blue LED, 30 min.

3.11 Concentration of styrene oxide (5a)

Entry	[mol/dm^3]	Yield of **7aa** [%]
1	0.05	18
**2**	**0.1**	**53**
3	0.2	36

Reaction conditions: styrene oxide (**5a**) (0.2 mmol, 1 equiv.), 4-iodotoluene (**6a**) (1.5 equiv.), B_{12} (**1**) (5 mol%), Zn (1.5 equiv.), NH_4Cl (3 equiv.), $NiCl_2$(DME) (20 mol%), dtbbpy (40 mol%), dry NMP (2 mL), Blue LED, 30 min.

3.12 The influence of water on the model reaction

Entry	H_2O (equiv.)	Yield of **7aa** [%]
1	0.3	50
2	0.6	42
**3**	**1.1**	**60**
4	2.2	60
5	4.4	59

Reaction conditions: styrene oxide (**5a**) (0.2 mmol), 4-iodotoluene (**6a**), B_{12} (**1**) (5 mol%), Zn (1.5 equiv.), NH_4Cl (3 equiv.), $NiCl_2$(DME) (20 mol%), dtbbpy (40 mol%), dry NMP (2 mL), Blue LED, 30 min.

4. General Procedures

***A**. General procedure for aryl epoxide:*

Each reaction was prepared in two glass vials (10 mL) sealed with aluminum caps with a rubber septa. The first one, equipped with a magnetic stirring bar, was charged with activated Zn^0 dust (20 mg, 0.3 mmol, 1.5 equiv.), NH_4Cl (32 mg, 0.6 mmol, 3 equiv.), and catalyst **1** (5 mol%, 13.5 mg). To the second vial were consecutively added dttbpy (40 mol%, 21 mg), aryl iodide (65 mg, 0.3 mmol, 1.5 equiv.), and $NiCl_2$(DME) (20 mol%, 9 mg). Then dry NMP (1 mL containing water – 0.55 equiv.) was added to each vial. The resulting mixture was degassed by purging with argon with simultaneous sonication in an ultrasonic bath for 15 min. An epoxide (0.2 mmol, 1.0 equiv.) was added dropwise *via* a syringe to the first vial and then the solution from the second was transferred to the first vial. The resulting mixture was irradiated with blue LED light (single diode, 10 W; λ = 460 nm) for 30 min. at room temperature. The resulting mixture was diluted with AcOEt, washed with water (10 mL) and brine (15 mL). The organic phase was dried over Na_2SO_4, then filtered through the cotton wool and concentrated *in vacuo*. A crude product was purified by means of column chromatography.

***B**. General procedure for aliphatic epoxide:*

Each reaction was prepared in two glass vials (10 mL) sealed with aluminum caps with a rubber septa. The first one, equipped with a magnetic stirring bar, was charged with activated Zn^0 dust (20 mg, 0.3 mmol, 1.5 equiv.), NH_4Cl (32 mg, 0.6 mmol, 3 equiv.), and catalyst **1** (5 mol%, 13.5 mg). To the second vial was consecutively added dttbpy (40 mol%, 21 mg), aryl iodide (65 mg, 0.3 mmol, 1.5 equiv.), and $NiCl_2$(DME) (20 mol%, 9 mg). Then dry NMP (1 mL containing water – 0.55 equiv.) was added to each vial. The resulting mixture was degassed by purging with argon with simultaneous sonication in an ultrasonic bath for 15 min. An epoxide (0.2 mmol, 1.0 equiv.) was added dropwise *via* a syringe to the first vial and then the solution from the second was transferred to the first vial. The resulting mixture was irradiated with blue LED light (single diode, 3 W; λ = 460 nm) for 16 h at room temperature. The resulting mixture was diluted with AcOEt, washed with water (10 mL) and brine (15 mL). The organic phase was dried over Na_2SO_4, then filtered through the cotton wool and concentrated *in vacuo*. A crude product was purified by means of column chromatography.

***C**. General procedure for cyclic epoxide with B_{12}:*

Each reaction was prepared in two glass vials (10 mL) sealed with aluminum caps with a rubber septa. The first one, equipped with a magnetic stirring bar, was charged with activated Zn^0 dust (20 mg, 0.3 mmol, 1.5 equiv.), NH_4Cl (32 mg, 0.6 mmol, 3 equiv.), and catalyst **1** (5 mol%, 13.5 mg). To the second vial was consecutively added dttbpy (40 mol%, 21 mg), aryl iodide (65 mg, 0.3 mmol, 1.5 equiv.), and $NiCl_2$(DME) (20 mol%, 9 mg). Then dry NMP (1 mL containing water – 0.55 equiv.) was added to each vial. The resulting mixture was degassed by purging with argon with simultaneous sonication in an ultrasonic bath for 15 min. An epoxide (0.2 mmol, 1.0 equiv.) was added dropwise *via* a syringe to the first vial and then the solution from the second was transferred to the first vial. The resulting mixture was irradiated with blue LED light (single diode, 3 W; λ = 460 nm) for 16 h at room temperature. The resulting mixture was diluted with AcOEt, washed with water (10 mL) and brine (15 mL). The organic phase was dried over Na_2SO_4, then filtered through the cotton wool and concentrated *in vacuo*. A crude product was purified by means of column chromatography.

D. General procedure for cyclic epoxide with HME:

Each reaction was prepared in two glass vials (10 mL) sealed with aluminum caps with a rubber septa. The first one, equipped with a magnetic stirring bar, was charged with activated Zn^0 dust (20 mg, 0.3 mmol, 1.5 equiv.), NH_4Cl (32 mg, 0.6 mmol, 3 equiv.), and catalyst **3** (5 mol%, 11 mg). To the second vial was consecutively added dttbpy (40 mol%, 21 mg), aryl iodide (65 mg, 0.3 mmol, 1.5 equiv.), and $NiCl_2$(DME) (20 mol%, 9 mg). Then acetone (1 mL) was added to each vial. The resulting mixture was degassed by purging with argon with simultaneous sonication in an ultrasonic bath for 5 min. An epoxide (0.2 mmol, 1.0 equiv.) was added dropwise *via* a syringe to the first vial and then the solution from the second was transferred to the first vial. The resulting mixture was irradiated with blue LED light (single diode, 3 W; λ = 460 nm) for 16 h at room temperature. The resulting mixture was diluted with AcOEt, filtered through the cotton wool and concentrated *in vacuo.* A crude product was purified by means of column chromatography.

4.1 Note:

- The reaction can be easily monitored by TLC chromatography (AcOEt/Hexane) using UV visualization, $KMnO_4$ or the Hanessian's stain;
- Reactions require using activated zinc (unactivated zinc gives a low yield);
- The mixture containing Zn, NH_4Cl, Cobalt-catalyst (B_{12} or HME) and a solvent should turned from red to dark green and finally brown. The color indicates the reduction of the cobalt from Co(III) to Co(I) oxidation state (see pictures below);
- If the color of the reaction does not change (from red to dark brown/green), we highly recommend to repeat zinc activation step;
- We highly advise to use two vials for two reasons: a) you can check by the color if the cobalt has been reduced, b) the reaction prepared in one vial gives product in slightly decreased yield.

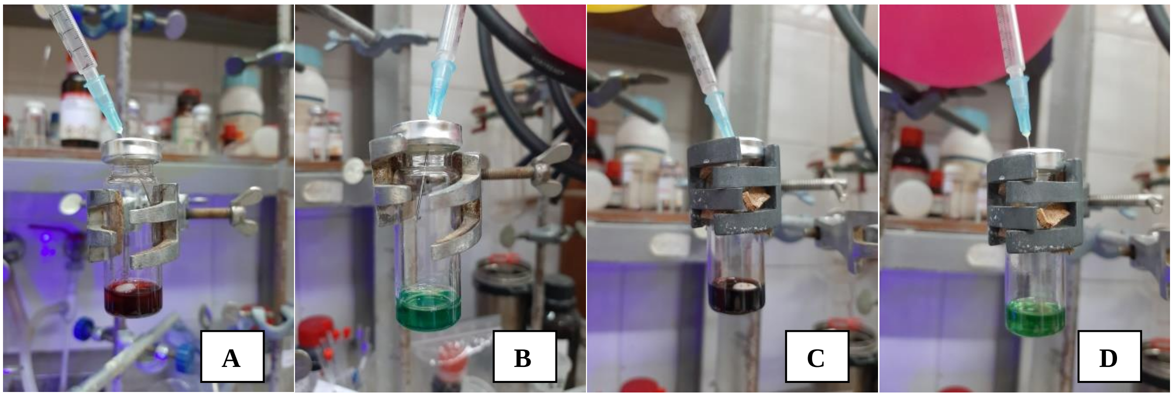

4.1 A) The vial containing B_{12} (5 mol%), NH_4Cl (3 equiv.), Zn (1.5 equiv.) – before degassing (**color: red**); B) The vial containing $NiCl_2$(DME) (20 mol%), dtbbpy (40 mol%), aryl halide (1.5 equiv.) – before degassing (**color: bluish green**); C) The vial containing B_{12} (5 mol%), NH_4Cl (3 equiv.), Zn (1.5 equiv.) – after degassing (**color: dark brown**); D) The vial containing $NiCl_2$(DME) (20 mol%), dtbbpy (40 mol%), aryl halide (1.5 equiv.) – after degassing (**color: light green**).

5. Scope and characterization of new compounds

1-phenyl-2-(*p*-tolyl)ethan-1-ol (7aa)

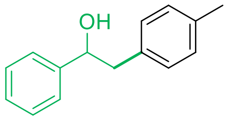

Following the general procedure **A** compound **7aa** was obtained from styrene oxide (**5a**) (24 mg, 0.20 mmol) and 4-iodotoluene (**6a**) (65 mg, 0.30 mmol). The crude product was purified by column chromatography (5:95 AcOEt/Hexane) to afford 25 mg of 1-phenyl-2-(*p*-tolyl)ethan-1-ol (**7aa**) as white solid, (yield = **60%**).

NMR data matched those reported in the literature.[11]

^{1}H NMR (400 MHz, CDCl$_3$): δ 7.39 – 7.26 (m, 5H), 7.13 – 7.07 (m, 4H), 4.88 (dd, *J* = 8.6, 4.7 Hz, 1H), 3.02 (dd, *J* = 13.7, 4.7 Hz, 1H), 2.94 (dd, *J* = 13.7, 8.6 Hz, 1H), 2.33 (s, 3H), 1.93 (s, 1H).

^{13}C NMR (100 MHz, CDCl$_3$): δ 143.9, 136.2, 134.9, 129.4, 129.2, 128.4, 127.6, 125.9, 75.4, 45.7, 21.0.

1-(4-(*tert*-butyl)phenyl)-2-(*p*-tolyl)ethan-1-ol (7ba)

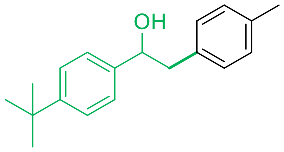

Following the general procedure **A** compound **7ba** was obtained from 4-*tert*-butylphenylethylene oxide (**5b**) (35 mg, 0.20 mmol) and 4-iodotoluene (**6a**) (65 mg, 0.30 mmol). The crude product was purified by column chromatography (5:95 AcOEt/Hexane) to afford 27 mg of 1-(4-(*tert*-butyl)phenyl)-2-(*p*-tolyl)ethan-1-ol (**7ba**) as white solid, (yield = **50%**).

NMR data matched those reported in the literature.[11]

^{1}H NMR (400 MHz, CDCl$_3$): δ 7.41 – 7.37 (m, 2H), 7.34 – 7.30 (m, 2H), 7.13 (s, 4H), 4.85 (dd, *J* = 9.0, 4.3 Hz, 1H), 3.02 (dd, *J* = 13.8, 4.3 Hz, 1H), 2.93 (dd, *J* = 13.8, 9.0 Hz, 1H), 2.34 (s, 3H), 1.90 (s, 1H), 1.33 (s, 9H).

^{13}C NMR (125 MHz, CDCl$_3$): δ 150.5, 141.0, 136.1, 135.2, 129.3, 129.2, 125.6, 125.3, 75.1, 45.5, 34.5, 31.4, 30.0, 21.0.

1-(4-fluorophenyl)-2-(*p*-tolyl)ethan-1-ol (7ca)

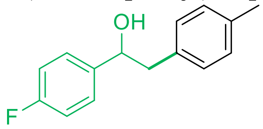

Following the general procedure **A** compound **7ca** was obtained from 4-fluorostyrene oxide (**5c**) (28 mg, 0.20 mmol) and 4-iodotoluene (**6a**) (65 mg, 0.30 mmol). The crude product was purified by column chromatography (5:95 AcOEt/Hexane) to afford 27 mg of 1-(4-fluorophenyl)-2-(*p*-tolyl)ethan-1-ol (**7ca**) as white solid, (yield = **59%**).

NMR data matched those reported in the literature.[11]

^{1}H NMR (400 MHz, CDCl$_3$): δ 7.33 – 7.30 (m, 2H), 7.13 – 6.98 (m, 6H), 4.87 (dd, *J* = 7.8, 5.4 Hz, 1H), 3.01 (dd, *J* = 12.0, 4.0 Hz, 1H), 2.94 (dd, *J* = 13.7, 8.1 Hz, 1H), 2.33 (s, 3H), 1.91 (s, 1H).

^{13}C NMR (150 MHz, CDCl$_3$): δ 162.2 (d, *J* = 247.0 Hz), 139.5 (d, *J* = 3.4 Hz), 136.3, 134.5, 129.4, 129.3, 127.5 (d, *J* = 8.8 Hz), 115.5 (d, *J* = 21.4 Hz), 74.7, 45.8, 21.1.

^{19}F NMR (376 MHz, CDCl$_3$): δ -115.1.

4-(1-hydroxy-2-(*p*-tolyl)ethyl)benzonitrile (7da)

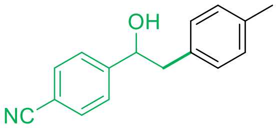

Following the general procedure **A** compound **7da** was obtained from 4-oxiran-2-ylbenzonitrile (**5d**) (29 mg, 0.20 mmol) and 4-iodotoluene (**6a**) (65 mg, 0.30 mmol). The crude product was purified by column chromatography (10:90 AcOEt/Hexane) to afford 25 mg of 4-(1-hydroxy-2-(*p*-tolyl)ethyl)benzonitrile (**7da**) as white solid, (yield = **53%**).

m.p. 79.5-80 °C

^{1}H NMR (400 MHz, CDCl$_3$): δ 7.63 (d, *J* = 8.1 Hz, 2H), 7.45 (d, *J* = 8.0 Hz, 2H), 7.13 (d, *J* = 7.8 Hz, 2H), 7.05 (d, *J* = 7.9 Hz, 2H), 4.96 – 4.92 (m, 1H), 3.01 (dd, *J* = 13.5, 4.8 Hz, 1H), 2.89 (dd, *J* = 13.6, 8.5 Hz, 1H), 2.34 (s, 3H), 2.03 (d, *J* = 3.6 Hz, 1H).

^{13}C NMR (125 MHz, CDCl$_3$): δ 149.0, 136.7, 133.6, 132.2, 129.4, 129.3, 126.6, 118.9, 111.2, 74.5, 45.7, 21.0.

HRMS (EI) $[M]^+$ calculated for $C_{16}H_{15}NO$: 237.1154, found: 237.1154.

GC Chromatogram: (99% purity)

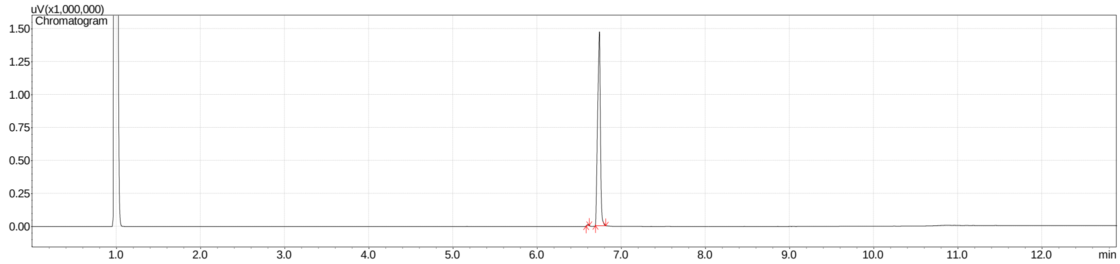

2-phenyl-1-(*p*-tolyl)propan-2-ol (7fa)

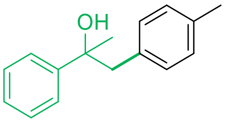

Following the general procedure **A** compound **7fa** was obtained from 2-methyl-2-phenyloxirane (**5f**) (27 mg, 0.20 mmol) and 4-iodotoluene (**6a**) (65 mg, 0.30 mmol). The crude product was purified by column chromatography (5:95 AcOEt/Hexane) to afford 20 mg of 2-phenyl-1-(*p*-tolyl)propan-2-ol (**7fa**) as white solid, (yield = **44%**).

NMR data matched those reported in the literature.[12]

^{1}H NMR (400 MHz, CDCl$_3$): δ 7.42 – 7.39 (m, 2H), 7.35 – 7.31 (m, 2H), 7.26 – 7.22 (m, 1H), 7.03 (d, *J* = 7.8 Hz, 2H), 6.88 (d, *J* = 8.0 Hz, 2H), 3.11 (d, *J* = 13.4 Hz, 1H), 2.99 (d, *J* = 13.4 Hz, 1H), 2.30 (s, 3H), 1.85 (s, 1H), 1.56 (s, 3H).

^{13}C NMR (125 MHz, CDCl$_3$): δ 147.0, 136.4, 133.7, 130.6, 129.0, 128.2, 126.7, 125.1, 74.5, 50.2, 29.6, 21.2.

1-phenyl-2-(*m*-tolyl)ethan-1-ol (7ab)

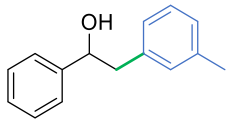

Following the general procedure **A** compound **7ab** was obtained from styrene oxide (**5a**) (24 mg, 0.20 mmol) and 3-iodotoluene (**6b**) (65 mg, 0.30 mmol). The crude product was purified by column chromatography (5:95 AcOEt/Hexane) to afford 20 mg of 1-phenyl-2-(*m*-tolyl)ethan-1-ol (**7ab**) as white solid, (yield = **47%**).

NMR data matched those reported in the literature.[11]

^{1}H NMR (400 MHz, CDCl$_3$): δ 7.40 – 7.26 (m, 5H), 7.22 – 7.18 (m, 1H), 7.08 – 6.99 (m, 3H), 4.90 (dd, *J* = 8.8, 4.5 Hz, 1H), 3.02 (dd, *J* = 13.7, 4.5 Hz, 1H), 2.93 (dd, *J* = 13.7, 8.9 Hz, 1H), 2.34 (s, 3H), 1.96 (s, 1H).

^{13}C NMR (125 MHz, CDCl$_3$): δ 143.9, 138.2, 138.0, 130.3, 128.44, 128.40, 127.6, 127.4, 126.5, 125.9, 75.3, 46.1, 21.4.

1-phenyl-2-(*o*-tolyl)ethan-1-ol (7ac)

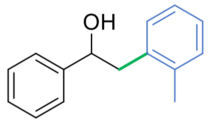

Following the general procedure **A** compound **7ac** was obtained from styrene oxide (**5a**) (24 mg, 0.20 mmol) and 2-iodotoluene (**6c**) (65 mg, 0.30 mmol). The crude product was purified by column chromatography (5:95 AcOEt/Hexane) to afford 14 mg of 1-phenyl-2-(*o*-tolyl)ethan-1-ol (**7ac**) as white solid, (yield = **33%**).

NMR data matched those reported in the literature.[11]

^{1}H NMR (400 MHz, CDCl$_3$): δ 7.39 – 7.27 (m, 5H), 7.18 – 7.14 (m, 4H), 4.91 (dd, *J* = 8.1, 5.2 Hz, 1H), 3.06 (dd, *J* = 12.0, 4.0 Hz, 1H), 3.01 (dd, *J* = 12.0, 8.0 Hz, 1H), 2.31 (s, 3H), 1.94 (s, 1H).

^{13}C NMR (125 MHz, CDCl$_3$): δ 144.1, 136.8, 136.3, 130.5, 130.3, 128.4, 127.6, 126.8, 126.0, 125.8, 74.4, 43.4, 19.6.

2-(4-chlorophenyl)-1-phenylethan-1-ol (7ad)

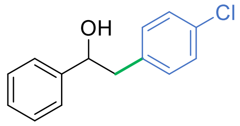

Following the general procedure **A** compound **7ad** was obtained from styrene oxide (**5a**) (24 mg, 0.20 mmol) and 1-chloro-4-iodobenzene (**6d**) (72 mg, 0.30 mmol). The crude product was purified by column chromatography (5:95 AcOEt/Hexane) to afford 21 mg of 2-(4-chlorophenyl)-1-phenylethan-1-ol (**7ad**) as white solid. (Yield = **44%**).

NMR data matched those reported in the literature.[13]

^{1}H NMR (400 MHz, CDCl$_3$): δ 7.37 – 7.28 (m, 5H), 7.27 – 7.22 (m, 2H), 7.11 – 7.07 (m, 2H), 4.87 (t, *J* = 6.6 Hz, 1H), 2.99 (d, *J* = 6.6 Hz, 2H), 1.92 (s, 1H).

^{13}C NMR (125 MHz, CDCl$_3$): δ 143.6, 136.5, 132.4, 130.9, 128.5, 128.5, 127.8, 125.9, 75.3, 45.2.

2-(benzo[*d*][1,3]dioxol-5-yl)-1-phenylethan-1-ol (7ae)

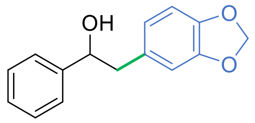

Following the general procedure **A** compound **7ae** was obtained from styrene oxide (**5a**) (24 mg, 0.20 mmol) and 5-iodo-1,3-benzodioxole (**6e**) (74 mg, 0.30 mmol). The crude product was purified by column chromatography (5:95 AcOEt/Hexane) to afford 30 mg of 2-(benzo[*d*][1,3]dioxol-5-yl)-1-phenylethan-1-ol (**7ae**) as white solid, (yield = **61%**).

m.p. 96.5-97.5 °C

^{1}H NMR (400 MHz, CDCl$_3$): δ 7.37 – 7.26 (m, 5H), 6.77 – 6.61 (m, 3H), 5.93 (s, 2H), 4.84 (dd, *J* = 8.3, 4.9 Hz, 1H), 2.96 (dd, *J* = 13.8, 4.9 Hz, 1H), 2.90 (dd, *J* = 13.8, 8.4 Hz, 1H), 2.01 (s, 1H).

^{13}C NMR (125 MHz, CDCl$_3$): δ 147.7, 146.3, 143.8, 131.7, 128.4, 127.6, 125.9, 122.5, 109.8, 108.3, 100.9, 75.4, 45.8.

HRMS (EI) [M]$^+$ calculated for $C_{15}H_{14}O_3$: 242.0943, found: 242.0938.

Elemental Analysis (%) calculated for $C_{15}H_{14}O_3$:C 74.36, H 5.82, found: C 74.33, H 5.86.

1-phenyl-2-(4-(trifluoromethyl)phenyl)ethan-1-ol (7af)

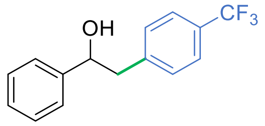

Following the general procedure **A** compound **7af** was obtained from styrene oxide (**5a**) (24 mg, 0.20 mmol) and 4-iodobenzotrifluoride (**6f**) (82 mg, 0.30 mmol). The crude product was purified by column chromatography (5:95 AcOEt/Hexane) to afford 19 mg of 1-phenyl-2-(4-(trifluoromethyl)phenyl)ethan-1-ol (**7af**) as white solid, (yield = **36%**).

NMR data matched those reported in the literature.[11]

^{1}H NMR (400 MHz, CDCl$_3$): δ 7.54 (d, *J* = 8.0 Hz, 2H), 7.38 – 7.27 (m, 7H), 4.92 (t, *J* = 5.0 Hz, 1H), 3.12 – 3.04 (m, 2H), 1.91 (s, 1H).

^{13}C NMR (125 MHz, CDCl$_3$): δ 143.6, 142.4, 130.0, 129.0 (q, *J* = 32.8 Hz), 128.7, 128.1, 126.0, 124.43 (q, J = 273.4 Hz), 125.41 (q, J = 2.8 Hz), 75.31, 45.73.

^{19}F NMR (376 MHz, CDCl$_3$): δ -62.4.

1-(4-(2-hydroxy-2-phenylethyl)phenyl)ethan-1-one (7ag)

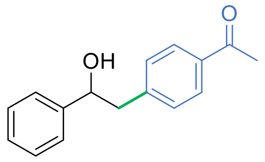

Following the general procedure **A** compound **7ag** was obtained from styrene oxide (**5a**) (24 mg, 0.20 mmol) and 4'-iodoacetophenone (**6g**) (74 mg, 0.30 mmol). The crude product was purified by column chromatography (10:90 AcOEt/Hexane) to afford 29 mg of 1-(4-(2-hydroxy-2-phenylethyl)phenyl)ethan-1-one (**7ag**) as white solid, (yield = **60%**).

NMR data matched those reported in the literature.[14]

^{1}H NMR (400 MHz, CDCl$_3$): δ 7.89 – 7.85 (m, 2H), 7.36 – 7.25 (m, 7H), 4.93 (dd, *J* = 7.4, 5.8 Hz, 1H), 3.11 (dd, *J* = 12.0, 8.0 Hz, 1H), 3.07 (dd, *J* = 16.0, 8.0 Hz, 1H), 2.57 (s, 3H), 1.99 (s, 1H).

^{13}C NMR (125 MHz, CDCl$_3$): δ 197.8, 143.8, 143.5, 135.6, 129.8, 128.5, 128.5, 127.9, 125.9, 75.1, 45.8, 26.5.

2-(4-methoxyphenyl)-1-phenylethan-1-ol (7ah)

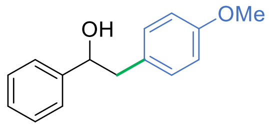

Following the general procedure **A** compound **7ah** was obtained from styrene oxide (**5a**) (24 mg, 0.20 mmol) and 4-iodoanisole (**6h**) (70 mg, 0.30 mmol). The crude product was purified by column chromatography (5:95 AcOEt/Hexane) to afford 22 mg of 2-(4-methoxyphenyl)-1-phenylethan-1-ol (**7ah**) as white solid, (yield = **48%**).

NMR data matched those reported in the literature.[14]

^{1}H NMR (400 MHz, CDCl$_3$): δ 7.35 (d, *J* = 4.4 Hz, 4H), 7.30 – 7.26 (m, 1H), 7.13 – 7.08 (m, 2H), 6.86 – 6.81 (m, 2H), 4.85 (dd, *J* = 8.3, 5.0 Hz, 1H), 3.79 (s, 3H), 3.00 (dd, *J* = 13.8, 4.9 Hz, 1H), 2.93 (dd, *J* = 13.8, 8.3 Hz, 1H), 1.99 (s, 1H).

^{13}C NMR (100 MHz, CDCl$_3$): δ 158.6, 144.0, 130.6, 130.1, 128.5, 127.7, 126.0, 114.1, 75.6, 55.4, 45.3.

4-(2-hydroxy-2-phenylethyl)benzonitrile (7ai)

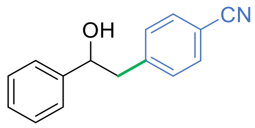

Following the general procedure **A** compound **7ai** was obtained from styrene oxide (**5a**) (24 mg, 0.20 mmol) and 4-iodobenzonitrile (**6i**) (69 mg, 0.30 mmol). The crude product was purified by column chromatography (5:95 AcOEt/Hexane) to afford 12 mg of 4-(2-hydroxy-2-phenylethyl)benzonitrile (**7ai**) as white solid, (yield = **28%**).

NMR data matched those reported in the literature.[14]

^{1}H NMR (400 MHz, CDCl$_3$): δ 7.57 – 7.52 (m, 2H), 7.37 – 7.24 (m, 7H), 4.91 (dd, *J* = 7.7, 5.4 Hz, 1H), 3.11 (dd, *J* = 13.6, 7.7 Hz, 1H), 3.05 (dd, *J* = 13.7, 5.4 Hz, 1H), 2.01 (s, 1H).

^{13}C NMR (125 MHz, CDCl$_3$): δ 143.8, 143.3, 132.0, 130.4, 128.6, 128.0, 125.8, 118.9, 110.4, 75.0, 45.7.

1,2-diphenylethan-1-ol (7aj)

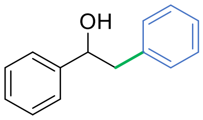

Following the general procedure **A** compound **7aj** was obtained from styrene oxide (**5a**) (24 mg, 0.20 mmol) and iodobenzene (**6j**) (61 mg, 0.30 mmol). The crude product was purified by column chromatography (5:95 AcOEt/Hexane) to afford 25 mg of 1,2-diphenylethan-1-ol (**7aj**) as white solid, (yield = **63%**).

NMR data matched those reported in the literature.[15]

^{1}H NMR (400 MHz, $CDCl_3$): δ 7.37 – 7.26 (m, 7H), 7.25 – 7.18 (m, 3H), 4.91 (dd, *J* = 8.4, 5.0 Hz, 1H), 3.05 (dd, *J* = 13.7, 4.9 Hz, 1H), 2.99 (dd, *J* = 13.7, 8.4 Hz, 1H), 1.93 (s, 1H).

^{13}C NMR (125 MHz, $CDCl_3$): δ 143.8, 138.0, 129.5, 128.5, 128.4, 127.6, 126.6, 125.9, 75.3, 46.1.

2-(18aphthalene-1-yl)-1-phenylethan-1-ol (7ak)

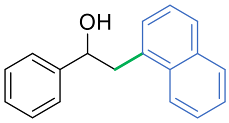

Following the general procedure **A** compound **7ak** was obtained from styrene oxide (**5a**) (24 mg, 0.20 mmol) and 1-iodonaphthalene (**6k**) (76 mg, 0.30 mmol). The crude product was purified by column chromatography (5:95 AcOEt/Hexane) to afford 21 mg of 2-(18aphthalene-1-yl)-1-phenylethan-1-ol (**7ak**) as white solid, (yield = **42%**).

m.p. 66.0-66.5 °C

^{1}H NMR (400 MHz, $CDCl_3$): δ 8.13 (dd, *J* = 12.0, 4.0 Hz, 1H), 7.89 (dd, *J* = 8.3, 1.1 Hz, 1H), 7.78 (d, *J* = 8.2 Hz, 1H), 7.58 – 7.48 (m, 2H), 7.46 – 7.28 (m, 7H), 5.08 (dd, *J* = 8.9, 4.3 Hz, 1H), 3.56 (dd, *J* = 14.0, 4.3 Hz, 1H), 3.41 (dd, *J* = 14.0, 8.9 Hz, 1H), 1.95 (s, 1H).

^{13}C NMR (125 MHz, $CDCl_3$): δ 144.1, 134.1, 134.0, 132.1, 128.9, 128.5, 127.9, 127.7, 127.6, 126.1, 125.8, 125.7, 125.5, 123.7, 74.4, 43.3.

HRMS (ESI) $[M+Na]^+$ calculated for $C_{18}H_{16}ONa$: 271.1099, found: 271.1096.

Elemental Analysis (%) calculated for $C_{18}H_{16}O$: C 87.06, H 6.49, found: C 86.92, H 6.59.

1-phenyl-2-(1-tosyl-1*H*-indol-5-yl)ethan-1-ol (7al)

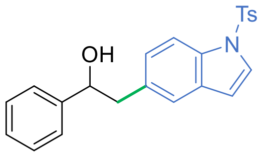

Following the general procedure **A** compound **7al** was obtained from styrene oxide (**5a**) (24 mg, 0.20 mmol) and 5-iodo-1-(4-methylphenylsulfonyl)indole (**6l**) (119 mg, 0.30 mmol). The crude product was purified by column chromatography (10:90 AcOEt/Hexane) to afford 34 mg of 1-phenyl-2-(1-tosyl-1*H*-indol-5-yl)ethan-1-ol (**7al**) as white solid, (yield = **44%**).

m.p. 47.6-47.9 °C

^{1}H NMR (400 MHz, CDCl$_3$): δ 7.91 (d, *J* = 8.5 Hz, 1H), 7.78 – 7.73 (m, 2H), 7.54 (d, *J* = 3.7 Hz, 1H), 7.37 – 7.27 (m, 6H), 7.21 (d, *J* = 8.2 Hz, 2H), 7.14 (dd, *J* = 8.5, 1.7 Hz, 1H), 6.60 (dd, *J* = 3.7, 0.8 Hz, 1H), 4.89 (dd, *J* = 8.7, 4.5 Hz, 1H), 3.10 (dd, *J* = 13.8, 4.6 Hz, 1H), 3.02 (dd, *J* = 13.8, 8.7 Hz, 1H), 2.34 (s, 3H), 1.96 (s, 1H).

^{13}C NMR (125 MHz, CDCl$_3$): δ 144.9, 143.8, 135.3, 133.8, 133.1, 131.1, 129.8, 128.4, 127.6, 126.8, 126.62, 126.1, 125.8, 122.0, 113.5, 108.9, 75.4, 45.9, 21.5.

HRMS (ESI) $[M+Na]^+$ calculated for $C_{23}H_{21}NO_3SNa$: 414.1140, found: 414.1134.

Elemental Analysis (%) calculated for $C_{23}H_{21}NO_3S$: C 70.57, H 5.41, N 3.58, S 8.19, found: C 70.31, H 5.44, N 3.52, S 7.99.

4-(2-hydroxy-2-phenylethyl)benzamide (7am)

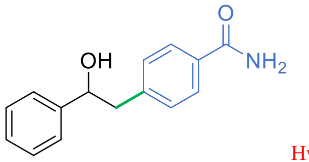

Hygroscopic compound

Following the general procedure **A** compound **7am** was obtained from styrene oxide (**5a**) (24 mg, 0.20 mmol) and 4-iodobenzamide (**6m**) (74 mg, 0.30 mmol). The crude product was purified by column chromatography (gradually from AcOEt/Hexane 50:50 to 80:20) to afford 16 mg of 4-(2-hydroxy-2-phenylethyl)benzamide (**7am**) as white solid, (yield = **33%**).

m.p. 175.7-176.2 °C

^{1}H NMR (400 MHz, MeOD): δ 7.74 – 7.70 (m, 2H), 7.27 (d, *J* = 4.3 Hz, 4H), 7.24 – 7.18 (m, 3H), 4.86 (dd, *J* = 7.3, 6.3 Hz, 1H), 3.10 (dd, *J* = 13.4, 7.4 Hz, 1H), 3.01 (dd, *J* = 13.4, 6.2 Hz, 1H).

^{13}C NMR (125 MHz, MeOD): δ 172.4, 145.5, 144.3, 132.7, 130.8, 129.2, 128.4, 128.4, 127.2, 76.1, 46.6.

HRMS (ESI) $[M+Na]^+$ calculated for $C_{15}H_{15}NO_2Na$: 264.1000, found: 264.0994.

Elemental Analysis (%) calculated for $C_{15}H_{15}NO_2$: C 74.67, H 6.27, N 5.81, found: C 74.31, H 6.55, N 5.56.

tert-butyl (4-(2-hydroxy-2-phenylethyl)phenyl)carbamate (7an)

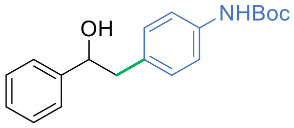

Following the general procedure **A** compound **7an** was obtained from styrene oxide (**5a**) (24 mg, 0.20 mmol) and *N*-Boc-4-iodoaniline (**6n**) (96 mg, 0.30 mmol). The crude product was purified by column chromatography (10:90 AcOEt/Hexane) to afford 38 mg of *tert*-butyl (4-(2-hydroxy-2-phenylethyl)phenyl)carbamate (**7an**) as yellow pale solid, (yield = **60%**).

m.p. 119.2-119.6 °C

^{1}H NMR (400 MHz, CDCl$_3$): δ 7.27 (d, J = 4.3 Hz, 4H), 7.24 – 7.18 (m, 3H), 7.02 (d, J = 8.5 Hz, 2H), 6.40 (s, 1H), 4.78 (dd, J = 8.3, 5.0 Hz, 1H), 2.92 (dd, J = 13.7, 5.0 Hz, 1H), 2.87 (dd, J = 13.7, 8.3 Hz, 1H), 1.93 (s, 1H), 1.45 (s, 9H).

^{13}C NMR (125 MHz, CDCl$_3$): δ 152.8, 143.8, 136.9, 132.5, 130.0, 128.4, 127.5, 125.9, 118.7, 80.5, 75.30, 45.4, 28.3.

HRMS (ESI) [M+Na]$^+$ calculated for $C_{19}H_{23}NO_3Na$: 336.1576, found: 336.1571.

Elemental Analysis (%) calculated for $C_{19}H_{23}NO_3$: C 72.82, H 7.40, N 4.47, found: C 72.75, H 7.45, N 4.42.

2-(4-(hydroxymethyl)phenyl)-1-phenylethan-1-ol (7ao)

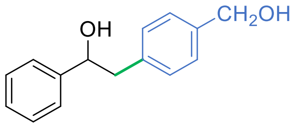

Hygroscopic compound

Following the general procedure **A** compound **7ao** was obtained from styrene oxide (**5a**) (24 mg, 0.20 mmol) and 4-iodobenzyl alcohol (**6o**) (70 mg, 0.30 mmol). The crude product was purified by column chromatography (25:75 AcOEt/Hexane) to afford 21 mg of 2-(4-(hydroxymethyl)phenyl)-1-phenylethan-1-ol (**7ao**) as white solid, (yield = **46%**).

m.p. 71.5-72.0 °C

^{1}H NMR (400 MHz, CDCl$_3$): δ 7.35 (d, J = 4.1 Hz, 4H), 7.31 – 7.26 (m, 3H), 7.19 (d, J = 7.9 Hz, 2H), 4.89 (dd, J = 8.3, 5.1 Hz, 1H), 4.65 (s, 2H), 3.05 (dd, J = 12.0, 4.0 Hz, 1H), 2.98 (dd, J = 16.0, 8.0 Hz, 1H), 1.99 (s, 1H), 1.69 (s, 1H).

^{13}C NMR (125 MHz, CDCl$_3$): δ 143.7, 139.2, 137.5, 129.7, 128.5, 127.7, 127.3, 125.9, 75.4, 65.2, 45.7.

HRMS (ESI) [M+Na]$^+$ calculated for $C_{15}H_{16}O_2Na$: 251.1048, found: 251.1047.

Elemental Analysis (%) calculated for $C_{15}H_{16}O_2 + 0.2{\cdot}H_2O$: C 77.69, H 7.13, found C 77.66, H 7.33.

1-(4-tolyl)hexan-2-ol (7ha)

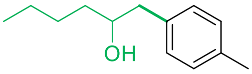

Following the general procedure **B** compound 7**ha** was obtained from 1,2-epoxyhexane (**5h**) (20 mg, 0.20 mmol) and 4-iodotoluene (**6a**) (65 mg, 0.30 mmol). The crude product was purified by column chromatography (5:95 AcOEt/Hexane) to afford 28 mg of 1-(4-tolyl)hexan-2-ol (**7ha**) as white solid, (yield = **74%**).

m.p. 37.2-36.5 °C

^{1}H NMR (400 MHz, $CDCl_3$): δ 7.14 – 7.09 (m, 4H), 3.82 – 3.76 (m, 1H), 2.80 (dd, *J* = 13.6, 4.2 Hz, 1H), 2.60 (dd, *J* = 13.6, 8.4 Hz, 1H), 2.33 (s, 3H), 1.55 – 1.45 (m, 4H), 1.40 – 1.31 (m, 3H), 0.92 (t, *J* = 7.2 Hz, 3H).

^{13}C NMR (125 MHz, $CDCl_3$): δ 136.1, 135.6, 129.44, 129.38, 72.7, 43.7, 36.7, 28.1, 22.9, 21.1, 14.2.

HRMS (EI) $[M]^+$ calculated for $C_{13}H_{20}O$: 192.1514, found: 192.1515.

Elemenal Analysis (%) calculated for $C_{13}H_{20}O$: C 81.20, H 10.48, found: C 80.83, H 10.52.

1-(4-tolyl)dodecan-2-ol (7ia)

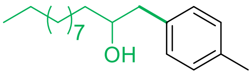

Following the general procedure **B** compound **7ia** was obtained from 1,2-epoxydodecane (**5i**) (37 mg, 0.20 mmol) and 4-iodotoluene (**6a**) (65 mg, 0.30 mmol). The crude product was purified by column chromatography (2:98 AcOEt/Hexane) to afford 43 mg of 1-(*p*-tolyl)dodecan-2-ol (**7ia**) as white solid, (yield = **77%**).

m.p. 53.9-54.8 °C

^{1}H NMR (400 MHz, $CDCl_3$): δ 7.14 – 7.07 (m, 4H), 3.82 – 3.76 (m, 1H), 2.79 (dd, *J* = 13.6, 4.2 Hz, 1H), 2.60 (dd, *J* = 13.6, 8.4 Hz, 1H), 2.33 (s, 3H), 1.55 – 1.42 (m, 5H), 1.26 (m, 14H), 0.88 (t, *J* = 8.0 Hz, 3H).

^{13}C NMR (125 MHz, $CDCl_3$): δ 135.9, 135.5, 129.29, 129.25, 72.7, 43.6, 36.8, 31.9, 29.7, 29.62, 29.61, 29.3, 25.8, 22.7, 21.0, 14.1.

HRMS (EI) $[M]^+$ calculated for $C_{19}H_{32}O$: 276.2453, found: 276.2454.

Elemental Analysis (%) calculated for $C_{19}H_{32}O$: C 82.55, H 11.67, found: C 82.67, H 11.48.

1-(benzyloxy)-3-(4-tolyl)propan-2-ol (7ja)

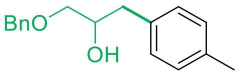

Following the general procedure **B** compound **7ja** was obtained from benzyl glycidyl ether (**5j**) (33 mg, 0.20 mmol) and 4-iodotoluene (**6a**) (65 mg, 0.30 mmol). The crude product was purified by column chromatography (10:90 AcOEt/Hexane) to afford 31 mg of 1-(benzyloxy)-3-(*p*-tolyl)propan-2-ol (**7ja**) as yellow pale oil, (yield = **61%**).

^{1}H NMR (400 MHz, $CDCl_3$): δ 7.40 – 7.28 (m, 5H), 7.11 (s, 4H), 4.55 (s, 2H), 4.04 (dd, *J* = 6.8, 3.5 Hz, 1H), 3.52 (dd, *J* = 9.5, 3.5 Hz, 1H), 3.41 (dd, *J* = 9.5, 6.9 Hz, 1H), 2.78 (d, *J* = 6.7 Hz, 2H), 2.33 (s, 3H), 1.62 (s, 1H).

^{13}C NMR (125 MHz, $CDCl_3$): δ 138.0, 135.9, 134.8, 129.19, 129.16, 128.4, 127.7, 73.6, 73.4, 71.5, 39.4, 21.0.

HRMS (EI) $[M]^+$ calculated for $C_{17}H_{20}O_2$: 256.1463, found: 256.14.74.

GC Chromatogram: (98% purity)

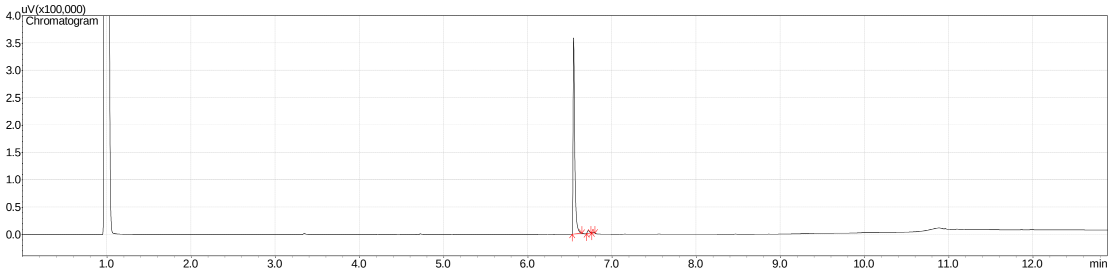

4-(phenylsulfonyl)-1-(*p*-tolyl)butan-2-ol (7ka)

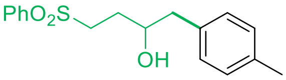

Following the general procedure **B** compound **7ka** was obtained from 4-(phenylsulfonyl)-1,2-epoxubutane (**5k**) (42 mg, 0.20 mmol) and 4-iodotoluene (**6a**) (65 mg, 0.30 mmol). The crude product was purified by column chromatography (25:75 AcOEt/Hexane) to afford 45 mg of 4-(phenylsulfonyl)-1-(4-tolyl)butan-2-ol (**7ka**) as white solid, (yield = **73%**).

m.p. 94.8-95.2 °C

^{1}H NMR (400 MHz, CDCl$_3$): δ 7.90 (d, *J* = 7.3 Hz, 2H), 7.67 – 7.64 (m, 1H), 7.58 – 7.54 (m, 2H), 7.11 (d, *J* = 7.8 Hz, 2H), 7.04 (d, *J* = 7.9 Hz, 2H), 3.92 – 3.84 (m, 1H), 3.34 (ddd, *J* = 15.2, 10.1, 5.3 Hz, 1H), 3.21 (ddd, J = 16.0, 12.0, 8.0 Hz, 1H), 2.76 (dd, *J* = 13.6, 4.4 Hz, 1H), 2.62 (dd, *J* = 13.6, 8.3 Hz, 1H), 2.32 (s, 3H), 2.08 – 1.99 (m, 1H), 1.87 – 1.77 (m, 1H), 1.67 (d, *J* = 4.0 Hz, 1H).

^{13}C NMR (125 MHz, CDCl$_3$): δ 139.2, 136.5, 134.1, 133.7, 129.5, 129.3, 129.2, 128.0, 70.8, 53.2, 43.6, 29.4, 21.0.

HRMS (ESI) $[M+Na]^+$ calculated for $C_{17}H_{20}O_3SNa$: 327.1031, found: 327.1015.

GC Chromatogram: (>99% purity)

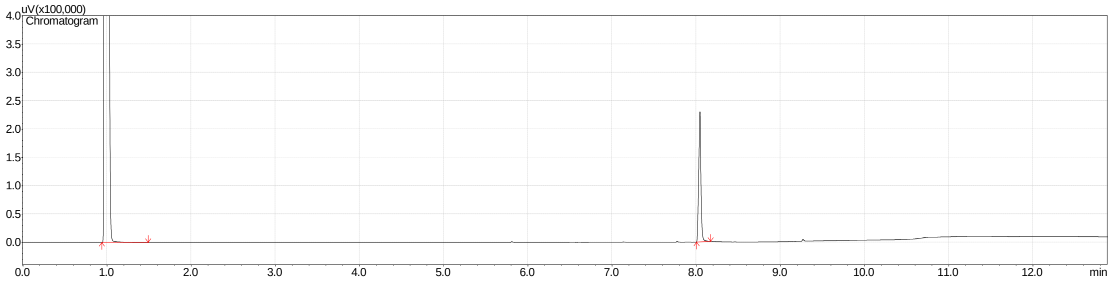

3,3-dimethyl-1-(4-tolyl)butan-2-ol (7la)

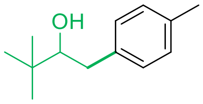

Unstable compound

Following the general procedure **B** compound **7la** was obtained from 3,3-dimethyl-1,2-epoxybutane (**5l**) (20 mg, 0.20 mmol) and 4-iodotoluene (**6a**) (65 mg, 0.30 mmol). The crude product was purified by column chromatography (5:95 AcOEt/Hexane) to afford 19 mg of 3,3-dimethyl-1-(4-tolyl)butan-2-ol (**7la**) as white solid, (yield = **37%**).

m.p. 65.3-66.0 °C

^{1}H NMR (400 MHz, CDCl$_3$): δ 7.13 (s, 4H), 3.41 (dd, J = 10.7, 2.1 Hz, 1H), 2.88 (dd, J = 13.6, 2.0 Hz, 1H), 2.43 (dd, J = 13.6, 10.7 Hz, 1H), 2.33 (s, 3H), 1.57 (s, 1H), 1.00 (s, 9H).

^{13}C NMR (125 MHz, CDCl$_3$): δ 136.7, 135.8, 129.3, 129.2, 80.6, 37.9, 34.8, 25.9, 21.0.

HRMS (EI) $[M]^+$ calculated for $C_{13}H_{20}O$: 192.1514, found: 192.1521.

GC Chromatogram: (97% purity)

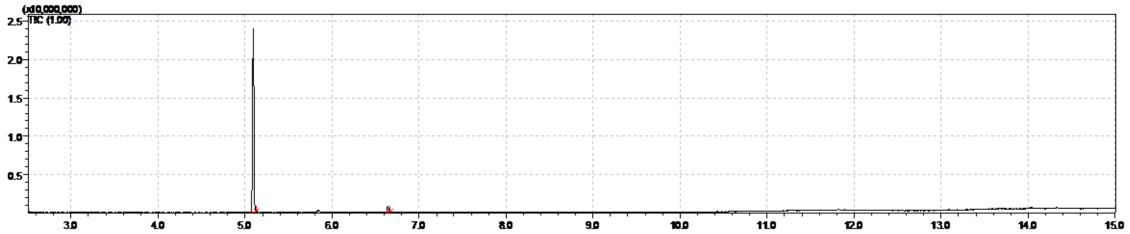

1-(4-methylbenzyl)cyclohexan-1-ol (7ma)

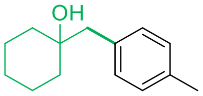

Following the general procedure **C** compound **7ma** was obtained from 1-oxaspiro(2.5)octane (**5m**) (22 mg, 0.20 mmol) and 4-iodotoluene (**6a**) (65 mg, 0.30 mmol). The crude product was purified by column chromatography (5:95 AcOEt/Hexane) to afford 9 mg of 1-(4-methylbenzyl)cyclohexan-1-ol (**7ma**) as white solid, (yield = **21%**).

Following the general procedure **D** compound **7ma** was obtained from 1-oxaspiro(2.5)octane (**5m**) (22 mg, 0.20 mmol) and 4-iodotoluene (**6a**) (65 mg, 0.30 mmol). The crude product was purified by column chromatography (5:95 AcOEt/Hexane) to afford 12 mg of 1-(4-methylbenzyl)cyclohexan-1-ol (**7ma**) as white solid, (yield = **29%**).

NMR data matched those reported in the literature.[16]

^{1}H NMR (400 MHz, CDCl$_3$): δ 7.10 (m, 4H), 2.71 (s, 2H), 2.33 (s, 3H), 1.61 – 1.38 (m, 11H).

^{13}C NMR (150 MHz, CDCl$_3$): δ 135.9, 134.0, 130.5, 128.9, 71.1, 48.2, 37.3, 25.8, 22.2, 21.0.

(1*S*,2*R*)-2-(4-tolyl)cyclopentan-1-ol (7na)

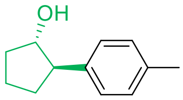

Following the general procedure **C** compound **7na** was obtained from 1,2-epoxycyclopentane (**5n**) (17 mg, 0.20 mmol) and 4-iodotoluene (**6a**) (65 mg, 0.30 mmol). The crude product was purified by column chromatography (10:90 AcOEt/Hexane) to afford 5 mg of (1*S*,2*R*)-2-(*p*-tolyl)cyclopentan-1-ol (**7na**) as yellow pale oil, (yield = **14%**).

Following the general procedure **D** compound **7na** was obtained from 1,2-epoxycyclopentane (**5n**) (17 mg, 0.20 mmol) and 4-iodotoluene (**6a**) (65 mg, 0.30 mmol). The crude product was purified by column chromatography (10:90 AcOEt/Hexane) to afford 20 mg of (1*S*,2*R*)-2-(4-tolyl)cyclopentan-1-ol (**7na**) as yellow pale oil, (yield = **57%**).

^{1}H NMR (400 MHz, CDCl$_3$): δ 7.18 – 7.09 (m, 4H), 4.14 (dd, *J* = 12.0, 8.0 Hz, 1H), (dd, *J* = 16.0, 8.0 Hz 1H), 2.33 (s, 3H), 2.16 – 2.06 (m, 2H), 1.90 – 1.65 (m, 4H), 1.63 (s, 1H).

^{13}C NMR (150 MHz, CDCl$_3$): δ 140.1, 136.0, 129.3, 127.3, 80.5, 54.1, 33.9, 31.8, 21.7, 21.0.

HRMS (EI) [M]$^+$ calculated for $C_{12}H_{16}O$: 176.1201, found: 176.1202.

GC Chromatogram: (>99% purity)

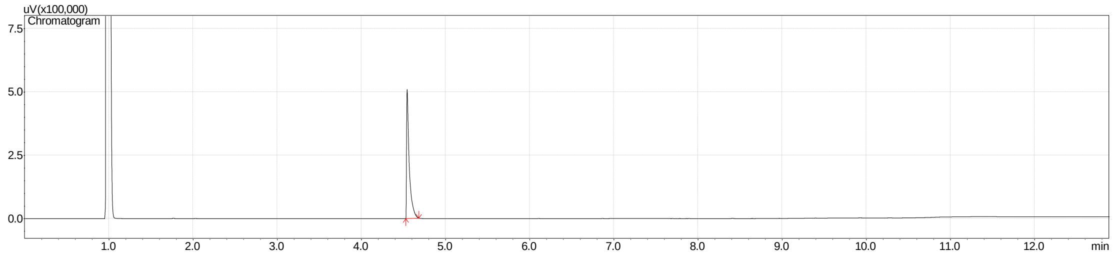

(1*S*,2*R*)-2-(4-tolyl)cyclohexan-1-ol (7oa)

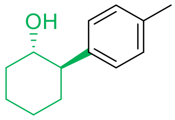

Following the general procedure **C** compound **7oa** was obtained from 1,2-epoxycyclohexane (**5o**) (20 mg, 0.20 mmol) and 4-iodotoluene (**6a**) (65 mg, 0.30 mmol). The crude product was purified by column chromatography (5:95 AcOEt/Hexane) to afford 12 mg of (1*S*,2*R*)-2-(*p*-tolyl)cyclohexan-1-ol (**7oa**) as white solid, (yield = **31%**).

Following the general procedure **D** compound **7oa** was obtained from 1,2-epoxycyclohexane (**5o**) (20 mg, 0.20 mmol) and *p*-iodotoluene (**6a**) (65 mg, 0.30 mmol). The crude product was purified by column chromatography (5:95 AcOEt/Hexane) to afford 21 mg of (1*S*,2*R*)-2-(4-tolyl)cyclohexan-1-ol (**7oa**) as white solid, (yield = **56%**).

NMR data matched those reported in the literature.[17]

^{1}H NMR (400 MHz, CDCl$_3$): δ 7.15 (s, 4H), 3.63 (td, *J* = 10.1, 4.3 Hz, 1H), 2.39 (ddd, *J* = 13.2, 10.0, 3.6 Hz, 1H), 2.33 (s, 3H), 2.14 – 2.08 (m, 1H), 1.89 – 1.72 (m, 3H), 1.59 – 1.31 (m, 5H).

^{13}C NMR (150 MHz, CDCl$_3$): δ 140.2, 136.4, 129.5, 127.8, 74.5, 52.8, 34.4, 33.4, 26.1, 25.1, 21.0.

1-(4-chlorophenyl)hexan-2-ol (7hd)

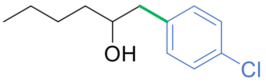

Following the general procedure **B** compound **7hd** was obtained from 1,2-epoxyhexane (**5h**) (20 mg, 0.20 mmol) and 1-chloro-4-iodobenzene (**6d**) (72 mg, 0.30 mmol). The crude product was purified by column chromatography (10:90 AcOEt/Hexane) to afford 23 mg of 1-(4-chlorophenyl)hexan-2-ol (**7hd**) as white solid, (yield = **54%**).

NMR data matched those reported in the literature.[18]

^{1}H NMR (400 MHz, $CDCl_3$): δ 7.29 – 7.26 (m, 2H), 7.16 – 7.14 (m, 2H), 3.82 – 3.76 (m, 1H), 2.79 (dd, *J* = 13.7, 4.3 Hz, 1H), 2.63 (dd, *J* = 13.7, 8.2 Hz, 1H), 1.56 – 1.30 (m, 7H), 0.91 (t, *J* = 7.1 Hz, 3H).

^{13}C NMR (125 MHz, $CDCl_3$): δ 137.34, 132.4, 130.9, 128.8, 72.7, 43.5, 36.7, 28.0, 22.8, 14.2.

1-(benzo[*d*][1,3]dioxol-5-yl)hexan-2-ol (7he)

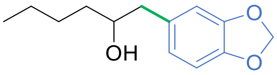

Following the general procedure **B** compound **7he** was obtained from 1,2-epoxyhexane (**5h**) (20 mg, 0.20 mmol) and 5-iodo-1,3-benzodioxole (**6e**) (74 mg, 0.30 mmol). The crude product was purified by column chromatography (10:90 AcOEt/Hexane) to afford 24 mg of 1-(benzo[*d*][1,3]dioxol-5-yl)hexan-2-ol (**7he**) as white solid, (yield = **55%**).

m.p. 65.3-66.0 °C

^{1}H NMR (400 MHz, $CDCl_3$): δ 6.78 – 6.63 (m, 3H), 5.93 (s, 2H), 3.758 – 3.72 (m, 1H), 2.75 (dd, *J* = 13.7, 4.2 Hz, 1H), 2.55 (dd, *J* = 13.7, 8.4 Hz, 1H), 1.53 – 1.30 (m, 7H), 0.91 (t, *J* = 7.1 Hz, 3H).

^{13}C NMR (125 MHz, $CDCl_3$): δ 147.8, 146.2, 132.3, 122.3, 109.7, 108.3, 100.9, 72.7, 43.7, 36.5, 27.9, 22.7, 14.0.

HRMS (EI) $[M]^+$ calculated for $C_{13}H_{18}O_3$: 222.1256, found: 222.1260.

GC Chromatogram: (>99% purity)

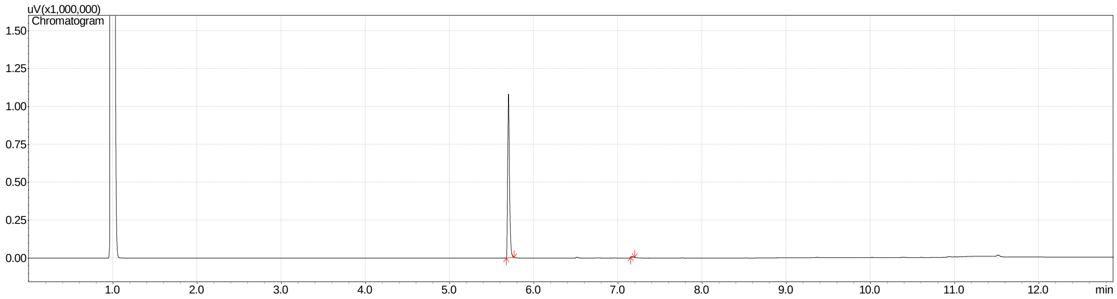

1-(4-(2-hydroxyhexyl)phenyl)ethan-1-one (7hg)

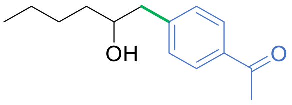

Following the general procedure **B** compound **7hg** was obtained from 1,2-epoxyhexane (**5h**) (20 mg, 0.20 mmol) and 4'-iodoacetophenone (**6g**) (74 mg, 0.30 mmol). The crude product was purified by column chromatography (20:80 AcOEt/Hexane) to afford 22 mg of 1-(4-(2-hydroxyhexyl)phenyl)ethan-1-one (**7hg**) as white solid, (yield = **50%**).

m.p. 65.9-66.4 °C

^{1}H NMR (400 MHz, CDCl$_3$): δ 7.92 – 7.87 (m, 2H), 7.33 – 7.29 (m, 2H), 3.88 – 3.82 (m, 1H), 2.87 (dd, J = 13.6, 4.3 Hz, 1H), 2.73 (dd, J = 13.6, 8.2 Hz, 1H), 2.58 (s, 3H), 1.56 – 1.29 (m, 7H), 0.91 (t, J = 7.2 Hz, 3H).

^{13}C NMR (125 MHz, CDCl$_3$): δ 197.8, 144.6, 135.5, 129.6, 128.6, 72.5, 44.0, 36.7, 27.9, 26.5, 22.6, 14.0.

HRMS (ESI) $[M+Na]^+$ calculated for $C_{14}H_{20}O_2Na$: 243.1361, found: 243.1350.

GC Chromatogram: (>99% purity)

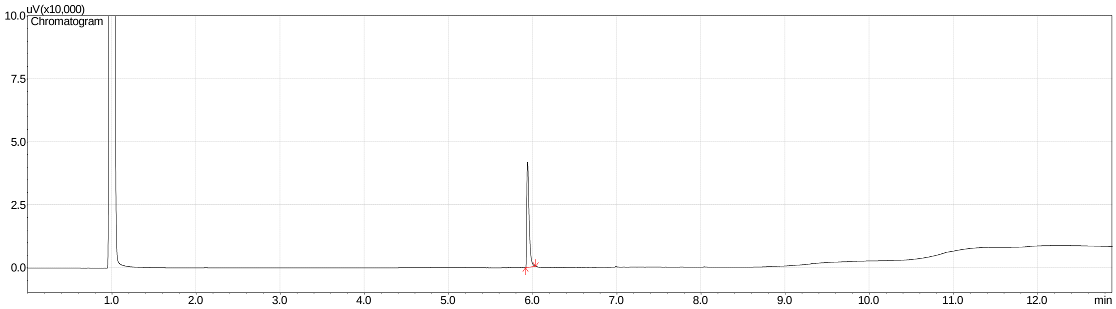

***tert*-butyl (4-(2-hydroxyhexyl)phenyl)carbamate (7hn)**

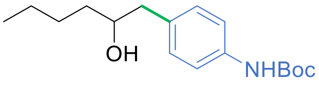

Following the general procedure **B** compound **7hn** was obtained from 1,2-epoxyhexane (**5h**) (20 mg, 0.20 mmol) and *N*-Boc-4-iodoaniline (**6n**) (96 mg, 0.30 mmol). The crude product was purified by column chromatography (10:90 AcOEt/Hexane) to afford 35 mg of *tert*-butyl (4-(2-hydroxyhexyl)phenyl)carbamate (**7hn**) as white solid, (yield = **60%**).

m.p. 96.6-97.1 °C

^{1}H NMR (400 MHz, CDCl$_3$): δ 7.30 – 7.28 (m, 2H), 7.14 – 7.11 (m, 2H), 6.50 (s, 1H), 3.79 – 3.73 (m, 1H), 2.77 (dd, J = 13.7, 4.3 Hz, 1H), 2.58 (dd, J = 13.7, 8.3 Hz, 1H), 1.51 (m, 13H), 1.38 – 1.29 (m, 3H), 0.90 (t, J = 7.2 Hz, 3H).

^{13}C NMR (125 MHz, CDCl$_3$): δ 152.9, 136.8, 133.2, 129.9, 118.9, 80.4, 72.7, 43.3, 36.4, 28.3, 27.9, 22.7, 14.0.

HRMS (ESI) $[M+Na]^+$ calculated for $C_{17}H_{27}NO_3Na$: 316.1889, found: 316.1879.

Elemental Analysis (%) calculated for $C_{17}H_{27}NO_3$: C 69.59, H 9.28, N 4.77, found: C 69.40, H 9.37, N 4.84.

1-(1-tosyl-1*H*-indol-5-yl)hexan-2-ol (7hl)

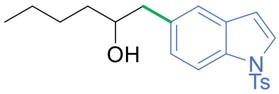

Following the general procedure **B** compound **7hl** was obtained from 1,2-epoxyhexane (**5h**) (20 mg, 0.20 mmol) and 5-iodo-1-(4-methylphenylsulfonyl)indole (**6l**) (119 mg, 0.30 mmol). The crude product was purified by column chromatography (10:90 AcOEt/Hexane) to afford 43 mg of 1-(1-tosyl-1*H*-indol-5-yl)hexan-2-ol (**7hl**) as white solid, (yield = **58%**).

m.p. 65.3-66.0 °C

^{1}H NMR (500 MHz, CDCl$_3$): δ 7.94 (d, *J* = 8.5 Hz, 1H), 7.79 (d, *J* = 8.4 Hz, 2H), 7.57 (d, *J* = 3.7 Hz, 1H), 7.39 (d, *J* = 1.7 Hz, 1H), 7.24 (d, *J* = 8.1 Hz, 2H), 7.18 (dd, *J* = 8.5, 1.7 Hz, 1H), 6.63 (dd, *J* = 3.6, 0.8 Hz, 1H), 3.84 – 3.81 (m, 1H), 2.91 (dd, *J* = 13.7, 4.0 Hz, 1H), 2.70 (dd, *J* = 13.7, 8.6 Hz, 1H), 2.36 (s, 3H), 1.57 – 1.28 (m, 7H), 0.93 (t, *J* = 7.1 Hz, 3H).

^{13}C NMR (125 MHz, CDCl$_3$): δ 145.0, 135.5, 133.83, 133.79, 131.2, 130.0, 127.0, 126.7, 126.2, 122.0, 113.6, 109.0, 73.0, 44.0, 36.7, 28.1, 22.8, 21.7, 14.2.

HRMS (EI) $[M]^+$ calculated for $C_{21}H_{25}NO_3S$: 371.1555, found: 371.1549.

GC Chromatogram: (97% purity)

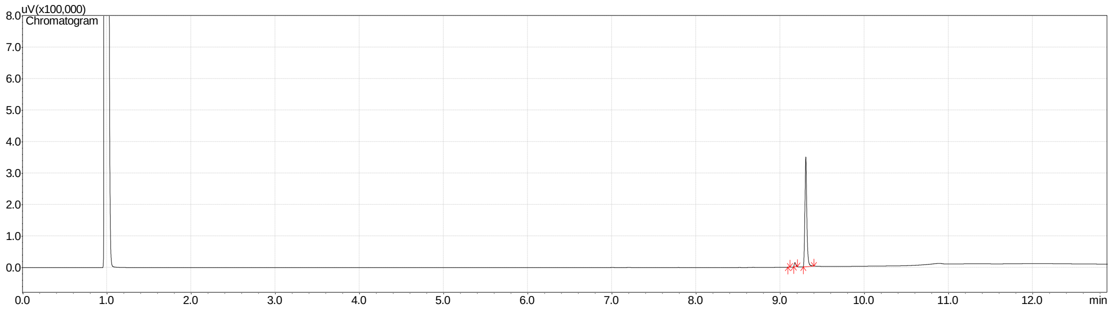

Aziridines – preliminary data

A mixture of 4-methyl-*N*-(2-phenyl-(*p*-tolyl)ethyl)benzenosulphonamide (S23) and 4-methyl-*N*-(1-phenyl-2-(*p*-tolyl)ethyl)benzenosulfonamide (S24)

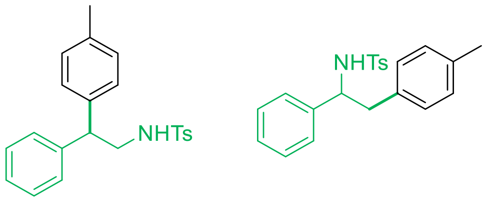

Following the general procedure **A** compounds **S23** and **S24** were obtained from *N*-(*p*-tolylsulfonyl)-2-phenylaziridine (**S20**) (55 mg, 0.20 mmol) and 4-iodotoluene (**6a**) (65 mg, 0.30 mmol). The crude product was purified by column chromatography (10:90 AcOEt/Hexane) to afford an inseparable by column chromatography mixture of 4-methyl-*N*-(2-phenyl-(*p*-tolyl)ethyl)benzenosulphonamide (**S23**) and *N*-(1-phenyl-2-(*p*-tolyl)ethyl)benzenosulfonamide (**S24**) as white solid, (17 mg, branched:linear = 1:2, yield = **23%**).

<u>NMR data matched those reported in the literature.</u>[19,20]

^{1}H NMR (400 MHz, $CDCl_3$): δ 7.68 (d, *J* = 8.0 Hz, $2H_{branched}$), 7.42 (d, *J* = 8.0 Hz, $2H_{linear}$), 7.28 (d, *J* = 10.8 Hz, $2H_{branched}$), 7.27 – 7.24 (m, $2H_{branched}$), 7.21 – 7.15 (m, $3H_{linear}$+$1H_{branched}$), 7.09 – 7.06 (m, $4H_{branched}$+$4H_{linear}$), 6.98 – 6.96 (m, $2H_{linear}$+$2H_{branched}$), 6.79 (d, *J* = 7.7 Hz, $2H_{linear}$), 4.70 (d, *J* = 5.9 Hz, $1H_{linear}$), 4.50 – 4.46 (m, $1H_{linear}$), 4.27 (t, *J* = 6.3 Hz, $1H_{branched}$), 4.07 – 3.98 (m, $1H_{branched}$), 3.54 – 3.51 (m, $2H_{branched}$), 2.93 (dd, *J* = 12.0, 4.0 Hz, $2H_{linear}$), 2.45 (s, $3H_{branched}$), 2.36 (s, $3H_{linear}$), 2.30 (s, $3H_{branched}$), 2.29 (s, $3H_{linear}$).

4-methyl-*N*-(2-(*p*-tolyl)cyclopentyl)benzenosulfonamide (S25)

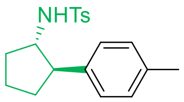

Following the general procedure **D** compound **S25** was obtained from *N*-tosyl-6-azabicyclo[3.1.0]hexane (**S21**) (47 mg, 0.20 mmol) and 4-iodotoluene (**6a**) (65 mg, 0.30 mmol). The crude product was purified by column chromatography (10:90 AcOEt/Hexane) to afford 4-methyl-*N*-(2-(*p*-tolyl)cyclopentyl) benzenosulfonamide **(S25)** as colorless oil, (14 mg, yield = **22%**).

^{1}H NMR (500 MHz, $CDCl_3$): δ 7.47 (d, *J* = 8.0 Hz, 2H), 7.11 (d, *J* = 7.9 Hz, 2H), 6.96 (d, *J* = 7.7 Hz, 2H), 6.86 (d, *J* = 7.7 Hz, 2H), 4.54 (d, *J* = 6.1 Hz, 1H), 3.47 – 3.37 (m, 1H), 2.69 (q, *J* = 9.4 Hz, 1H), 2.39 (s, 3H), 2.30 (s, 3H), 2.18 – 2.11 (m, 1H), 2.05 – 2.0 (m, 1H), 1.77 – 1.71 (m, 2H), 1.63 – 1.51 (m, 2H).

^{13}C NMR (125 MHz, $CDCl_3$): δ 143.0, 138.4, 137.3, 136.3, 129.5, 129.4, 127.2, 127.2, 61.5, 52.3, 33.3, 32.3, 22.1, 21.6, 21.2.

HRMS (ESI) $[M+Na]^+$ calculated for $C_{19}H_{23}NO_2SNa$: 352.1347, found: 352.1351.

GC Chromatogram: (99% purity)

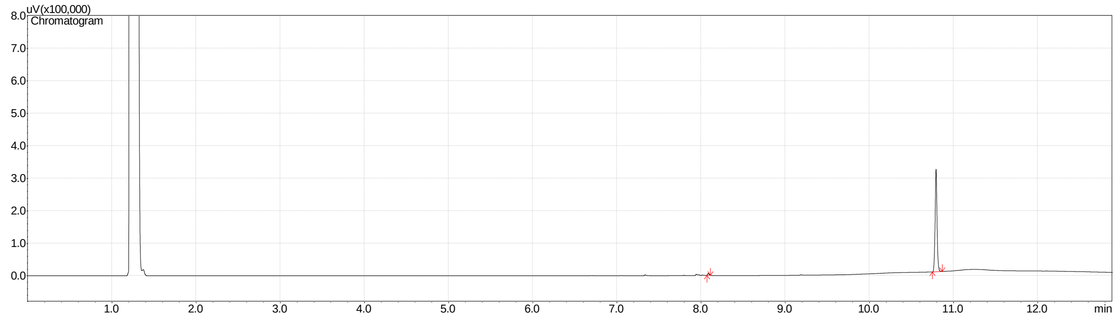

4-methyl-*N*-(1-(*p*-tolyl)hexan-2-yl)benzenosulfonamide (S26)

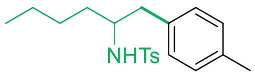

Following the general procedure **D** compound **S26** was obtained from 2-butyl-N-tosylaziridine (**S22**) (51 mg, 0.20 mmol) and 4-iodotoluene (**6a**) (65 mg, 0.30 mmol). The crude product was purified by column chromatography (10:90 AcOEt/Hexane) to afford **4**-methyl-*N*-(1-(*p*-tolyl)hexan-2-yl)benzenosulfonamide **(S26)** as colorless oil, (19 mg, yield = **28%**).

^{1}H NMR (500 MHz, CDCl$_3$): δ 7.63 (d, *J* = 8.0 Hz, 2H), 7.22 (d, *J* = 8.0 Hz, 2H), 7.00 (d, *J* = 7.6 Hz, 2H), 6.88 (d, *J* = 7.7 Hz, 2H), 4.22 (d, *J* = 8.1 Hz, 1H), 3.42 – 3.36 (m 1H), 2.61 (dd, *J* = 12.0, 4.0 Hz, 2H), 2.40 (s, 3H), 2.30 (s, 3H), 1.46 – 1.42 (m, 1H), 1.33 – 1.26 (m, 2H), 1.18 – 1.13 (m, 3H), 0.78 (t, *J* = 6.9 Hz, 3H).

^{13}C NMR (125 MHz, CDCl$_3$): δ 143.1, 138.1, 136.2, 134.1, 129.6, 129.5, 129.3, 127.2, 55.1, 40.9, 34.3, 27.7, 22.5, 21.6, 21.2, 14.0.

HRMS (ESI) [M+Na]$^+$ calculated for $C_{20}H_{27}NO_2SNa$: 368.1660, found: 368.1659.

GC Chromatogram: (98% purity)

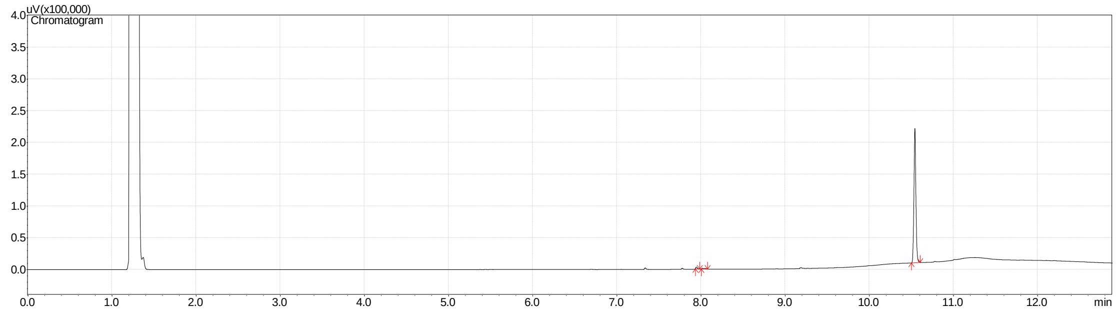

6. Mechanistic consideration

6.1. Proposed mechanism

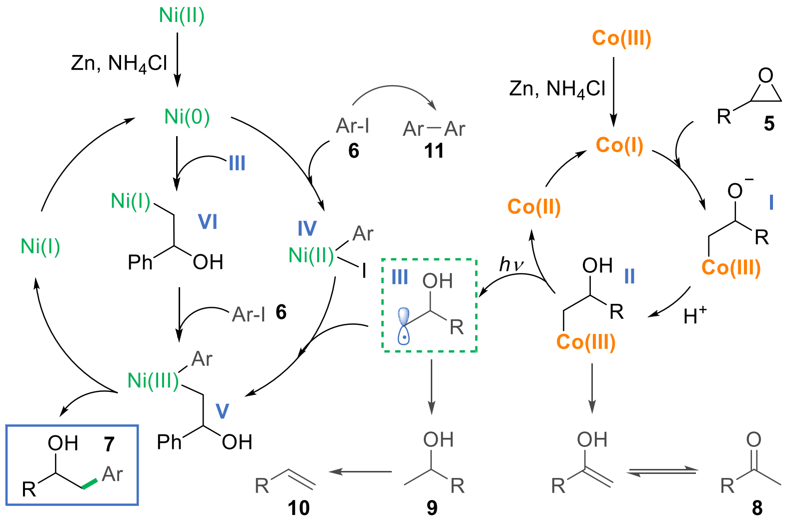

6.2 Mass spectrometry studies

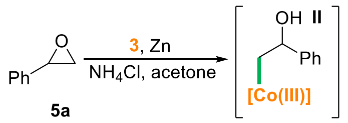

Reaction conditions: styrene oxide (**5a)** (0.2 mmol,) Zn (1.5 equiv.), NH_4Cl (3 equiv.), HME (5 mol%), acetone (c = 0.1 M), Blue LEDs, 30 min.

The reaction was setup according to the procedure **D** (without the addition of aryl halide, dttbpy and $NiCl_2$(DME). After 10 minutes of light irradiation a small portion was taken from the reaction mixture and was examined by LRMS.

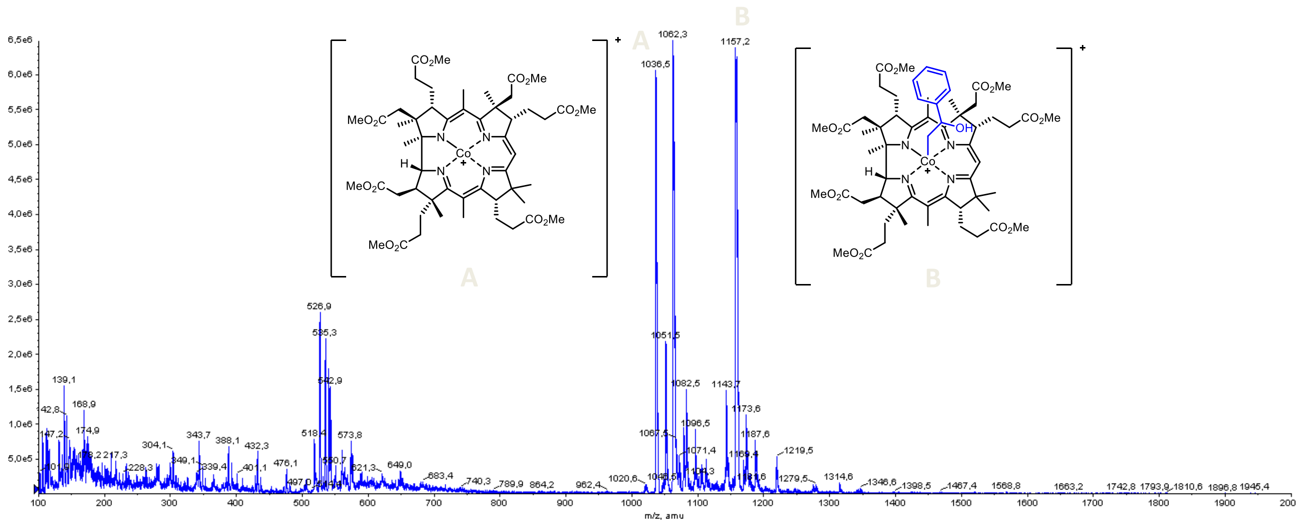

The LRMS spectrum of the reaction mixture recorded after 10 minutes under light irradiation indicates the presence of two forms of the catalyst: **A** and **B**. The first signal (**A**) corresponds to a catalyst lacking both axial ligands: H_2O and CN^-. The second signal (**B**) corresponds to the mass of the Co-alkyl complex.

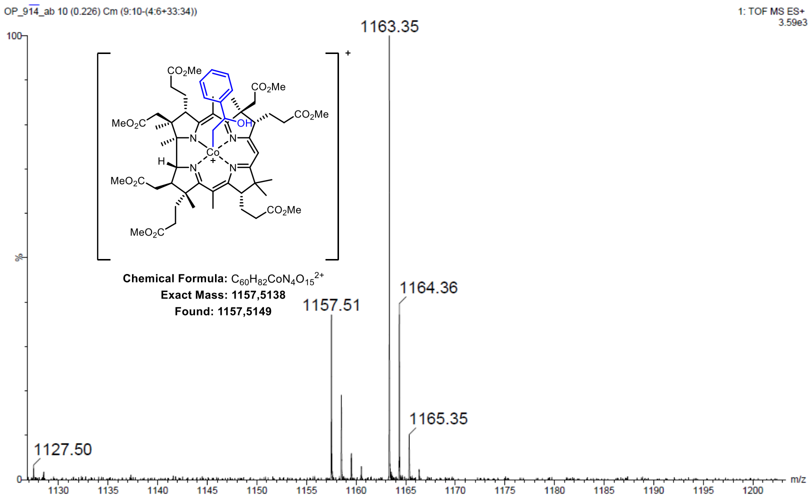

<u>Conclusion</u>: *Alkylcobalamin is an intermediate in this reaction generated via the nucleophilic attack of the Co(I) form of the catalyst on the epoxide.*

6.3 Studies of regioselectivity

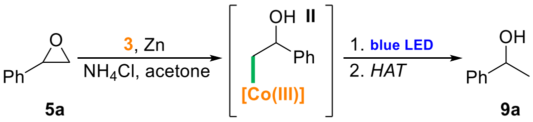

Reaction conditions: styrene oxide (**5a)** (0.2 mmol,) Zn (1.5 equiv.), NH_4Cl (3 equiv.), HME (5 mol%), acetone (c = 0.1 M), Blue LEDs, 16h.

Several experiments were performed to confirm the formation of the desired linear regioisomer. The reaction was setup according to procedure **D** (without addition of aryl halide, dttbpy and $NiCl_2DME$). After that time, the resulting mixture was diluted with AcOEt, filtered through the cotton wool. An aliquot was taken from the reaction mixture and was examined by GC-MS.

GC-MS from the reaction mixture:

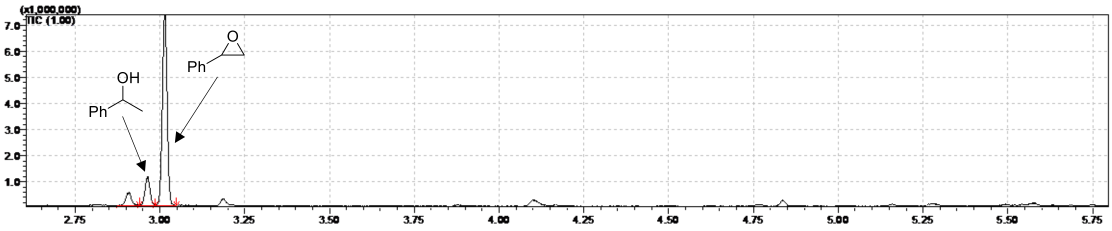

<u>Retention time of compound</u> **9a**: 2.965 min.

<u>Retention time of compound</u> **5a**: 3.015 min.

Fragmentation peaks detected for compound 9a:

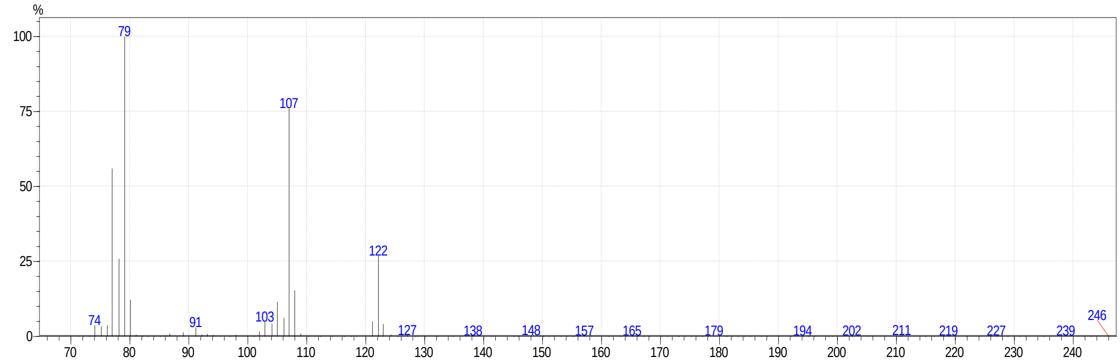

The obtained results were compared with commercially available *1-phenylethanol* and *2-phenylethanol*.

GC-MS of 1-phenylethanol:

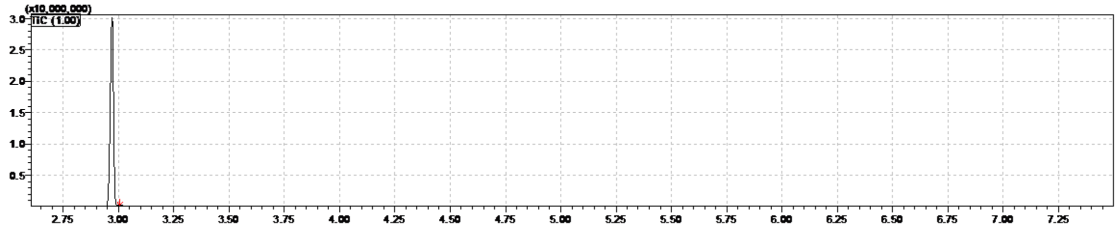

Retention time of **1-phenylethanol**: 2.972 min.

GC-MS of 2-phenylethanol:

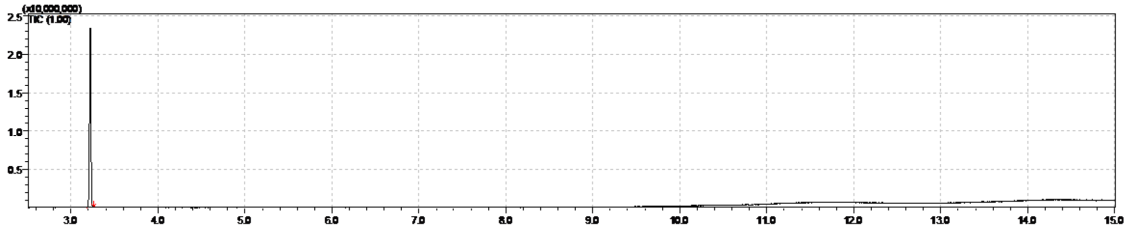

Retention time of **2-phenylethanol**: 3.222 min.

Fragmentation of 1-phenylethanol:

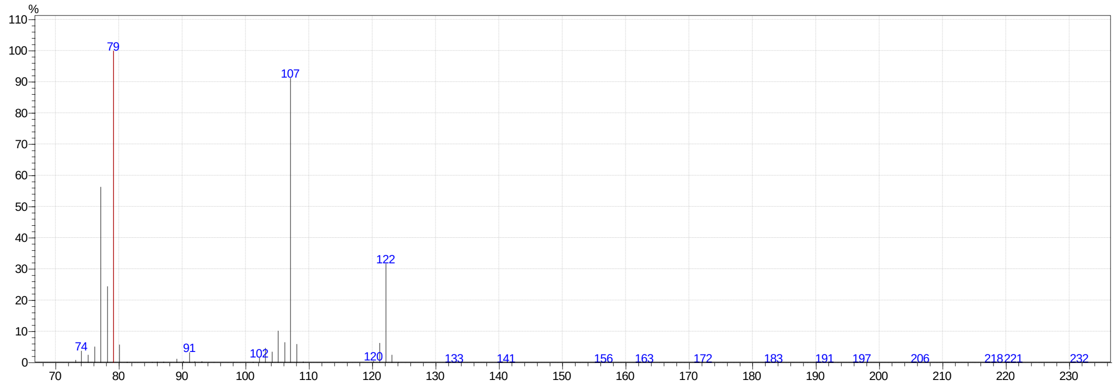

Fragmentation of 2-phenylethanol:

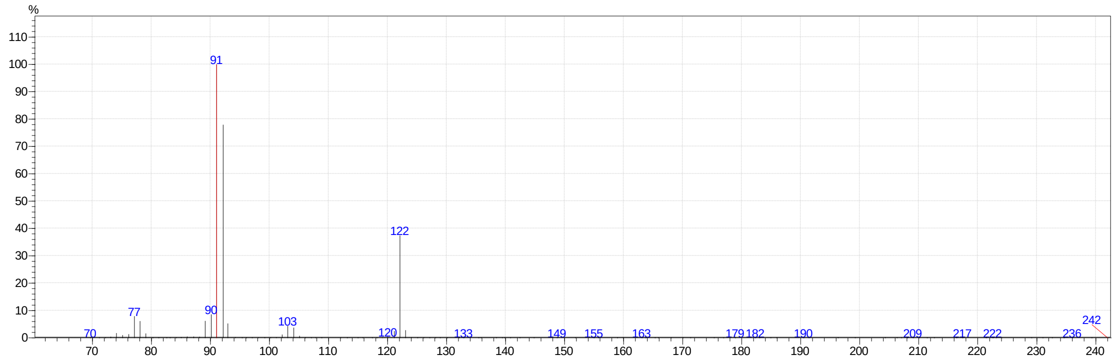

Comparison of the reaction mixture with 1-phenylethanol and 2-phenylethanol:

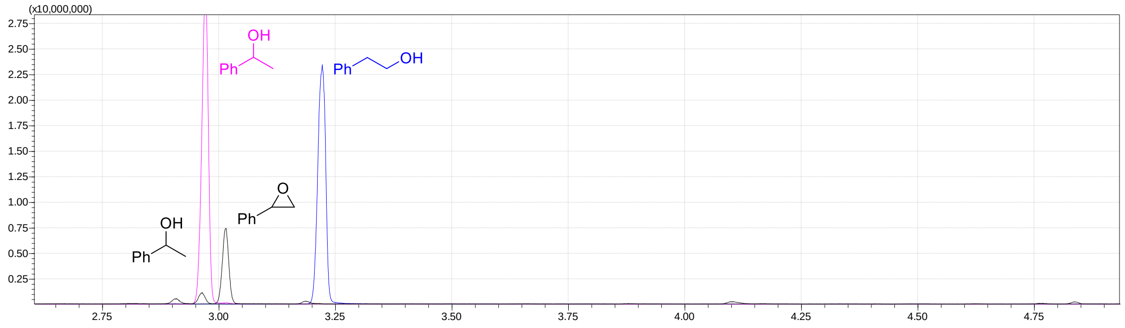

*<u>Conclusion</u>: The signal at 2.965 min. measured for compound **9a** overlaps with the signal corresponding to 1-phenylethanol (Sigma Aldrich) (2.972 min.). In addition, fragmentation peaks (MS) detected for compound **9a** are the same as for 1-phenylethanol. These experiments prove the formation of the linear isomer **9a** as a sole product.*

6.4 Stereoselectivity studies

Reaction with racemic styrene oxide: The model reaction with the racemic starting material was stopped after 15 minutes and the enantiomeric purity of both the substrate and the product was evaluated by HPLC. Both compounds were racemic corroborating that the kinetic resolution is not taking place in this reaction.

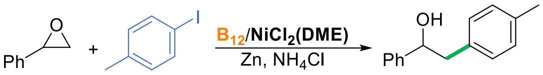

5a **6a** **7aa**

Reaction conditions: styrene oxide (**5a**) (0.2 mmol, 1 equiv.), 4-iodotoluene (**6a**) (1.5 equiv.), Zn (1.5 equiv.), NH_4Cl (3 equiv.), B_{12} (**1**) (5 mol%), $NiCl_2$(DME) (20 mol%), dtbbpy (40 mol%), water (1.1 equiv.), dry NMP (c = 0.1 M), blue LED, 30 min.

Racemic product **7aa**: Chiralpak ID-H, 10% AcOEt in hexanes, 40 min run, 1 mL/min

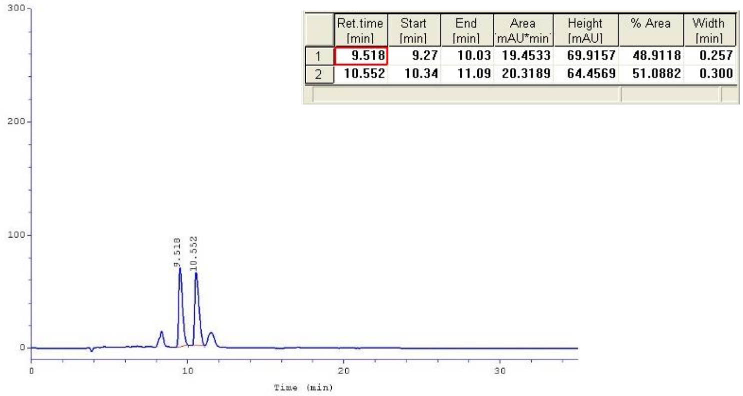

	Ret.time [min]	Start [min]	End [min]	Area mAU*min	Height [mAU]	% Area	Width [min]
1	9.518	9.27	10.03	19.4533	69.9157	48.9118	0.257
2	10.552	10.34	11.09	20.3189	64.4569	51.0882	0.300

Reaction with enantiomericall pure (R)-styrene oxide: The model reaction with (R)-styrene oxide gave expectadly enantiomerically pure product as the reaction does not involve the stereogenic center.

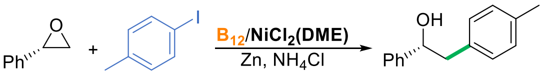

S27 **6a** **S28**

Reaction conditions: styrene oxide (**S27**) (0.2 mmol, 1 equiv.), 4-iodotoluene (**6a**) (1.5 equiv.), Zn (1.5 equiv.), NH_4Cl (3 equiv.), B_{12} (**1**) (5 mol%), $NiCl_2$(DME) (20 mol%), dtbbpy (40 mol%), water (1.1 equiv.), dry NMP (c = 0.1 M), blue LED, 30 min.

Reaction product (**S28**): Chiralpak ID-H, 10% AcOEt in hexanes, 40 min run, 1 mL/min

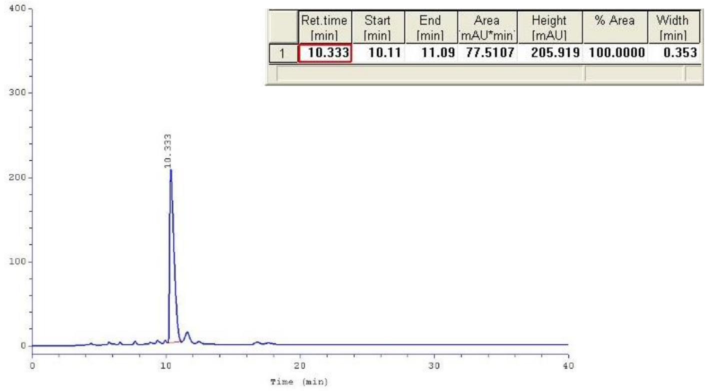

	Ret.time [min]	Start [min]	End [min]	Area mAU*min	Height [mAU]	% Area	Width [min]
1	10.333	10.11	11.09	77.5107	205.919	100.0000	0.353

<u>Conclusion</u>: Our observation indicates that the formation of a radical at the internal position of the aryl epoxide does not occur.

6.5 DFT calculations

Computational methods

DFT calculations were performed with Gaussian 16. Geometry optimizations were computed at BP86/6-31G(d) level of theory with the D3 version of Grimme's empirical dispersion correction[21] and solvation (acetone) with SMD model.[22] Frequency analysis was performed at the same level to provide correction to thermodynamic functions and confirm the nature of optimized structures (minima and transition states featured zero or one imaginary frequency, respectively). Single point energies were computed at BP86/6-311++G(2df,p) level of theory with the D3 version of Grimme's empirical dispersion correction and solvation (acetone) with SMD model. Molecular structures were visualized in CYLview.[23]

Performance of selected DFT methods

Performance of several commonly used functional (BP86, B3LYP, M06, PBE0 and wB97XD) was investigated and summarized in Tables 6.5.1 and 6.5.2. Geometry optimizations and frequency calculations were performed at BP86/6-31G(d) level of theory with the D3 version of Grimme's empirical dispersion correction[21] and solvation (acetone) with SMD model. Then, single point energies were computed using given functional (with or without dispersion correction) and 6-311++G(2df,p) bases set including solvation (acetone) with PCM model. Performance of BP86-D3 and xB97XD was compared in Scheme 6.5.1.

Table 6.5.1. Calculated Gibbs free energies (in kJ/mol, relative to substrates – styrene oxide and Co(I)-corin complex).

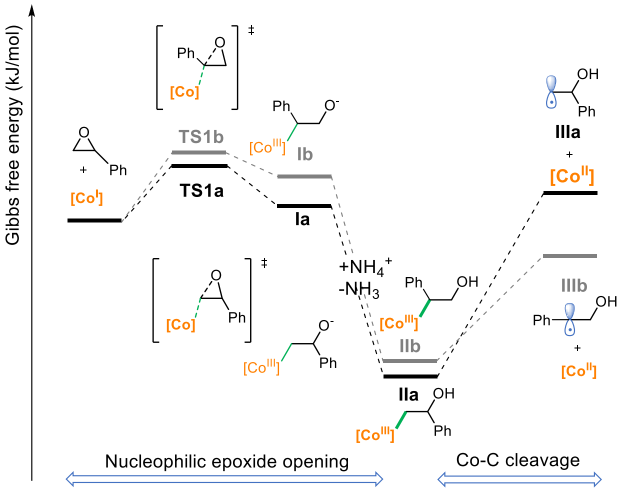

Entry	Functional	TS1a	TS1b	Ia	Ib	IIa	IIb	IIIa +[Co^{II}]	IIIb +[Co^{II}]
1	BP86-D3	43.7	51.9	12.7	34.8	-119.8	-107.7	22.1	-25.1
2	wB97XD	94.9	108.0	42.3	71.9	-131.4	-114.2	47.9	-92.6
3	M06-D3	81.6	88.9	45.0	69.5	-112.5	-96.5	-33.9	-82.0
4	M06	112.3	120.3	75.5	100.9	-80.9	-64.0	-33.0	-81.6
5	PBE0-D3	98.6	112.1	56.1	84.0	-108.4	-92.7	-70.5	-115.1
6	B3LYP-D3	84.4	92.2	40.6	65.1	-127.2	-112.8	-75.0	-122.5

Table 6.5.2. Comparison of the calculated barriers for epoxide ring opening and Gibbs free energies of homolysis of intermediate **Ia** and **IIa** (in kJ/mol).

Entry	Functional	Barrier height ($\Delta G^{\#}$) of styrene oxide opening with [Co^{I}]		ΔG of Co-C cleavage	
		TS1a	TS1b	in IIa	in IIb
1	BP86-D3	43.7	51.9	142.6	82.6
2	wB97XD	94.9	108.0	83.4	21.6
3	M06-D3	81.6	88.9	78.7	14.5
4	M06	112.3	120.3	47.9	-17.6
5	PBE0-D3	98.6	112.1	38.0	-22.4
6	B3LYP-D3	84.4	92.2	52.3	-9.7

BP86-D3 **wB97XD**

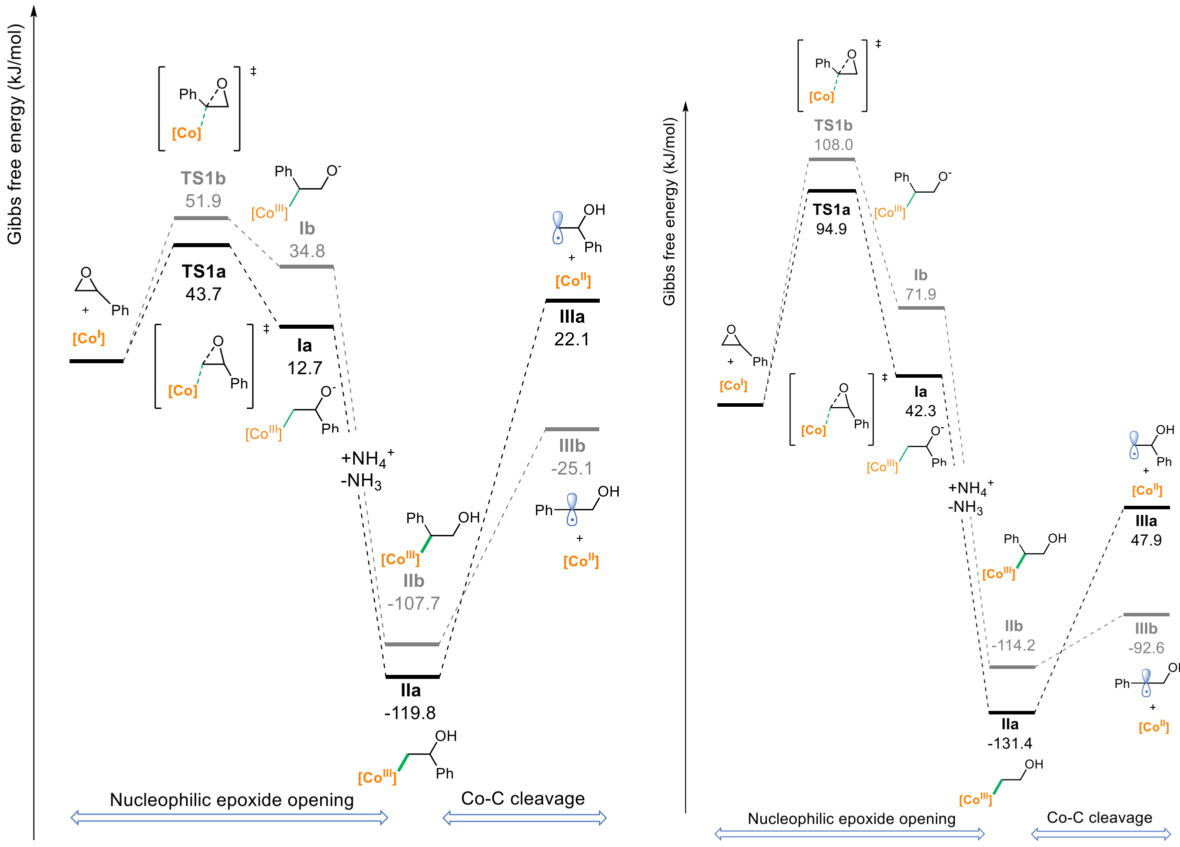

Scheme 6.5.1. Comparison of the reaction profiles calculated with BP86-D3 and xB97XD.

TD DFT calculations

Three lowest singlet and triplet exited states for intermediate **IIa** and **IIb** were calculated at TD-BP86-D3/6-311++G(2df,p) level of theory including solvation (acetone) with SMD model.

$(CH_3)_{15}$(corrin)Co(III)-CH_2-CH(OH)Ph – IIa (vertical excitation)

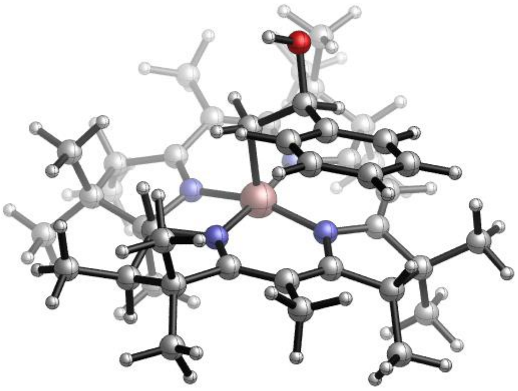

```
Excitation energies and oscillator strengths:
  Excited State   1:      Singlet-A      2.2027 eV  562.88 nm  f=0.0086  <S**2>=0.000
     185 -> 188         0.14945
     187 -> 188        -0.14429
     187 -> 189         0.66047
 Total Energy, E(TD-HF/TD-DFT) =  -3314.49012657

 Excited State   2:      Singlet-A      2.2693 eV  546.36 nm  f=0.0408  <S**2>=0.000
     185 -> 189        -0.21367
     186 -> 190         0.10502
     187 -> 188         0.63447
     187 -> 189         0.13240

 Excited State   3:      Singlet-A      2.3455 eV  528.60 nm  f=0.0118  <S**2>=0.000
     186 -> 188         0.67630
     187 -> 190         0.13075

Excitation energies and oscillator strengths:
   Excited State   1:      Triplet-A      1.6701 eV  742.39 nm  f=0.0000  <S**2>=2.000
     187 -> 188         0.70619
  Total Energy, E(TD-HF/TD-DFT) =  -3314.50969999

 Excited State   2:      Triplet-A      1.8431 eV  672.68 nm  f=0.0000  <S**2>=2.000
     187 -> 189         0.69916

 Excited State   3:      Triplet-A      2.0703 eV  598.87 nm  f=0.0000  <S**2>=2.000
     186 -> 188        -0.24033
     186 -> 189         0.65698
```

Simulated UV spectrum of IIa calculated for 20 and 50 excited states at TD-BP86/TZVP level of theory

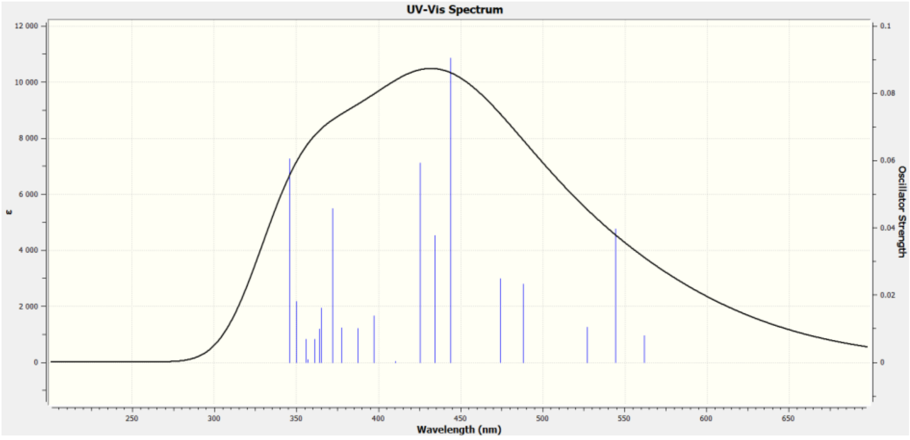

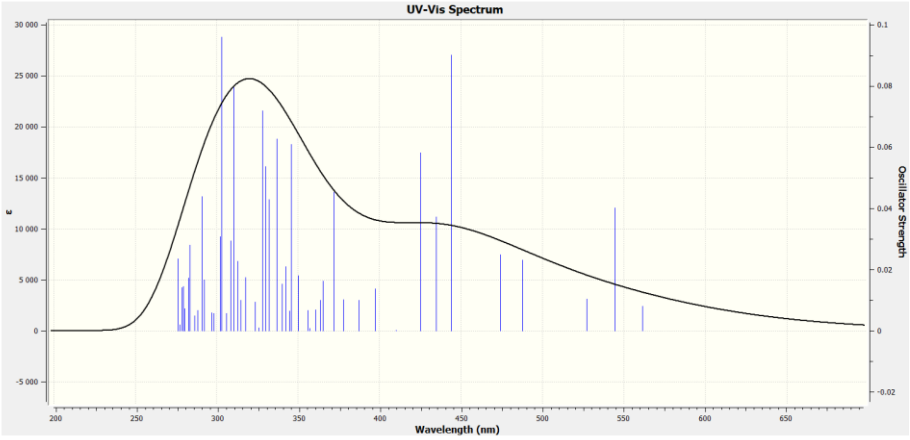

Performance of selected DFT methods

Performance of several commonly used functional, including hybrid and long-range corrected functionals (BP86, wB97XD, B3LYP, CAM-B3LYP M06, PBE0), in calculation of excited states for intermediate **IIa** was investigated. Calculated vertical excitation energies (in eV) for three lowest singlet excited states are summarized in Tables 6.5.3.

Table 6.5.3. Comparison of the calculated three lowest excitation states for intermediate **IIa**

Entry	Level of theory	S1	S2	S3
1	BP86/6-311++G(2df,p)	2.2027	2.2693	2.3455
2	BP86/TZPV	2.2076	2.2777	2.3518
3	wB97XD/6-311++G(2df,p)	2.2063	2.5045	3.0270
4	wB97XD/TZVP	2.2079	2.5142	3.0252
5	CAM-B3LYP/6-311++G(2df,p)	2.2285	2.5266	3.0574
6	CAM-B3LYP/TZVP	2.2304	2.5365	3.0569
7	B3LYP/6-311++G(2df,p)	2.2397	2.5336	2.8627
8	B3LYP/TZVP	2.2428	2.5431	2.8713
9	M06/6-311++G(2df,p)	1.8827	2.1827	2.5712
10	M06/TZVP	1.8968	2.2082	2.5955
11	PBE0/6-311++G(2df,p)	2.1888	2.4840	2.9338
12	PBE0/TZVP	2.1879	2.4904	2.9363

$(CH_3)_{15}$(corrin)Co(III)-CH_2-CH(OH)Ph – IIa-S1 (Relaxed)

Geometry of the **IIa-S1** was optimized at TD-BP86-D3/6-31G(d) (root=1) including solvation (acetone) with SMD model.

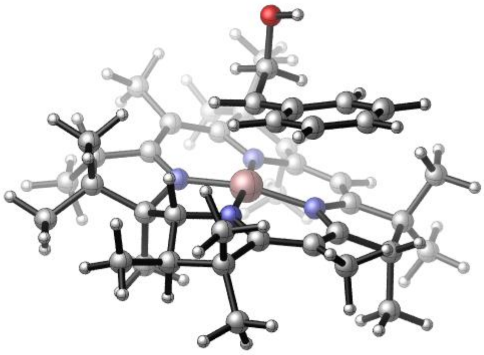

E (BP86-D3/6-31G(d) + SMD (acetone)) = -3313.808499
E (TD-BP86-D3/6-311++G(2df,p)//TD-BP86-D3/6-31G(d) + SMD (acetone)) = -3314.500880

Cartesian coordinates of the optimized geometry:
Charge = 1 Multiplicity = 1 Root=1

C	2.64652800	-1.90628700	0.22208100
C	2.88288400	-3.39037900	0.55391800
C	1.76460900	-4.06213000	-0.30393500
C	0.76337500	-2.94796600	-0.44260200
N	1.31973200	-1.72537900	-0.18954700
H	1.32061300	-4.92442900	0.22592800
C	-0.53845100	-3.15676300	-0.89210700
H	-0.82100200	-4.18682800	-1.12481600
C	3.56379300	-0.86277100	0.34886000
C	-1.47626500	-2.15775800	-1.14281200
C	-2.78431900	-2.43013900	-1.86085400
N	-1.30174700	-0.83193700	-0.83998200
C	-3.55940700	-1.09168000	-1.63851200
C	-2.50070700	-0.15682500	-1.06147600
C	3.20541900	0.46510200	-0.05725300
C	4.17737700	1.62109800	-0.21330700
N	1.95900700	0.82809400	-0.34733800
C	3.22784500	2.87510500	-0.18709700
C	1.86819000	2.27489400	-0.71940100
C	-2.73383700	1.17373300	-0.71587900
C	-1.63948300	2.04088400	-0.38833300
C	-1.70211400	3.56421800	-0.15743600
N	-0.39601300	1.59029100	-0.24613600
C	-0.20621900	3.95890000	-0.42763900
C	0.55866300	2.70010400	0.00131000
Co	0.35883100	-0.07840000	-0.22524000
H	-4.30470800	-1.26480700	-0.83601200
H	-0.11285200	4.06522400	-1.52493100
H	4.84615700	1.67551500	0.66497100
H	0.74848300	2.71752500	1.08908500
C	0.35925000	-0.14118700	1.94973300
C	-0.59131800	-1.19494200	2.44460000
H	-0.44372100	-2.12832500	1.87095500
O	-0.27224600	-1.61107700	3.82322600

C	-2.06402200	-0.81351500	2.35850200
C	-3.00735200	-1.75880700	1.91128500
C	-2.52924000	0.44965700	2.78023000
C	-4.37769600	-1.45379400	1.87120200
H	-2.65432800	-2.74227900	1.57814400
C	-3.89830000	0.75967800	2.74803500
H	-1.81687600	1.20227900	3.13672500
C	-4.82863900	-0.19009100	2.28992000
H	-5.09276200	-2.20196000	1.50964700
H	-4.23953400	1.74849100	3.07542100
H	-5.89634700	0.05448900	2.25720100
H	0.14767500	0.87401200	2.32466100
H	1.40610000	-0.42025000	2.16128100
C	-4.15528800	1.69881400	-0.68184600
H	-4.28306700	2.41357700	0.14553300
H	-4.45482900	2.21402400	-1.61223000
H	-4.87049300	0.88213100	-0.49823100
C	-2.10427200	3.82276000	1.31667400
H	-3.09653400	3.39539000	1.53702200
H	-1.38187600	3.37415800	2.02120500
H	-2.15272000	4.90854400	1.51282300
C	-2.61691200	4.35187900	-1.11602700
H	-3.67910500	4.30677000	-0.83026700
H	-2.32237800	5.41710700	-1.09788400
H	-2.51644400	3.99025200	-2.15484800
C	0.25432400	5.25985900	0.23230900
H	1.26651700	5.53431400	-0.10860300
H	-0.41890300	6.09573800	-0.03054700
H	0.28026200	5.17580200	1.33258200
C	1.69384400	2.36102000	-2.24893600
H	1.64141700	3.40708100	-2.59052300
H	2.53164800	1.86877900	-2.76671600
H	0.76279100	1.84481000	-2.54218400
C	3.75759200	4.05550100	-1.01507500
H	3.05218800	4.90432800	-0.98503900
H	4.71820300	4.40695300	-0.59658300
H	3.92774100	3.79600200	-2.07231200
C	3.12137300	3.31133300	1.29342100
H	4.12058800	3.61327400	1.65631900
H	2.44622200	4.17234000	1.42639700
H	2.76672100	2.48736200	1.94064900
C	5.08157900	1.42422200	-1.45058000
H	5.78503700	2.26826800	-1.55071500
H	5.67709900	0.50112400	-1.34147200
H	4.50089800	1.34115400	-2.38420700
C	4.96164900	-1.09158900	0.89105700
H	4.94682800	-1.75711000	1.76814000
H	5.64068700	-1.53909500	0.14336900
H	5.41691800	-0.14472400	1.21841700
C	4.26851400	-3.99357700	0.25716000
H	5.01360400	-3.71915000	1.01814000
H	4.18340700	-5.09504100	0.27482000
H	4.65405400	-3.69581800	-0.73197900
C	2.54933800	-3.57519900	2.06204400
H	1.53043000	-3.22538600	2.30420600
H	2.62478800	-4.64545200	2.32794900
H	3.25970700	-3.01196200	2.69188200
C	2.20166800	-4.51864500	-1.71747700
H	2.92665400	-5.34737800	-1.66025000
H	1.32439200	-4.87164200	-2.28672200

H	2.66160400	-3.68433700	-2.27667900
C	-4.30833300	-0.57741900	-2.88175400
H	-4.94316400	-1.38294300	-3.29133200
H	-4.97071800	0.26728900	-2.64328300
H	-3.61148700	-0.24991400	-3.67162300
C	-3.57002400	-3.64045400	-1.32254400
H	-4.53886000	-3.70737800	-1.85020600
H	-3.02968100	-4.58818200	-1.49390700
H	-3.77165600	-3.54249300	-0.24286000
C	-2.40322200	-2.69041800	-3.34698000
H	-1.76855500	-3.59099800	-3.41532100
H	-3.30845100	-2.86346800	-3.95386000
H	-1.84461000	-1.84255100	-3.78043300
H	-0.23545900	-0.77081600	4.33491500

Considerable elongation of Co-C bond was noticed upon relaxation of the S1-excited state (from 1.96 to 2.18 Å).

$(CH_3)_{15}$ (corrin)Co(III)-CHPh-CH_2OH – IIb (vertical excitation)

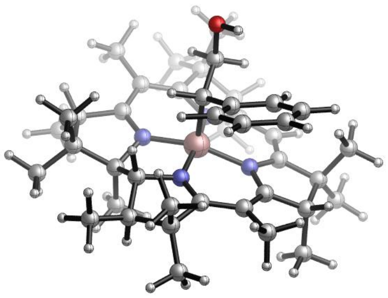

Excitation energies and oscillator strengths:

```
 Excited State   1:      Singlet-A      1.9362 eV  640.36 nm  f=0.0140  <S**2>=0.000
     187 -> 188        0.66537
     187 -> 189        0.19611
 Total Energy, E(TD-HF/TD-DFT) =  -3314.49920187

 Excited State   2:      Singlet-A      2.2025 eV  562.92 nm  f=0.0121  <S**2>=0.000
     185 -> 188       -0.12292
     186 -> 188        0.56061
     187 -> 188       -0.14307
     187 -> 189        0.35481

 Excited State   3:      Singlet-A      2.2371 eV  554.22 nm  f=0.0305  <S**2>=0.000
     185 -> 188       -0.27576
     186 -> 188       -0.40825
     187 -> 188       -0.10239
     187 -> 189        0.47369
```

Excitation energies and oscillator strengths:

```
 Excited State   1:      Triplet-A      1.5754 eV  787.02 nm  f=0.0000  <S**2>=2.000
     187 -> 188        0.70532
 Total Energy, E(TD-HF/TD-DFT) =  -3314.51246127

 Excited State   2:      Triplet-A      1.6767 eV  739.46 nm  f=0.0000  <S**2>=2.000
     187 -> 189        0.69909
```

Excited State 3: Triplet-A 1.7828 eV 695.45 nm f=0.0000 <S**2>=2.000
186 -> 188 0.68287
186 -> 189 0.16261

$(CH_3)_{15}$ (corrin)Co(III)-CHPh-CH_2OH – IIb_S1 (Relaxed)

Geometry of the **IIb-S1** was optimized at TD-BP86-D3/6-31G(d) (root=1) including solvation (acetone) with SMD model.

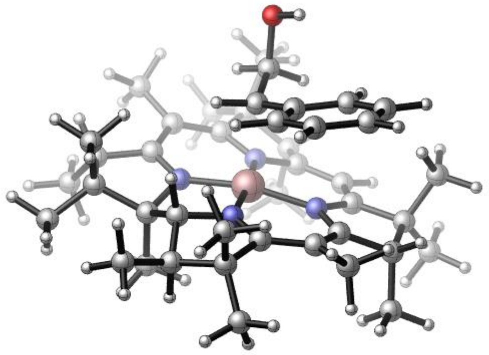

E (BP86-D3/6-31G(d) + SMD (acetone)) = -3313.818385
E (TD-BP86-D3/6-311++G(2df,p)//TD-BP86-D3/6-31G(d) + SMD (acetone)) = -3314.293878

Cartesian coordinates of the optimized geometry:
Charge = 1 Multiplicity = 1 Root=1

C	2.64652800	-1.90628700	0.22208100
C	2.88288400	-3.39037900	0.55391800
C	1.76460900	-4.06213000	-0.30393500
C	0.76337500	-2.94796600	-0.44260200
N	1.31973200	-1.72537900	-0.18954700
H	1.32061300	-4.92442900	0.22592800
C	-0.53845100	-3.15676300	-0.89210700
H	-0.82100200	-4.18682800	-1.12481600
C	3.56379300	-0.86277100	0.34886000
C	-1.47626500	-2.15775800	-1.14281200
C	-2.78431900	-2.43013900	-1.86085400
N	-1.30174700	-0.83193700	-0.83998200
C	-3.55940700	-1.09168000	-1.63851200
C	-2.50070700	-0.15682500	-1.06147600
C	3.20541900	0.46510200	-0.05725300
C	4.17737700	1.62109800	-0.21330700
N	1.95900700	0.82809400	-0.34733800
C	3.22784500	2.87510500	-0.18709700
C	1.86819000	2.27489400	-0.71940100
C	-2.73383700	1.17373300	-0.71587900
C	-1.63948300	2.04088400	-0.38833300
C	-1.70211400	3.56421800	-0.15743600
N	-0.39601300	1.59029100	-0.24613600
C	-0.20621900	3.95890000	-0.42763900
C	0.55866300	2.70010400	0.00131000
Co	0.35883100	-0.07840000	-0.22524000
H	-4.30470800	-1.26480700	-0.83601200
H	-0.11285200	4.06522400	-1.52493100

H	4.84615700	1.67551500	0.66497100
H	0.74848300	2.71752500	1.08908500
C	0.35925000	-0.14118700	1.94973300
C	-0.59131800	-1.19494200	2.44460000
H	-0.44372100	-2.12832500	1.87095500
O	-0.27224600	-1.61107700	3.82322600
C	-2.06402200	-0.81351500	2.35850200
C	-3.00735200	-1.75880700	1.91128500
C	-2.52924000	0.44965700	2.78023000
C	-4.37769600	-1.45379400	1.87120200
H	-2.65432800	-2.74227900	1.57814400
C	-3.89830000	0.75967800	2.74803500
H	-1.81687600	1.20227900	3.13672500
C	-4.82863900	-0.19009100	2.28992000
H	-5.09276200	-2.20196000	1.50964700
H	-4.23953400	1.74849100	3.07542100
H	-5.89634700	0.05448900	2.25720100
H	0.14767500	0.87401200	2.32466100
H	1.40610000	-0.42025000	2.16128100
C	-4.15528800	1.69881400	-0.68184600
H	-4.28306700	2.41357700	0.14553300
H	-4.45482900	2.21402400	-1.61223000
H	-4.87049300	0.88213100	-0.49823100
C	-2.10427200	3.82276000	1.31667400
H	-3.09653400	3.39539000	1.53702200
H	-1.38187600	3.37415800	2.02120500
H	-2.15272000	4.90854400	1.51282300
C	-2.61691200	4.35187900	-1.11602700
H	-3.67910500	4.30677000	-0.83026700
H	-2.32237800	5.41710700	-1.09788400
H	-2.51644400	3.99025200	-2.15484800
C	0.25432400	5.25985900	0.23230900
H	1.26651700	5.53431400	-0.10860300
H	-0.41890300	6.09573800	-0.03054700
H	0.28026200	5.17580200	1.33258200
C	1.69384400	2.36102000	-2.24893600
H	1.64141700	3.40708100	-2.59052300
H	2.53164800	1.86877900	-2.76671600
H	0.76279100	1.84481000	-2.54218400
C	3.75759200	4.05550100	-1.01507500
H	3.05218800	4.90432800	-0.98503900
H	4.71820300	4.40695300	-0.59658300
H	3.92774100	3.79600200	-2.07231200
C	3.12137300	3.31133300	1.29342100
H	4.12058800	3.61327400	1.65631900
H	2.44622200	4.17234000	1.42639700
H	2.76672100	2.48736200	1.94064900
C	5.08157900	1.42422200	-1.45058000
H	5.78503700	2.26826800	-1.55071500
H	5.67709900	0.50112400	-1.34147200
H	4.50089800	1.34115400	-2.38420700
C	4.96164900	-1.09158900	0.89105700
H	4.94682800	-1.75711000	1.76814000
H	5.64068700	-1.53909500	0.14336900
H	5.41691800	-0.14472400	1.21841700
C	4.26851400	-3.99357700	0.25716000
H	5.01360400	-3.71915000	1.01814000
H	4.18340700	-5.09504100	0.27482000
H	4.65405400	-3.69581800	-0.73197900
C	2.54933800	-3.57519900	2.06204400

H	1.53043000	-3.22538600	2.30420600
H	2.62478800	-4.64545200	2.32794900
H	3.25970700	-3.01196200	2.69188200
C	2.20166800	-4.51864500	-1.71747700
H	2.92665400	-5.34737800	-1.66025000
H	1.32439200	-4.87164200	-2.28672200
H	2.66160400	-3.68433700	-2.27667900
C	-4.30833300	-0.57741900	-2.88175400
H	-4.94316400	-1.38294300	-3.29133200
H	-4.97071800	0.26728900	-2.64328300
H	-3.61148700	-0.24991400	-3.67162300
C	-3.57002400	-3.64045400	-1.32254400
H	-4.53886000	-3.70737800	-1.85020600
H	-3.02968100	-4.58818200	-1.49390700
H	-3.77165600	-3.54249300	-0.24286000
C	-2.40322200	-2.69041800	-3.34698000
H	-1.76855500	-3.59099800	-3.41532100
H	-3.30845100	-2.86346800	-3.95386000
H	-1.84461000	-1.84255100	-3.78043300
H	-0.23545900	-0.77081600	4.33491500

Exclusion of the ring opening of epoxide via protonation

The pathway involving protonation of the epoxide was not considered as accessible under developed conditions. The pKa of protonated epoxide is estimated to be ~ -3, while the pKa of NH_4^+ is 10.5 and 9.2 in DMSO and water, respectively. We calculated ΔG for protonation of styrene oxide, which was found to be 86.2 kJ/mol. The calculated geometry of the protonated styrene oxide resembles a benzyl carbocation stabilized by neighboring OH group (opened epoxide ring). It is consistent with the textbook fact that acid mediated ring opening of epoxides proceeds according to Markovnikov's rule, i.e. at side delivering more stabilized carbocation (usually more substituted).

Calculated geometry of protonated styrene oxide:

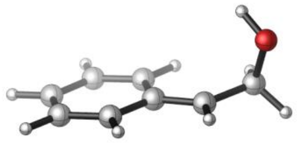

E (BP86-D3/6-31G(d) + SMD (acetone)) = -385.285837
E (BP86-D3/6-311++G(2df,p)// BP86-D3/6-31G(d) + SMD (acetone)) = -385.409967

Zero-point correction=	0.146957
Thermal correction to Energy=	0.155201
Thermal correction to Enthalpy=	0.156145
Thermal correction to Gibbs Free Energy=	0.113745

Charge = 0 Multiplicity = 1

C	2.52508100	-0.32356700	0.48023600
C	1.34736500	0.58964600	0.41982600
H	1.60472200	1.65632600	0.45594700
O	3.18431400	0.04159200	-0.75873000
C	0.00844400	0.25230700	0.18870500
C	-0.94453700	1.32364900	0.04286300
C	-0.45038400	-1.11210500	0.13629900
C	-2.29251200	1.03990500	-0.13004300

H	-0.58653800	2.35775500	0.08003100
C	-1.80146700	-1.37683100	-0.03800200
H	0.26369200	-1.93492600	0.23109500
C	-2.71785500	-0.30686500	-0.17148200
H	-3.02156100	1.84837300	-0.23661000
H	-2.16113200	-2.40905600	-0.07748900
H	-3.78111000	-0.52890200	-0.31358100
H	2.29071700	-1.39895300	0.53681200
H	3.20098100	-0.05361700	1.30746800
H	2.67090600	-0.38656700	-1.48424900

Optimized geometries, energies and corrections to thermodynamic functions

$(CH_3)_{15}$(corrin)Co(I)

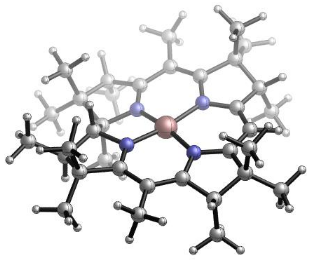

E (BP86-D3/6-31G(d) + SMD (acetone)) = -2928.477102
E (BP86-D3/6-311++G(2df,p)// BP86-D3/6-31G(d) + SMD (acetone)) = -2929.055777
E (wB97XD/6-311++G(2df,p)// BP86-D3/6-31G(d) + SMD (acetone)) = -2928.240361
E (M06-D3/6-311++G(2df,p)// BP86-D3/6-31G(d) + SMD (acetone)) = -2927.454493
E (M06/6-311++G(2df,p)// BP86-D3/6-31G(d) + SMD (acetone)) = -2927.427618
E (PBE0-D3/6-311++G(2df,p)// BP86-D3/6-31G(d) + SMD (acetone)) = -2926.656664
E (B3LYP-D3/6-311++G(2df,p)// BP86-D3/6-31G(d) + SMD (acetone)) = -2928.828969

Zero-point correction=	0.777157
Thermal correction to Energy=	0.816751
Thermal correction to Enthalpy=	0.817695
Thermal correction to Gibbs Free Energy=	0.712569

Charge = 0 Multiplicity = 1

C	2.89796800	-0.88780100	-0.29370100
C	4.32173700	-0.37091400	-0.60043500
C	4.26146600	1.04280500	0.05023900
C	2.78668000	1.34831300	-0.03665400
N	2.02435100	0.20638400	-0.10672600
H	4.86199000	1.76519300	-0.53452600
C	2.28150400	2.64274500	0.00507500
H	3.00938400	3.45996900	0.03233000
C	2.49581400	-2.21796200	-0.27565700
C	0.93192300	2.97282400	0.00182100
C	0.41558000	4.40299700	-0.09568000
N	-0.08484000	2.04349700	-0.00739900
C	-1.04433100	4.20901300	0.39642400
C	-1.30331300	2.74106100	0.06831100
C	1.14056600	-2.55441400	0.03138200
C	0.63340400	-3.96386300	0.29524200
N	0.17817600	-1.63445000	0.13849800
C	-0.91955900	-3.80045100	0.12579600
C	-1.12962700	-2.28283300	0.49382800
C	-2.57194400	2.18758600	-0.05012400
C	-2.73328400	0.77184300	-0.18724400
C	-4.05142100	0.00672400	-0.41839000
N	-1.67220300	-0.04597000	-0.18285700
C	-3.61678200	-1.44904000	-0.03270900
C	-2.11827700	-1.45953100	-0.35841900
Co	0.13336500	0.17296300	-0.04823000
H	-1.71663700	4.88538300	-0.15999200
H	-3.73246600	-1.51982700	1.06567300
H	0.99062700	-4.65251800	-0.49373500
H	-1.96420400	-1.71536400	-1.42233300
C	-3.79101700	3.09284600	0.00182300

H	-4.51492300	2.84461300	-0.79279100
H	-4.32978600	3.01926400	0.96461900
H	-3.52131000	4.15041400	-0.13712200
C	-4.43590500	0.12490000	-1.91464900
H	-4.59654400	1.18169800	-2.19153400
H	-3.64312200	-0.27819800	-2.57058100
H	-5.37222600	-0.42294900	-2.12703600
C	-5.24695500	0.41387400	0.46969700
H	-5.76248400	1.31941700	0.11370500
H	-5.99292000	-0.40305300	0.46704900
H	-4.93237000	0.57916600	1.51585900
C	-4.44128700	-2.56548000	-0.67890500
H	-4.18115300	-3.54867900	-0.25168700
H	-5.52220300	-2.41011000	-0.50606100
H	-4.27712700	-2.61879900	-1.76960100
C	-1.38260500	-2.04245800	1.99732700
H	-2.32559900	-2.50050400	2.33898000
H	-0.55990700	-2.45384000	2.60306800
H	-1.42483200	-0.95560100	2.18485700
C	-1.73376000	-4.77629800	0.99049100
H	-2.81733400	-4.60407000	0.86501600
H	-1.52598400	-5.81828700	0.68426800
H	-1.50399400	-4.69289500	2.06506400
C	-1.23281300	-4.08467200	-1.36264900
H	-0.96136700	-5.12968600	-1.60087500
H	-2.30269900	-3.95969600	-1.59728700
H	-0.65197000	-3.42751500	-2.03584800
C	1.15661600	-4.52716500	1.63469100
H	0.78126300	-5.55148500	1.80715600
H	2.25983700	-4.57480700	1.61793900
H	0.86580600	-3.90210000	2.49546100
C	3.45703000	-3.35090500	-0.60068500
H	4.08496000	-3.11231000	-1.47469100
H	4.13653100	-3.59842100	0.23523700
H	2.90916500	-4.27248500	-0.85291600
C	5.52089100	-1.18313700	-0.07330000
H	5.73864700	-2.06495700	-0.69449100
H	6.42336700	-0.54449700	-0.09799200
H	5.37320300	-1.52206400	0.96613700
C	4.44591300	-0.20672200	-2.13932100
H	3.66116000	0.46599000	-2.53154300
H	5.43087900	0.22471300	-2.40027400
H	4.35032400	-1.18040000	-2.65180700
C	4.70988000	1.11033600	1.52840100
H	5.78860400	0.90417200	1.64316000
H	4.51277900	2.11982500	1.93153100
H	4.14863300	0.38401800	2.14467200
C	-1.25862600	4.43909100	1.90851700
H	-1.06322600	5.48925700	2.18950700
H	-2.30000800	4.20052500	2.18753500
H	-0.59135600	3.78990500	2.50458000
C	0.39917500	4.81064500	-1.59374900
H	-0.01522400	5.82979400	-1.71325500
H	1.42166100	4.80005900	-2.01263800
H	-0.22277700	4.11500600	-2.18698900
C	1.23856100	5.43001300	0.69864200
H	2.23817100	5.56491700	0.24732400
H	0.73849600	6.41588500	0.68622800
H	1.38182400	5.12587900	1.74962900

Styrene oxide (5a)

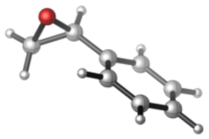

E (BP86-D3/6-31G(d) + SMD (acetone)) = -384.864773
E (BP86-D3/6-311++G(2df,p)// BP86-D3/6-31G(d) + SMD (acetone)) = -384.988857
E (wB97XD/6-311++G(2df,p)// BP86-D3/6-31G(d) + SMD (acetone)) = -384.843489
E (M06-D3/6-311++G(2df,p)// BP86-D3/6-31G(d) + SMD (acetone)) = -384.694325
E (M06/6-311++G(2df,p)// BP86-D3/6-31G(d) + SMD (acetone)) = -384.692796
E (PBE0-D3/6-311++G(2df,p)// BP86-D3/6-31G(d) + SMD (acetone)) = -384.530381
E (B3LYP-D3/6-311++G(2df,p)// BP86-D3/6-31G(d) + SMD (acetone)) = -384.99321

Zero-point correction=	0.134965
Thermal correction to Energy=	0.142345
Thermal correction to Enthalpy=	0.143289
Thermal correction to Gibbs Free Energy=	0.102785

Charge = 0 Multiplicity = 1

C	2.59437100	-0.04897300	0.73917500
C	1.60686700	0.60965100	-0.15344600
H	1.84497600	1.62556100	-0.50545200
O	2.50403700	-0.42054900	-0.65567200
C	0.15006000	0.27919200	-0.09355000
C	-0.80201500	1.31717800	-0.04163800
C	-0.28956500	-1.06077600	-0.06972200
C	-2.17191700	1.02083600	0.05138800
H	-0.46623700	2.36109000	-0.07257900
C	-1.65808000	-1.35582500	0.01841700
H	0.45175900	-1.86484600	-0.13803000
C	-2.60395800	-0.31618800	0.08296500
H	-2.90277300	1.83667500	0.09291900
H	-1.98905300	-2.40072700	0.03241600
H	-3.67296900	-0.54792500	0.15065200
H	2.23834300	-0.80622300	1.45422700
H	3.50907200	0.49023100	1.02968200

$(CH_3)_{15}$(corrin)Co(I) - styrene oxide – TS1a

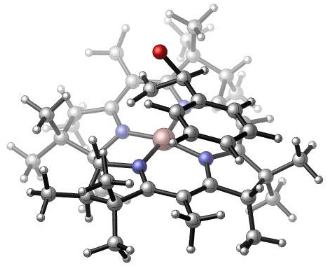

E (BP86-D3/6-31G(d) + SMD (acetone)) = -3313.352516
E (BP86-D3/6-311++G(2df,p)// BP86-D3/6-31G(d) + SMD (acetone)) = -3314.051616
E (wB97XD/6-311++G(2df,p)// BP86-D3/6-31G(d) + SMD (acetone)) = -3313.071329
E (M06-D3/6-311++G(2df,p)// BP86-D3/6-31G(d) + SMD (acetone)) = -3312.141359

E (M06/6-311++G(2df,p)// BP86-D3/6-31G(d) + SMD (acetone)) = -3312.134298
E (PBE0-D3/6-311++G(2df,p)// BP86-D3/6-31G(d) + SMD (acetone)) = -3311.203294
E (B3LYP-D3/6-311++G(2df,p)// BP86-D3/6-31G(d) + SMD (acetone)) = -3313.83971

Zero-point correction=	0.912471
Thermal correction to Energy=	0.960448
Thermal correction to Enthalpy=	0.961392
Thermal correction to Gibbs Free Energy=	0.838993

Charge = 0 Multiplicity = 1

C	2.66075000	-1.88004100	0.21567600
C	2.92143400	-3.34429900	0.63074000
C	1.81071700	-4.08364700	-0.17857200
C	0.78609600	-2.98954300	-0.36804900
N	1.32655100	-1.74131100	-0.21360400
H	1.39890300	-4.92420400	0.41129000
C	-0.51742000	-3.23896300	-0.78725700
H	-0.78922900	-4.28450300	-0.96538500
C	3.55661100	-0.82082900	0.31870300
C	-1.47963000	-2.26908300	-1.05293600
C	-2.80783600	-2.59163900	-1.72285500
N	-1.30735400	-0.93050300	-0.80936800
C	-3.59344800	-1.25249800	-1.52741900
C	-2.53062800	-0.27940000	-1.01543300
C	3.17070900	0.49808900	-0.09331300
C	4.12082300	1.67224100	-0.26639700
N	1.91228400	0.81991000	-0.38165700
C	3.14183500	2.90207200	-0.25880600
C	1.80200700	2.26124300	-0.79036400
C	-2.78218500	1.05663600	-0.71939900
C	-1.70303200	1.95544100	-0.44015000
C	-1.79489800	3.48216400	-0.23612100
N	-0.44316700	1.52362600	-0.34376900
C	-0.31604400	3.89939100	-0.55694200
C	0.47877400	2.66945400	-0.10504300
Co	0.35013200	-0.12038700	-0.36851000
H	-4.32713500	-1.40760900	-0.71051100
H	-0.25028100	3.96915300	-1.65996500
H	4.78390100	1.76094300	0.61448000
H	0.65415700	2.72562600	0.98471600
C	0.50260600	-0.03356300	2.05436400
C	-0.46569100	-1.00662300	2.61104900
H	-0.26219200	-2.06126800	2.31886900
O	0.18293000	-0.53909400	3.75944400
C	-1.94541400	-0.70921400	2.50708500
C	-2.84722400	-1.68258000	2.04259100
C	-2.44797200	0.53818300	2.92784300
C	-4.22569900	-1.41491100	1.98349600
H	-2.45948400	-2.65474800	1.71448000
C	-3.82114700	0.81539900	2.86141600
H	-1.75032800	1.28265100	3.32739000
C	-4.71652500	-0.16213000	2.38764400
H	-4.91630900	-2.18595900	1.62222500
H	-4.19745500	1.79297500	3.18473100
H	-5.79046500	0.05154100	2.33939700
H	0.25426200	1.02696800	2.11383300
H	1.55300300	-0.31920300	1.99010000
C	-4.21546700	1.55760200	-0.66855900
H	-4.33920600	2.29449500	0.14069300
H	-4.55249300	2.04040700	-1.60427000

H	-4.91273000	0.73520100	-0.44239600
C	-2.16815500	3.78276700	1.23659700
H	-3.15024400	3.34857900	1.48824100
H	-1.42837800	3.35946400	1.93910200
H	-2.22706000	4.87276300	1.40972700
C	-2.74642900	4.23112500	-1.19183400
H	-3.80231700	4.16839000	-0.88514100
H	-2.47798100	5.30403300	-1.20512900
H	-2.65999600	3.84820400	-2.22466500
C	0.13067700	5.23267400	0.04651400
H	1.12658000	5.52195300	-0.32899100
H	-0.56952500	6.04460700	-0.22299200
H	0.18789900	5.18638000	1.14815600
C	1.65425700	2.31715200	-2.32437900
H	1.58402800	3.35461100	-2.69122600
H	2.51005500	1.83180000	-2.81957300
H	0.74061500	1.77330300	-2.62155200
C	3.65318000	4.08607900	-1.09456000
H	2.93215500	4.92215300	-1.08034600
H	4.60396000	4.46232300	-0.67383000
H	3.83723800	3.82024600	-2.14807000
C	3.01603300	3.35511600	1.21509300
H	4.00437400	3.68801600	1.58195800
H	2.31695500	4.19943900	1.33431100
H	2.67759000	2.52999200	1.86914000
C	5.04095600	1.48911300	-1.49275900
H	5.72825000	2.34628600	-1.60315100
H	5.65696200	0.58085700	-1.36825900
H	4.47164100	1.37786100	-2.43085300
C	4.96099000	-1.00951300	0.87006200
H	4.95894000	-1.63238900	1.77924700
H	5.64973000	-1.48249900	0.14668800
H	5.40635300	-0.04345100	1.15416400
C	4.31561500	-3.94096500	0.35375300
H	5.06550300	-3.61261000	1.08880000
H	4.25480000	-5.04217500	0.43034900
H	4.68744200	-3.69007800	-0.65409600
C	2.60734100	-3.46219600	2.14661000
H	1.57577400	-3.13730500	2.36971500
H	2.71717700	-4.51219000	2.47748500
H	3.29556900	-2.83876200	2.74430800
C	2.24415600	-4.61973000	-1.56262300
H	2.98621200	-5.43241000	-1.47600600
H	1.36636100	-5.01982700	-2.10051900
H	2.68005600	-3.81278800	-2.17974700
C	-4.37268300	-0.79145300	-2.77397500
H	-5.01727700	-1.61102000	-3.14000600
H	-5.02992900	0.06381200	-2.55780500
H	-3.69453300	-0.49508800	-3.59265700
C	-3.55963500	-3.77791300	-1.08918800
H	-4.55030700	-3.89145900	-1.56837700
H	-3.01386700	-4.72917900	-1.22573500
H	-3.71794900	-3.62328100	-0.00800900
C	-2.49812300	-2.92691200	-3.20633300
H	-1.85206500	-3.82178900	-3.25415600
H	-3.41956200	-3.14441400	-3.77550800
H	-1.96504300	-2.09942700	-3.70747400

(CH_3)$_{15}$(corrin)Co(I) - styrene oxide – TS1b

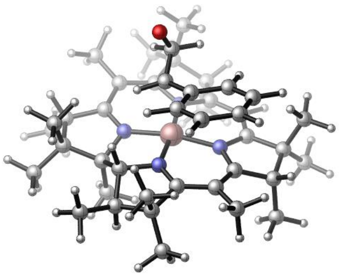

E (BP86-D3/6-31G(d) + SMD (acetone)) = -3313.346153
E (BP86-D3/6-311++G(2df,p)// BP86-D3/6-31G(d) + SMD (acetone)) = -3314.048305
E (wB97XD/6-311++G(2df,p)// BP86-D3/6-31G(d) + SMD (acetone)) = -3313.066167
E (M06-D3/6-311++G(2df,p)// BP86-D3/6-31G(d) + SMD (acetone)) = -3312.138419
E (M06/6-311++G(2df,p)// BP86-D3/6-31G(d) + SMD (acetone)) = -3312.098033
E (PBE0-D3/6-311++G(2df,p)// BP86-D3/6-31G(d) + SMD (acetone)) = -3311.167819
E (B3LYP-D3/6-311++G(2df,p)// BP86-D3/6-31G(d) + SMD (acetone)) = -3313.810532

Zero-point correction=	0.912828
Thermal correction to Energy=	0.960938
Thermal correction to Enthalpy=	0.961883
Thermal correction to Gibbs Free Energy=	0.838808

Charge = 0 Multiplicity = 1

C	3.09586900	-0.98180400	0.12629100
C	3.93977200	-2.27050700	0.24968300
C	3.11217500	-3.23764300	-0.65189400
C	1.73075400	-2.63836800	-0.54651800
N	1.77270500	-1.30186000	-0.24025100
H	3.14092200	-4.26700000	-0.24818100
C	0.57154800	-3.35977300	-0.80465800
H	0.68949100	-4.43038300	-0.99897300
C	3.54136100	0.31136700	0.36745300
C	-0.71310800	-2.82777600	-0.86092000
C	-1.94862400	-3.69381800	-1.08881100
N	-1.00130000	-1.49667200	-0.67986900
C	-3.03305000	-2.60569400	-1.35719400
C	-2.37957700	-1.32047700	-0.85049400
C	2.70319500	1.42379100	0.03423700
C	3.19274900	2.85772300	-0.10266400
N	1.41616000	1.28868300	-0.27554800
C	1.85143700	3.66995400	-0.24096000
C	0.86677500	2.57854300	-0.81219200
C	-3.07121800	-0.12095600	-0.72529300
C	-2.36382800	1.11915100	-0.60463900
C	-2.96899400	2.53443100	-0.75616100
N	-1.04028100	1.14735100	-0.42637600
C	-1.66977300	3.37345500	-1.01900300
C	-0.58539900	2.56222000	-0.29850100
Co	0.26894300	-0.13394200	-0.30426200
H	-3.94908400	-2.84340400	-0.78611900
H	-1.47801900	3.30335800	-2.10601300
H	3.69196500	3.17173300	0.83455600
H	-0.59221000	2.79933400	0.78166600
C	0.10154200	0.05691800	2.30075300
C	1.16178200	-0.83327100	2.82331000
H	2.17812000	-0.61944300	2.43874400
O	0.79944400	-0.20528600	4.01033600

H	0.37865600	1.10241600	2.15168200
C	-4.58973300	-0.13955000	-0.76858300
H	-5.01584600	0.63671700	-0.11446900
H	-5.00534100	0.02435700	-1.78001200
H	-4.98219700	-1.10420700	-0.40734900
C	-3.70049500	3.01686100	0.51797800
H	-4.50631500	2.32474800	0.80846800
H	-3.01545400	3.11330100	1.37504200
H	-4.15954200	4.00555900	0.33580700
C	-3.91330400	2.67714400	-1.97088500
H	-4.92600400	2.29177500	-1.77115300
H	-4.01742600	3.74906300	-2.22475400
H	-3.51003500	2.15497800	-2.85740700
C	-1.75621400	4.85330500	-0.63985100
H	-0.86868600	5.40185500	-0.99568500
H	-2.64106900	5.32820200	-1.10228200
H	-1.82377300	4.99868600	0.45245400
C	0.89528500	2.47472300	-2.35275900
H	0.53525200	3.39677900	-2.83815500
H	1.91700300	2.27181800	-2.71034800
H	0.25628800	1.63140400	-2.66937500
C	2.00275800	4.91999300	-1.12330400
H	1.04749900	5.46568700	-1.20534600
H	2.73800200	5.61196900	-0.67274200
H	2.34586200	4.68807000	-2.14425100
C	1.43994400	4.12150300	1.17917000
H	2.21841900	4.78806200	1.59381300
H	0.48965200	4.68313900	1.17636800
H	1.33361500	3.26322400	1.86856700
C	4.23671100	3.01895700	-1.23024200
H	4.57230400	4.06778800	-1.30850900
H	5.12700200	2.40305500	-1.01826900
H	3.84489900	2.70729900	-2.21290100
C	4.90308700	0.59552700	0.97950400
H	5.14947900	-0.13531200	1.76656500
H	5.73047800	0.58165800	0.24681900
H	4.91217000	1.58880900	1.45801900
C	5.41074900	-2.17938000	-0.20133100
H	6.05138500	-1.70869100	0.55978900
H	5.80260300	-3.20055200	-0.36244300
H	5.52506400	-1.61670700	-1.14356000
C	3.88332300	-2.79865700	1.70737800
H	2.84558600	-3.01993200	2.01185100
H	4.47033000	-3.73288000	1.78769800
H	4.30269400	-2.06801900	2.42072700
C	3.53119900	-3.27874000	-2.13954300
H	4.53730100	-3.71397000	-2.27088600
H	2.81609800	-3.89677400	-2.71128900
H	3.52993200	-2.26429200	-2.57888600
C	-3.42018000	-2.43349800	-2.84385400
H	-3.86167600	-3.35910500	-3.25282700
H	-4.16261900	-1.62762300	-2.96441700
H	-2.53695200	-2.17097000	-3.45348800
C	-2.28022300	-4.46651400	0.21182500
H	-3.17983900	-5.09384500	0.06499800
H	-1.44195500	-5.12435600	0.50500600
H	-2.48005900	-3.77117900	1.04173600
C	-1.75759900	-4.71790300	-2.22618200
H	-1.00831800	-5.47735900	-1.93976200
H	-2.70288800	-5.25374700	-2.42693500

H	-1.41734000	-4.24664700	-3.16373200
H	0.96302900	-1.92699700	2.74296200
C	-1.32786400	-0.24255700	2.49084100
C	-2.23687300	0.81912200	2.69662800
C	-1.83264200	-1.56350900	2.51347200
C	-3.59982400	0.57759300	2.91986000
H	-1.85236300	1.84517400	2.69532600
C	-3.19345900	-1.80798200	2.74168200
H	-1.15037300	-2.40128800	2.33472100
C	-4.08774400	-0.73978600	2.94196800
H	-4.28126100	1.41985300	3.08178500
H	-3.56440900	-2.83925700	2.76129500
H	-5.15178800	-0.93442700	3.11716900

$(CH_3)_{15}$(corrin)Co(III)-CH_2-CH(O^-)Ph - Ia

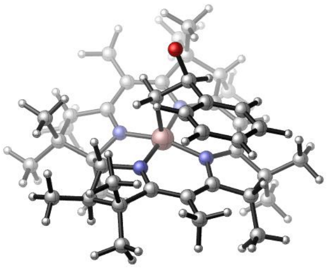

E (BP86-D3/6-31G(d) + SMD (acetone)) = -3313.362102
E (BP86-D3/6-311++G(2df,p)// BP86-D3/6-31G(d) + SMD (acetone)) = -3314.063958
E (wB97XD/6-311++G(2df,p)// BP86-D3/6-31G(d) + SMD (acetone)) = -3313.091918
E (M06-D3/6-311++G(2df,p)// BP86-D3/6-31G(d) + SMD (acetone)) = -3312.155852
E (M06/6-311++G(2df,p)// BP86-D3/6-31G(d) + SMD (acetone)) = -3312.115847
E (PBE0-D3/6-311++G(2df,p)// BP86-D3/6-31G(d) + SMD (acetone)) = -3311.189843
E (B3LYP-D3/6-311++G(2df,p)// BP86-D3/6-31G(d) + SMD (acetone)) = -3313.830875

Zero-point correction=	0.912884
Thermal correction to Energy=	0.960990
Thermal correction to Enthalpy=	0.961935
Thermal correction to Gibbs Free Energy=	0.839526

Charge = 0 Multiplicity = 1

C	2.60941200	-1.95715700	0.21475400
C	2.79440100	-3.43267300	0.62295300
C	1.67352500	-4.11797000	-0.22221600
C	0.70749000	-2.97859500	-0.43925300
N	1.29523100	-1.76358400	-0.24850800
H	1.19934900	-4.93441500	0.35322700
C	-0.59430300	-3.16625300	-0.90077300
H	-0.89780600	-4.19378500	-1.12295700
C	3.53704300	-0.93330300	0.35412200
C	-1.52044800	-2.15547800	-1.13330100
C	-2.84335500	-2.39731700	-1.84380400
N	-1.32316700	-0.83911100	-0.80949100
C	-3.61014000	-1.06843000	-1.54185800
C	-2.51995000	-0.14117700	-1.00433400
C	3.20329300	0.40812000	-0.04324700
C	4.20009600	1.54499600	-0.20360900
N	1.96706200	0.78694200	-0.33186900
C	3.27123200	2.81469600	-0.18957800

C	1.90797300	2.23389200	-0.72983100
C	-2.72316100	1.19517100	-0.67844700
C	-1.60919800	2.05314200	-0.39351700
C	-1.64582600	3.57927800	-0.16969700
N	-0.36869500	1.58501200	-0.30507200
C	-0.15105100	3.94876200	-0.48158400
C	0.59805900	2.68518300	-0.04314500
Co	0.36116900	-0.09956400	-0.29833000
H	-4.30769500	-1.26820000	-0.70314200
H	-0.07934600	4.03011400	-1.58339100
H	4.86011400	1.59610900	0.68197300
H	0.76708300	2.70899400	1.04851800
C	0.36770900	-0.13613500	1.73333700
C	-0.55557000	-1.15531600	2.42521700
H	-0.45385500	-2.14537700	1.88842300
O	-0.06388300	-1.12097000	3.69335100
C	-2.04434000	-0.78164100	2.36798700
C	-3.01608900	-1.71259400	1.95707800
C	-2.48750100	0.47313700	2.83920200
C	-4.38838200	-1.40243000	1.98990500
H	-2.68473900	-2.69311500	1.59045600
C	-3.85133300	0.80019800	2.85973600
H	-1.74676100	1.19659700	3.19922300
C	-4.81234200	-0.13882400	2.43418600
H	-5.12547800	-2.14706600	1.66393500
H	-4.17123400	1.78910800	3.21082800
H	-5.87919500	0.11323000	2.45424400
H	0.16038700	0.88663600	2.09925600
H	1.41207900	-0.39817000	1.97636800
C	-4.13750500	1.74162800	-0.60230300
H	-4.22411900	2.47718500	0.21228100
H	-4.47321600	2.23851500	-1.53076500
H	-4.85388500	0.93898300	-0.36644800
C	-2.00650200	3.85934500	1.31083500
H	-3.00117600	3.45237200	1.55854000
H	-1.27805900	3.39977400	2.00219500
H	-2.02976900	4.94716400	1.50309400
C	-2.57274600	4.37316000	-1.11169700
H	-3.62967100	4.33851800	-0.80460600
H	-2.26966900	5.43657700	-1.10647100
H	-2.49845300	4.00540400	-2.15089100
C	0.34177700	5.25616800	0.14125600
H	1.34941900	5.51127400	-0.22781800
H	-0.32638800	6.09666700	-0.12119000
H	0.39267900	5.19337900	1.24220400
C	1.76482600	2.30365200	-2.26364500
H	1.73397200	3.34608100	-2.62093800
H	2.60335200	1.79153400	-2.76095900
H	0.83141500	1.79919700	-2.56937900
C	3.83146300	3.98267100	-1.01578500
H	3.14428800	4.84673100	-0.99451400
H	4.79577400	4.31619500	-0.59031800
H	4.00617600	3.71773700	-2.07110900
C	3.15747700	3.26096800	1.28711700
H	4.15708900	3.55065100	1.65966100
H	2.49289700	4.13226000	1.40865900
H	2.78280000	2.44609600	1.93407400
C	5.11687600	1.33034700	-1.42704700
H	5.83706700	2.16086700	-1.52755300
H	5.69631300	0.39816700	-1.30475300

H	4.54800600	1.24801600	-2.36828900
C	4.92324800	-1.17967000	0.92544000
H	4.88068600	-1.80991500	1.82815400
H	5.60226600	-1.67306400	0.20687200
H	5.40062200	-0.23422700	1.22540100
C	4.16623200	-4.09062900	0.37748200
H	4.91469900	-3.78920500	1.12506900
H	4.05533000	-5.18736400	0.46030300
H	4.56783100	-3.86390600	-0.62456000
C	2.44112300	-3.52923700	2.13430000
H	1.46712400	-3.06504500	2.37200900
H	2.41321300	-4.59029800	2.44614700
H	3.20647200	-3.01526600	2.74275000
C	2.11971200	-4.67488400	-1.59405300
H	2.81710200	-5.52280400	-1.48337500
H	1.23979600	-5.03283100	-2.15740600
H	2.61339500	-3.89158600	-2.19765400
C	-4.43435200	-0.54065600	-2.73045200
H	-5.06250000	-1.35377800	-3.13675600
H	-5.11275900	0.27397600	-2.43826900
H	-3.78746500	-0.16689500	-3.54271100
C	-3.62250400	-3.62262100	-1.33291600
H	-4.61117500	-3.66453400	-1.82687400
H	-3.09724300	-4.56762200	-1.56059700
H	-3.78511500	-3.56957800	-0.24249900
C	-2.50942700	-2.59395600	-3.34728000
H	-1.86450900	-3.48253100	-3.46827700
H	-3.42366400	-2.75428300	-3.94556200
H	-1.96902300	-1.72348100	-3.76034900

$(CH_3)_{15}$(corrin)Co(III)-CHPh-$CH_2(O^-)$ - Ib

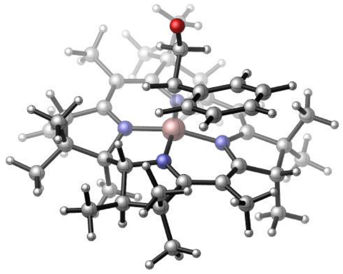

E (BP86-D3/6-31G(d) + SMD (acetone)) = -3313.353368
E (BP86-D3/6-311++G(2df,p)// BP86-D3/6-31G(d) + SMD (acetone)) = -3314.056514
E (wB97XD/6-311++G(2df,p)// BP86-D3/6-31G(d) + SMD (acetone)) = -3313.081588
E (M06-D3/6-311++G(2df,p)// BP86-D3/6-31G(d) + SMD (acetone)) = -3312.147473
E (M06/6-311++G(2df,p)// BP86-D3/6-31G(d) + SMD (acetone)) = -3312.107111
E (PBE0-D3/6-311++G(2df,p)// BP86-D3/6-31G(d) + SMD (acetone)) = -3311.180201
E (B3LYP-D3/6-311++G(2df,p)// BP86-D3/6-31G(d) + SMD (acetone)) = -3313.822528

Zero-point correction=	0.913456
Thermal correction to Energy=	0.961535
Thermal correction to Enthalpy=	0.962479
Thermal correction to Gibbs Free Energy=	0.840491

Charge = 0 Multiplicity = 1

C	3.10634600	-0.96790400	0.15658900

C	3.95554200	-2.25702800	0.21719900
C	3.11331200	-3.19480900	-0.70379000
C	1.73392000	-2.59941700	-0.56111200
N	1.78261500	-1.27911900	-0.21035000
H	3.14232500	-4.23579600	-0.33292800
C	0.57198100	-3.31505200	-0.82731300
H	0.69093900	-4.37815000	-1.05595000
C	3.55477500	0.31817700	0.42457500
C	-0.71344800	-2.78282700	-0.87091200
C	-1.94310300	-3.63755100	-1.16490500
N	-1.00694600	-1.46311300	-0.64849700
C	-3.02133800	-2.53853100	-1.41515500
C	-2.37713500	-1.27336600	-0.85101100
C	2.71624300	1.43705200	0.10936600
C	3.21448200	2.86786400	-0.03301200
N	1.42875200	1.31709000	-0.18200300
C	1.87688500	3.68848400	-0.17168500
C	0.88539900	2.60039600	-0.73614900
C	-3.06498200	-0.07460700	-0.71094600
C	-2.35267500	1.16351200	-0.56711400
C	-2.94897000	2.58237900	-0.72227200
N	-1.03895100	1.19144900	-0.36194400
C	-1.64220600	3.42021700	-0.95564800
C	-0.56950900	2.59865300	-0.22849200
Co	0.26020000	-0.11331000	-0.16987900
H	-3.94744000	-2.79250100	-0.86863700
H	-1.43468600	3.36328100	-2.04034200
H	3.71387200	3.17418800	0.90636800
H	-0.57706300	2.83032000	0.85252000
C	0.05067800	-0.11725400	1.93924900
C	1.02339000	-1.06165900	2.67244300
H	2.07103900	-0.82250400	2.35195900
O	0.72430000	-0.79251500	3.96234400
H	0.33151900	0.92829600	2.15931000
C	-4.58194700	-0.08596500	-0.77551300
H	-5.01190300	0.67889900	-0.11098900
H	-4.98270400	0.09967300	-1.78874500
H	-4.98099400	-1.05609700	-0.43773000
C	-3.69877000	3.05251100	0.54633400
H	-4.50924100	2.35901100	0.81909100
H	-3.02726800	3.14245600	1.41484900
H	-4.15371500	4.04250100	0.36274300
C	-3.87286100	2.73588500	-1.95059400
H	-4.89047800	2.35547900	-1.76809300
H	-3.96610500	3.80987000	-2.19886000
H	-3.45843200	2.21706800	-2.83385900
C	-1.72962000	4.89521000	-0.55846300
H	-0.83348400	5.44414000	-0.89132700
H	-2.60409600	5.37888400	-1.03104200
H	-1.81608600	5.02606300	0.53415600
C	0.92091400	2.47687500	-2.27550400
H	0.57270600	3.39740600	-2.77177500
H	1.94265200	2.26119700	-2.62493600
H	0.27414900	1.63867300	-2.59054300
C	2.03195500	4.93256200	-1.06077400
H	1.07702300	5.47800400	-1.14708800
H	2.76711700	5.62580300	-0.61242400
H	2.37646900	4.69400100	-2.07970200
C	1.46729400	4.14485000	1.24730300
H	2.25193300	4.80316800	1.66306200

H	0.52325600	4.71684900	1.24076400
H	1.34906400	3.28760900	1.93608700
C	4.26114000	3.02133800	-1.15846400
H	4.59814900	4.06935700	-1.23678200
H	5.14974600	2.40471000	-0.94286000
H	3.87080700	2.70935700	-2.14147500
C	4.91486400	0.58813900	1.04355000
H	5.16071700	-0.16598100	1.80829700
H	5.74223400	0.59859700	0.31121200
H	4.91908100	1.56666800	1.55164900
C	5.41613000	-2.13770300	-0.25918700
H	6.06699900	-1.69042600	0.50722100
H	5.81111800	-3.14949400	-0.46413900
H	5.50819200	-1.54046100	-1.18231300
C	3.93049200	-2.83916300	1.65510800
H	2.90036100	-3.07464700	1.97365600
H	4.52354000	-3.77240400	1.68409900
H	4.36312500	-2.13370200	2.38515600
C	3.50820000	-3.18984400	-2.19856400
H	4.51158800	-3.62194300	-2.35509800
H	2.78452500	-3.79031500	-2.77792800
H	3.50244600	-2.16254700	-2.60642500
C	-3.37948000	-2.31181900	-2.90185200
H	-3.81378400	-3.22217400	-3.34997200
H	-4.11920200	-1.50174500	-3.00784100
H	-2.48463800	-2.02982100	-3.48503000
C	-2.30906600	-4.46606900	0.09178800
H	-3.18623600	-5.10490600	-0.12284200
H	-1.46981700	-5.11801700	0.39441100
H	-2.56329400	-3.81016300	0.93875400
C	-1.71901600	-4.61083500	-2.34051000
H	-0.98437500	-5.38748900	-2.06350100
H	-2.66062500	-5.12976000	-2.59397900
H	-1.34653800	-4.10039600	-3.24462700
H	0.85580700	-2.12858600	2.31865600
C	-1.37974000	-0.35069500	2.30543700
C	-2.25167900	0.72936200	2.55920000
C	-1.92026700	-1.65500300	2.40043200
C	-3.60430000	0.52855700	2.88162500
H	-1.84727100	1.74696100	2.51241300
C	-3.26765600	-1.86605900	2.71816600
H	-1.26362200	-2.51156000	2.21486500
C	-4.12479100	-0.77320100	2.95649300
H	-4.25016800	1.39225300	3.07709000
H	-3.65632900	-2.88954100	2.78727100
H	-5.17930100	-0.93825400	3.20617800

NH_3

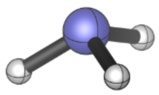

E (BP86-D3/6-31G(d) + SMD (acetone)) = -56.553288
E (BP86-D3/6-311++G(2df,p)// BP86-D3/6-31G(d) + SMD (acetone)) = -56.58880
E (wB97XD/6-311++G(2df,p)// BP86-D3/6-31G(d) + SMD (acetone)) = -56.567956
E (M06-D3/6-311++G(2df,p)// BP86-D3/6-31G(d) + SMD (acetone)) = -56.544989
E (M06/6-311++G(2df,p)// BP86-D3/6-31G(d) + SMD (acetone)) = -56.544988

E (PBE0-D3/6-311++G(2df,p)// BP86-D3/6-31G(d) + SMD (acetone)) = -56.516998
E (B3LYP-D3/6-311++G(2df,p)// BP86-D3/6-31G(d) + SMD (acetone)) = -56.58989

Zero-point correction=	0.033453
Thermal correction to Energy=	0.036310
Thermal correction to Enthalpy=	0.037255
Thermal correction to Gibbs Free Energy=	0.015381

Charge = 0 Multiplicity = 1

N	0.00000000	0.00000000	0.12792900
H	0.00000000	0.93828000	-0.29850200
H	-0.81257500	-0.46914000	-0.29850200
H	0.81257500	-0.46914000	-0.29850200

NH_4^+

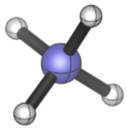

E (BP86-D3/6-31G(d) + SMD (acetone)) = -57.021003
E (BP86-D3/6-311++G(2df,p)// BP86-D3/6-31G(d) + SMD (acetone)) = -57.047387
E (wB97XD/6-311++G(2df,p)// BP86-D3/6-31G(d) + SMD (acetone)) = -57.031286
E (M06-D3/6-311++G(2df,p)// BP86-D3/6-31G(d) + SMD (acetone)) = -57.001833
E (M06/6-311++G(2df,p)// BP86-D3/6-31G(d) + SMD (acetone)) = -57.001831
E (PBE0-D3/6-311++G(2df,p)// BP86-D3/6-31G(d) + SMD (acetone)) = -56.978602
E (B3LYP-D3/6-311++G(2df,p)// BP86-D3/6-31G(d) + SMD (acetone)) = -57.049074

Zero-point correction=	0.048305
Thermal correction to Energy=	0.051165
Thermal correction to Enthalpy=	0.052109
Thermal correction to Gibbs Free Energy=	0.030973

Charge = 1 Multiplicity = 1

N	0.00000000	0.00000000	0.00000000
H	0.59708800	0.59708800	0.59708800
H	-0.59708800	-0.59708800	0.59708800
H	-0.59708800	0.59708800	-0.59708800
H	0.59708800	-0.59708800	-0.59708800

$(CH_3)_{15}$(corrin)Co(III)-CH_2-CH(OH)Ph - IIa

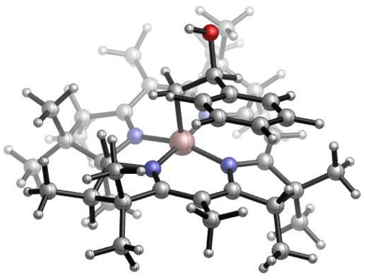

E (BP86-D3/6-31G(d) + SMD (acetone)) = -3313.879274
E (BP86-D3/6-311++G(2df,p)// BP86-D3/6-31G(d) + SMD (acetone)) = -3314.571074
E (wB97XD/6-311++G(2df,p)// BP86-D3/6-31G(d) + SMD (acetone)) = -3313.619455
E (M06-D3/6-311++G(2df,p)// BP86-D3/6-31G(d) + SMD (acetone)) = -3312.67077

E (M06/6-311++G(2df,p)// BP86-D3/6-31G(d) + SMD (acetone)) = -3312.6303
E (PBE0-D3/6-311++G(2df,p)// BP86-D3/6-31G(d) + SMD (acetone)) = -3311.712196
E (B3LYP-D3/6-311++G(2df,p)// BP86-D3/6-31G(d) + SMD (acetone)) = -3314.352068

Zero-point correction=	0.927117
Thermal correction to Energy=	0.975645
Thermal correction to Enthalpy=	0.976589
Thermal correction to Gibbs Free Energy=	0.853187

Charge = 1 Multiplicity = 1

C	2.62964900	-1.94135100	0.21759200
C	2.84535500	-3.43469000	0.53143600
C	1.74738500	-4.08554800	-0.36807000
C	0.74818100	-2.96290700	-0.49070200
N	1.31613700	-1.74729400	-0.25039900
H	1.29945300	-4.96181900	0.13465200
C	-0.56737500	-3.15852900	-0.90222100
H	-0.86229400	-4.18519800	-1.13738200
C	3.53595800	-0.91081700	0.41815300
C	-1.51065100	-2.15620000	-1.10635100
C	-2.83832900	-2.41427400	-1.79960300
N	-1.31825200	-0.83634100	-0.79806000
C	-3.60769700	-1.08327100	-1.51757900
C	-2.52100200	-0.14493400	-0.99559300
C	3.19856900	0.43298000	0.02793200
C	4.19365300	1.57182900	-0.11928300
N	1.96983500	0.80758200	-0.28301600
C	3.26173400	2.84018200	-0.13849700
C	1.90966500	2.25097400	-0.69843000
C	-2.72685700	1.19413200	-0.68997600
C	-1.61531200	2.05940300	-0.41397900
C	-1.65885000	3.58904500	-0.21950100
N	-0.37520000	1.59975900	-0.30663100
C	-0.16053500	3.95827000	-0.51514700
C	0.58633800	2.70582600	-0.04217800
Co	0.36036300	-0.09060300	-0.26761100
H	-4.30280200	-1.26981400	-0.67417000
H	-0.07138700	4.01953400	-1.61658900
H	4.83305500	1.62897000	0.78061300
H	0.73305400	2.74596400	1.05255800
C	0.35727900	-0.13023600	1.69730900
C	-0.59948400	-1.15777100	2.28865100
H	-0.48518000	-2.11864000	1.75950800
O	-0.17401800	-1.46133400	3.65076200
C	-2.07123700	-0.77248900	2.28651700
C	-3.04501700	-1.74347400	1.98555300
C	-2.49840200	0.51676600	2.66467900
C	-4.41376500	-1.43702000	2.04490900
H	-2.71912300	-2.74711400	1.68717200
C	-3.86600200	0.82704600	2.73472000
H	-1.76128500	1.29270700	2.89990000
C	-4.82912700	-0.14765500	2.42121100
H	-5.15557900	-2.20408700	1.79424300
H	-4.17969300	1.83621700	3.02481700
H	-5.89629900	0.09723600	2.46623000
H	0.14056800	0.88688700	2.06925900
H	1.38542500	-0.40205500	1.99361500
C	-4.14165600	1.73778500	-0.62381300
H	-4.22912500	2.48963400	0.17491100
H	-4.47466000	2.21355900	-1.56344400

H	-4.85502300	0.93775900	-0.37191400
C	-2.04132800	3.89436800	1.25089700
H	-3.03573100	3.48568400	1.49543400
H	-1.31659200	3.45898400	1.96156500
H	-2.07561000	4.98577300	1.41645900
C	-2.57564900	4.35833500	-1.19098300
H	-3.63580900	4.32900600	-0.89572000
H	-2.27305600	5.42141900	-1.20514600
H	-2.48708800	3.96736200	-2.22028500
C	0.31746200	5.27795700	0.09265600
H	1.32868500	5.53142100	-0.26661400
H	-0.35107200	6.10948100	-0.19488700
H	0.35224400	5.23446400	1.19500500
C	1.79588500	2.29722400	-2.23539800
H	1.77141700	3.33487100	-2.60578200
H	2.64497400	1.77995400	-2.70809400
H	0.86915500	1.78868800	-2.55456500
C	3.83625200	3.99700000	-0.97021400
H	3.14903500	4.86076100	-0.97208600
H	4.79236000	4.33525500	-0.53100200
H	4.02998800	3.71799100	-2.01834400
C	3.11717500	3.30591300	1.32912000
H	4.10844600	3.60320100	1.71672800
H	2.44843100	4.17733800	1.42384800
H	2.73213700	2.49947400	1.98076500
C	5.13725600	1.34608700	-1.32049200
H	5.85524700	2.17908000	-1.41043200
H	5.71752500	0.41810700	-1.17618500
H	4.58969700	1.25404100	-2.27316700
C	4.90426000	-1.15008400	1.03052100
H	4.84791000	-1.85152100	1.87721300
H	5.63203600	-1.55810300	0.30638700
H	5.32697000	-0.21313600	1.42498800
C	4.23696700	-4.03398100	0.25463300
H	4.96559500	-3.77824900	1.03800800
H	4.15250600	-5.13576900	0.24325700
H	4.64543700	-3.71246800	-0.71803700
C	2.46856500	-3.65059800	2.02283900
H	1.44404300	-3.30021900	2.23989900
H	2.52850900	-4.72649300	2.27032300
H	3.15951700	-3.10460200	2.68865600
C	2.20903100	-4.50375600	-1.78328500
H	2.93373800	-5.33425500	-1.74120400
H	1.34059500	-4.84005500	-2.37665600
H	2.67776100	-3.65516300	-2.31361800
C	-4.43349400	-0.57554200	-2.71397300
H	-5.05791000	-1.39806800	-3.10564600
H	-5.11495000	0.24015600	-2.43369400
H	-3.78769300	-0.21277500	-3.53163000
C	-3.60802400	-3.63479100	-1.26355100
H	-4.59504700	-3.69070200	-1.75848600
H	-3.07618100	-4.57983500	-1.47393400
H	-3.77301700	-3.56134400	-0.17522500
C	-2.50897100	-2.63544500	-3.30157000
H	-1.86360300	-3.52469600	-3.41165900
H	-3.42781500	-2.80808600	-3.88820300
H	-1.97423100	-1.77048200	-3.73249100
H	-0.27008600	-0.61977900	4.15279800

$(CH_3)_{15}$(corrin)Co(III)-CHPh-CH_2OH - IIb

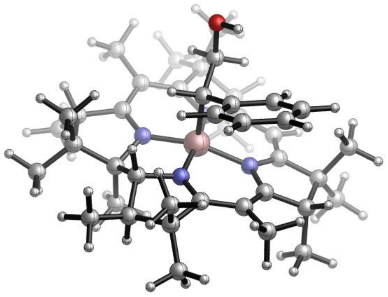

E (BP86-D3/6-31G(d) + SMD (acetone)) = -3313.877792
E (BP86-D3/6-311++G(2df,p)// BP86-D3/6-31G(d) + SMD (acetone)) = -3314.570354
E (wB97XD/6-311++G(2df,p)// BP86-D3/6-31G(d) + SMD (acetone)) = -3313.616803
E (M06-D3/6-311++G(2df,p)// BP86-D3/6-31G(d) + SMD (acetone)) = -3312.668526
E (M06/6-311++G(2df,p)// BP86-D3/6-31G(d) + SMD (acetone)) = -3312.627742
E (PBE0-D3/6-311++G(2df,p)// BP86-D3/6-31G(d) + SMD (acetone)) = -3311.710079
E (B3LYP-D3/6-311++G(2df,p)// BP86-D3/6-31G(d) + SMD (acetone)) = -3314.35043

Zero-point correction=	0.928394
Thermal correction to Energy=	0.975817
Thermal correction to Enthalpy=	0.976761
Thermal correction to Gibbs Free Energy=	0.857064

Charge = 1 Multiplicity = 1

C	3.11167700	-0.94832000	0.14500900
C	3.96513100	-2.23129800	0.20900400
C	3.13266800	-3.17303500	-0.71747900
C	1.75060500	-2.58496000	-0.58320400
N	1.79326700	-1.26358400	-0.23895100
H	3.16205200	-4.21357900	-0.34733200
C	0.59161700	-3.30853300	-0.83932900
H	0.71587500	-4.37225800	-1.06044200
C	3.53696600	0.33526000	0.45161000
C	-0.69653300	-2.78402800	-0.88953700
C	-1.91793400	-3.64647700	-1.18928200
N	-0.99930000	-1.46632500	-0.67464600
C	-3.01534600	-2.55827600	-1.40702000
C	-2.37341100	-1.28781900	-0.85678300
C	2.69880700	1.45331900	0.11500200
C	3.19393500	2.88390400	-0.02610600
N	1.42310900	1.32815500	-0.20794800
C	1.85511700	3.70093200	-0.18147000
C	0.87153700	2.61234700	-0.75960400
C	-3.06829400	-0.09459900	-0.69975900
C	-2.36205800	1.15012400	-0.57551900
C	-2.96724000	2.56533800	-0.72454900
N	-1.04829500	1.19226700	-0.39200600
C	-1.66746000	3.40729000	-0.98747900
C	-0.58536600	2.60083800	-0.25745200
Co	0.26145900	-0.11011400	-0.18572400
H	-3.92151700	-2.82239400	-0.83310200
H	-1.47115300	3.33255400	-2.07310200
H	3.68320700	3.19199200	0.91739000
H	-0.60111500	2.83130800	0.82372400
C	0.01752600	-0.07458400	1.81979700
C	0.99177700	-1.01834000	2.52797800
H	2.02769200	-0.72474500	2.31781800

O	0.85460300	-0.90893700	3.95923600
H	0.28739100	0.96679100	2.07012800
C	-4.58495300	-0.11806200	-0.72872900
H	-5.00427400	0.64326600	-0.05388900
H	-5.00705900	0.06597900	-1.73296600
H	-4.96742800	-1.09238700	-0.38466500
C	-3.68686200	3.03261400	0.56319400
H	-4.47512000	2.32752900	0.86898100
H	-2.99011700	3.14647900	1.40866300
H	-4.16406100	4.01201600	0.38136600
C	-3.91919300	2.70653600	-1.93168200
H	-4.93163300	2.32769800	-1.72069700
H	-4.01800900	3.77834800	-2.18530900
H	-3.52540800	2.18113600	-2.82024500
C	-1.75673800	4.88701200	-0.61095200
H	-0.86758600	5.43361200	-0.96535200
H	-2.64000600	5.35799500	-1.07929900
H	-1.82891600	5.03429600	0.48043700
C	0.91500800	2.49246800	-2.29830100
H	0.55985500	3.41314800	-2.78873500
H	1.93998500	2.28898800	-2.64520100
H	0.27608600	1.65121300	-2.62129600
C	2.01730800	4.94445200	-1.06946300
H	1.06308200	5.48990200	-1.16199800
H	2.74919100	5.63635200	-0.61442900
H	2.36982100	4.70541900	-2.08537800
C	1.42811000	4.15505800	1.23283900
H	2.20555400	4.81680900	1.65583600
H	0.48191200	4.72291200	1.21495400
H	1.30675000	3.29799400	1.92169700
C	4.25038600	3.03124800	-1.14336800
H	4.58069900	4.08064300	-1.22488500
H	5.14063200	2.42213600	-0.91493700
H	3.86912100	2.71015300	-2.12676200
C	4.88482200	0.61384100	1.08999400
H	5.13793900	-0.15313800	1.83820800
H	5.71409200	0.65350400	0.36153300
H	4.86694500	1.58144000	1.61767200
C	5.42738200	-2.09780100	-0.25654800
H	6.06764600	-1.64480100	0.51522900
H	5.83203500	-3.10596900	-0.45857800
H	5.51950500	-1.49937500	-1.17863000
C	3.92996600	-2.81136800	1.64855100
H	2.90107400	-3.06477300	1.95660500
H	4.53533000	-3.73562700	1.68379800
H	4.34470800	-2.10038600	2.38341200
C	3.53335200	-3.16378900	-2.21081400
H	4.53960700	-3.59117700	-2.35830700
H	2.81656400	-3.76860400	-2.79379600
H	3.52532800	-2.13637400	-2.61763300
C	-3.42089600	-2.33150800	-2.88153200
H	-3.85310200	-3.24855700	-3.31673400
H	-4.17769400	-1.53476700	-2.96175800
H	-2.54933300	-2.03319000	-3.49069100
C	-2.25979700	-4.51165400	0.04970800
H	-3.12348800	-5.16206800	-0.18133400
H	-1.40677800	-5.15442400	0.33201100
H	-2.52886500	-3.88559800	0.91477000
C	-1.68358600	-4.58711100	-2.38989000
H	-0.92980400	-5.35312800	-2.13713700

H	-2.61684400	-5.11919200	-2.64489000
H	-1.33196400	-4.04703500	-3.28480100
H	0.85510100	-2.06520800	2.18944700
C	-1.39903600	-0.32862500	2.22047500
C	-2.23892900	0.74573300	2.58383100
C	-1.93213700	-1.63682400	2.30448300
C	-3.56659600	0.53421400	2.98334900
H	-1.83213600	1.76200200	2.54900600
C	-3.25745900	-1.85671700	2.70584600
H	-1.30004600	-2.48789800	2.03015400
C	-4.08588600	-0.77034000	3.04015100
H	-4.19562800	1.39009700	3.25180600
H	-3.64763400	-2.87986900	2.75579500
H	-5.12319900	-0.94052800	3.34928300
H	-0.07045700	-1.17644200	4.16026300

$(CH_3)_{15}$(corrin)Co(II)

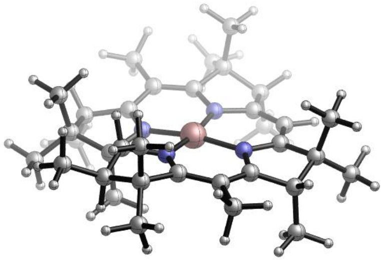

E (BP86-D3/6-31G(d) + SMD (acetone)) = -2928.348782
E (BP86-D3/6-311++G(2df,p)// BP86-D3/6-31G(d) + SMD (acetone)) = -2928.920801
E (wB97XD/6-311++G(2df,p)// BP86-D3/6-31G(d) + SMD (acetone)) = -2928.135257
E (M06-D3/6-311++G(2df,p)// BP86-D3/6-31G(d) + SMD (acetone)) = -2927.342949
E (M06/6-311++G(2df,p)// BP86-D3/6-31G(d) + SMD (acetone)) = -2927.316126
E (PBE0-D3/6-311++G(2df,p)// BP86-D3/6-31G(d) + SMD (acetone)) = -2926.56083
E (B3LYP-D3/6-311++G(2df,p)// BP86-D3/6-31G(d) + SMD (acetone)) = -2928.726932

Zero-point correction=	0.365872
Thermal correction to Energy=	0.385009
Thermal correction to Enthalpy=	0.385953
Thermal correction to Gibbs Free Energy=	0.318753

Charge = 0 Multiplicity = 1

C	-1.04576000	2.77500100	-0.08371400
C	-2.31403100	3.58787200	-0.24874800
C	-3.43292300	2.55671800	-0.01268900
C	-2.69138400	1.23811300	-0.01699200
N	-1.33315300	1.40921800	-0.01248900
H	-2.35333100	4.00502300	-1.27222600
H	-4.22628700	2.57687900	-0.77884500
C	-3.33201600	0.00011700	0.00016900
H	-4.42623800	0.00015300	0.00027900
C	0.21724400	3.32453300	-0.03381900
H	0.31604700	4.41141000	-0.10211700
C	-2.69146800	-1.23792900	0.01720600
C	-3.43314800	-2.55645600	0.01310800
N	-1.33325400	-1.40914000	0.01245400

C	-2.31425200	-3.58781700	0.24826600
H	-4.22596700	-2.57663600	0.77983500
C	-1.04594500	-2.77494600	0.08347400
H	-2.35335400	-4.00574600	1.27142600
C	1.39269200	2.54047100	0.10500500
C	2.81064700	3.05983100	0.24160300
N	1.35051800	1.21856300	0.16189900
C	3.65766400	1.79254200	-0.03678300
H	2.96384200	3.43676000	1.27244000
C	2.71229400	0.65575600	0.38739100
H	4.61302700	1.77719600	0.51064900
C	0.21703400	-3.32454300	0.03358700
H	0.31576200	-4.41143600	0.10176400
C	1.39254100	-2.54054900	-0.10508000
C	2.81048200	-3.05999400	-0.24151900
N	1.35045600	-1.21863600	-0.16195200
C	3.65754000	-1.79276400	0.03700700
H	2.96378300	-3.43690300	-1.27234800
C	2.71229700	-0.65591500	-0.38727500
H	4.61297900	-1.77746600	-0.51029400
Co	-0.03616400	0.00000100	-0.00004600
H	-2.34146400	-4.43918400	-0.45187000
H	-3.92719900	-2.69661200	-0.96700900
H	-3.92622700	2.69712100	0.96777600
H	-2.34105000	4.43975400	0.45076000
H	3.01544400	-3.89714400	0.44706000
H	3.87130100	-1.71107200	1.11865400
H	3.87156900	1.71081600	-1.11839900
H	3.01574600	3.89695000	-0.44697300
H	2.80958900	-0.43021000	-1.46913500
H	2.80943900	0.43004700	1.46926300

2-hydroxy-2-phenyl-ethyl radical - IIIa

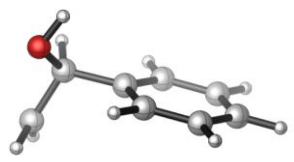

E (BP86-D3/6-31G(d) + SMD (acetone)) = -385.430636
E (BP86-D3/6-311++G(2df,p)// BP86-D3/6-31G(d) + SMD (acetone)) = -385.564351
E (wB97XD/6-311++G(2df,p)// BP86-D3/6-31G(d) + SMD (acetone)) = -385.420779
E (M06-D3/6-311++G(2df,p)// BP86-D3/6-31G(d) + SMD (acetone)) = -385.266213
E (M06/6-311++G(2df,p)// BP86-D3/6-31G(d) + SMD (acetone)) = -385.264301
E (PBE0-D3/6-311++G(2df,p)// BP86-D3/6-31G(d) + SMD (acetone)) = -385.105271
E (B3LYP-D3/6-311++G(2df,p)// BP86-D3/6-31G(d) + SMD (acetone)) = -385.573588

Zero-point correction=	0.142268
Thermal correction to Energy=	0.151030
Thermal correction to Enthalpy=	0.151974
Thermal correction to Gibbs Free Energy=	0.108138

Charge = 0 Multiplicity = 2

C	-2.23005100	-0.60482000	1.16092700
C	-1.72567800	-0.36823400	-0.22392600

H	-1.91888800	-1.26567700	-0.85363600
O	-2.40456200	0.78681900	-0.75075400
C	-0.20369900	-0.16363700	-0.14353100
C	0.67707700	-1.24905800	-0.32525600
C	0.32461900	1.10746900	0.16587300
C	2.06402500	-1.06747800	-0.20081900
H	0.27086200	-2.23876000	-0.56959100
C	1.71093500	1.28960300	0.28487400
H	-0.36329100	1.95021600	0.29728100
C	2.58550600	0.20239300	0.10467600
H	2.74010200	-1.91733000	-0.35093800
H	2.11121700	2.28329500	0.51816200
H	3.66814300	0.34526500	0.19759200
H	-2.39645400	0.25840800	1.81462800
H	-2.14508400	-1.60008300	1.60701500
H	-1.94651400	1.01268800	-1.59138600

2-hydroxy-1-phenyl-ethyl radical - IIIb

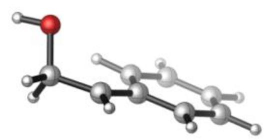

E (BP86-D3/6-31G(d) + SMD (acetone)) = -385.450209
E (BP86-D3/6-311++G(2df,p)// BP86-D3/6-31G(d) + SMD (acetone)) = -385.584219
E (wB97XD/6-311++G(2df,p)// BP86-D3/6-31G(d) + SMD (acetone)) = -385.439457
E (M06-D3/6-311++G(2df,p)// BP86-D3/6-31G(d) + SMD (acetone)) = -385.286187
E (M06/6-311++G(2df,p)// BP86-D3/6-31G(d) + SMD (acetone)) = -385.284479
E (PBE0-D3/6-311++G(2df,p)// BP86-D3/6-31G(d) + SMD (acetone)) = -385.1239
E (B3LYP-D3/6-311++G(2df,p)// BP86-D3/6-31G(d) + SMD (acetone)) = -385.593337

Zero-point correction=	0.143843
Thermal correction to Energy=	0.152401
Thermal correction to Enthalpy=	0.153345
Thermal correction to Gibbs Free Energy=	0.109788

Charge = 0 Multiplicity = 2

C	-1.32076300	1.04368600	0.13564300
C	-2.57326800	0.26091200	0.38926200
O	-2.76024300	-0.73707800	-0.64816600
H	-1.40877900	2.13286700	0.04157300
H	-2.50585700	-0.23731400	1.38451000
C	-0.01828600	0.47738900	0.06493500
C	1.12451500	1.32510200	-0.12133200
C	0.22126100	-0.93234600	0.19225000
C	2.41640500	0.79966500	-0.16615800
H	0.96736700	2.40594500	-0.22429900
C	1.51903300	-1.44502400	0.14891400
H	-0.63525600	-1.60510200	0.29730000
C	2.62697900	-0.58908600	-0.02857100
H	3.27153600	1.47116600	-0.30689600
H	1.67697000	-2.52551100	0.24886000
H	3.64209900	-0.99975800	-0.06397900
H	-3.44048000	0.95230400	0.43295300

H	-3.46092300	-1.33976500	-0.31434900

Propylene oxide

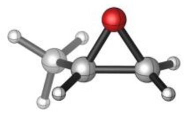

E (BP86-D3/6-31G(d) + SMD (acetone)) = -193.116591
E (BP86-D3/6-311++G(2df,p)// BP86-D3/6-31G(d) + SMD (acetone)) = -193.186628

Zero-point correction=	0.083131
Thermal correction to Energy=	0.087595
Thermal correction to Enthalpy=	0.088539
Thermal correction to Gibbs Free Energy=	0.056727

Charge = 0 Multiplicity = 1

C	-1.04194000	0.62571000	-0.06391300
C	0.15610700	-0.03405300	0.49377000
C	1.51540900	0.09572200	-0.15239600
H	0.16153700	-0.24285800	1.57660700
O	-0.84014200	-0.80137600	-0.23956200
H	2.09476300	0.90418400	0.33121200
H	2.09058600	-0.84271300	-0.04722800
H	-1.87544500	0.90924500	0.59769200
H	-0.94595700	1.23152200	-0.97878000
H	1.41819500	0.32735800	-1.22777400

Cyclohexene oxide (5o)

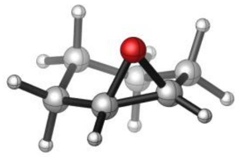

E (BP86-D3/6-31G(d) + SMD (acetone)) = -309.867094
E (BP86-D3/6-311++G(2df,p)// BP86-D3/6-31G(d) + SMD (acetone)) = -309.968367

Zero-point correction=	0.147541
Thermal correction to Energy=	0.153648
Thermal correction to Enthalpy=	0.154592
Thermal correction to Gibbs Free Energy=	0.117916

Charge = 0 Multiplicity = 1

C	0.98720600	0.84213000	-0.30180800
C	1.09675000	-0.63335600	-0.42211500
C	-0.35046800	1.52228900	-0.02911000
C	-0.13082900	-1.51657600	-0.29172400
H	1.92233100	-1.04800600	-1.02479000
O	1.53195800	0.03967700	0.79048300
C	-1.55708600	0.57047200	-0.17687400
H	-0.46002900	2.38496800	-0.71272100
H	-0.31378400	1.93646200	0.99770400
C	-1.28127000	-0.80788200	0.44934800

H	-0.45628400	-1.79308800	-1.31519300
H	0.14931300	-2.45882300	0.21863200
H	-1.78004300	0.43006200	-1.25403100
H	-2.45393500	1.03393100	0.27491100
H	-2.18839500	-1.43902200	0.41403600
H	-1.01084100	-0.68485000	1.51563800
H	1.75019000	1.45848900	-0.80435500

***cis*-1-phenylpropylene oxide**

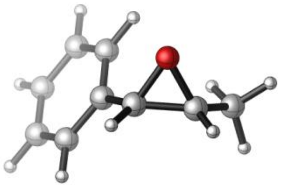

E (BP86-D3/6-31G(d) + SMD (acetone)) = -424.187226
E (BP86-D3/6-311++G(2df,p)// BP86-D3/6-31G(d) + SMD (acetone)) = -424.322652

Zero-point correction=	0.162298
Thermal correction to Energy=	0.171145
Thermal correction to Enthalpy=	0.172089
Thermal correction to Gibbs Free Energy=	0.128320

Charge = 0 Multiplicity = 1

C	2.40649000	0.29792400	0.23858800
C	1.23298000	0.83124600	-0.51606500
H	1.32445000	1.86349400	-0.89299200
O	2.15040700	-0.10866300	-1.13430800
C	-0.17545500	0.38453500	-0.28213900
C	-1.12375500	1.31641000	0.19037400
C	-0.58054400	-0.94390300	-0.52419300
C	-2.44764200	0.92110700	0.43681800
H	-0.81933900	2.35560900	0.36671200
C	-1.90654300	-1.33758000	-0.28139200
H	0.15308000	-1.65613500	-0.91581200
C	-2.84325300	-0.40832200	0.20316100
H	-3.17363300	1.65376000	0.80780600
H	-2.20971300	-2.37282800	-0.47632700
H	-3.87821800	-0.71566300	0.39147000
C	2.31128700	-0.75660100	1.31314900
H	1.38642900	-1.35052200	1.22872400
H	3.17540600	-1.44309700	1.25234900
H	2.33042700	-0.27608300	2.30869800
H	3.26647200	0.98187700	0.33402700

***trans*-1-phenylpropylene oxide**

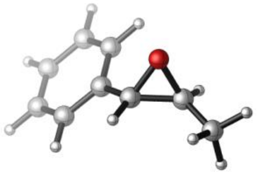

E (BP86-D3/6-31G(d) + SMD (acetone)) = -424.188449
E (BP86-D3/6-311++G(2df,p)// BP86-D3/6-31G(d) + SMD (acetone)) = -424.324107

Zero-point correction=	0.162156
Thermal correction to Energy=	0.171092
Thermal correction to Enthalpy=	0.172036
Thermal correction to Gibbs Free Energy=	0.127970

Charge = 0 Multiplicity = 1

C	2.18947200	-0.23741400	0.40991800
C	1.15634400	0.39687200	-0.45341800
H	1.42933500	1.35009700	-0.93414100
O	1.92408800	-0.74852400	-0.92402400
C	-0.30620700	0.19571500	-0.22276500
C	-1.16838700	1.31026500	-0.17300500
C	-0.84011900	-1.09587500	-0.03151500
C	-2.53893500	1.13826900	0.08110200
H	-0.76122000	2.31645000	-0.33262900
C	-2.20999600	-1.26715600	0.21815700
H	-0.17151900	-1.96140900	-0.09827200
C	-3.06389800	-0.15070700	0.27861200
H	-3.19875000	2.01278100	0.11830500
H	-2.61439800	-2.27596400	0.36136700
H	-4.13413900	-0.28567900	0.47222300
C	3.54280100	0.39023700	0.63967000
H	3.81605500	1.05576400	-0.19761400
H	3.53660700	0.98067700	1.57425100
H	4.32054500	-0.38965300	0.73558100
H	1.81834400	-0.89611300	1.21257900

$(CH_3)_{15}$(corrin)Co(I) - Propylene oxide – TS2a

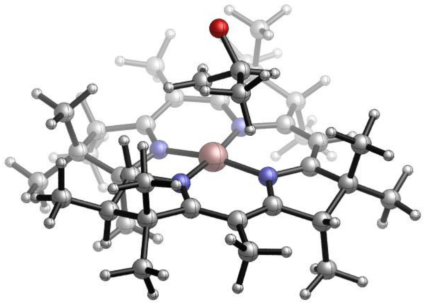

E (BP86-D3/6-31G(d) + SMD (acetone)) = -3121.599058
E (BP86-D3/6-311++G(2df,p)// BP86-D3/6-31G(d) + SMD (acetone)) = -3122.245755

Zero-point correction=	0.860764
Thermal correction to Energy=	0.905782
Thermal correction to Enthalpy=	0.906727
Thermal correction to Gibbs Free Energy=	0.790568

Charge = 0 Multiplicity = 1

C	-3.01576600	-0.48713100	-0.03235100
C	-4.33582400	0.25550100	0.26985500
C	-4.05912600	1.62640400	-0.41519500
C	-2.55448600	1.69600700	-0.33624400
N	-1.98649500	0.45259600	-0.24791300
H	-4.53224600	2.44837700	0.15395600
C	-1.85096300	2.89511300	-0.38755700
H	-2.44208500	3.81496400	-0.43050700
C	-2.81888800	-1.86220700	0.02063500
C	-0.46623600	3.01030300	-0.39212200
C	0.26505200	4.34617800	-0.35667600
N	0.39541200	1.93969200	-0.35411600
C	1.66905600	3.91102100	-0.85986100

C	1.70296100	2.43164300	-0.48677800
C	-1.52781500	-2.42058200	-0.25425300
C	-1.24511500	-3.90137500	-0.45734700
N	-0.43364300	-1.67233000	-0.37485800
C	0.31565700	-3.97259200	-0.28457600
C	0.75809600	-2.52186300	-0.71024900
C	2.87146100	1.68928700	-0.36717800
C	2.81419500	0.27114900	-0.16407100
C	4.00309700	-0.67516200	0.09680200
N	1.64626500	-0.37540500	-0.10975000
C	3.35010300	-2.06281700	-0.22612800
C	1.87158600	-1.83183300	0.10734600
Co	-0.11439400	0.11779700	-0.21318100
H	2.44752200	4.48895200	-0.33231300
H	3.44401400	-2.19657800	-1.32052600
H	-1.70377400	-4.48907500	0.36012200
H	1.69692200	-2.02878600	1.18009000
C	-0.43240700	-0.20621700	2.18216800
C	-0.14746300	1.07338800	2.86830200
H	-0.77263000	1.92907200	2.51638500
O	-0.66628200	0.32157600	3.92150600
H	0.29523800	-1.01297400	2.30262700
H	-1.46485800	-0.49074300	1.98249000
C	4.21591800	2.38838600	-0.47338000
H	4.89627700	2.08017200	0.33844100
H	4.72942500	2.17065200	-1.42767900
H	4.11559000	3.48152100	-0.40242000
C	4.39541000	-0.55081400	1.59101600
H	4.68974700	0.48565800	1.83188200
H	3.55425600	-0.82265000	2.25343400
H	5.25105100	-1.20849200	1.82974100
C	5.24687100	-0.49404300	-0.79847300
H	5.89716600	0.33337900	-0.47476900
H	5.85578200	-1.41660000	-0.75729900
H	4.96285300	-0.32499700	-1.85265700
C	3.99950400	-3.26222700	0.46917000
H	3.58741900	-4.21214300	0.08926400
H	5.08971800	-3.28252300	0.28719700
H	3.83828300	-3.23975200	1.56137300
C	1.03187900	-2.37810400	-2.22225300
H	1.88149200	-3.00005900	-2.54887800
H	0.14606100	-2.66788900	-2.80878500
H	1.25623200	-1.32226300	-2.45304300
C	0.96757900	-5.09710200	-1.10448700
H	2.06488200	-5.09080500	-0.98126000
H	0.60056300	-6.08034000	-0.75670100
H	0.75121700	-5.02285900	-2.18246200
C	0.58184700	-4.23598600	1.21651200
H	0.15057100	-5.21389600	1.49918800
H	1.65865800	-4.26907700	1.45133600
H	0.11124700	-3.46632100	1.85604200
C	-1.85101700	-4.43645000	-1.77305300
H	-1.64105000	-5.51384700	-1.89375600
H	-2.94797500	-4.30990800	-1.76205100
H	-1.46546700	-3.90739500	-2.66047600
C	-3.94694900	-2.81605700	0.38030200
H	-4.52904000	-2.44798200	1.24068400
H	-4.65576500	-2.98071400	-0.45146900
H	-3.55372100	-3.80308200	0.66916300
C	-5.65569000	-0.36721000	-0.22427600

H	-6.00188000	-1.18860600	0.42071900
H	-6.44442600	0.40761000	-0.20702600
H	-5.57729400	-0.74902400	-1.25638700
C	-4.40076400	0.46958000	1.80712500
H	-3.50178700	0.99629300	2.17671800
H	-5.28910100	1.07376400	2.07128900
H	-4.47051400	-0.49559700	2.33916500
C	-4.49960500	1.72882900	-1.89346200
H	-5.59794400	1.69193400	-1.99876600
H	-4.14944600	2.68511000	-2.32149400
H	-4.06389400	0.90871100	-2.49323300
C	1.89014300	4.05788000	-2.38125500
H	1.85550800	5.11648500	-2.69360300
H	2.87654500	3.65145200	-2.66560900
H	1.11933900	3.50276500	-2.94660100
C	0.36795900	4.79971300	1.12455900
H	0.92882500	5.75078800	1.19697300
H	-0.63754100	4.95492700	1.55553600
H	0.89085700	4.04377600	1.73715900
C	-0.40432000	5.45834100	-1.18005800
H	-1.36064600	5.76495700	-0.71932400
H	0.24389600	6.35304700	-1.21345500
H	-0.61432900	5.14187200	-2.21598900
C	1.31628300	1.48312100	3.00757100
H	1.91436400	0.61181300	3.33277600
H	1.72751700	1.85349200	2.05312700
H	1.42316200	2.27600000	3.77148500

$(CH_3)_{15}$(corrin)Co(I) - Propylene oxide – TS2b

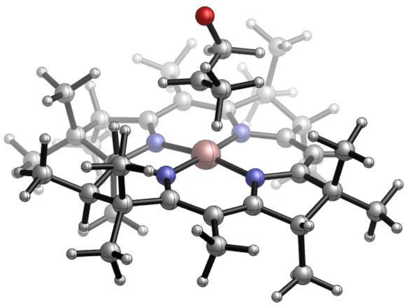

E (BP86-D3/6-31G(d) + SMD (acetone)) = -3121.590068
E (BP86-D3/6-311++G(2df,p)// BP86-D3/6-31G(d) + SMD (acetone)) = -3122.237732

Zero-point correction=	0.860720
Thermal correction to Energy=	0.905896
Thermal correction to Enthalpy=	0.906841
Thermal correction to Gibbs Free Energy=	0.790292

Charge = 0 Multiplicity = 1

C	-3.00724000	-0.39843900	-0.08430200
C	-4.35624600	0.34798900	0.03397600
C	-3.97374400	1.73401000	-0.56695200
C	-2.49071500	1.78123500	-0.29491700
N	-1.94752100	0.52743400	-0.17967400
H	-4.50882500	2.54782000	-0.04281800

C	-1.77288900	2.97170700	-0.24063600
H	-2.35010400	3.90045200	-0.28081000
C	-2.83490400	-1.77609200	-0.02787200
C	-0.38762900	3.06724900	-0.19373300
C	0.36763800	4.38669800	-0.10048700
N	0.45808600	1.98267900	-0.21955000
C	1.74267500	3.95728300	-0.68227300
C	1.76765700	2.46164000	-0.37783300
C	-1.53848300	-2.34879100	-0.23411100
C	-1.27439800	-3.83483300	-0.43736300
N	-0.42889200	-1.61651400	-0.31058400
C	0.28793100	-3.92576700	-0.28035800
C	0.73839000	-2.47859500	-0.70169400
C	2.92849500	1.69794100	-0.34750300
C	2.85850000	0.27273500	-0.19989900
C	4.04176600	-0.69500700	-0.00243600
N	1.68458900	-0.35800700	-0.11648200
C	3.35725000	-2.06829300	-0.32277200
C	1.89686200	-1.82028000	0.07092800
Co	-0.07294500	0.16995100	-0.12241100
H	2.55154700	4.49984000	-0.16304400
H	3.40970200	-2.18819700	-1.42148500
H	-1.73512600	-4.40773700	0.39036600
H	1.76909000	-2.03004200	1.14799900
C	0.09606000	-0.03485900	2.37141800
C	-1.31729500	0.05189800	2.81468600
H	-2.01355800	-0.65599400	2.31218500
O	-0.80199500	-0.33410500	4.03702500
H	0.48191400	-1.05017900	2.25229500
C	4.27827100	2.37842300	-0.49644600
H	4.98868200	2.04110600	0.27726300
H	4.74442500	2.17216400	-1.47715400
H	4.20104100	3.47109300	-0.39845600
C	4.48562700	-0.59895500	1.47982400
H	4.82005000	0.42538700	1.72017200
H	3.65792900	-0.85167400	2.16689200
H	5.32829700	-1.28417800	1.68405200
C	5.25499900	-0.51540300	-0.93848500
H	5.92757900	0.29809800	-0.62527900
H	5.85247200	-1.44628500	-0.93424900
H	4.93552900	-0.32533900	-1.97884600
C	4.01229700	-3.28590200	0.33508900
H	3.57443700	-4.22531900	-0.04179300
H	5.09551100	-3.31900500	0.11699600
H	3.88700600	-3.27521900	1.43215600
C	0.95182600	-2.31703200	-2.22253000
H	1.77783200	-2.94669400	-2.59214100
H	0.03907200	-2.58395800	-2.77725400
H	1.18333900	-1.26150300	-2.44782700
C	0.91845300	-5.05107300	-1.11581900
H	2.01713000	-5.05309700	-1.00877400
H	0.54976700	-6.03363400	-0.76799600
H	0.68768200	-4.96933500	-2.19015800
C	0.57429100	-4.19874200	1.21493400
H	0.13152700	-5.17007400	1.50238600
H	1.65476200	-4.25073200	1.43066600
H	0.12828200	-3.42300200	1.86480000
C	-1.89232100	-4.38296000	-1.74257000
H	-1.68805600	-5.46300600	-1.84754200
H	-2.98797100	-4.25326100	-1.73144500

H	-1.50791800	-3.87102100	-2.64018900
C	-3.99232500	-2.71725200	0.26522000
H	-4.66248400	-2.30742100	1.03722200
H	-4.61083800	-2.93991800	-0.62344200
H	-3.62427100	-3.68120600	0.65183000
C	-5.56697100	-0.27556500	-0.68879900
H	-6.00514900	-1.11342000	-0.12607700
H	-6.35860100	0.48951100	-0.79003600
H	-5.31004200	-0.63661300	-1.69933500
C	-4.69644300	0.54752800	1.53508700
H	-3.90511700	1.12097300	2.04879400
H	-5.64532900	1.10794000	1.63213200
H	-4.81366400	-0.41738800	2.05800400
C	-4.20895900	1.88857200	-2.08752400
H	-5.28300400	1.86461000	-2.34170100
H	-3.79976000	2.85530500	-2.43128300
H	-3.69919700	1.08498800	-2.65009600
C	1.89739100	4.17107900	-2.20384700
H	1.86944800	5.24382300	-2.46447500
H	2.86107200	3.76103000	-2.55317900
H	1.09015200	3.65730000	-2.75727800
C	0.54094700	4.74817900	1.39901300
H	1.10459400	5.69467200	1.50190700
H	-0.44190600	4.87332700	1.88803700
H	1.09563400	3.95833500	1.93623500
C	-0.30821400	5.55936900	-0.82810600
H	-1.23624400	5.85933300	-0.30874700
H	0.36044100	6.43937100	-0.83800400
H	-0.56980300	5.30924100	-1.87044300
H	-1.74317300	1.08522600	2.74038500
C	1.07848200	1.03991900	2.76679900
H	1.64960000	0.75102900	3.66743900
H	1.80691300	1.23642800	1.96539300
H	0.54713500	1.98338700	2.98244300

$(CH_3)_{15}$(corrin)Co(I) – cycloxexane oxide – TS3

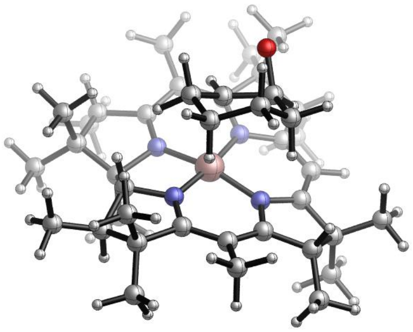

E (BP86-D3/6-31G(d) + SMD (acetone)) = -3238.344929
E (BP86-D3/6-311++G(2df,p)// BP86-D3/6-31G(d) + SMD (acetone)) = -3239.020761

Zero-point correction=	0.925281
Thermal correction to Energy=	0.971894
Thermal correction to Enthalpy=	0.972838
Thermal correction to Gibbs Free Energy=	0.854170

Charge = 0 Multiplicity = 1

C	3.03053400	-0.60714300	-0.01642000

C	3.97700300	-1.80752700	0.19110300
C	3.24906300	-2.90340700	-0.65266500
C	1.82494400	-2.40421600	-0.64291700
N	1.74705500	-1.06730300	-0.36987600
H	3.33070900	-3.88902200	-0.15732800
C	0.73249200	-3.21436700	-0.93733100
H	0.94287500	-4.25801700	-1.19139100
C	3.34612700	0.73302300	0.17133400
C	-0.59945300	-2.81114000	-0.91855400
C	-1.73668200	-3.72949300	-1.33293000
N	-1.02740800	-1.54845100	-0.59464600
C	-2.97226900	-2.96507600	-0.75930500
C	-2.43085100	-1.54731100	-0.54808400
C	2.38313200	1.74302700	-0.16088100
C	2.70947400	3.20962300	-0.39192100
N	1.10002400	1.46620300	-0.36758100
C	1.28576300	3.88046300	-0.41195900
C	0.36162600	2.67986200	-0.85580800
C	-3.21546400	-0.43257400	-0.26900200
C	-2.63577800	0.87764600	-0.22517100
C	-3.39441800	2.22134700	-0.17931600
N	-1.31214100	1.05681700	-0.22592100
C	-2.27238500	3.18575900	-0.70241600
C	-1.00386700	2.51645200	-0.16306200
Co	0.12072200	-0.07123600	-0.28987000
H	-3.17489900	-3.38522600	0.25059500
H	-2.27277300	3.08966200	-1.80553700
H	3.26872200	3.61244500	0.47407300
H	-0.88620300	2.77511600	0.90415800
C	0.46458400	-0.34400800	2.19043000
C	0.22957400	-1.79640000	2.45676400
H	0.64756100	-2.47724100	1.68216800
O	1.04359500	-1.57141700	3.56225000
H	1.48213100	-0.05193000	1.92406700
C	-4.68929600	-0.61247300	0.05587900
H	-5.04190900	0.17998800	0.73218600
H	-5.34893000	-0.60110200	-0.83084700
H	-4.85576300	-1.56545700	0.58533300
C	-3.79635200	2.57702100	1.27483400
H	-4.45844800	1.81005600	1.70958300
H	-2.91475100	2.66593800	1.93314800
H	-4.34126400	3.53819000	1.29734600
C	-4.62106700	2.30230000	-1.11016300
H	-5.50939200	1.80158800	-0.69310000
H	-4.89212100	3.36275500	-1.26935000
H	-4.40272500	1.85566000	-2.09706400
C	-2.44999500	4.66044100	-0.33657400
H	-1.70544400	5.28557500	-0.85707200
H	-3.45008700	5.02507800	-0.63467100
H	-2.33447500	4.83377600	0.74751400
C	0.20738300	2.55177000	-2.38540700
H	-0.31633600	3.42054300	-2.81773100
H	1.19054800	2.45901900	-2.87332800
H	-0.36932400	1.63869500	-2.61642300
C	1.21705400	5.11008300	-1.33206800
H	0.20552200	5.55141200	-1.32725400
H	1.91708700	5.88751800	-0.97438100
H	1.47914700	4.88094900	-2.37747900
C	0.98195600	4.33459600	1.03476400
H	1.72680500	5.08970600	1.34640800

H	-0.01590200	4.79698900	1.12450900
H	1.03974900	3.49323700	1.75006400
C	3.61167500	3.41233200	-1.62933700
H	3.84059600	4.48192500	-1.77929800
H	4.57075700	2.88388400	-1.49026800
H	3.14952400	3.02453800	-2.55271500
C	4.70033700	1.18458900	0.69282700
H	5.05045700	0.54114200	1.51596200
H	5.48883500	1.18877300	-0.08190600
H	4.63903900	2.20796500	1.09754800
C	5.44449600	-1.63816400	-0.25026400
H	6.03134800	-1.04569900	0.46738800
H	5.92060600	-2.63442900	-0.30686300
H	5.53226500	-1.16230200	-1.24184500
C	3.93593800	-2.19468300	1.69474900
H	2.90635300	-2.29048100	2.08399000
H	4.46996700	-3.15107000	1.85286200
H	4.43805800	-1.42277700	2.30536800
C	3.72769000	-3.04772800	-2.11572600
H	4.76375600	-3.42396200	-2.17571200
H	3.07689600	-3.76193500	-2.65129400
H	3.67599800	-2.07942400	-2.64641200
C	-4.24237600	-3.14785300	-1.61018700
H	-4.34451700	-4.21161900	-1.89216700
H	-5.15907300	-2.87429600	-1.06879000
H	-4.20270600	-2.55081000	-2.53767600
C	-1.63793300	-5.15048100	-0.75109400
H	-2.56575400	-5.71195300	-0.96959400
H	-0.79782900	-5.71746900	-1.19118200
H	-1.50012400	-5.12294800	0.34491000
C	-1.73002700	-3.79739100	-2.88336000
H	-0.76470600	-4.20776100	-3.23001500
H	-2.53297800	-4.45160700	-3.26720100
H	-1.85353100	-2.79448500	-3.33064400
C	-0.37659900	0.68256200	2.91790700
H	-0.37046800	1.62201400	2.33808700
H	0.11250400	0.91973500	3.88337700
C	-1.21592300	-2.20171900	2.79284000
H	-1.80453500	-2.25452000	1.85740900
H	-1.19519100	-3.22036700	3.22873500
C	-1.86822700	-1.20390600	3.76407300
H	-1.31433100	-1.22523900	4.72221200
H	-2.91390600	-1.49838000	3.97687000
C	-1.82204900	0.21370300	3.16842200
H	-2.36653800	0.20579400	2.20654600
H	-2.34085500	0.93847000	3.82474500

($(CH_3)_{15}$(corrin)Co(I) - *cis*-1-phenylpropylene oxide – TS4a

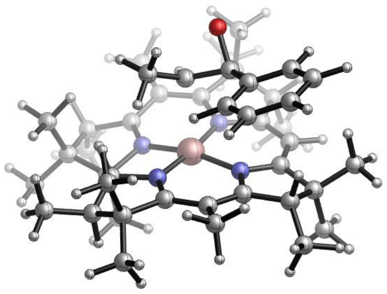

E (BP86-D3/6-31G(d) + SMD (acetone)) = -3352.66206
E (BP86-D3/6-311++G(2df,p)// BP86-D3/6-31G(d) + SMD (acetone)) = -3353.373699

Zero-point correction=	0.940140
Thermal correction to Energy=	0.989665
Thermal correction to Enthalpy=	0.990609
Thermal correction to Gibbs Free Energy=	0.864500

Charge = 0 Multiplicity = 1

C	2.01640900	-2.52315600	0.04273900
C	1.80227400	-4.04121900	0.21771700
C	0.63198400	-4.28794000	-0.78623600
C	-0.01669200	-2.92702600	-0.83841400
N	0.83705300	-1.93103700	-0.45179800
H	-0.06521200	-5.04865700	-0.38799300
C	-1.31846700	-2.72342100	-1.28303100
H	-1.87571900	-3.60751400	-1.60836600
C	3.15647900	-1.80661400	0.38554400
C	-1.96962300	-1.49504700	-1.34117100
C	-3.35802000	-1.34298700	-1.93788400
N	-1.42048800	-0.30035300	-0.94952700
C	-3.74884300	0.08523800	-1.44499900
C	-2.40126200	0.69889600	-1.05378100
C	3.22571400	-0.40117800	0.10870800
C	4.50681900	0.41664200	0.08338600
N	2.15491400	0.31763500	-0.21456600
C	3.97000900	1.89511400	0.08015800
C	2.54178800	1.72536700	-0.56906000
C	-2.20850700	2.03854400	-0.73284600
C	-0.90321500	2.54128800	-0.42491400
C	-0.51503600	4.01810600	-0.20271000
N	0.14774700	1.72861300	-0.28389500
C	1.03192200	3.94141700	-0.45212300
C	1.37000600	2.52688800	0.03022100
Co	0.38354700	-0.07811600	-0.39371100
H	-4.31688500	-0.03691100	-0.49636100
H	1.16748000	3.97491500	-1.55039700
H	5.07541300	0.26028500	1.01968300
H	1.48083300	2.53083900	1.12840800
C	0.13378500	-0.54160100	2.07898000
C	-1.11465800	-1.35257400	2.10620300
H	-1.12295700	-2.12299000	1.30441500
O	-0.61019400	-1.80216400	3.33014900
C	-2.50668800	-0.73909900	2.16614100
C	-3.58585800	-1.64199600	2.29505000
C	-2.79526500	0.63867700	2.13150400

C	-4.90984600	-1.18875700	2.40025300
H	-3.37577000	-2.71927200	2.30983700
C	-4.11921000	1.10063500	2.24563700
H	-1.99040800	1.36211200	1.98537400
C	-5.18189000	0.19194200	2.38010400
H	-5.72972600	-1.91090400	2.49348900
H	-4.31790200	2.17814800	2.21903600
H	-6.21307500	0.55432000	2.46357500
H	1.03249000	-1.12680400	1.88162700
C	-3.41057700	2.96078200	-0.62490300
H	-3.22930300	3.76407000	0.10329800
H	-3.68816700	3.44322000	-1.57998500
H	-4.29002100	2.40959100	-0.25647600
C	-0.83241600	4.43155200	1.25710600
H	-1.90887200	4.33023400	1.47663500
H	-0.28446300	3.80927000	1.98691900
H	-0.55572700	5.48725000	1.43114800
C	-1.13812000	5.02058400	-1.19627300
H	-2.17667800	5.28905700	-0.94631100
H	-0.55084500	5.95778800	-1.18569600
H	-1.12172700	4.62226600	-2.22683200
C	1.85182900	5.06895000	0.17806800
H	2.90152000	5.02649600	-0.15734100
H	1.45438400	6.05836100	-0.11397400
H	1.84883700	5.01427900	1.28076100
C	2.54582900	1.84254300	-2.10726400
H	2.84006500	2.85196900	-2.43966500
H	3.23906600	1.11307900	-2.55513000
H	1.53382900	1.62421500	-2.49123200
C	4.89609800	2.86836500	-0.66678500
H	4.48400000	3.89238500	-0.65806500
H	5.88256500	2.90610100	-0.16889300
H	5.06410200	2.58247300	-1.71756900
C	3.88017000	2.34103500	1.55814600
H	4.89059300	2.33121000	2.00658000
H	3.48183800	3.36451300	1.66064200
H	3.24358000	1.66030000	2.15275600
C	5.43812500	-0.01017300	-1.07246600
H	6.36467500	0.59032700	-1.07463200
H	5.72805500	-1.06872500	-0.95334000
H	4.95586400	0.09328100	-2.05902900
C	4.36203100	-2.46602300	1.03493800
H	4.05800800	-3.18374700	1.81353800
H	5.00143700	-3.00982700	0.31567300
H	4.99862800	-1.71580500	1.53080100
C	2.99659500	-4.97229800	-0.07062200
H	3.72600400	-4.98557600	0.75294200
H	2.62483300	-6.00725900	-0.18493900
H	3.52875400	-4.69766200	-0.99724300
C	1.30121400	-4.28277900	1.66829100
H	0.45143900	-3.63068600	1.94070500
H	0.99241100	-5.33848500	1.79022900
H	2.10863500	-4.08031600	2.39489300
C	1.05505900	-4.70543300	-2.21345600
H	1.52286000	-5.70515000	-2.22913700
H	0.16752800	-4.73656400	-2.87052300
H	1.76875600	-3.97868200	-2.64291000
C	-4.64661000	0.83658500	-2.44597500
H	-5.42889800	0.15151800	-2.82050000
H	-5.16709900	1.69257000	-1.99416000

H	-4.07300500	1.20586800	-3.31370700
C	-4.36609800	-2.39797700	-1.44937200
H	-5.38034700	-2.14446400	-1.81154500
H	-4.12215800	-3.40699200	-1.82823000
H	-4.39287200	-2.43632100	-0.34573900
C	-3.20596900	-1.43867300	-3.47957400
H	-2.78370800	-2.42360400	-3.74787100
H	-4.17825200	-1.33706900	-3.99360000
H	-2.52396100	-0.65986900	-3.86633600
C	0.31421900	0.71200500	2.90325000
H	1.34076300	0.77606100	3.30458700
H	0.13362800	1.62596600	2.31347000
H	-0.39084800	0.71684800	3.75048400

(CH$_3$)$_{15}$(corrin)Co(I) - *trans*-1-phenylpropylene oxide – TS4b

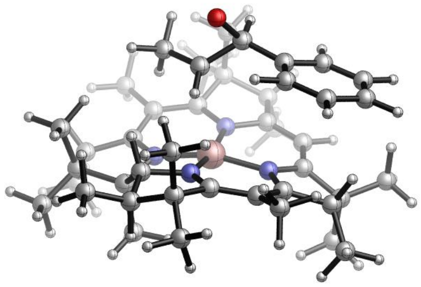

E (BP86-D3/6-31G(d) + SMD (acetone)) = -3352.662226
E (BP86-D3/6-311++G(2df,p)// BP86-D3/6-31G(d) + SMD (acetone)) = -3353.373598

Zero-point correction=	0.938001
Thermal correction to Energy=	0.988071
Thermal correction to Enthalpy=	0.989015
Thermal correction to Gibbs Free Energy=	0.861067

Charge = 0 Multiplicity = 1

C	1.94354400	-2.53425200	0.05994100
C	1.69349000	-3.98988400	0.50951300
C	0.40659000	-4.33425400	-0.30526100
C	-0.19606700	-2.96529300	-0.52531600
N	0.71924400	-1.96085800	-0.33475700
H	-0.26379900	-4.98015400	0.29328900
C	-1.47496100	-2.76668400	-1.04244400
H	-2.05613000	-3.65911500	-1.29616100
C	3.15089500	-1.83987400	0.08740500
C	-2.04188400	-1.52554100	-1.31304400
C	-3.30270100	-1.31701700	-2.13319700
N	-1.47939400	-0.33246800	-0.92677300
C	-3.73176900	0.11043600	-1.66287000
C	-2.43649400	0.68024600	-1.07679400
C	3.19926200	-0.45710100	-0.29130100
C	4.46324000	0.36479900	-0.48944900
N	2.09417200	0.26433300	-0.47811100
C	3.93272800	1.84092400	-0.36010900
C	2.42729900	1.69185500	-0.80457600
C	-2.26507700	1.99700200	-0.65702700
C	-0.96532400	2.48238200	-0.30399100
C	-0.56919700	3.92564100	0.06372900
N	0.09298300	1.66626400	-0.26839700
C	0.96292000	3.89191900	-0.28206200
C	1.33987100	2.43409900	0.01008200
Co	0.32191200	-0.12671800	-0.45059300
H	-4.44605400	-0.02108400	-0.82343200
H	1.03044900	4.05440700	-1.37524200
H	5.17123700	0.18763700	0.34173300
H	1.57145900	2.31261400	1.08424300
C	0.17307500	0.09673200	2.57527400
C	-0.97297900	-0.64535300	3.19166400
H	-0.73445800	-1.71217100	3.40638500
O	-0.69992800	0.25075300	4.25076800
C	-2.36220500	-0.53184600	2.60010300
C	-3.06396400	-1.67377200	2.17037300

C	-3.00710900	0.72084100	2.57067800
C	-4.39713100	-1.57061200	1.73951800
H	-2.56880900	-2.65251200	2.19642600
C	-4.33374000	0.82911300	2.12800100
H	-2.46178800	1.59925200	2.93308000
C	-5.03654100	-0.31902200	1.71833000
H	-4.94528300	-2.47013100	1.43742900
H	-4.82645300	1.80775200	2.11445800
H	-6.07935600	-0.23956900	1.39022100
H	-0.06130900	1.04615800	2.09090100
C	-3.47659600	2.90401500	-0.52945500
H	-3.37321700	3.57112200	0.34108400
H	-3.65038900	3.54524100	-1.41334800
H	-4.39005000	2.31356200	-0.35688300
C	-0.81560100	4.14201700	1.57859400
H	-1.88546300	4.02045600	1.82216200
H	-0.24975500	3.41715700	2.19184100
H	-0.51659000	5.16120400	1.88494600
C	-1.24452900	5.04345500	-0.75624400
H	-2.26689900	5.27069700	-0.41554900
H	-0.65731900	5.97542600	-0.65629900
H	-1.28536900	4.78292600	-1.82922200
C	1.81542800	4.94933500	0.42270200
H	2.84384600	4.95819500	0.02262600
H	1.39671200	5.96128300	0.27171900
H	1.87849100	4.76771800	1.51019500
C	2.21069600	1.90836800	-2.31665100
H	2.43973200	2.94326900	-2.62074900
H	2.84602900	1.22644100	-2.90391400
H	1.15904900	1.68823400	-2.56866800
C	4.73404700	2.85477500	-1.19292400
H	4.30676000	3.86865400	-1.09282100
H	5.77882300	2.89815500	-0.83367300
H	4.76080200	2.60593900	-2.26605400
C	4.06451000	2.22615600	1.13167100
H	5.13235300	2.22092200	1.41822000
H	3.67052600	3.23396400	1.34118800
H	3.54133700	1.50751600	1.78634600
C	5.20136000	-0.02735800	-1.78718600
H	6.11915200	0.57257500	-1.91979800
H	5.49947200	-1.09030800	-1.74055400
H	4.56981600	0.10129800	-2.68242300
C	4.43517200	-2.49404100	0.57174700
H	4.28206600	-3.01470500	1.53245800
H	4.84226700	-3.23115100	-0.14299200
H	5.22245300	-1.74434200	0.74333100
C	2.79980100	-5.03822500	0.28662100
H	3.61033100	-4.95469700	1.02621100
H	2.36232000	-6.04693900	0.40409900
H	3.24337100	-4.97649100	-0.72134300
C	1.33871300	-3.93909100	2.02037200
H	0.48414100	-3.26325900	2.20557700
H	1.06855200	-4.94834900	2.38476300
H	2.19743100	-3.57588100	2.61269700
C	0.65564900	-5.01496400	-1.67079900
H	1.08507600	-6.02550000	-1.55554700
H	-0.29686100	-5.11475500	-2.22073700
H	1.34289500	-4.41178200	-2.29214700
C	-4.42683700	0.93499900	-2.76165400
H	-5.19587700	0.31645700	-3.25993000

H	-4.93977700	1.82234600	-2.36181500
H	-3.71177400	1.27675200	-3.53000900
C	-4.40723100	-2.36061100	-1.90739200
H	-5.33304900	-2.04954700	-2.42624200
H	-4.11999600	-3.34920600	-2.31000200
H	-4.63249700	-2.47815200	-0.83418800
C	-2.86704500	-1.34706400	-3.62488900
H	-2.42901900	-2.33366600	-3.86028400
H	-3.72439000	-1.18483800	-4.30278300
H	-2.10399700	-0.57640100	-3.83745200
C	1.58184500	-0.29747700	2.86948100
H	1.61275600	-1.09702600	3.62801700
H	2.06713200	-0.67885900	1.95506700
H	2.17675600	0.56109200	3.22869200

6.6 Kinetic studies for model reaction

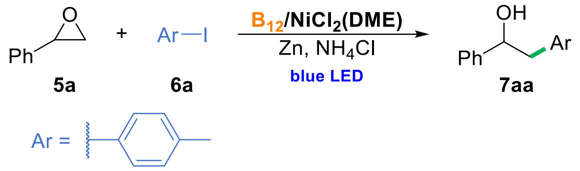

Reaction conditions: styrene oxide (**5a**) (0.2 mmol), 4-iodotoluene (**6a**) (1.5 equiv.), Zn (1.5 equiv.), NH_4Cl (3 equiv.), B_{12} (**1**) (5mol%), $NiCl_2(DME)$ (20mol%), dtbbpy (40mol%), H_2O (1.1 equiv.), dry NMP (c = 0.1 M), Blue LED (single diode 3 W).

The reaction was setup according to the procedure **A** on 0.4 mmol scale with the addition of dodecane as an internal standard (58 mg). The reaction was monitored by GC/FID for 16 h.

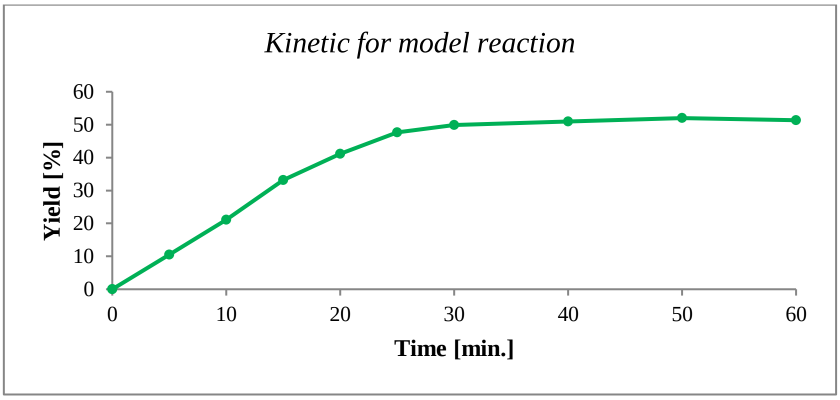

<u>Conclusion:</u> *This experiment suggests that optimal time for this reaction is only 30 minutes.*

6.7 Reactions with deuterated solvents

<u>Side-products by reaction with aryl epoxide</u>

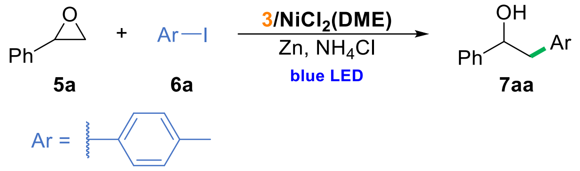

Reaction conditions: styrene oxide (**5a**) (0.2 mmol), 4-iodotoluene (**6a**) (1.5 equiv.), Zn (1.5 equiv.), NH_4Cl (3 equiv.), HME (**3**) (5mol%), $NiCl_2(DME)$ (20mol%), dtbbpy (40 mol%), acetone-d6, Blue LED (single diode, 3 W), 16 h.

The reaction was setup according to the procedure **D** (acetone was replacement on acetone-d_6). After 16 h a small portion was taken from the reaction mixture and ^1H NMR spectrum was recorded. This experiment enabled identification of side-products formed.

^{1}H NMR of the crude reaction mixture:

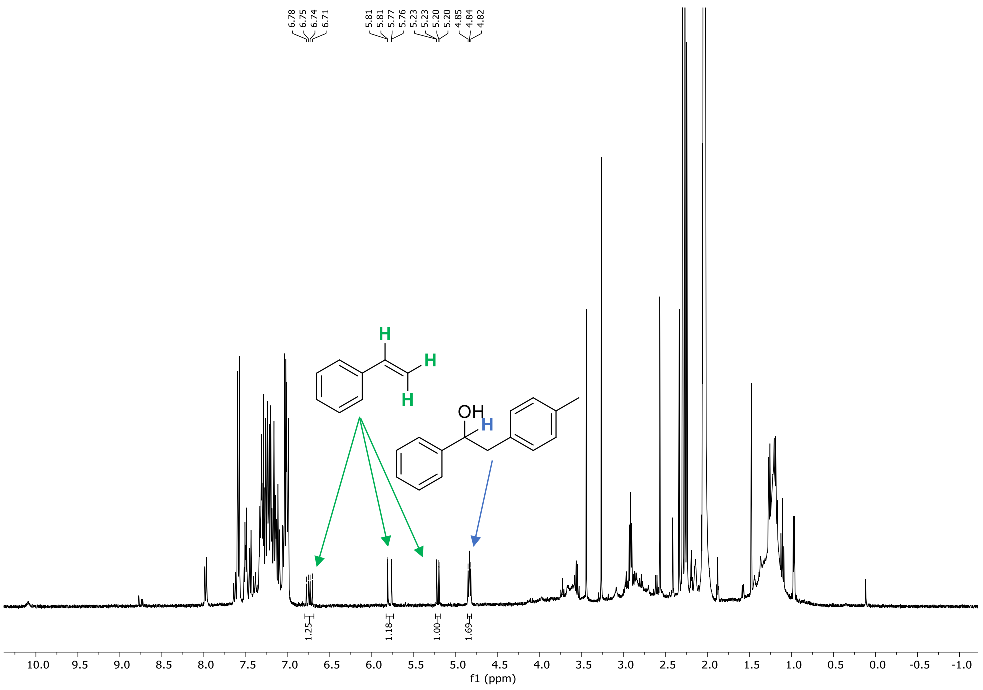

We compared the obtained ^{1}H NMR spectrum with spectra measured for the samples of the original styrene and product in the same deuterated solvent.

^{1}H NMR spectrum of styrene (solvent: acetone-d_6):

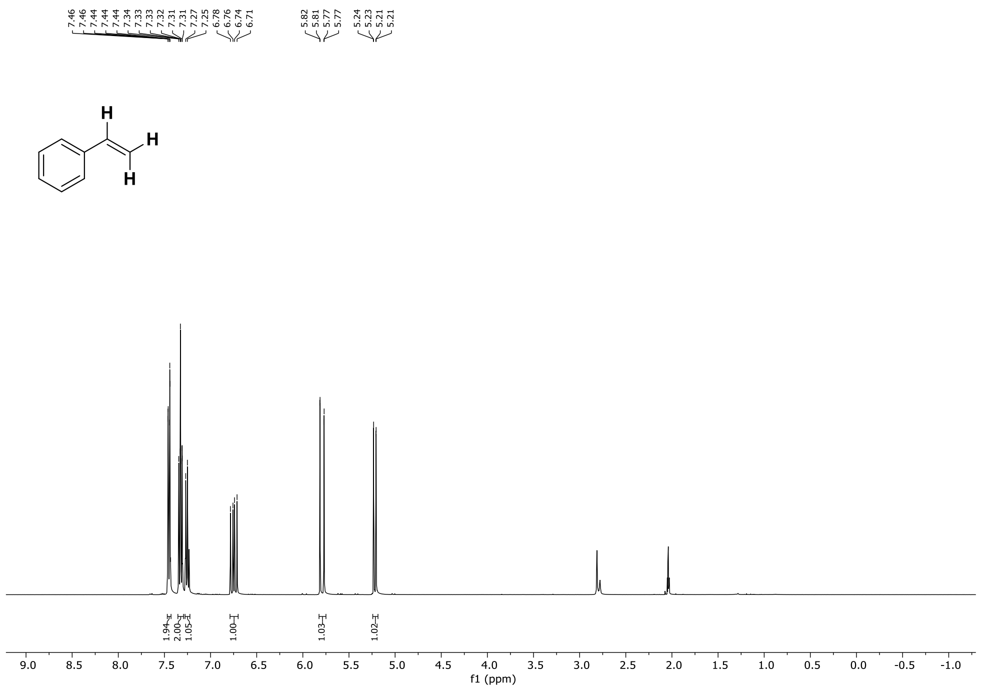

^{1}H NMR spectrum of product (7aa) (solvent: acetone-d_6):

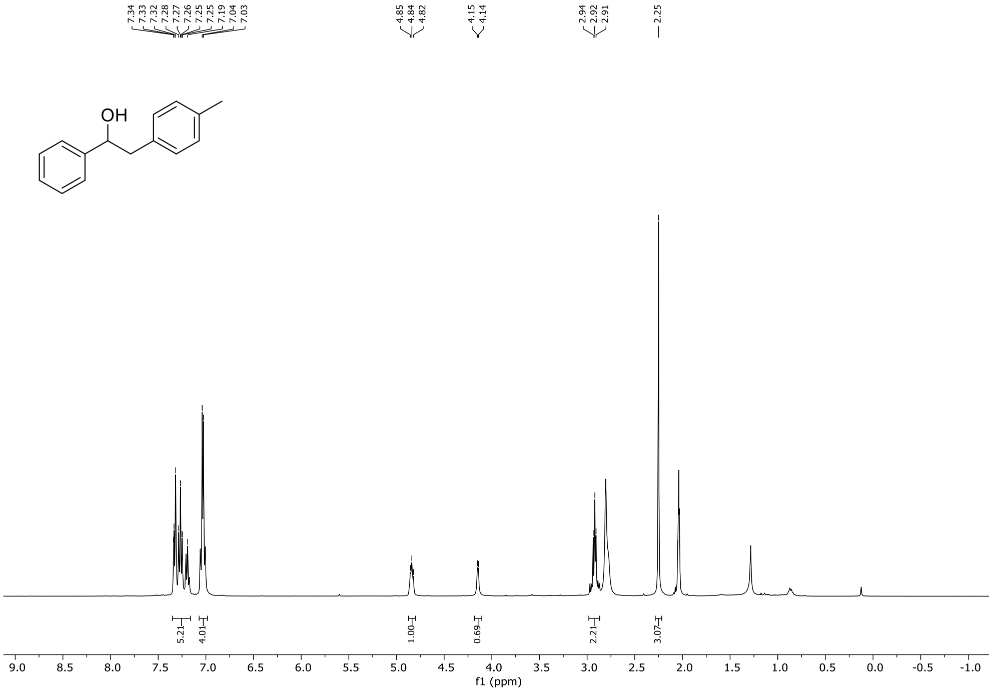

<u>Conclusion</u>: *Based on ^{1}H NMR spectrum, the major side-product is styrene. The ratio between* ***product*** *and* ***styrene*** *is* ***1:0.6****.*

7. References

(1) HISANO, T.; TASAKI, M.; TSUMOTO, K.; MATSUOKA, T.; ICHIKAWA, M. Synthesis and Antiinflammatory Activity of N1-(Substituted Phenyl)Pyridinecarboxamidines. *Chem. Pharm. Bull. (Tokyo).* **1983**, *31* (7), 2484–2490. https://doi.org/10.1248/cpb.31.2484.

(2) Chen, G.; Shigenari, T.; Jain, P.; Zhang, Z.; Jin, Z.; He, J.; Li, S.; Mapelli, C.; Miller, M. M.; Poss, M. A.; Scola, P. M.; Yeung, K.-S.; Yu, J.-Q. Ligand-Enabled β-C–H Arylation of α-Amino Acids Using a Simple and Practical Auxiliary. *J. Am. Chem. Soc.* **2015**, *137* (9), 3338–3351. https://doi.org/10.1021/ja512690x.

(3) Ma, X.; Pan, S.; Wang, H.; Chen, W. Rhodium-Catalyzed Transannulation of N-Sulfonyl-1,2,3-Triazoles and Epoxides: Regioselective Synthesis of Substituted 3,4-Dihydro-2 H-1,4-Oxazines. *Org. Lett.* **2014**, *16* (17), 4554–4557. https://doi.org/10.1021/ol5021042.

(4) Li, S.; Shi, Y.; Li, P.; Xu, J. Nucleophilic Organic Base DABCO-Mediated Chemospecific Meinwald Rearrangement of Terminal Epoxides into Methyl Ketones. *J. Org. Chem.* **2019**, *84* (7), 4443–4450. https://doi.org/10.1021/acs.joc.8b03171.

(5) Lopchuk, J. M.; Fjelbye, K.; Kawamata, Y.; Malins, L. R.; Pan, C.-M.; Gianatassio, R.; Wang, J.; Prieto, L.; Bradow, J.; Brandt, T. A.; Collins, M. R.; Elleraas, J.; Ewanicki, J.; Farrell, W.; Fadeyi, O. O.; Gallego, G. M.; Mousseau, J. J.; Oliver, R.; Sach, N. W.; Smith, J. K.; Spangler, J. E.; Zhu, H.; Zhu, J.; Baran, P. S. Strain-Release Heteroatom Functionalization: Development, Scope, and Stereospecificity. *J. Am. Chem. Soc.* **2017**, *139* (8), 3209–3226. https://doi.org/10.1021/jacs.6b13229.

(6) Weijers, C. A. G. M.; Könst, P. M.; Franssen, M. C. R.; Sudhölter, E. J. R. Stereochemical Preference of Yeast Epoxide Hydrolase for the O-Axial C3 Epimers of 1-Oxaspiro[2.5]Octanes, 2007, Vol. 5. https://doi.org/10.1039/b709742e.

(7) Steiman, T. J.; Liu, J.; Mengiste, A.; Doyle, A. G. Synthesis of β-Phenethylamines via Ni/Photoredox Cross-Electrophile Coupling of Aliphatic Aziridines and Aryl Iodides. *J. Am. Chem. Soc.* **2020**, *142* (16), 7598–7605. https://doi.org/10.1021/jacs.0c01724.

(8) Singh, A.; Anandhi, U.; Cinellu, M. A.; Sharp, P. R. Diimine Supported Group 10 Hydroxo, Oxo, Amido, and Imido Complexes. *Dalt. Trans.* **2008**, No. 17, 2314–2327. https://doi.org/10.1039/b715663d.

(9) Ociepa, M.; Wierzba, A. J.; Turkowska, J.; Gryko, D. Polarity-Reversal Strategy for the Functionalization of Electrophilic Strained Molecules via Light-Driven Cobalt Catalysis. *J. Am. Chem. Soc.* **2020**, *142* (11), 5355–5361. https://doi.org/10.1021/jacs.0c00245.

(10) Weiss, M. E.; Kreis, L. M.; Lauber, A.; Carreira, E. M. Cobalt-Catalyzed Coupling of Alkyl Iodides with Alkenes: Deprotonation of Hydridocobalt Enables Turnover. *Angew. Chemie Int. Ed.* **2011**, *50* (47), 11125–11128. https://doi.org/10.1002/anie.201105235.

(11) Nielsen, D. K.; Doyle, A. G. Nickel-Catalyzed Cross-Coupling of Styrenyl Epoxides with

Boronic Acids. *Angew. Chemie Int. Ed.* **2011**, *50* (27), 6056–6059. https://doi.org/10.1002/anie.201101191.

(12) Taniguchi, T.; Zaimoku, H.; Ishibashi, H. A Mild Oxidative Aryl Radical Addition into Alkenes by Aerobic Oxidation of Arylhydrazines. *Chem. - A Eur. J.* **2011**, *17* (15), 4307–4312. https://doi.org/10.1002/chem.201003060.

(13) Suh, Y.; Lee, J.; Kim, S.-H.; Rieke, R. D. Direct Preparation of Benzylic Manganese Reagents from Benzyl Halides, Sulfonates, and Phosphates and Their Reactions: Applications in Organic Synthesis. *J. Organomet. Chem.* **2003**, *684* (1–2), 20–36. https://doi.org/10.1016/S0022-328X(03)00500-X.

(14) Wang, J.; Xue, L.; Hong, M.; Ni, B.; Niu, T. Heterogeneous Visible-Light-Induced Meerwein Hydration Reaction of Alkenes in Water Using Mpg-C 3 N 4 as a Recyclable Photocatalyst. *Green Chem.* **2020**, *22* (2), 411–416. https://doi.org/10.1039/C9GC03679B.

(15) Zhao, Y.; Weix, D. J. Nickel-Catalyzed Regiodivergent Opening of Epoxides with Aryl Halides: Co-Catalysis Controls Regioselectivity. *J. Am. Chem. Soc.* **2014**, *136* (1), 48–51. https://doi.org/10.1021/ja410704d.

(16) Harada, T.; Kaneko, T.; Fujiwara, T.; Oku, A. A Novel 1,2-Migration of Arylzincates Bearing a Leaving Group at Benzylic Position: Application to a Three-Component Coupling of p-Iodobenzyl Derivatives, Trialkylzincates, and Electrophiles Leading to Functionalized p-Substituted Benzenes. *Tetrahedron* **1998**, *54* (32), 9317–9332. https://doi.org/10.1016/S0040-4020(98)00569-9.

(17) Parasram, M.; Shields, B. J.; Ahmad, O.; Knauber, T.; Doyle, A. G. Regioselective Cross-Electrophile Coupling of Epoxides and (Hetero)Aryl Iodides via Ni/Ti/Photoredox Catalysis. *ACS Catal.* **2020**, *10* (10), 5821–5827. https://doi.org/10.1021/acscatal.0c01199.

(18) Li, C.; Kan, J.; Qiu, Z.; Li, J.; Lv, L.; Li, C. Synergistic Relay Reactions To Achieve Redox-Neutral A-Alkylations of Olefinic Alcohols with Ruthenium(II) Catalysis. *Angew. Chemie Int. Ed.* **2020**, *59* (11), 4544–4549. https://doi.org/10.1002/anie.201915218.

(19) Woods, B. P.; Orlandi, M.; Huang, C.-Y.; Sigman, M. S.; Doyle, A. G. Nickel-Catalyzed Enantioselective Reductive Cross-Coupling of Styrenyl Aziridines. *J. Am. Chem. Soc.* **2017**, *139* (16), 5688–5691. https://doi.org/10.1021/jacs.7b03448.

(20) Guo, J.; Wu, Q. L.; Xie, Y.; Weng, J.; Lu, G. Visible-Light-Mediated Decarboxylative Benzylation of Imines with Arylacetic Acids. *J. Org. Chem.* **2018**, *83* (20), 12559–12567. https://doi.org/10.1021/acs.joc.8b01849.

(21) Grimme, S.; Antony, J.; Ehrlich, S.; Krieg, H. A Consistent and Accurate Ab Initio Parametrization of Density Functional Dispersion Correction (DFT-D) for the 94 Elements H-Pu. *J. Chem. Phys.* **2010**, *132* (15). https://doi.org/10.1063/1.3382344.

(22) Marenich, A. V.; Cramer, C. J.; Truhlar, D. G. Universal Solvation Model Based on Solute Electron Density and on a Continuum Model of the Solvent Defined by the Bulk Dielectric

Constant and Atomic Surface Tensions. *J. Phys. Chem. B* **2009**, *113* (18), 6378–6396. https://doi.org/10.1021/jp810292n.

(23) Legault, C. Y., CYLview, 1.0b; Université de Sherbrooke, 2009, http://www.cylview.org.

6. NMR Spectra

1-phenyl-2-(4-tolyl)ethan-1-ol (7aa)

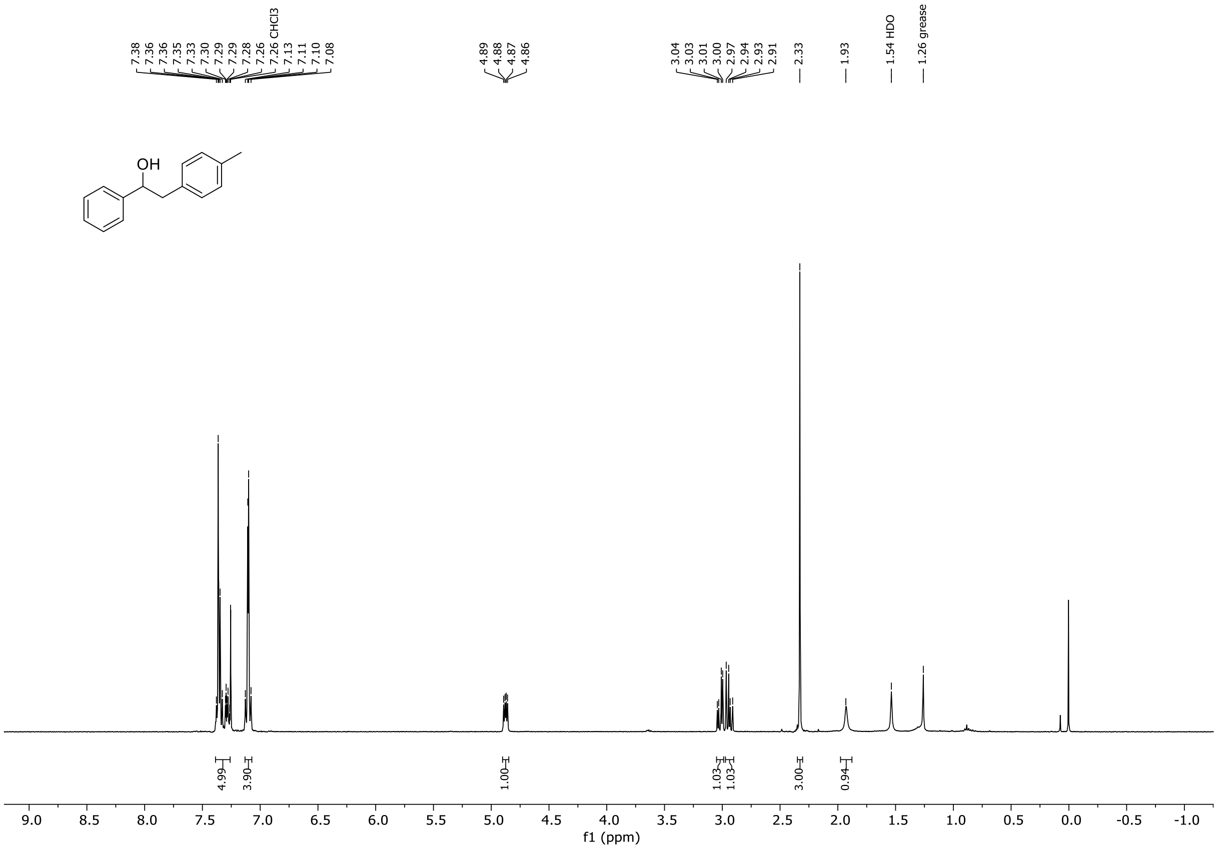

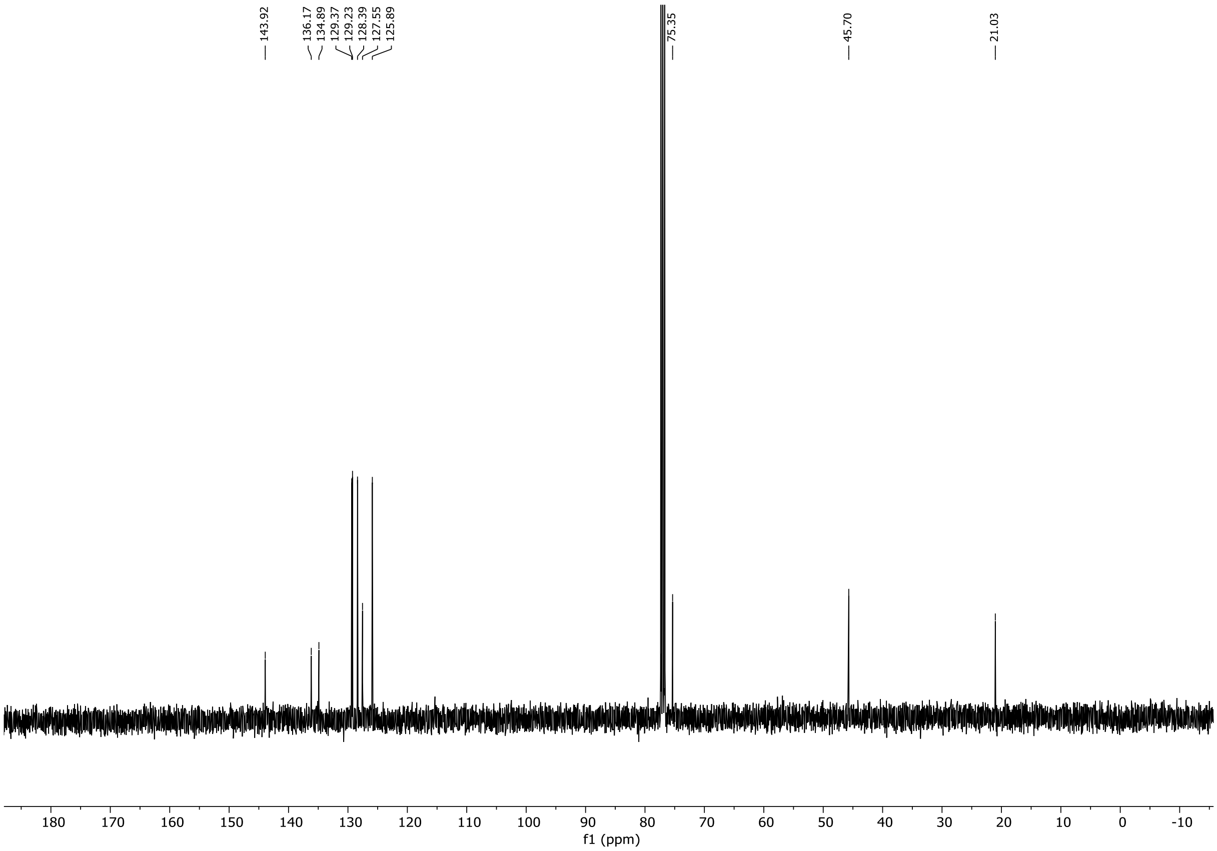

1-(4-(*tert*-butyl)phenyl)-2-(4-tolyl)ethan-1-ol (7ba)

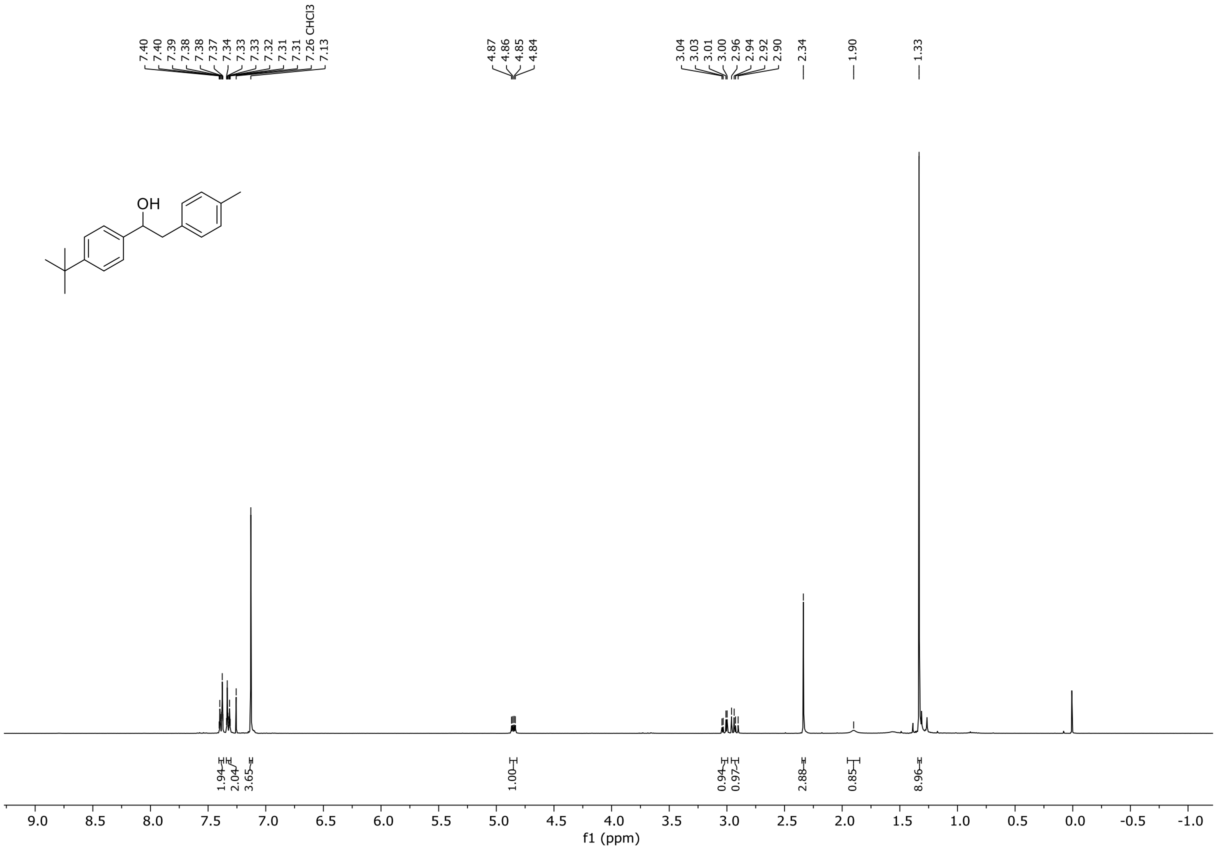

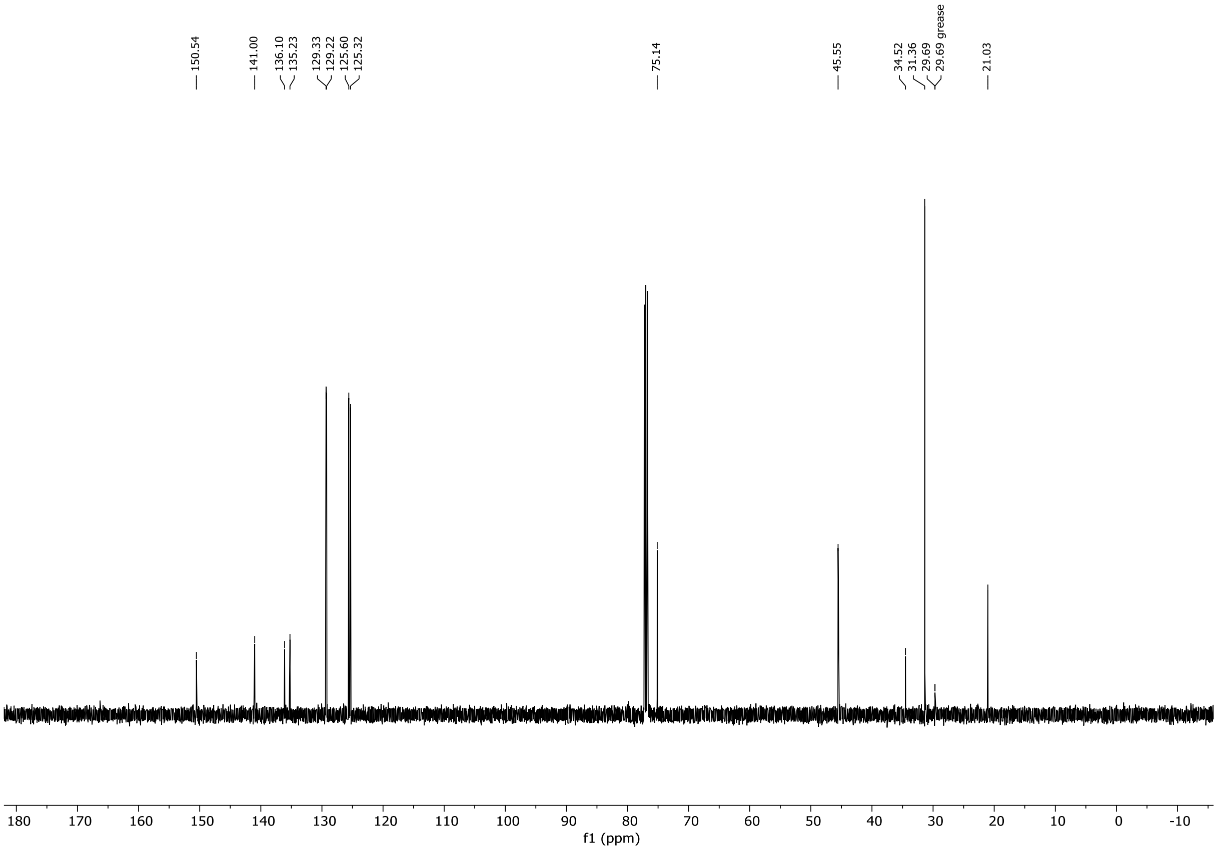

1-(4-fluorophenyl)-2-(4-tolyl)ethan-1-ol (7ca)

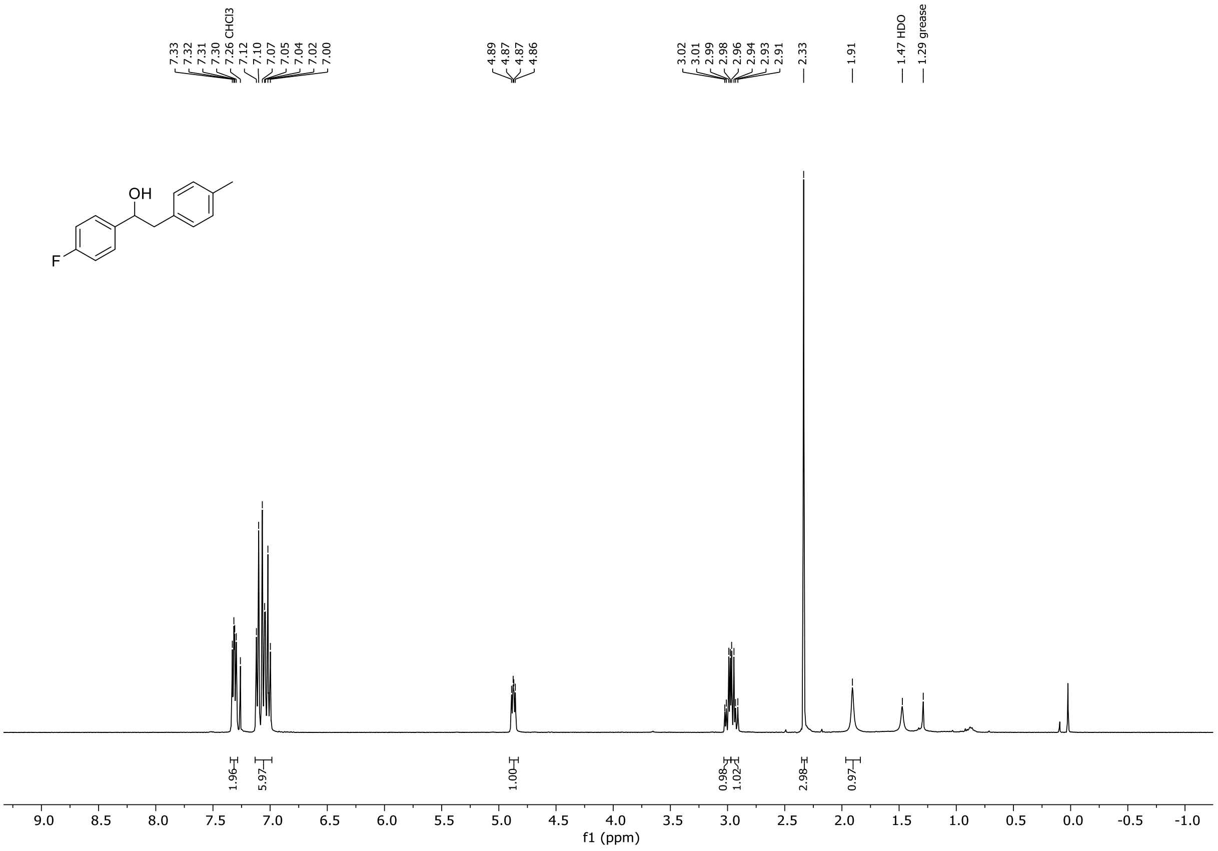

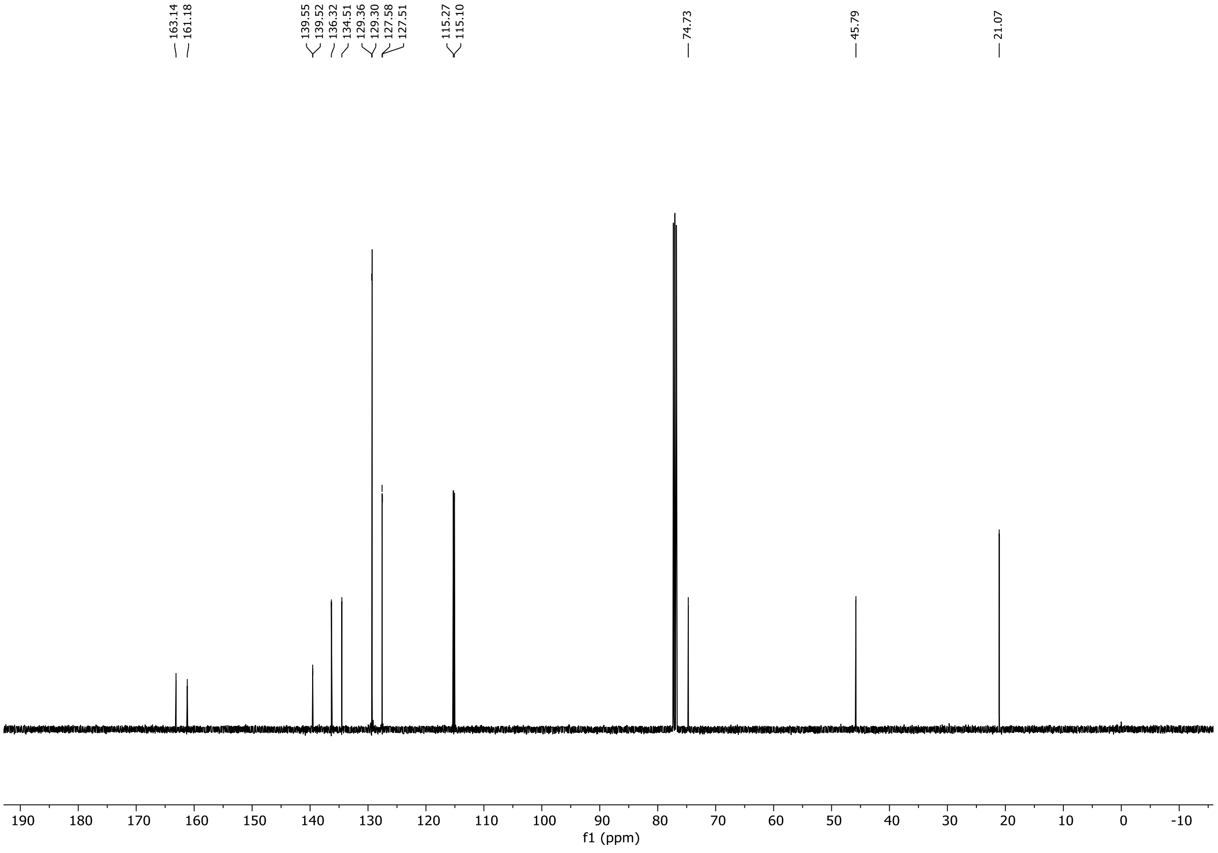

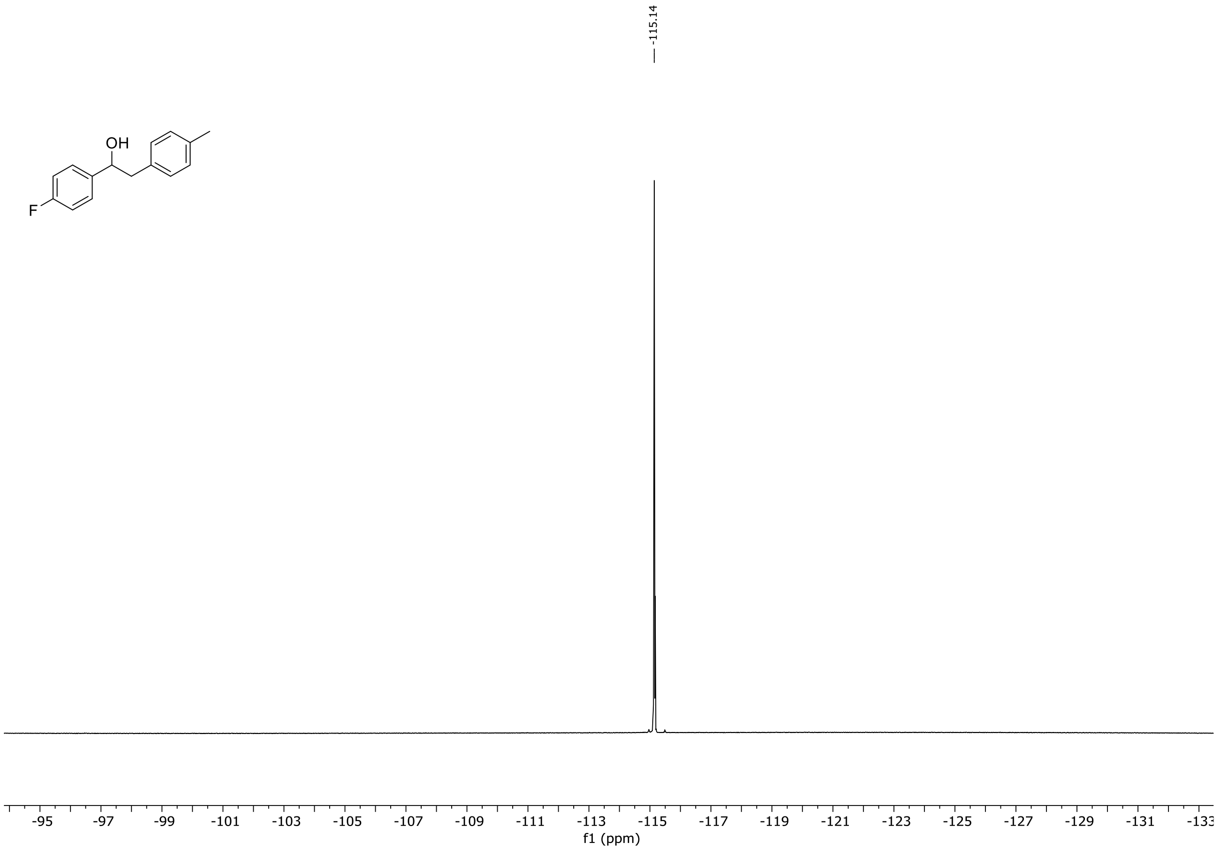
OH
F
-115.14
-95
-97
-99
-101
-103
-105
-107
-109
-111
-113
-115
-117
-119
-121
-123
-125
-127
-129
-131
-133
f1 (ppm)

4-(1-hydroxy-2-(4-tolyl)ethyl)benzonitrile (7da)

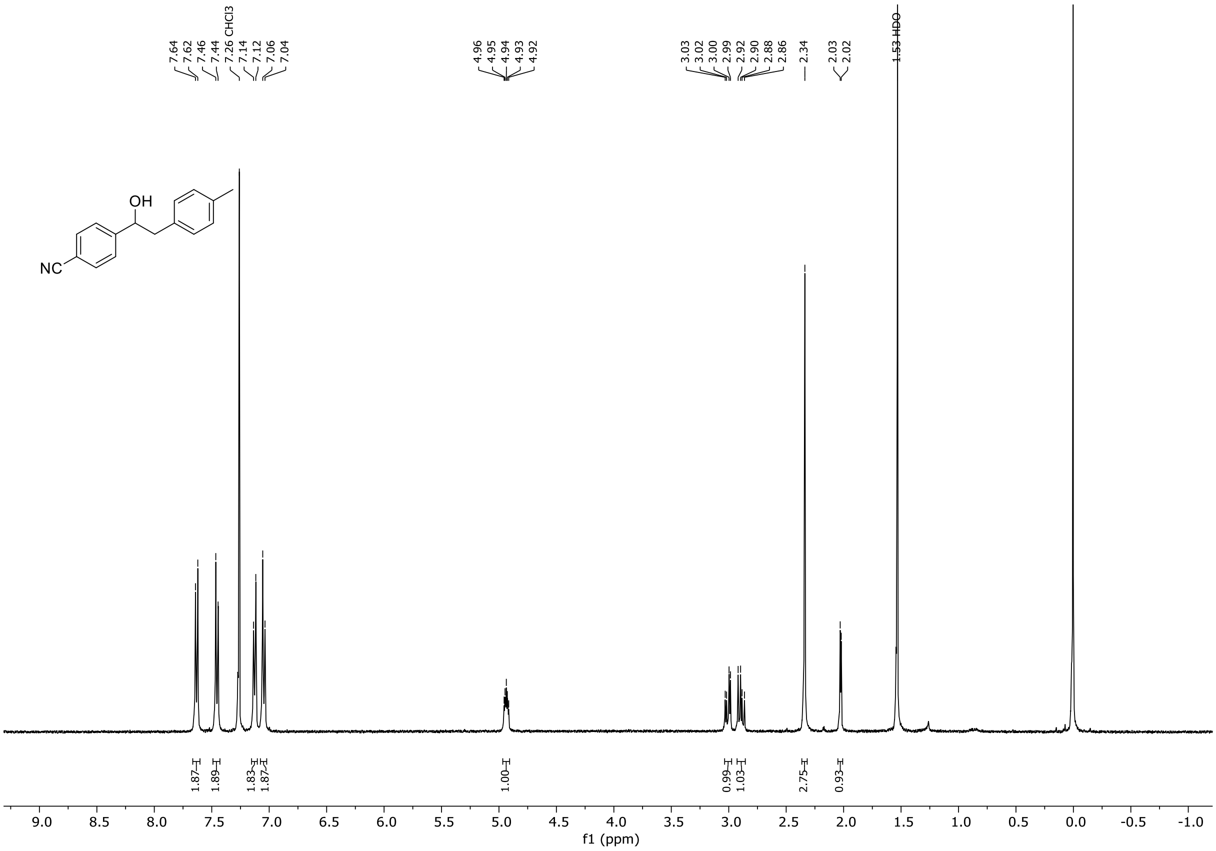

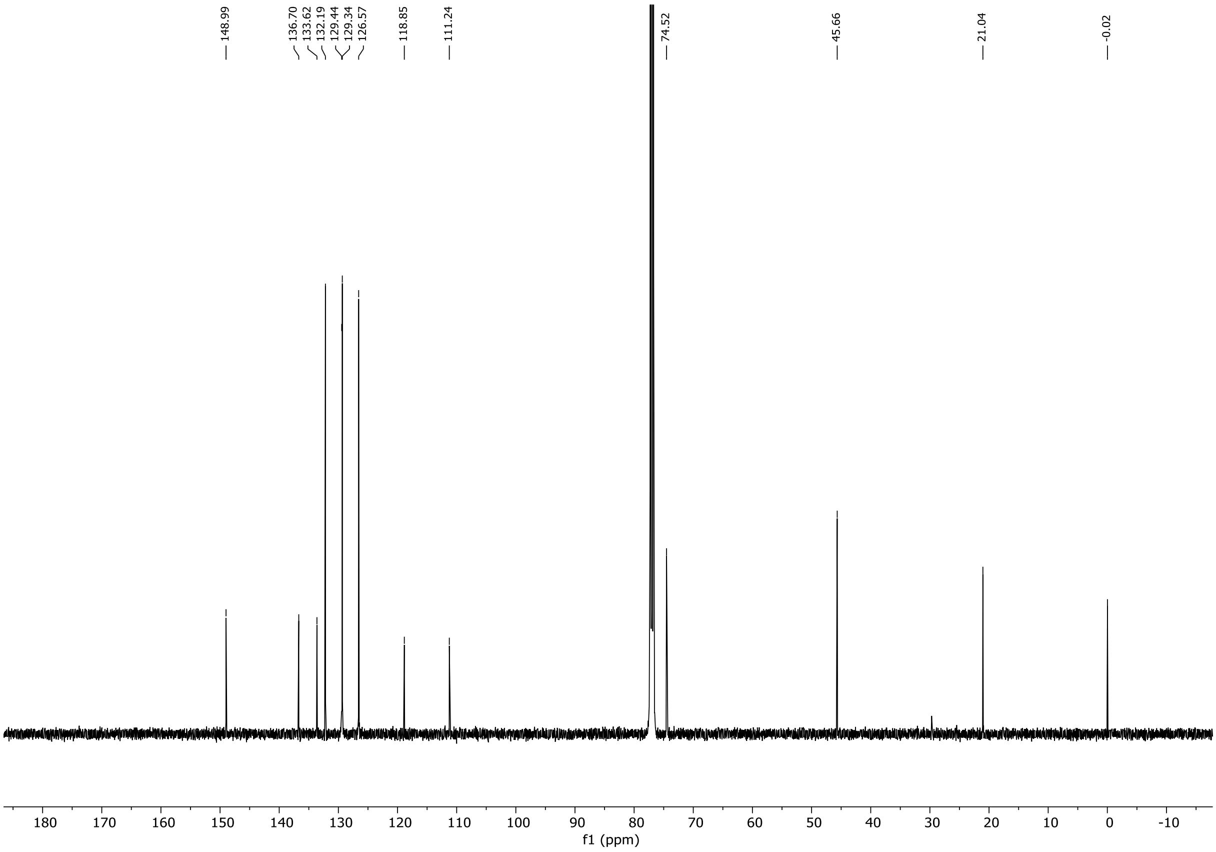

2-phenyl-1-(4-tolyl)propan-2-ol (7fa)

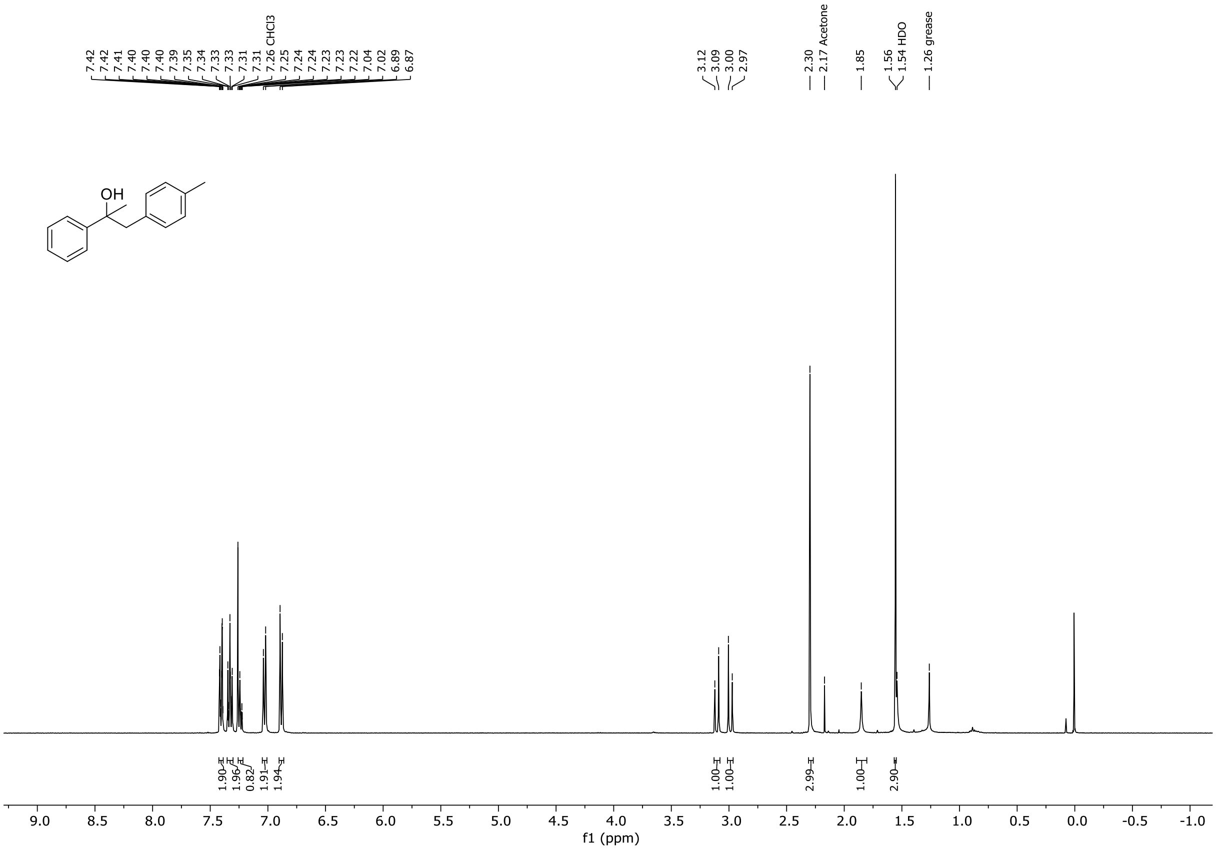

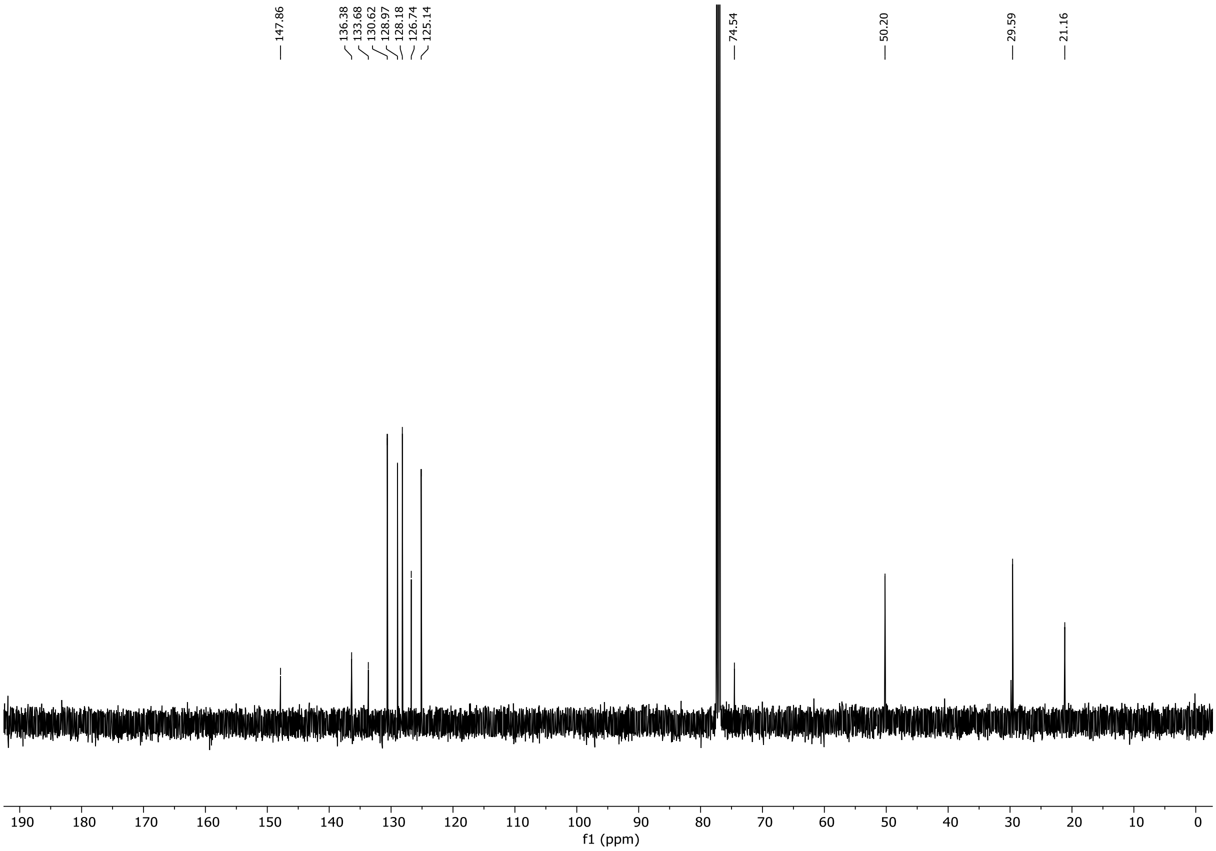

1-phenyl-2-(3-tolyl)ethan-1-ol (7ab)

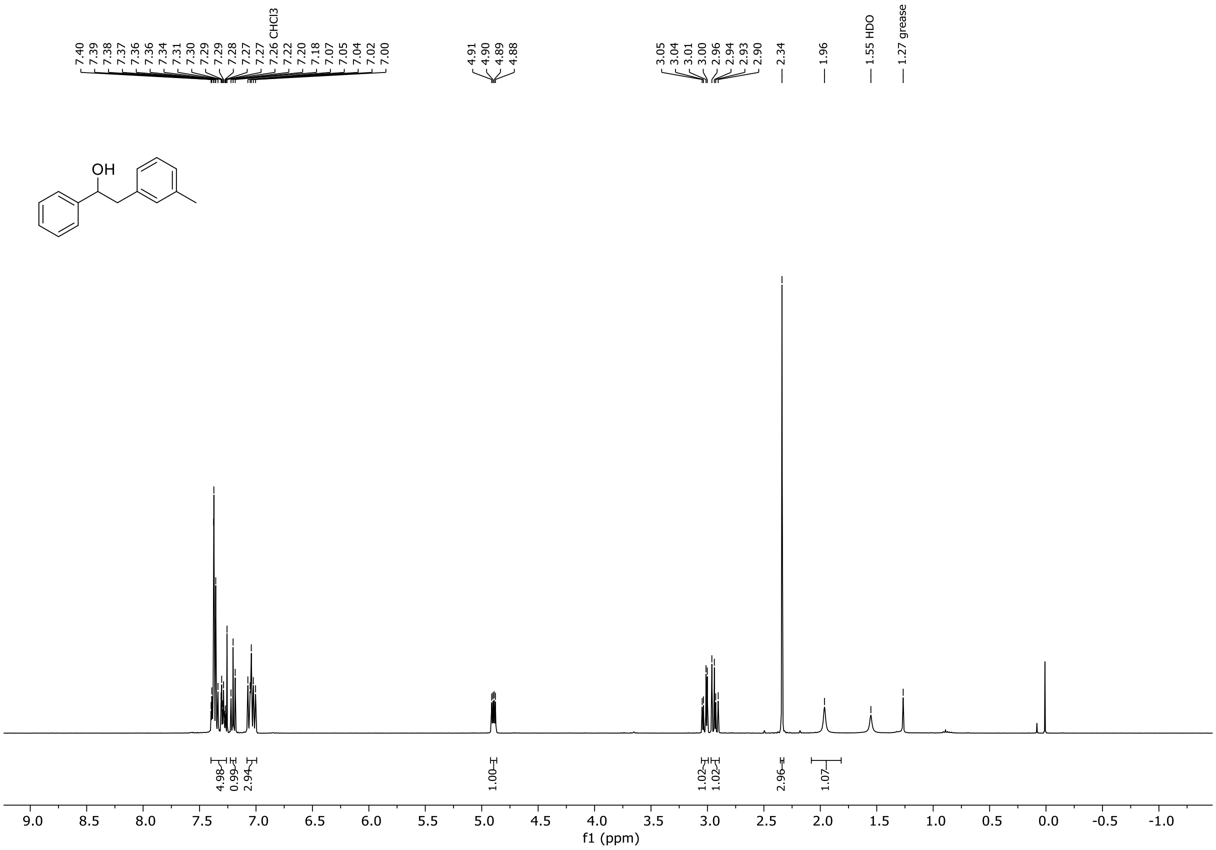

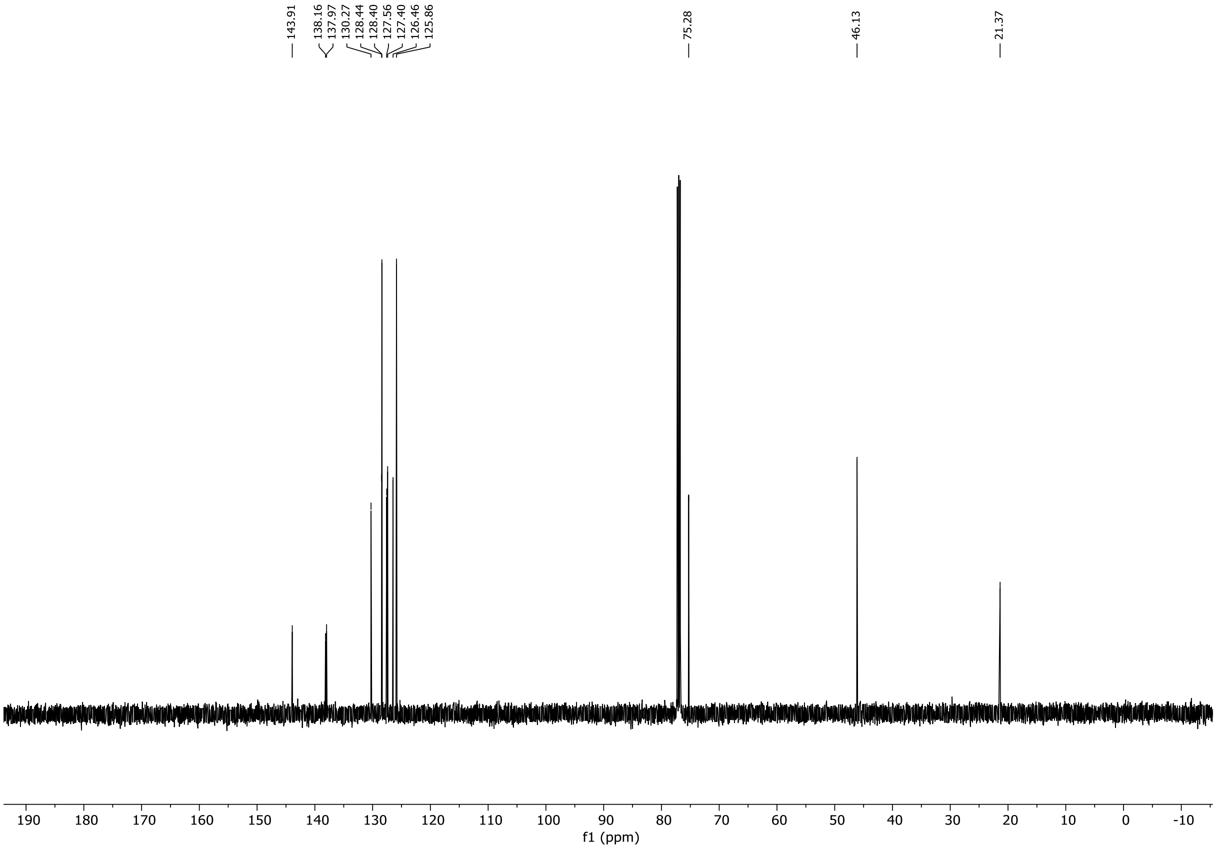

1-phenyl-2-(2-tolyl)ethan-1-ol (7ac)

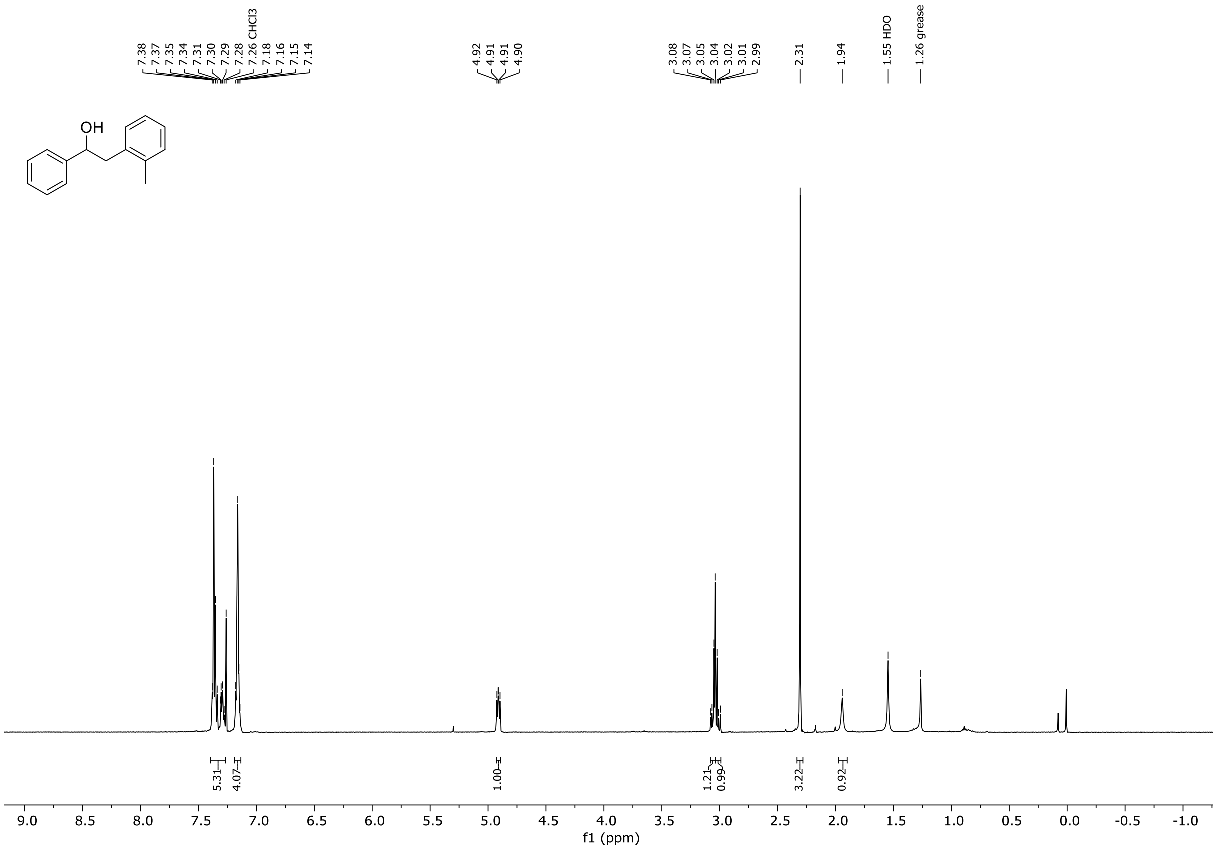

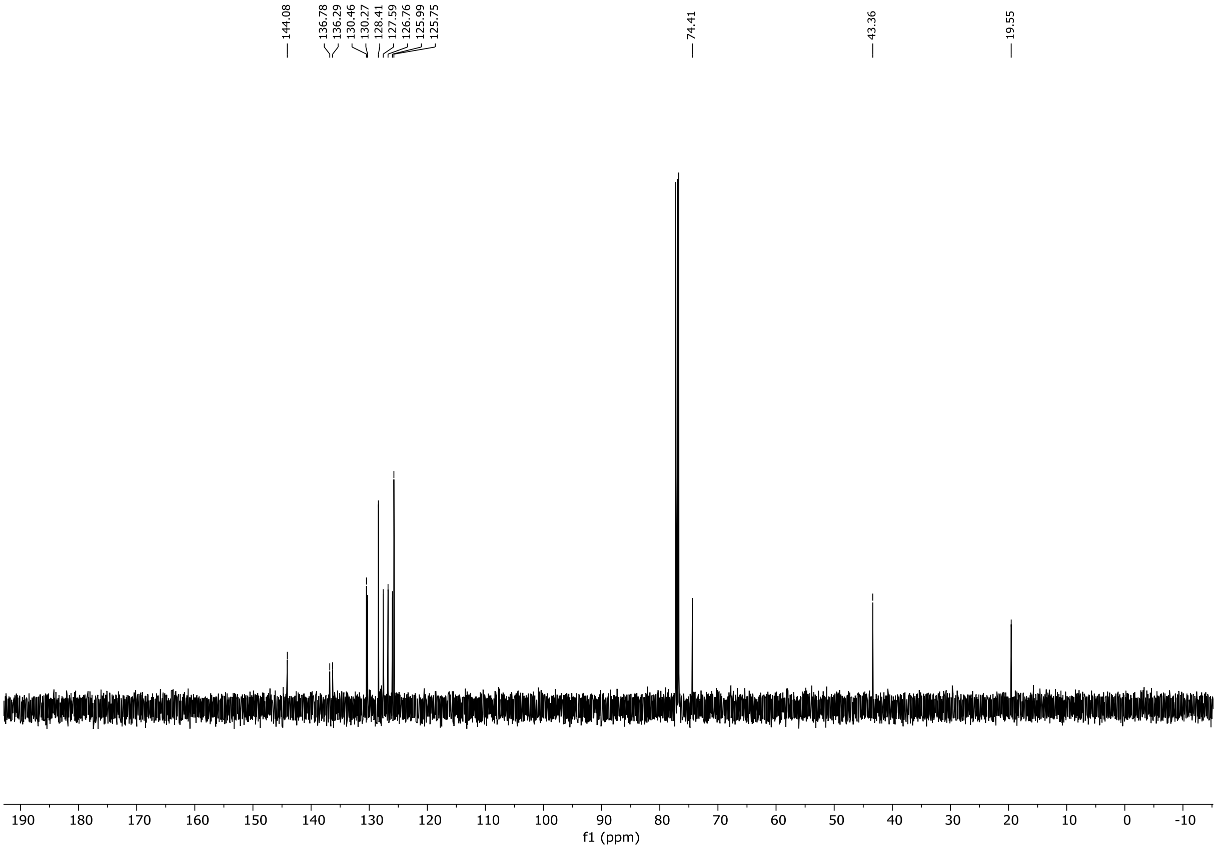

2-(4-chlorophenyl)-1-phenylethan-1-ol (7ad)

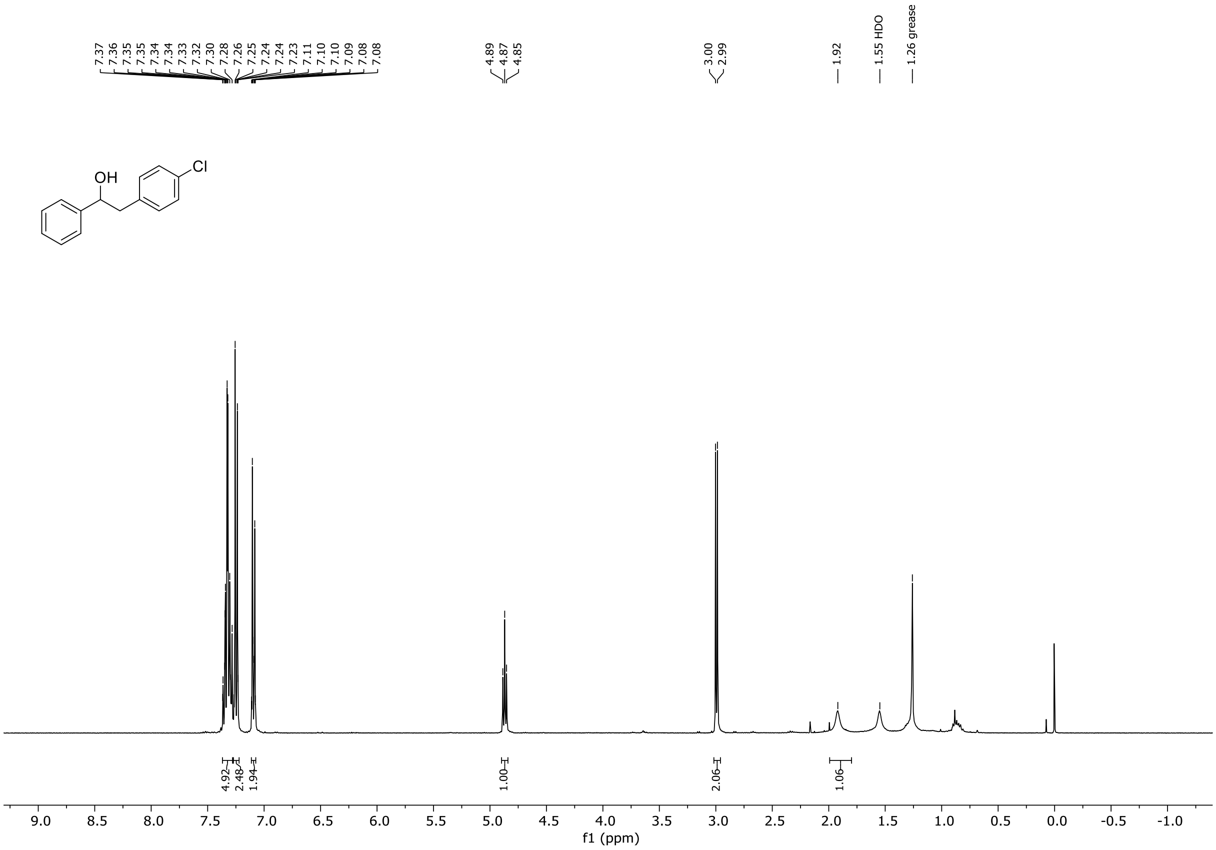

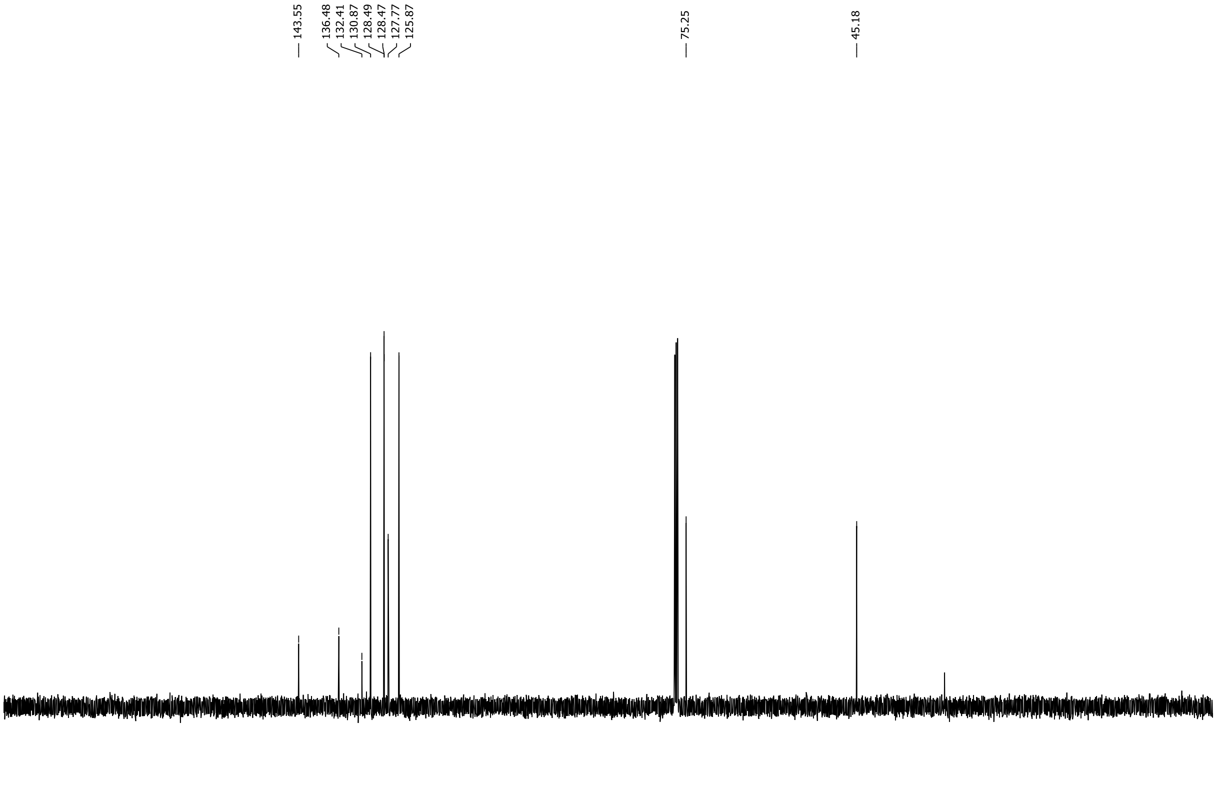

2-(benzo[*d*][1,3]dioxol-5-yl)-1-phenylethan-1-ol (7ae)

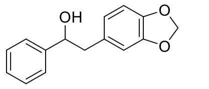

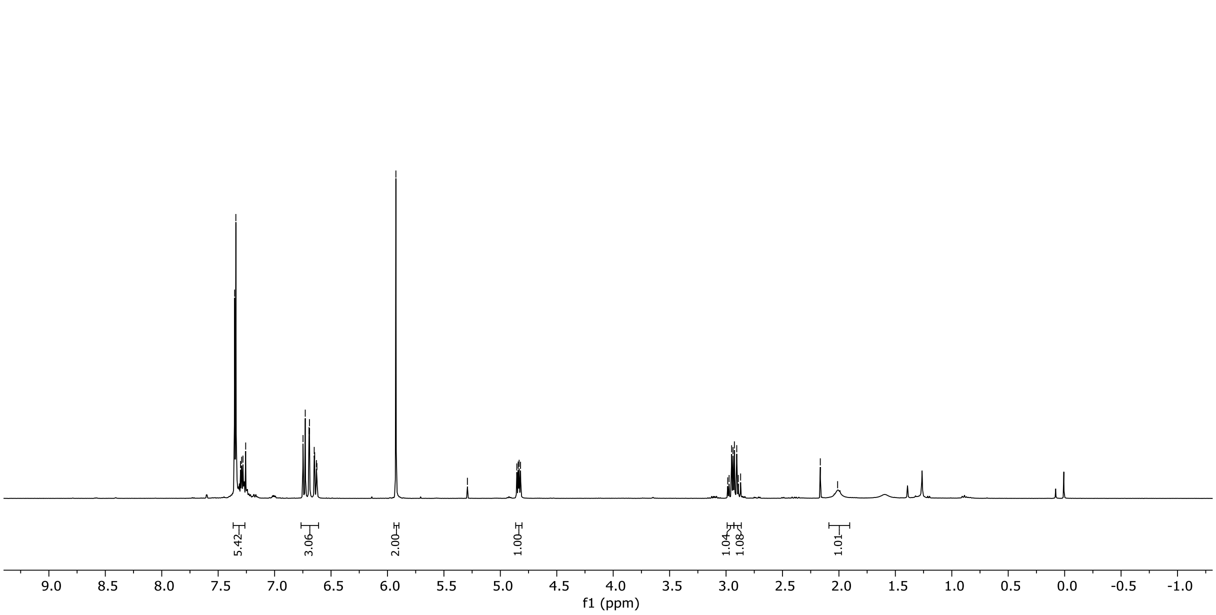

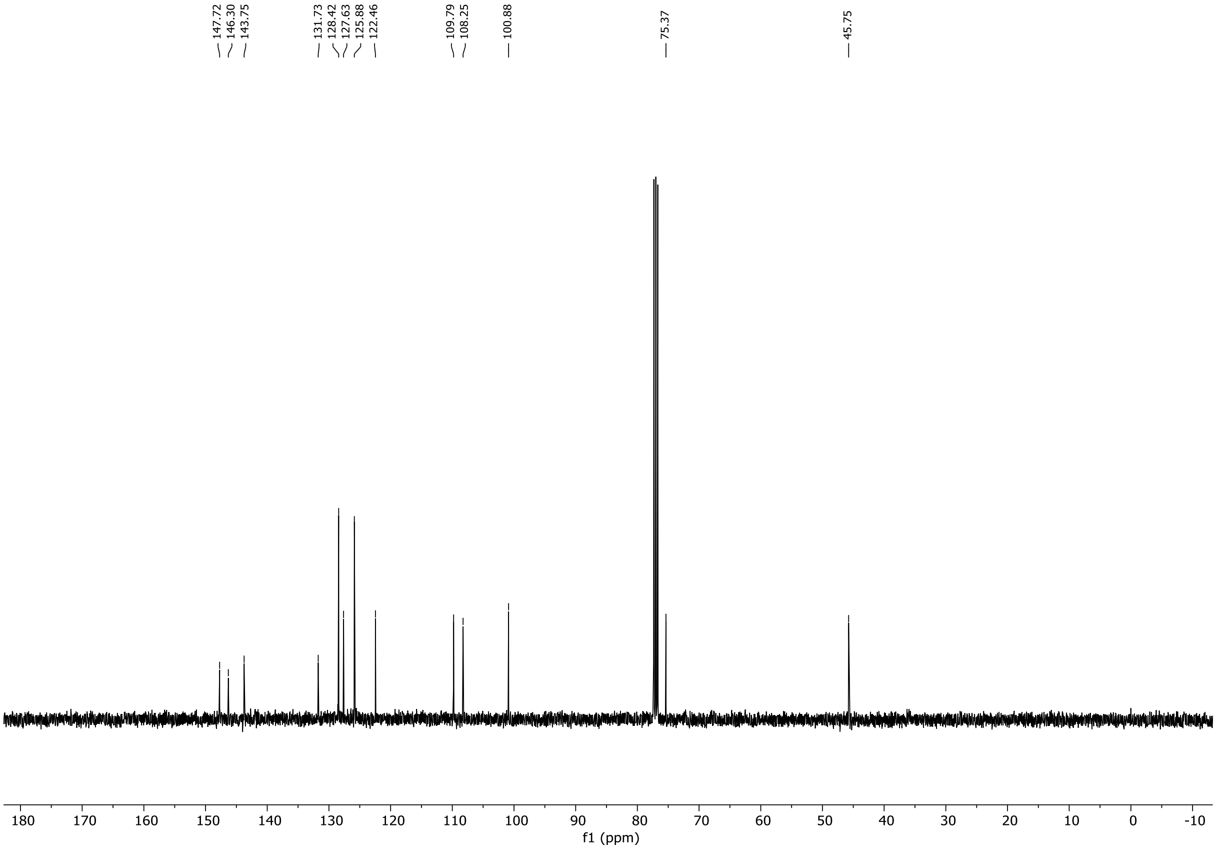

1-phenyl-2-(4-(trifluoromethyl)phenyl)ethan-1-ol (7af)

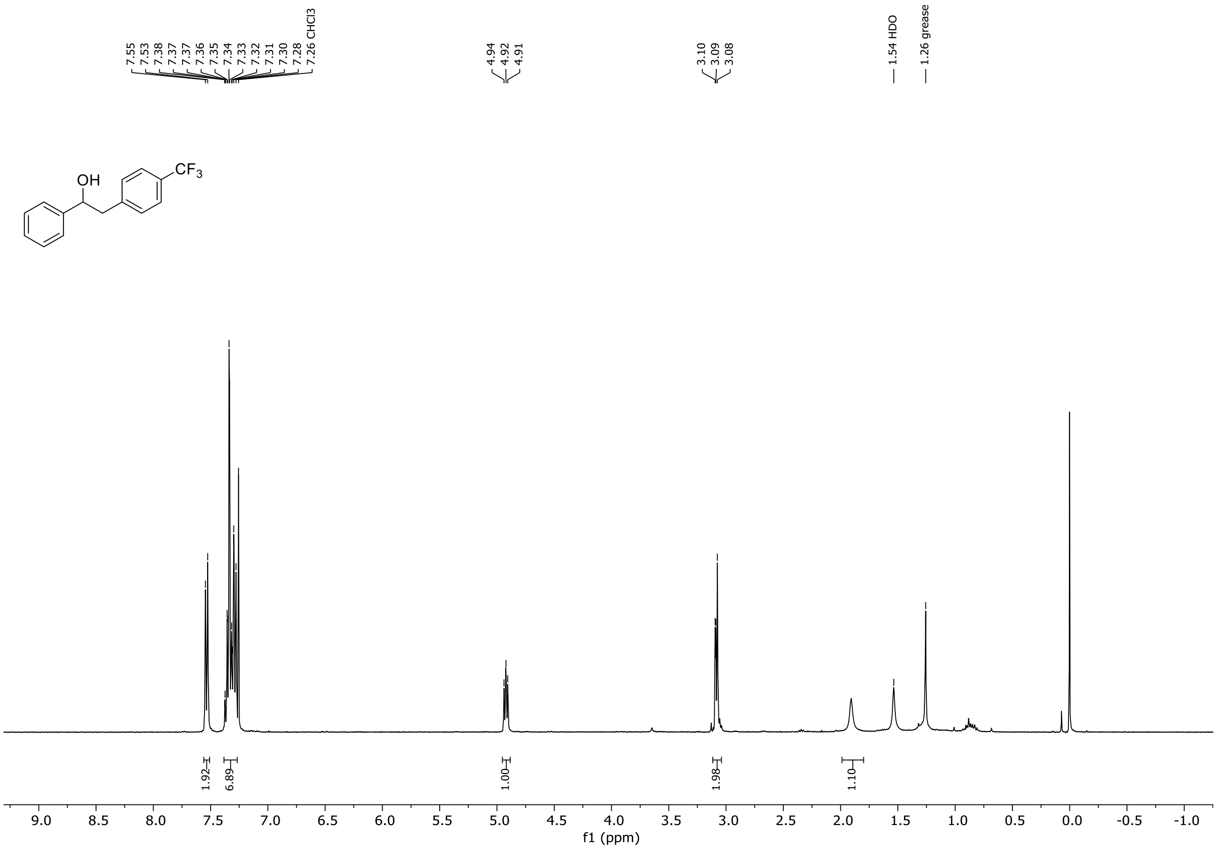

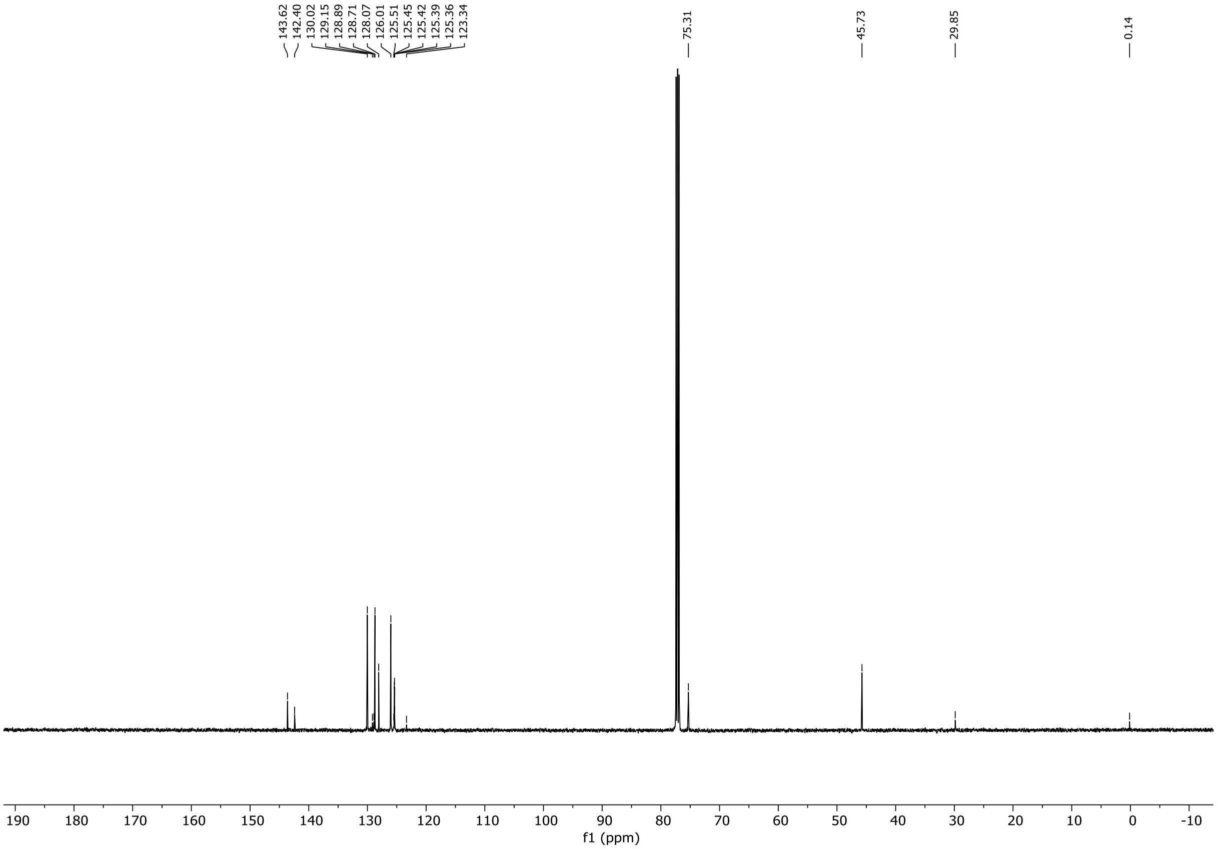

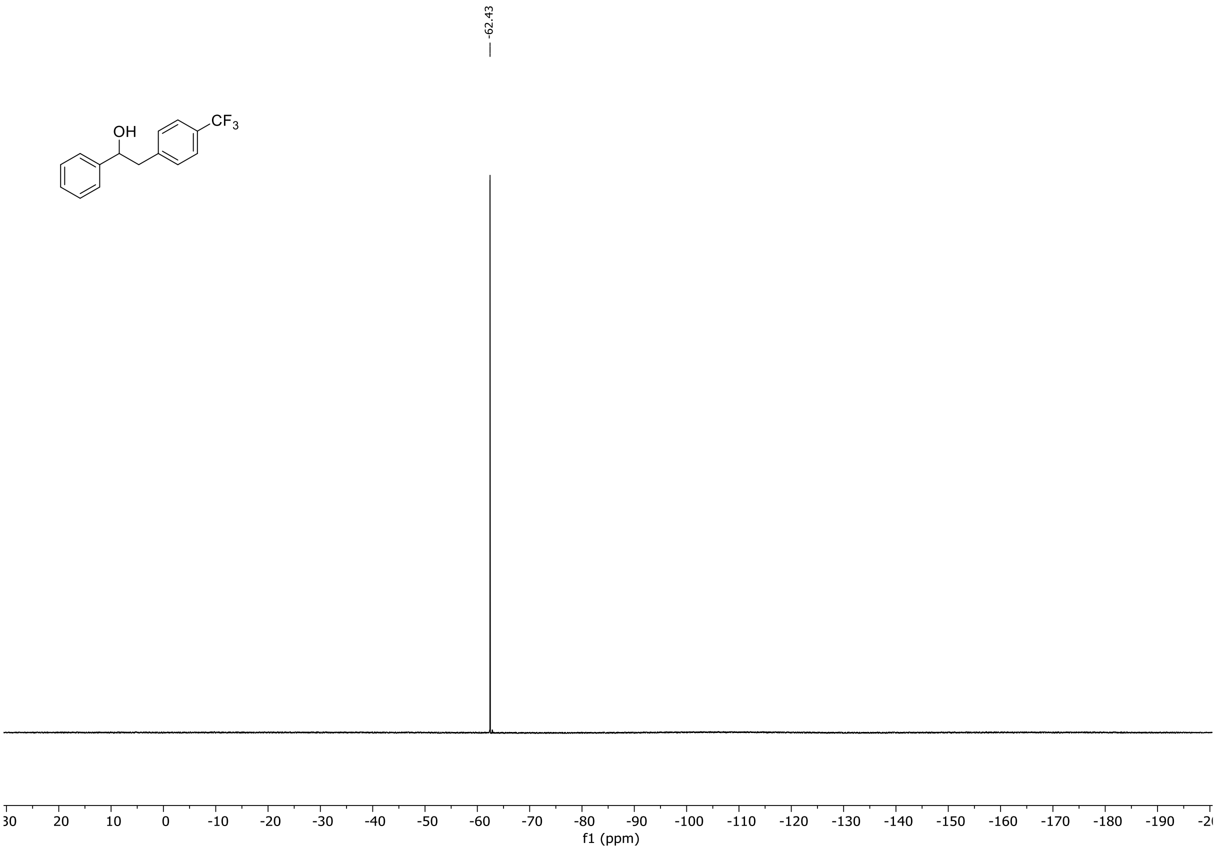
-62.43
OH
CF3
30
20
10
0
-10
-20
-30
-40
-50
-60
-70
-80
-90
-100
-110
-120
-130
-140
-150
-160
-170
-180
-190
f1 (ppm)

1-(4-(2-hydroxy-2-phenylethyl)phenyl)ethan-1-one (7ag)

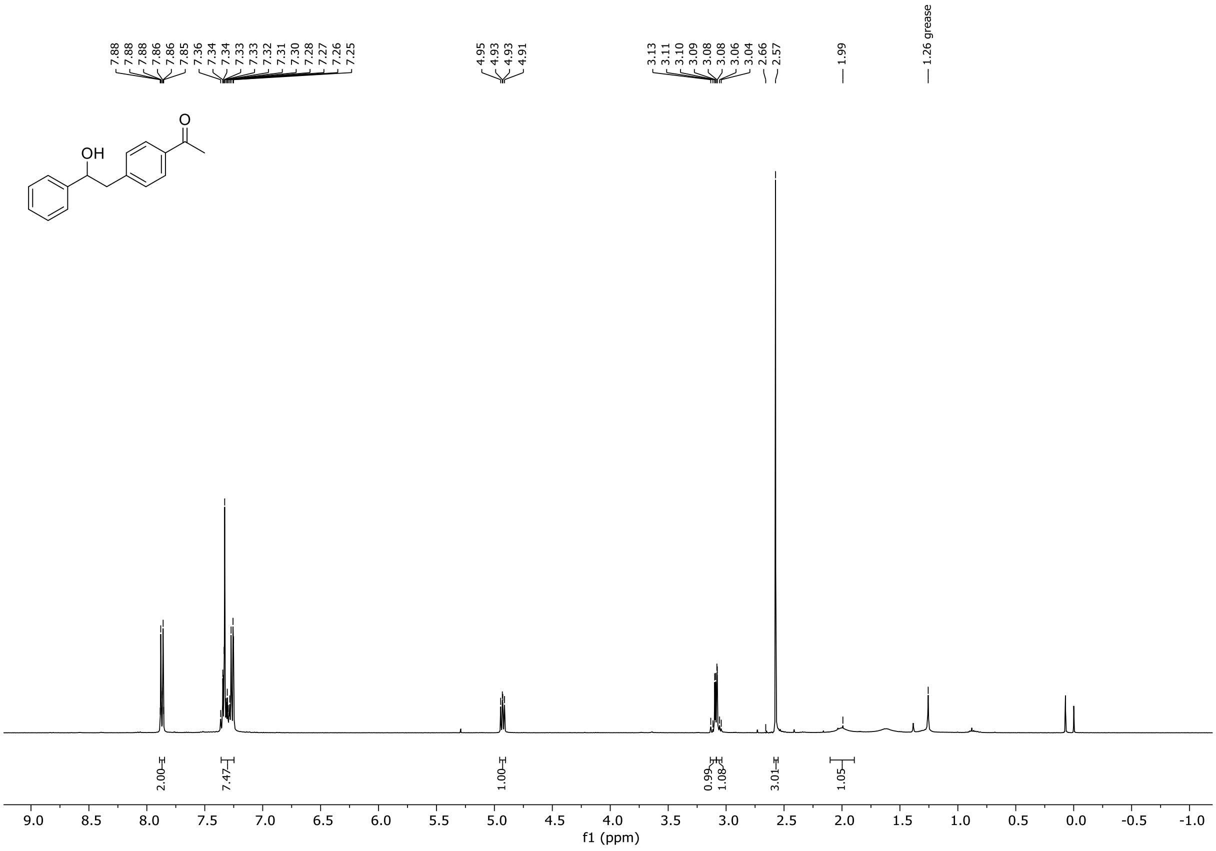

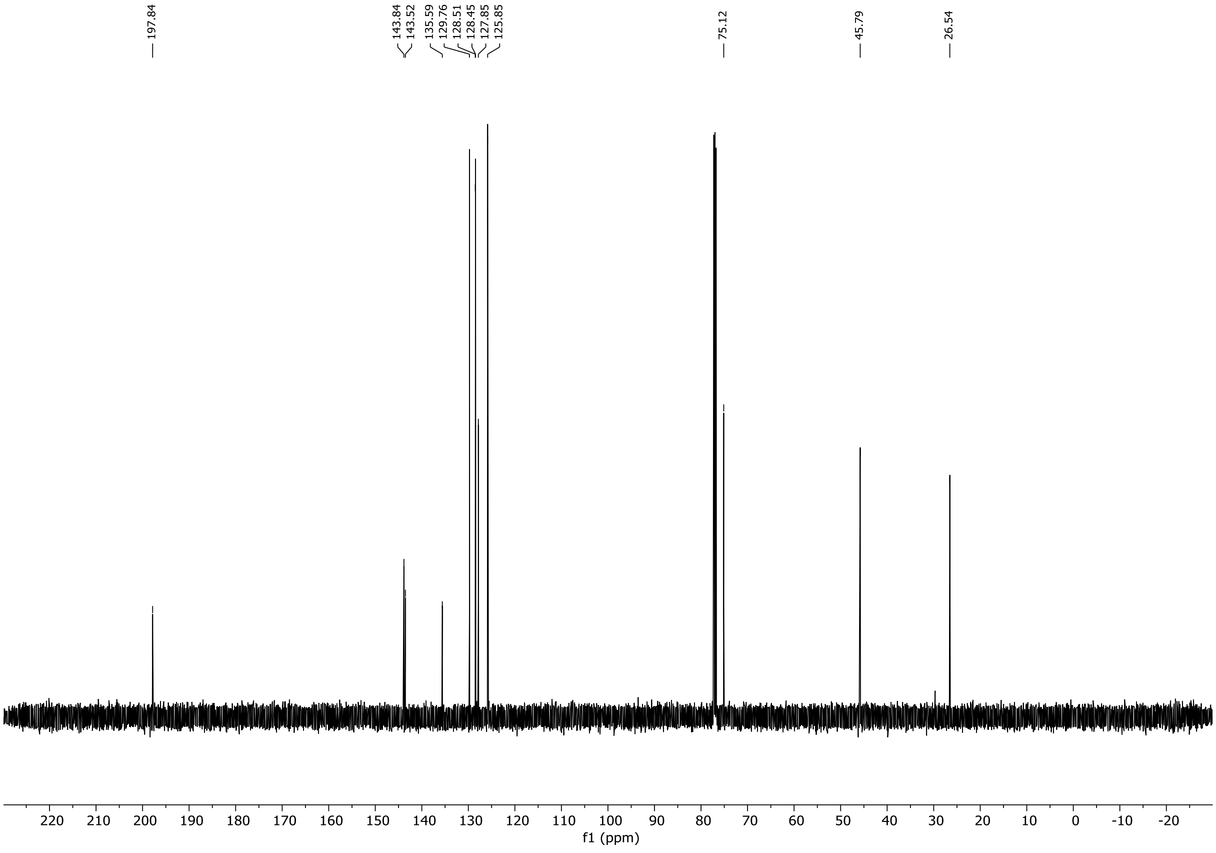

2-(4-methoxyphenyl)-1-phenylethan-1-ol (7ah)

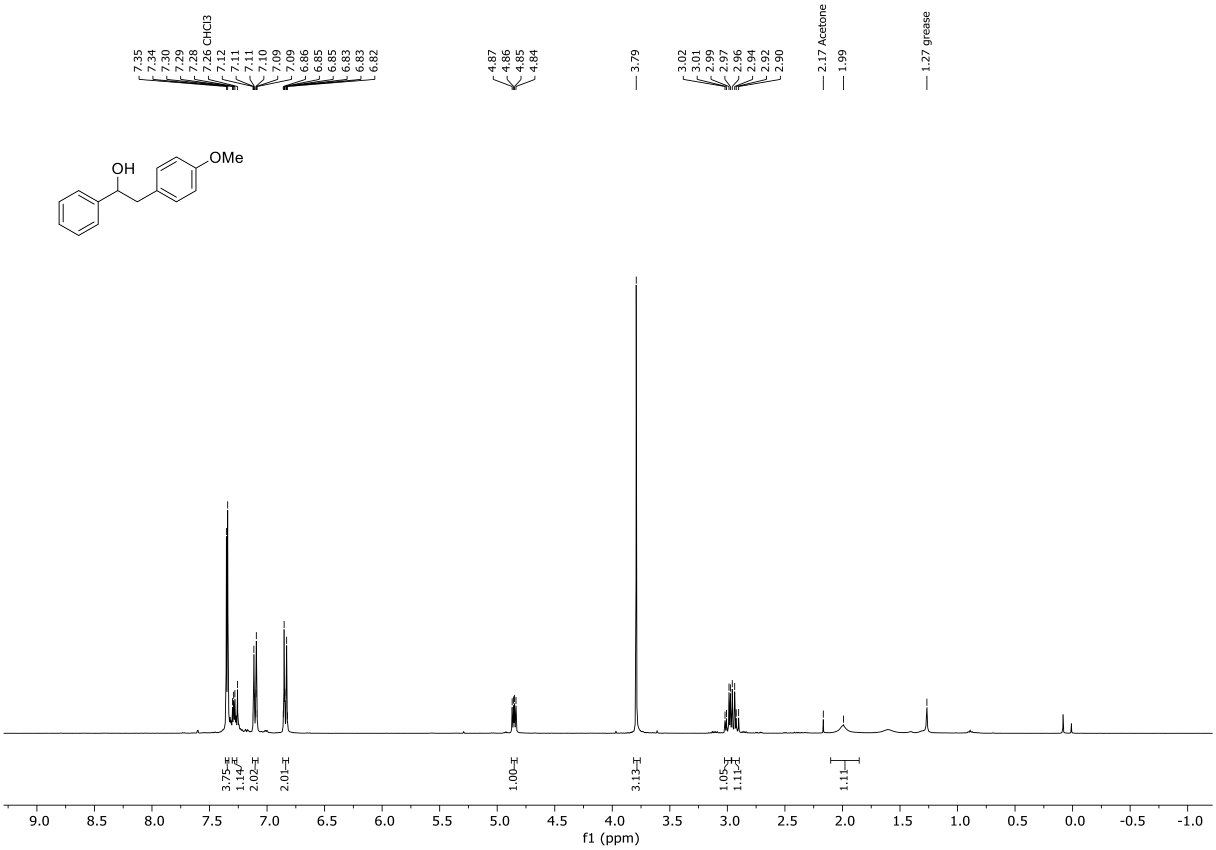

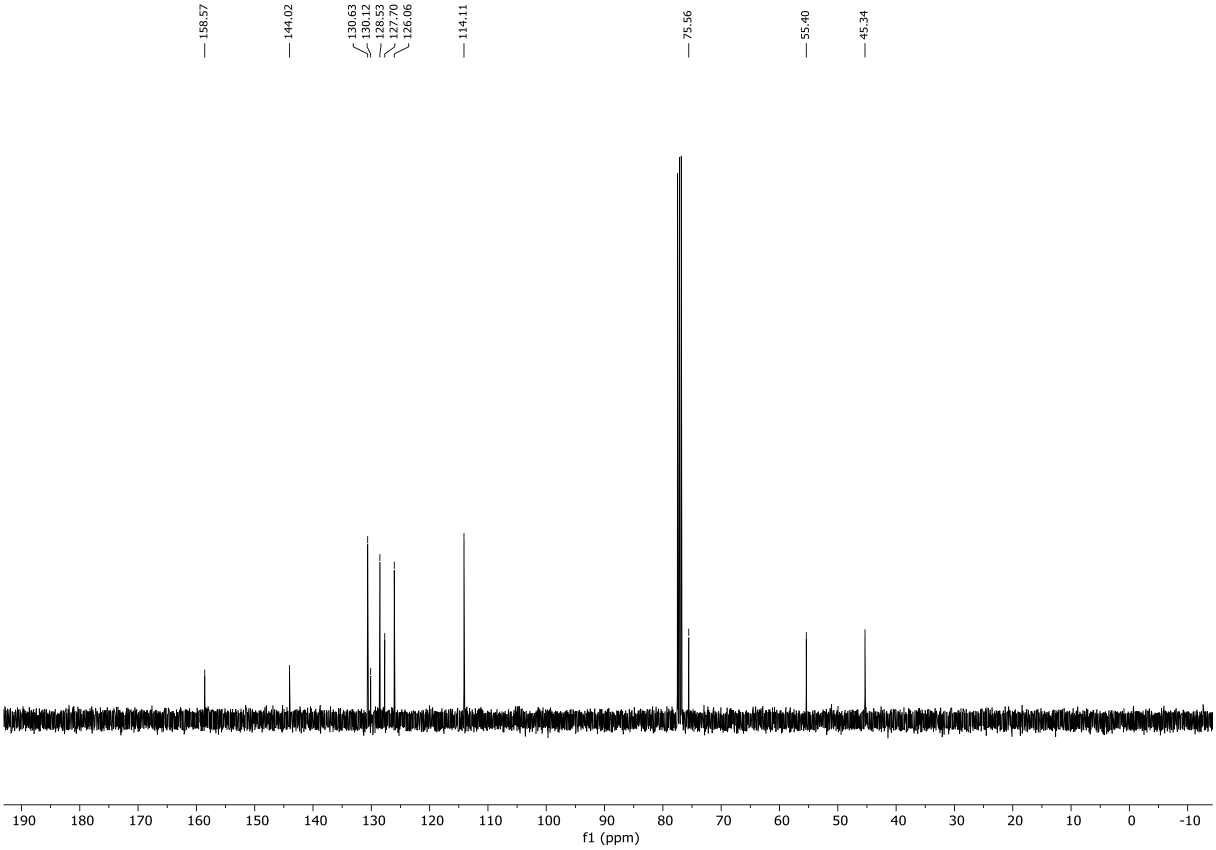

4-(2-hydroxy-2-phenylethyl)benzonitrile (7ai)

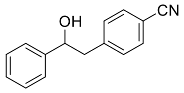

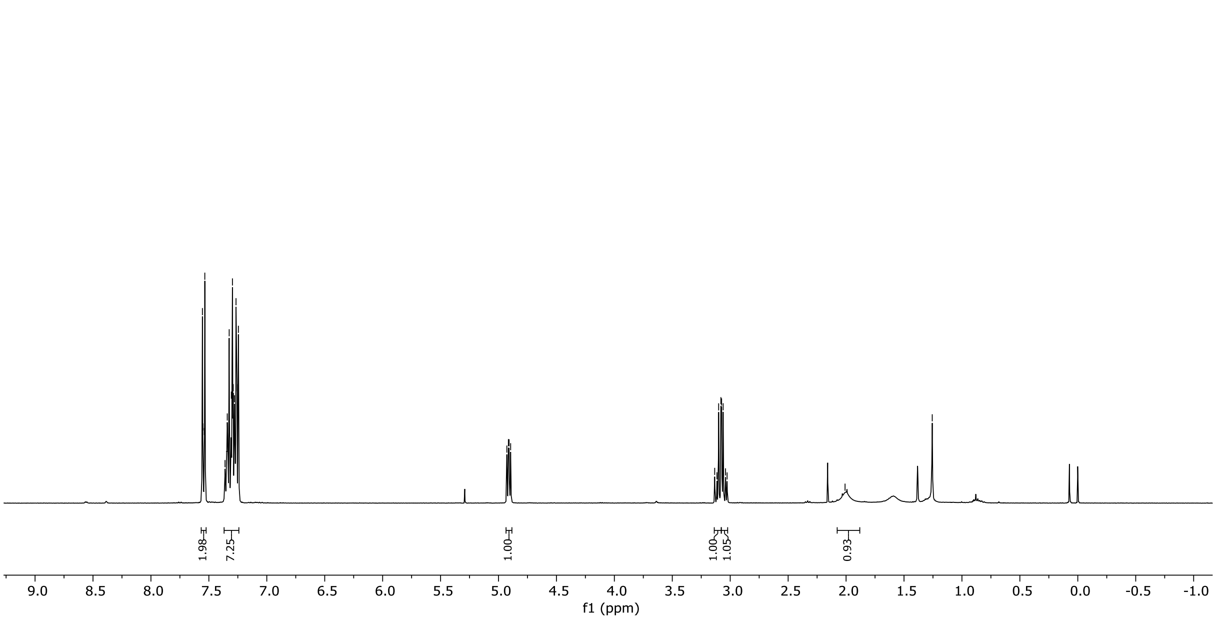

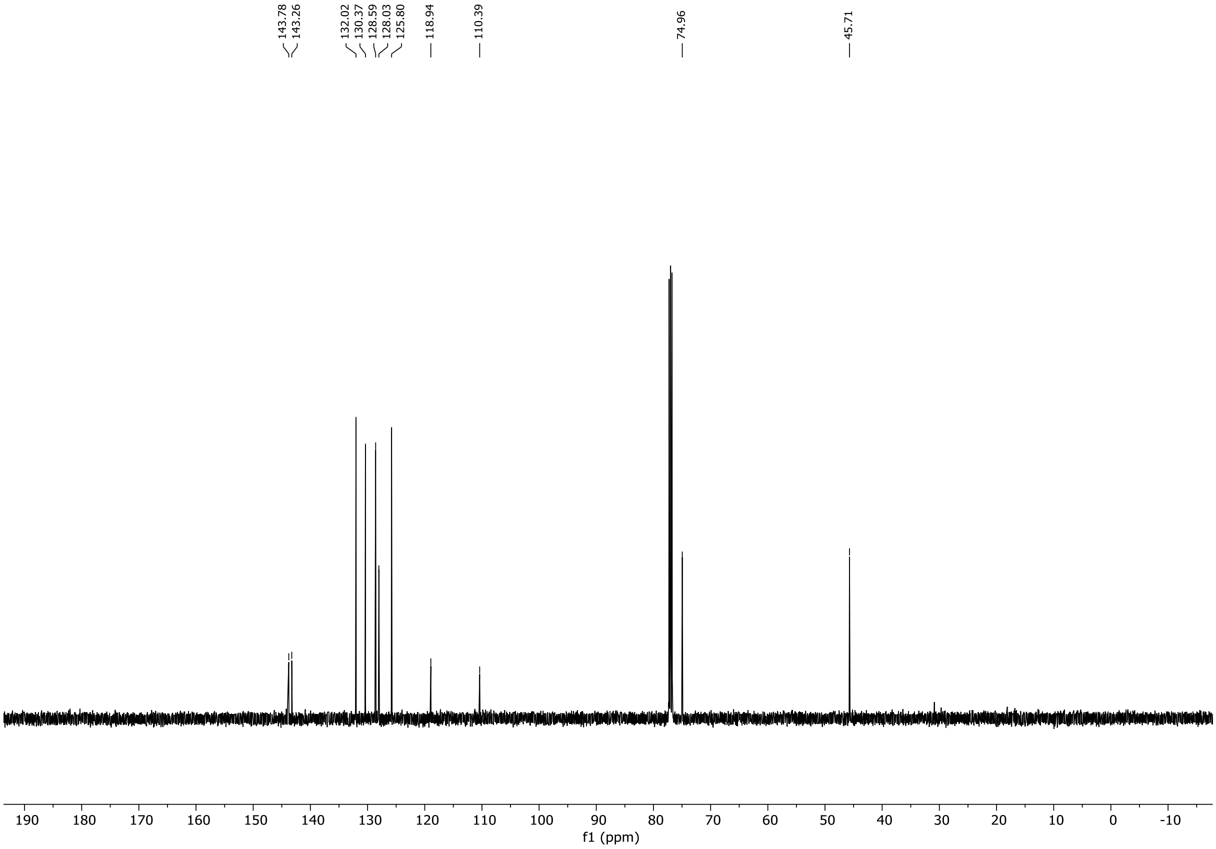

1,2-diphenylethan-1-ol (7aj)

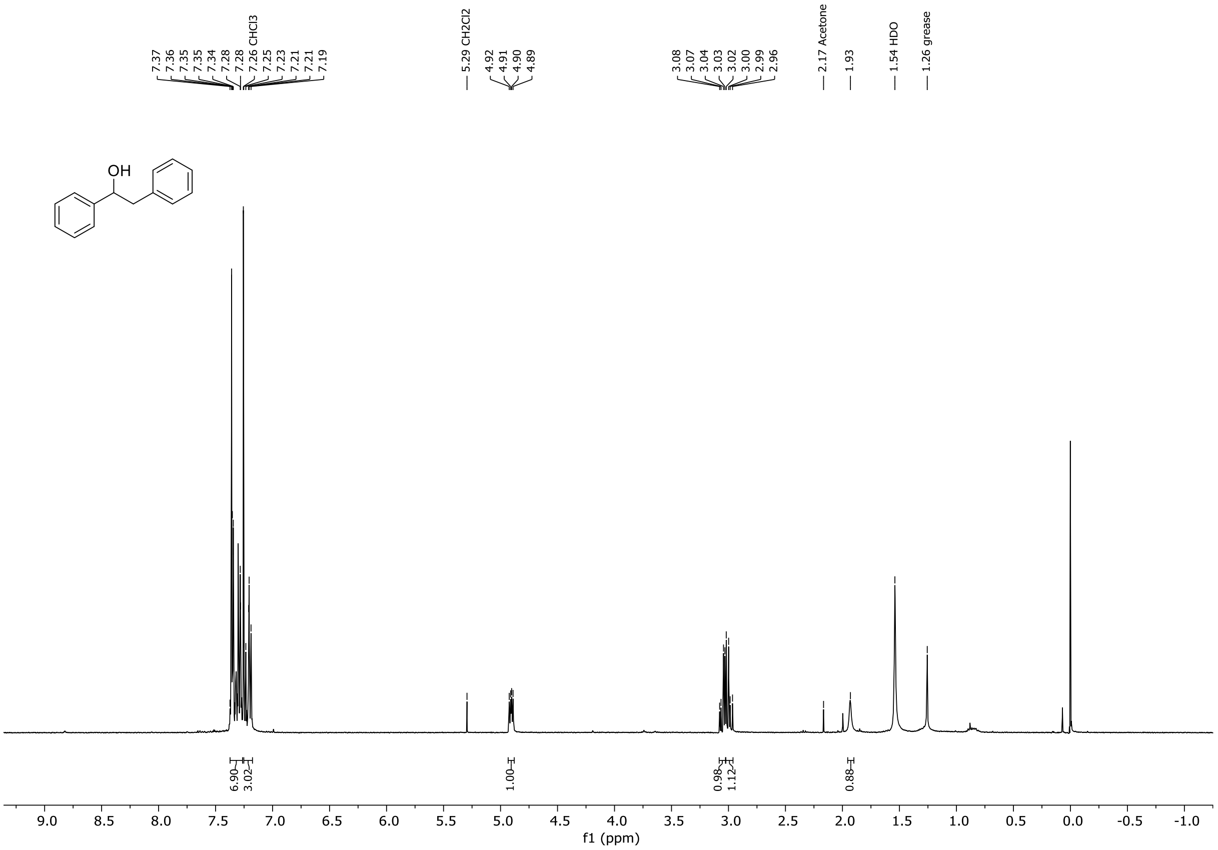

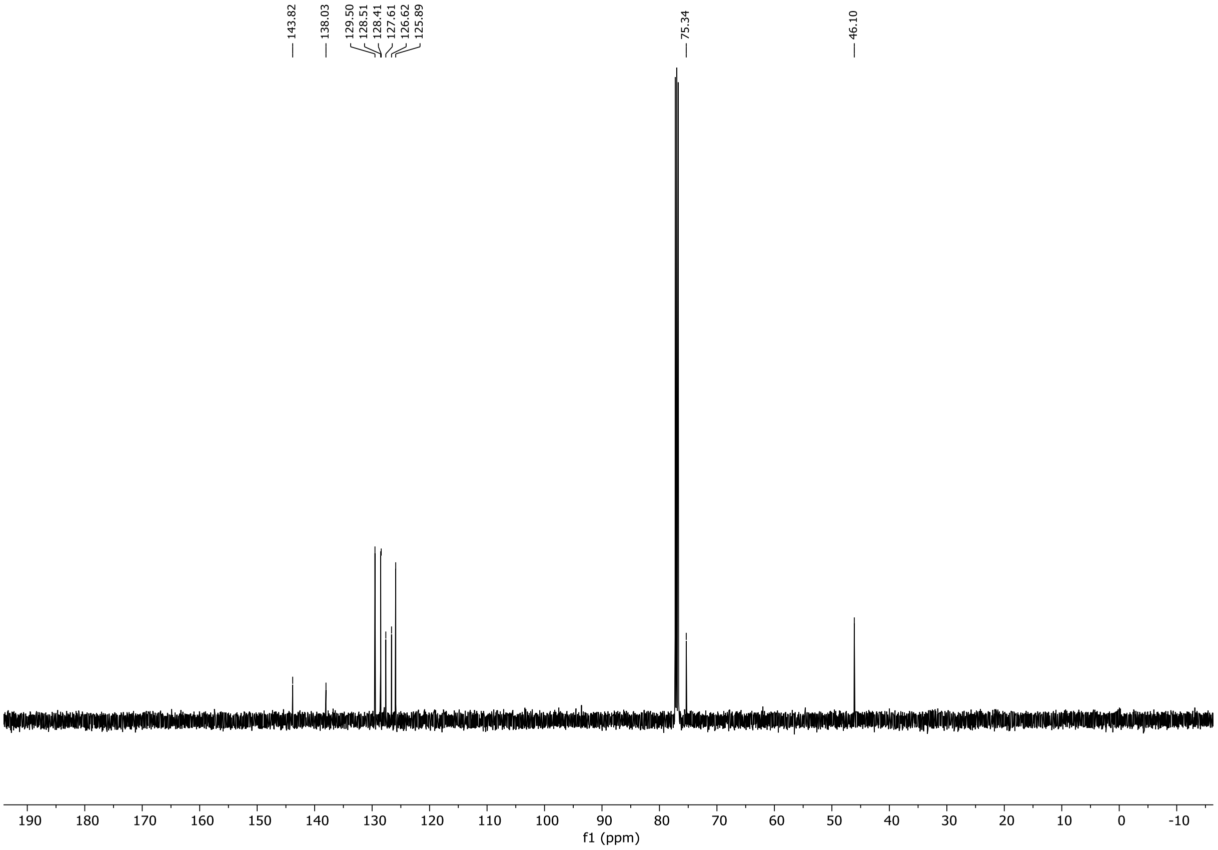

2-(naphthalen-1-yl)-1-phenylethan-1-ol (7ak)

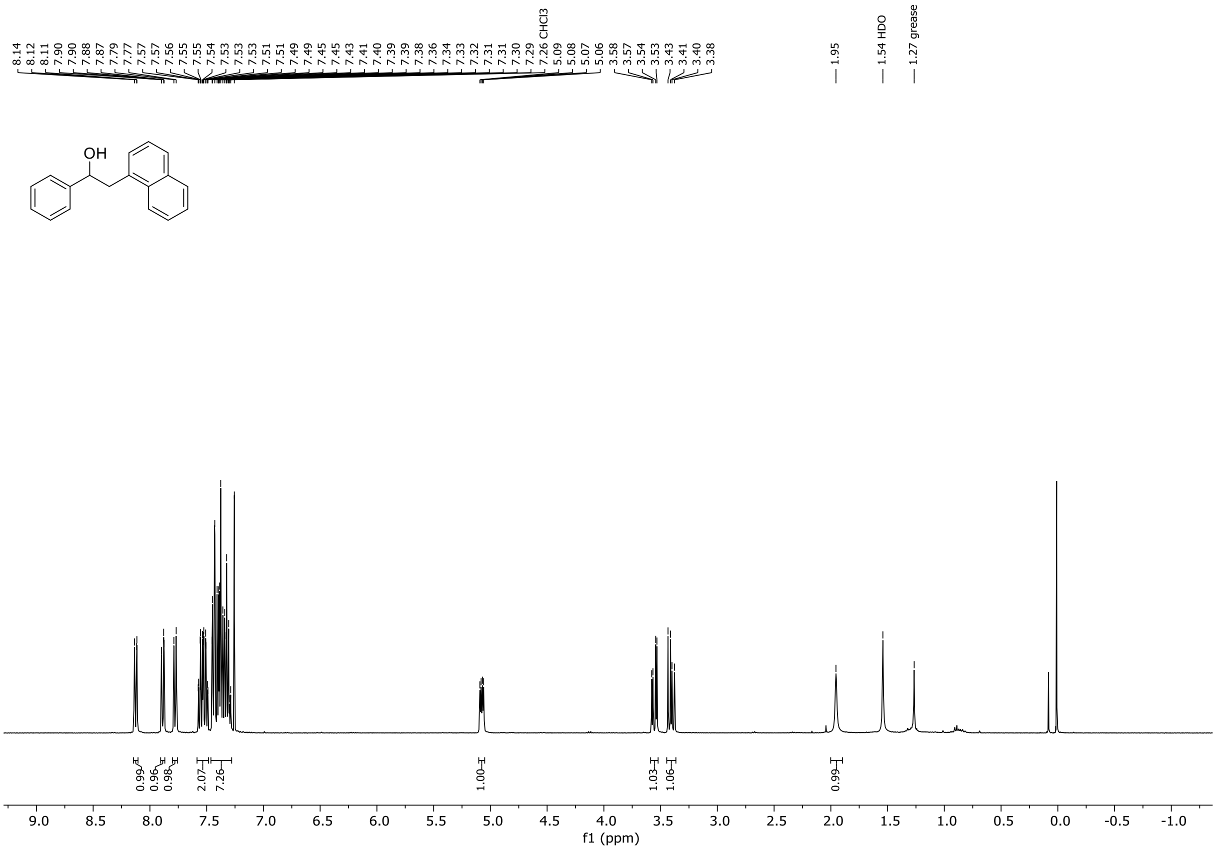

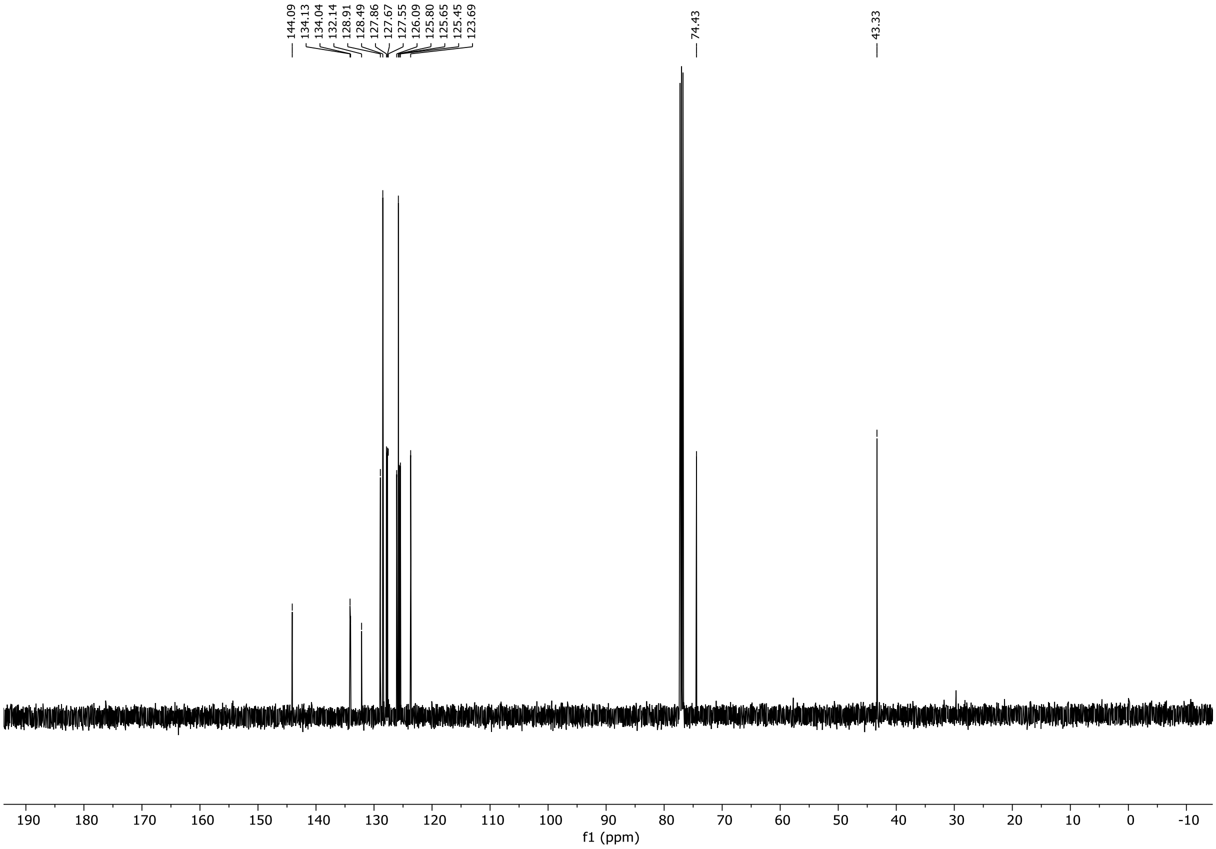

1-phenyl-2-(1-tosyl-1*H*-indol-5-yl)ethan-1-ol (7al)

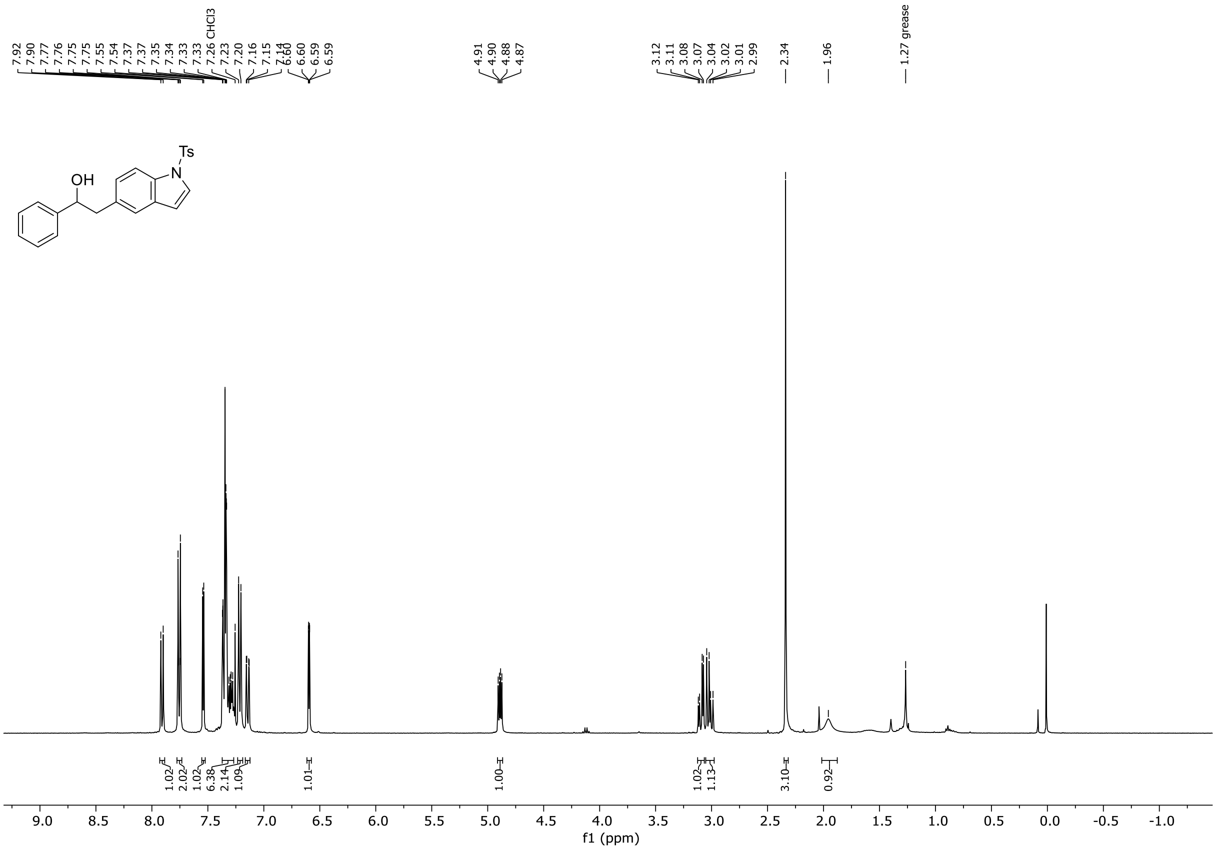

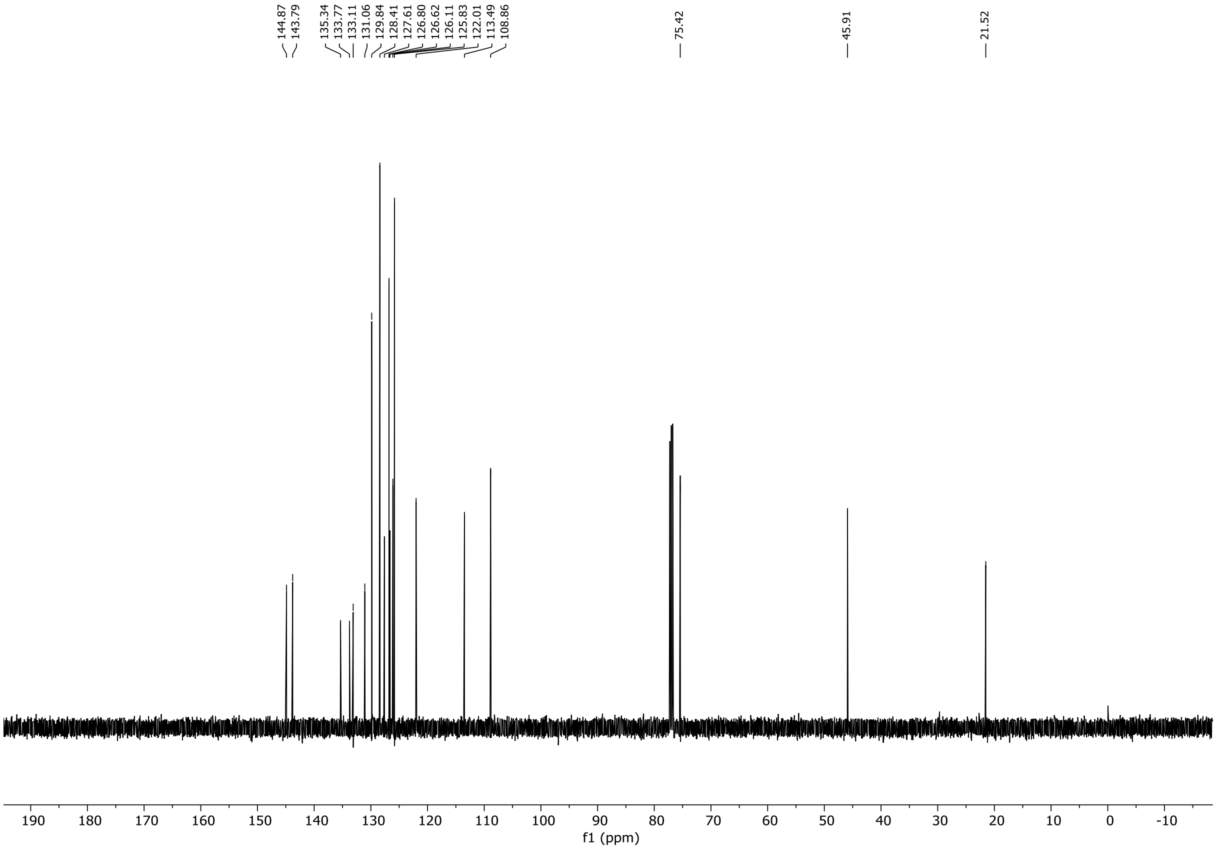

4-(2-hydroxy-2-phenylethyl)benzamide (7am)

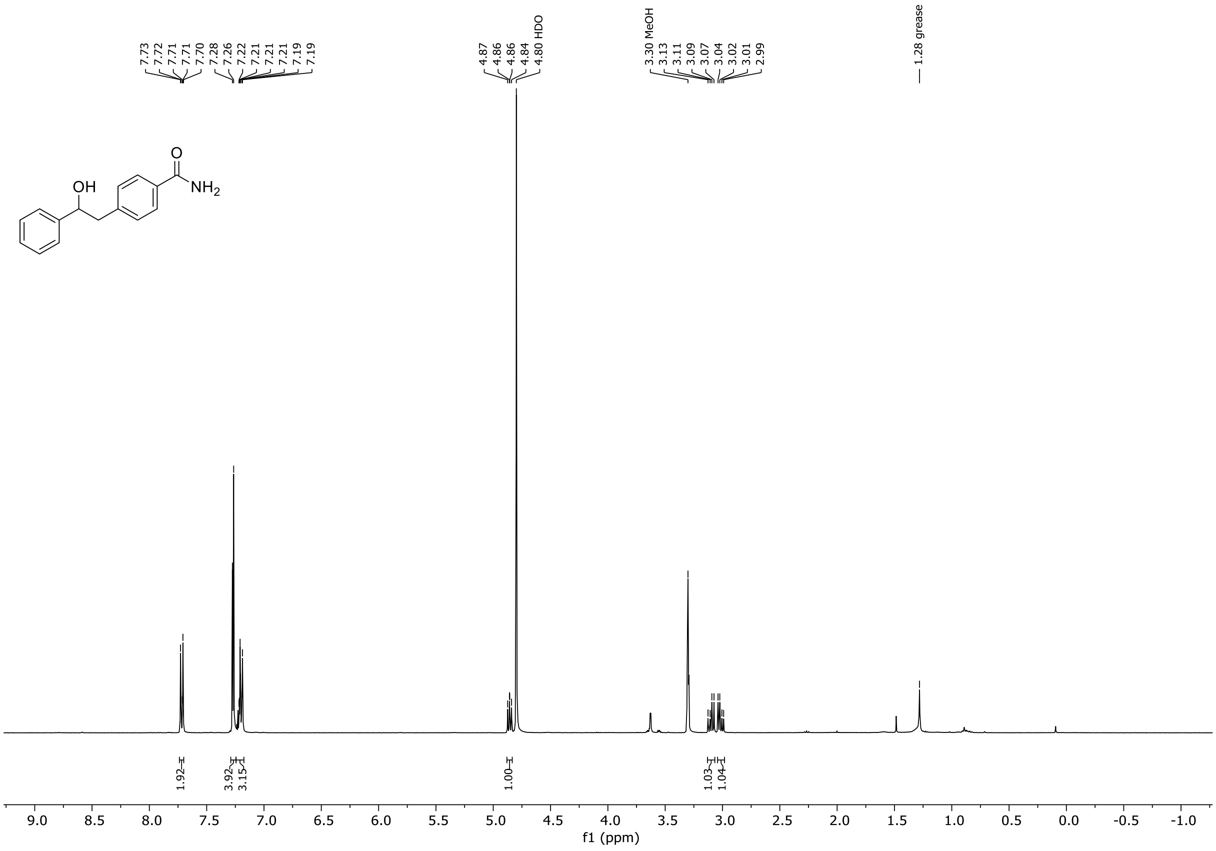

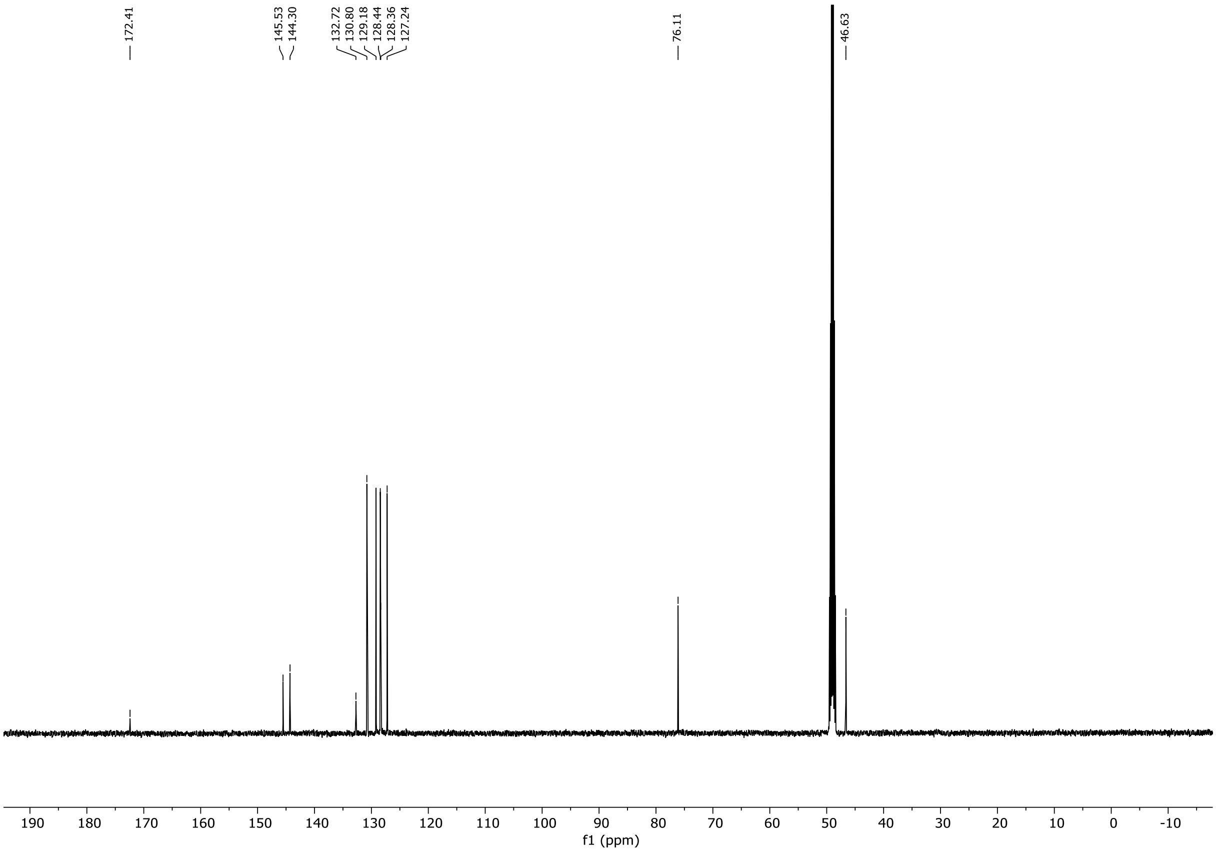

***tert*-butyl (4-(2-hydroxy-2-phenylethyl)phenyl)carbamate (7an)**

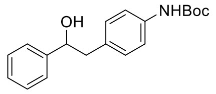

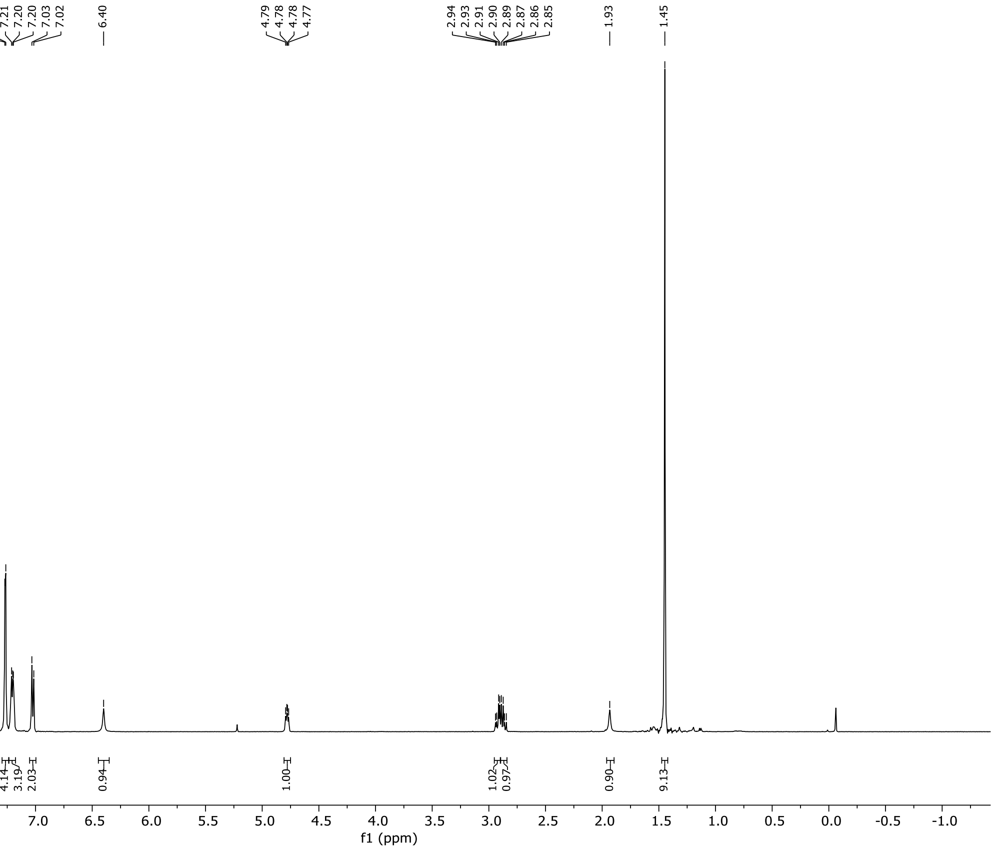

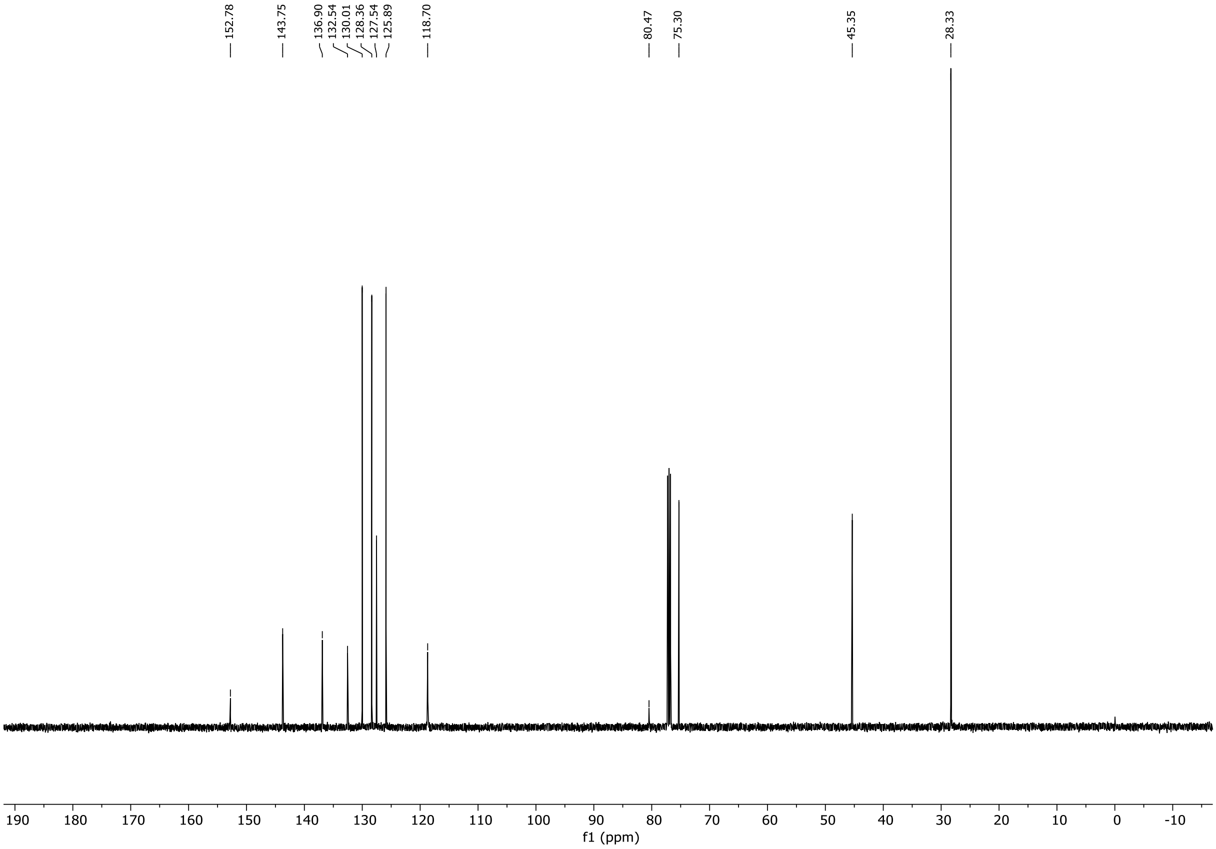

2-(4-(hydroxymethyl)phenyl)-1-phenylethan-1-ol (7ao)

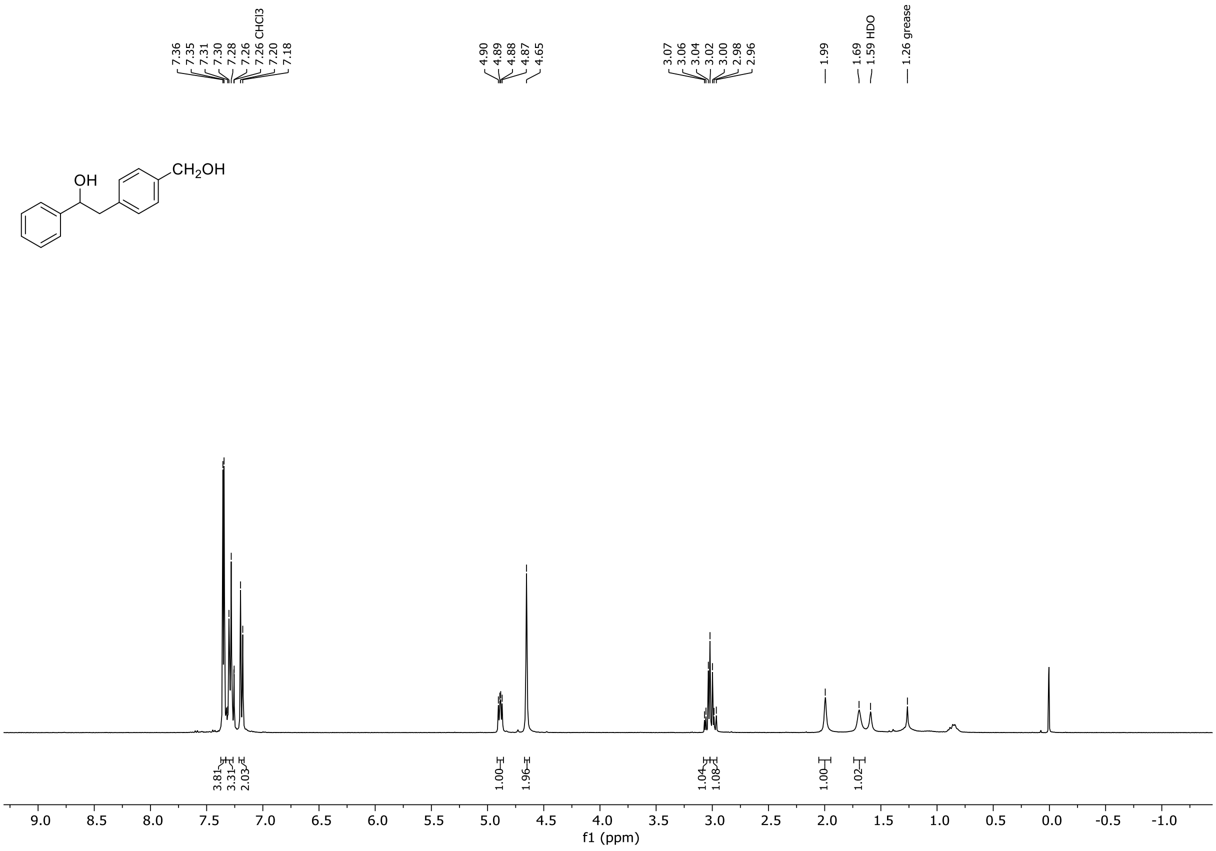

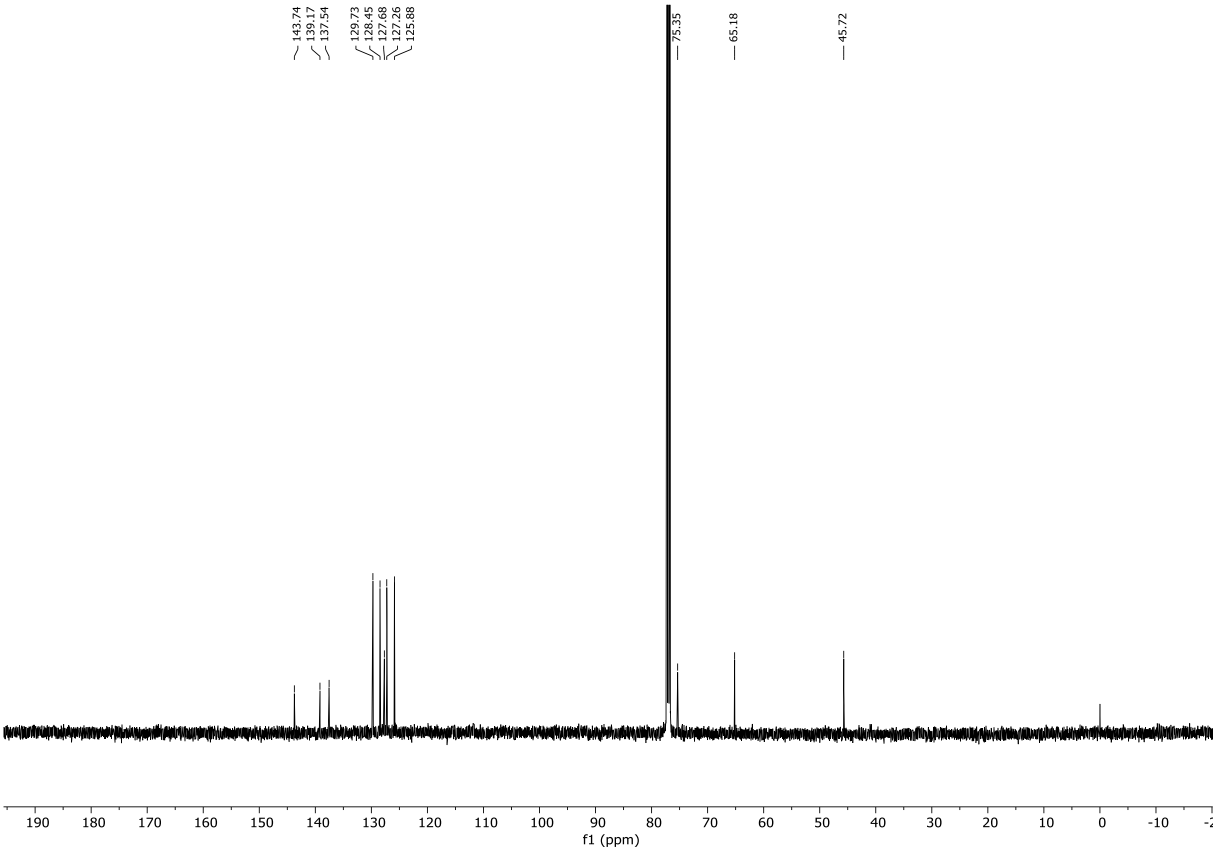

1-(*p*-tolyl)hexan-2-ol (7ha)

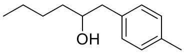

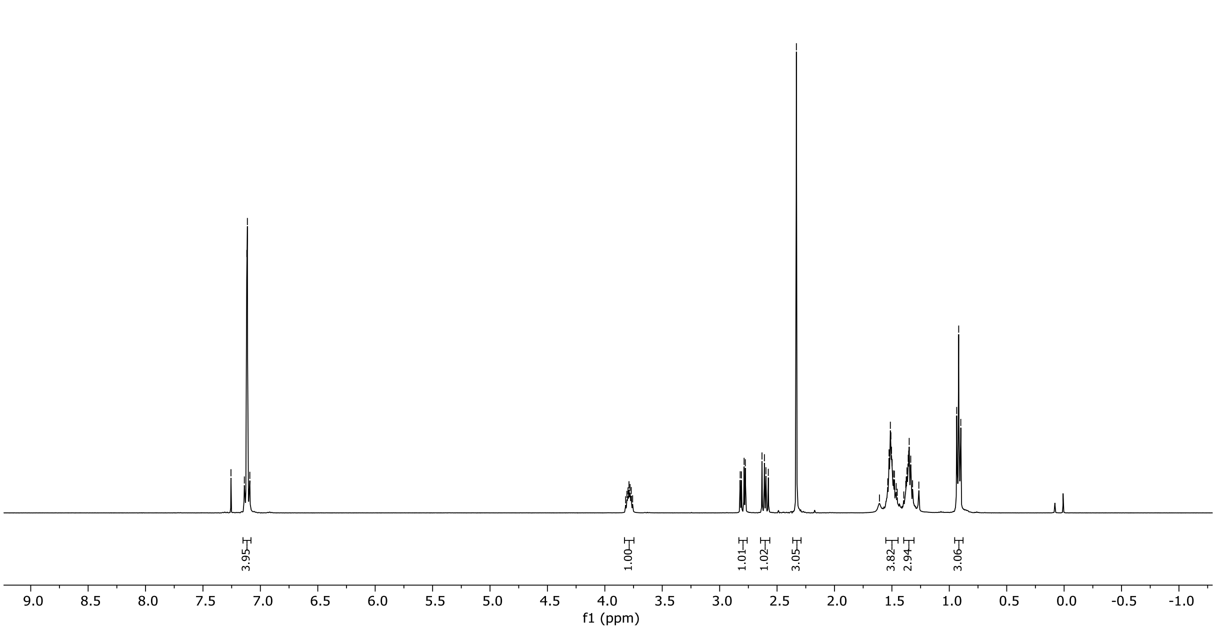

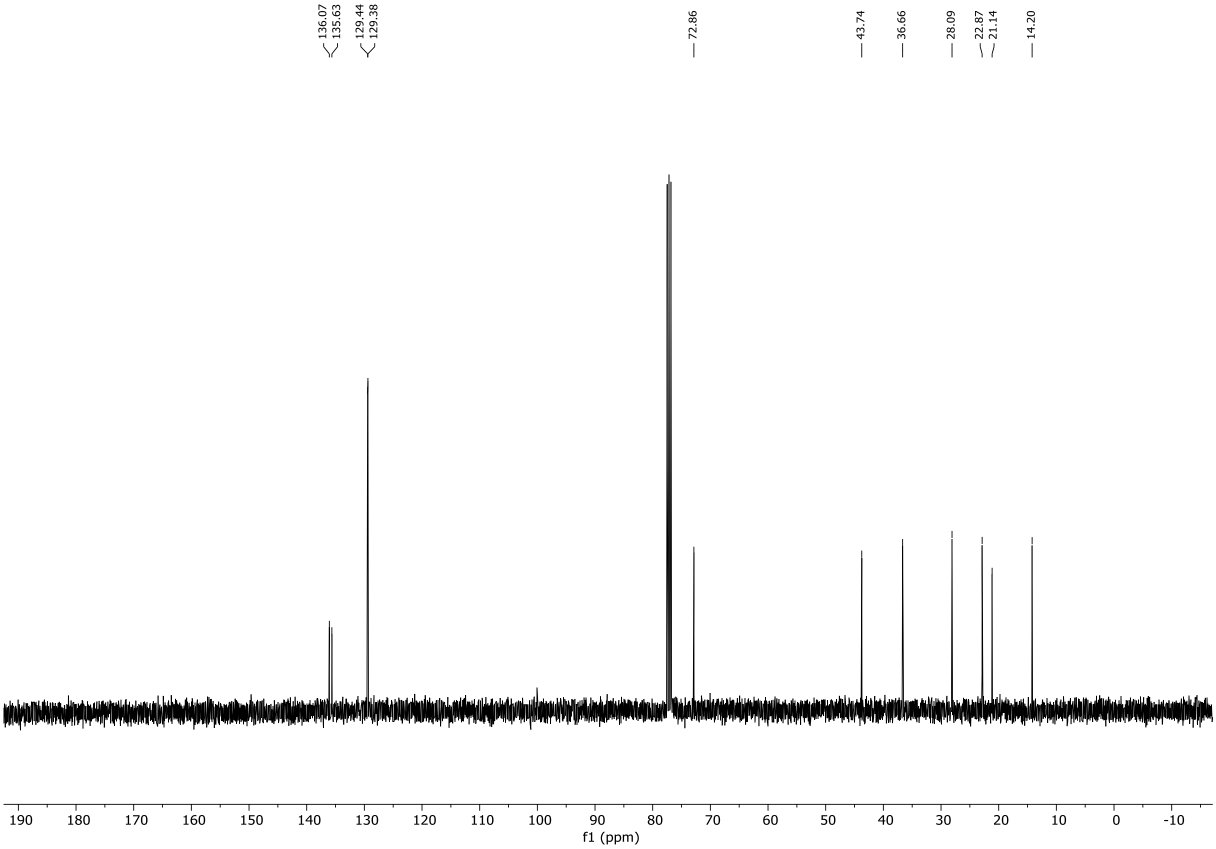

1-(*p*-tolyl)dodecan-2-ol (7ia)

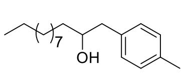

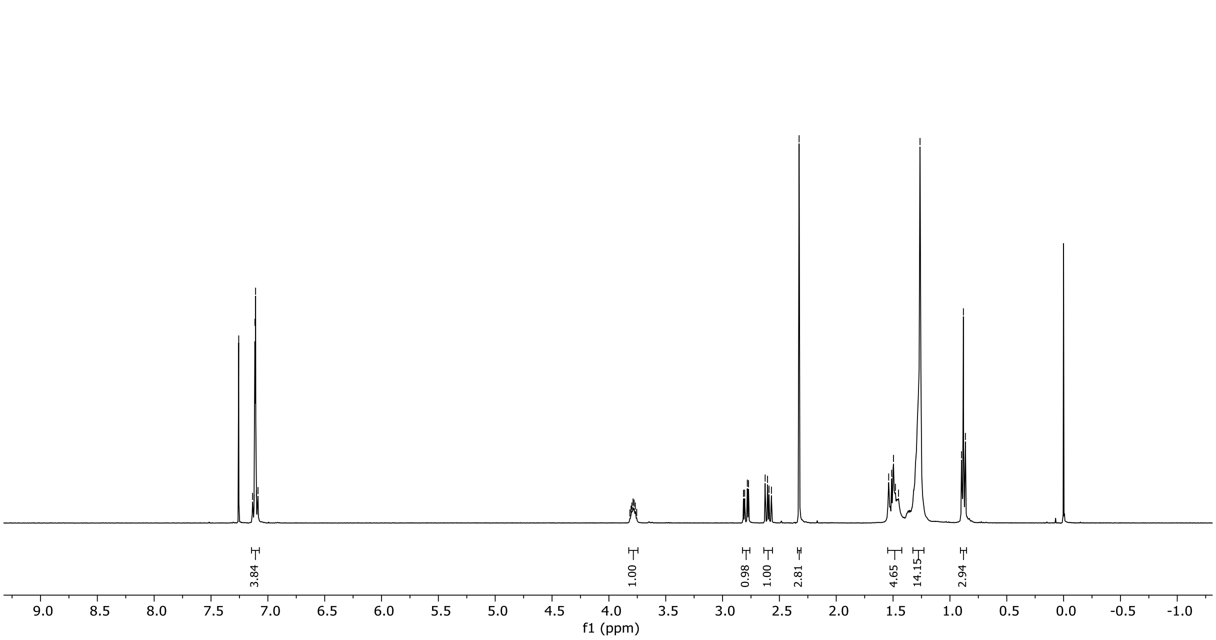

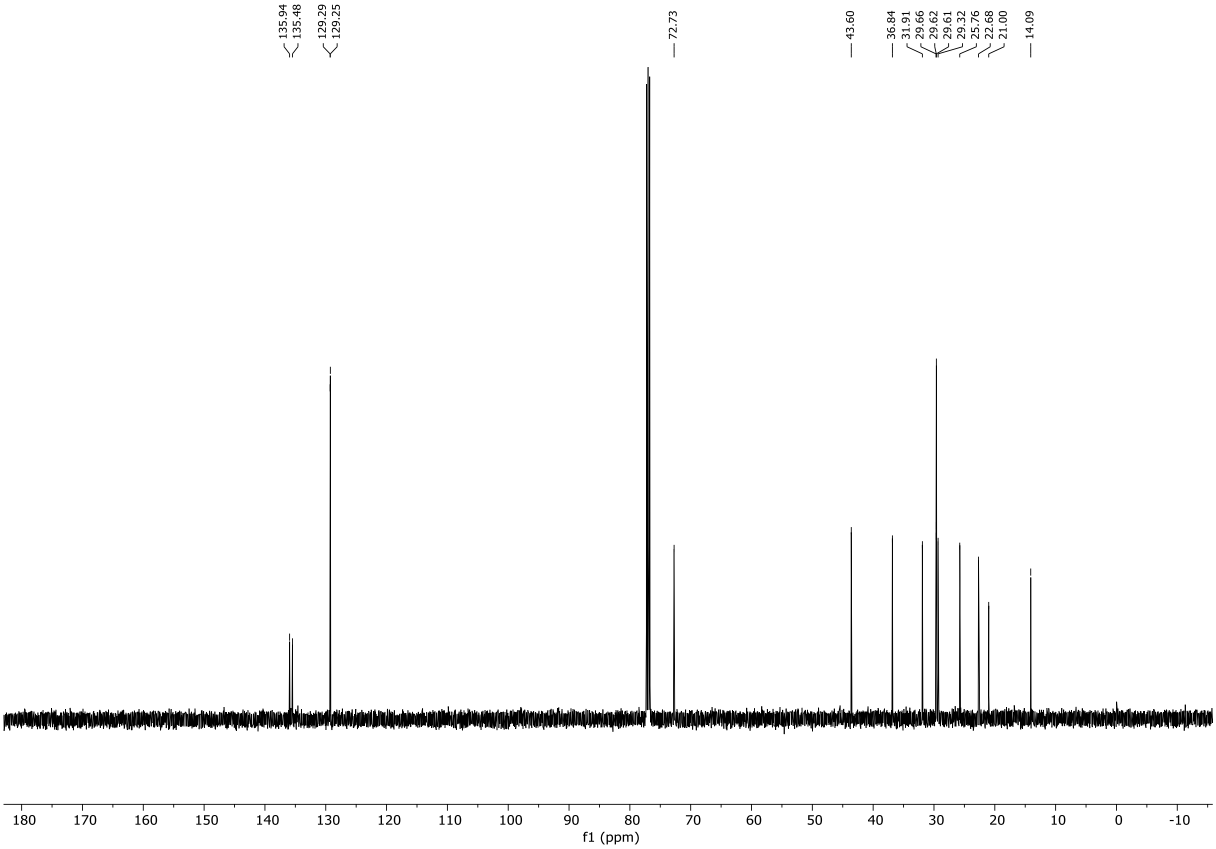

1-(benzyloxy)-3-(4-tolyl)propan-2-ol (7ja)

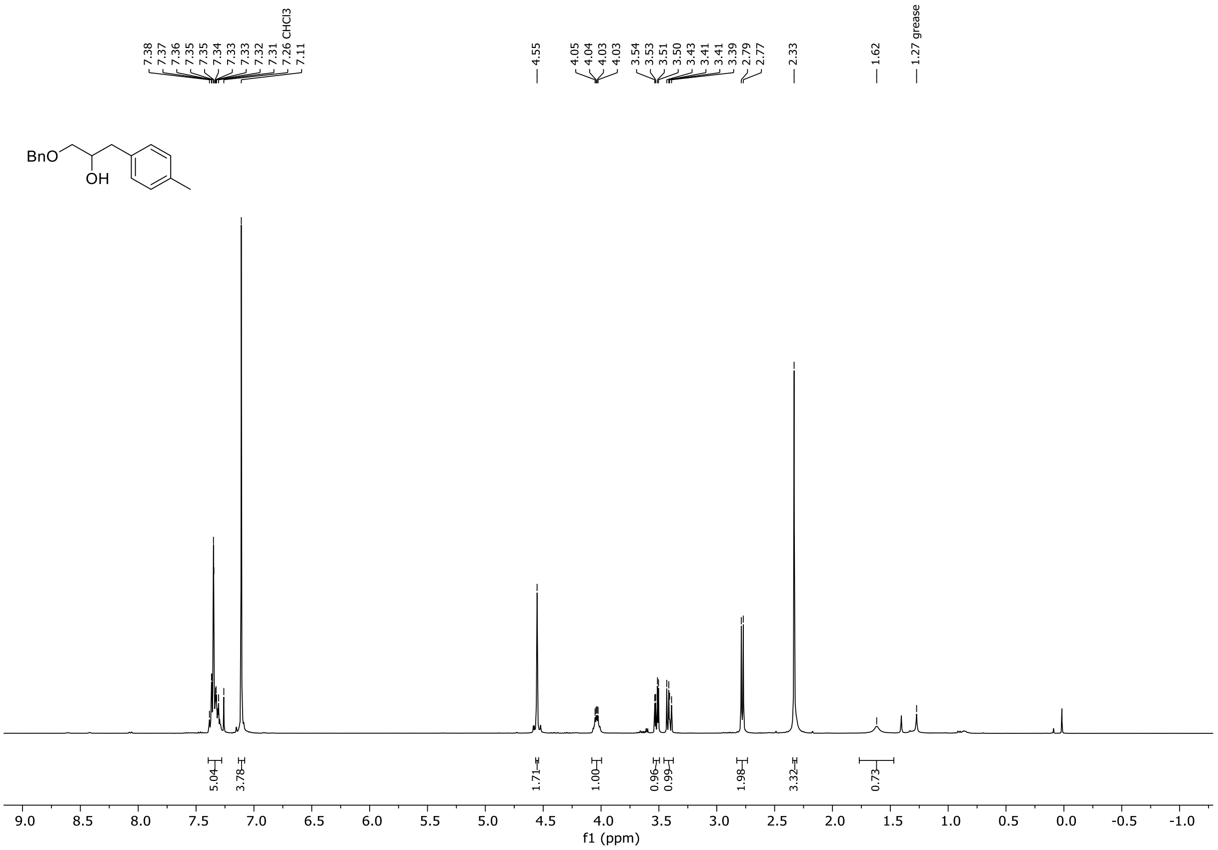

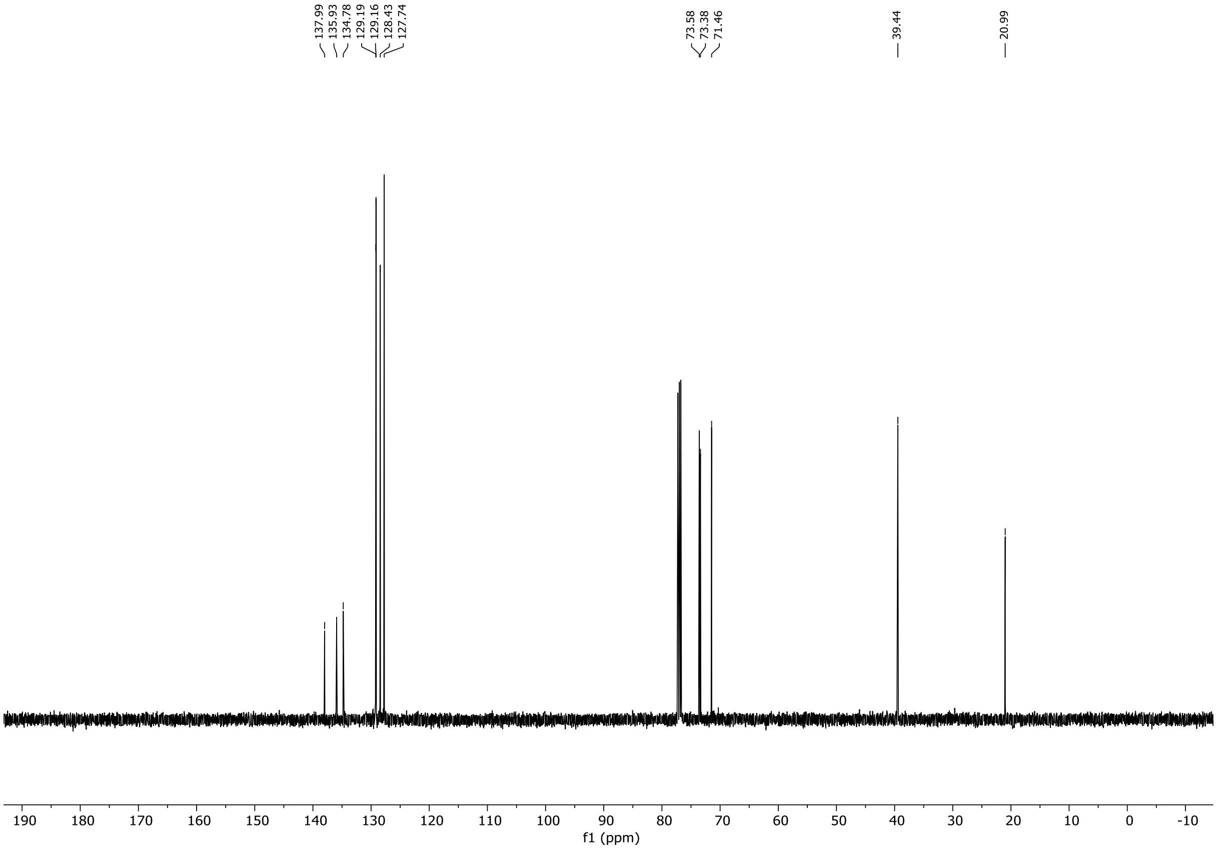

4-(phenylsulfonyl)-1-(4-tolyl)butan-2-ol (7ka)

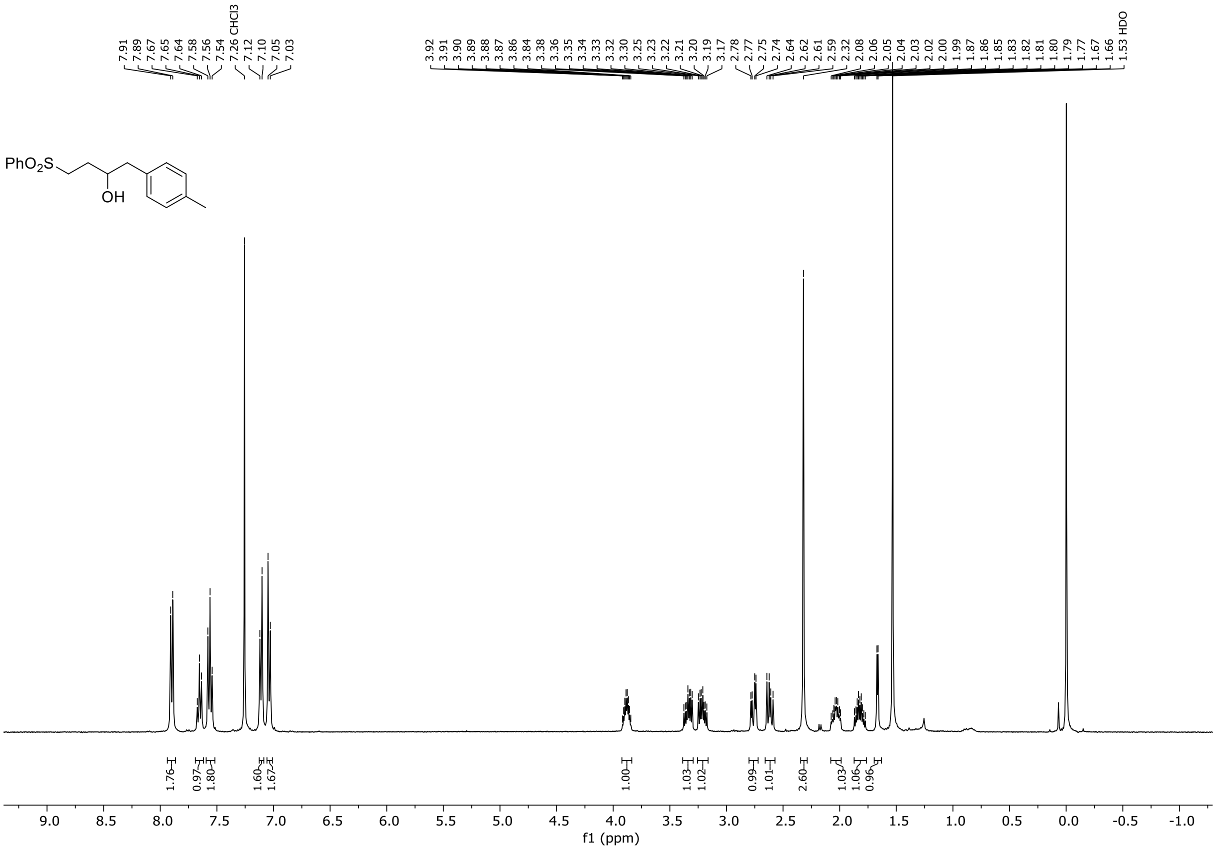

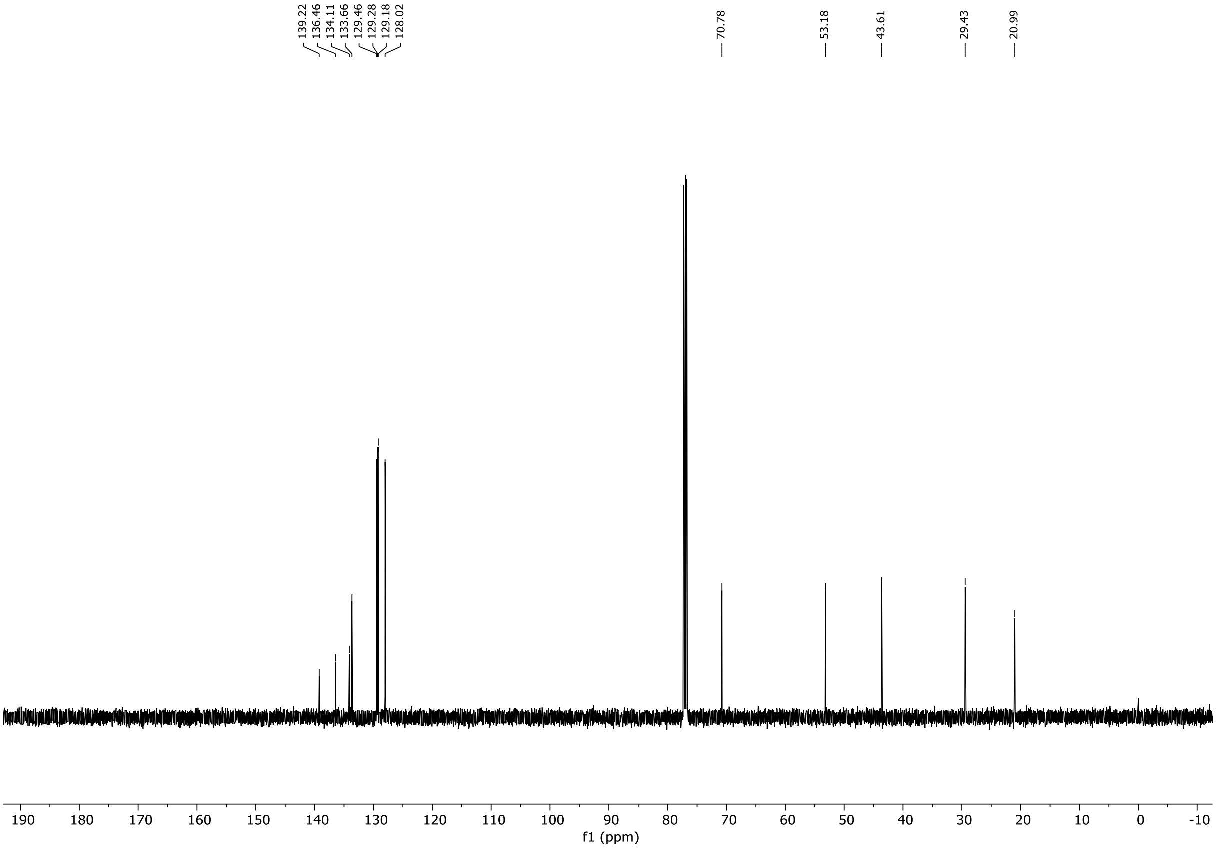

3,3-dimethyl-1-(4-tolyl)butan-2-ol (7la)

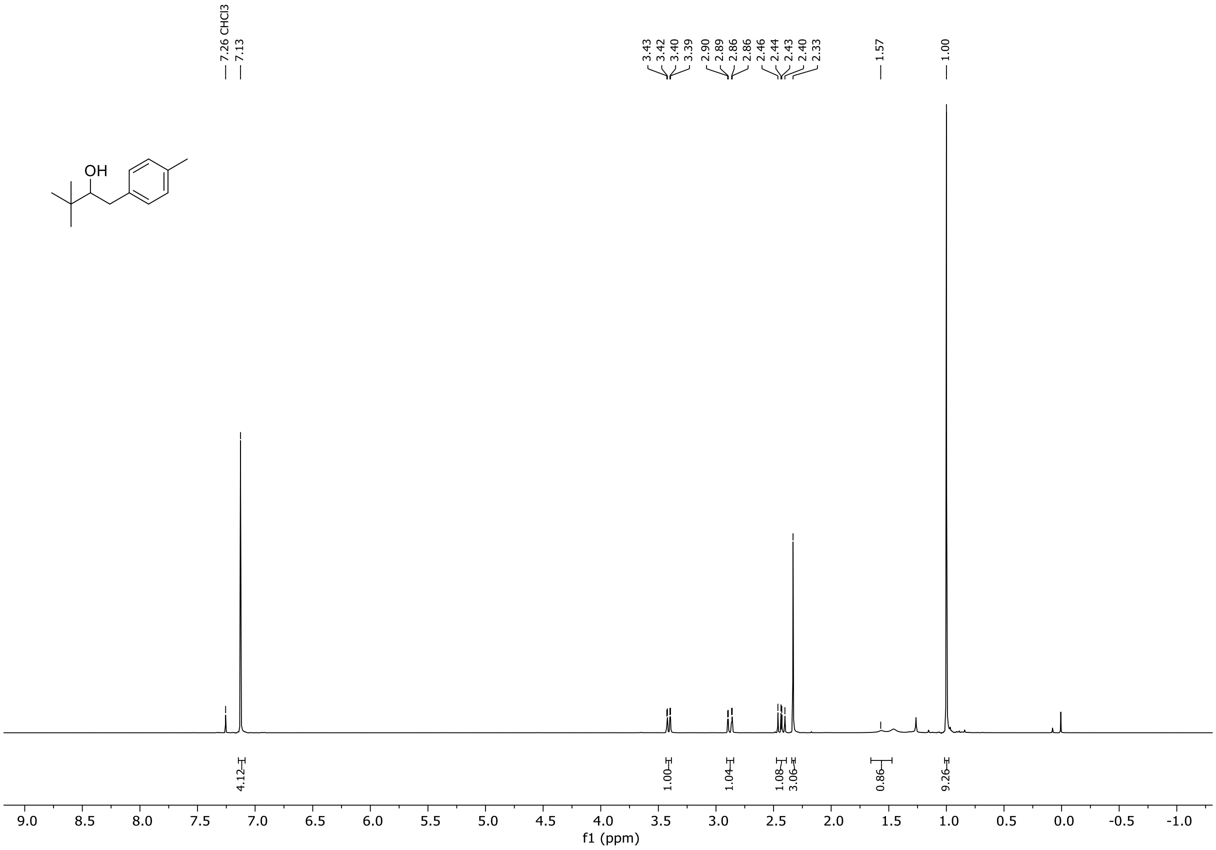

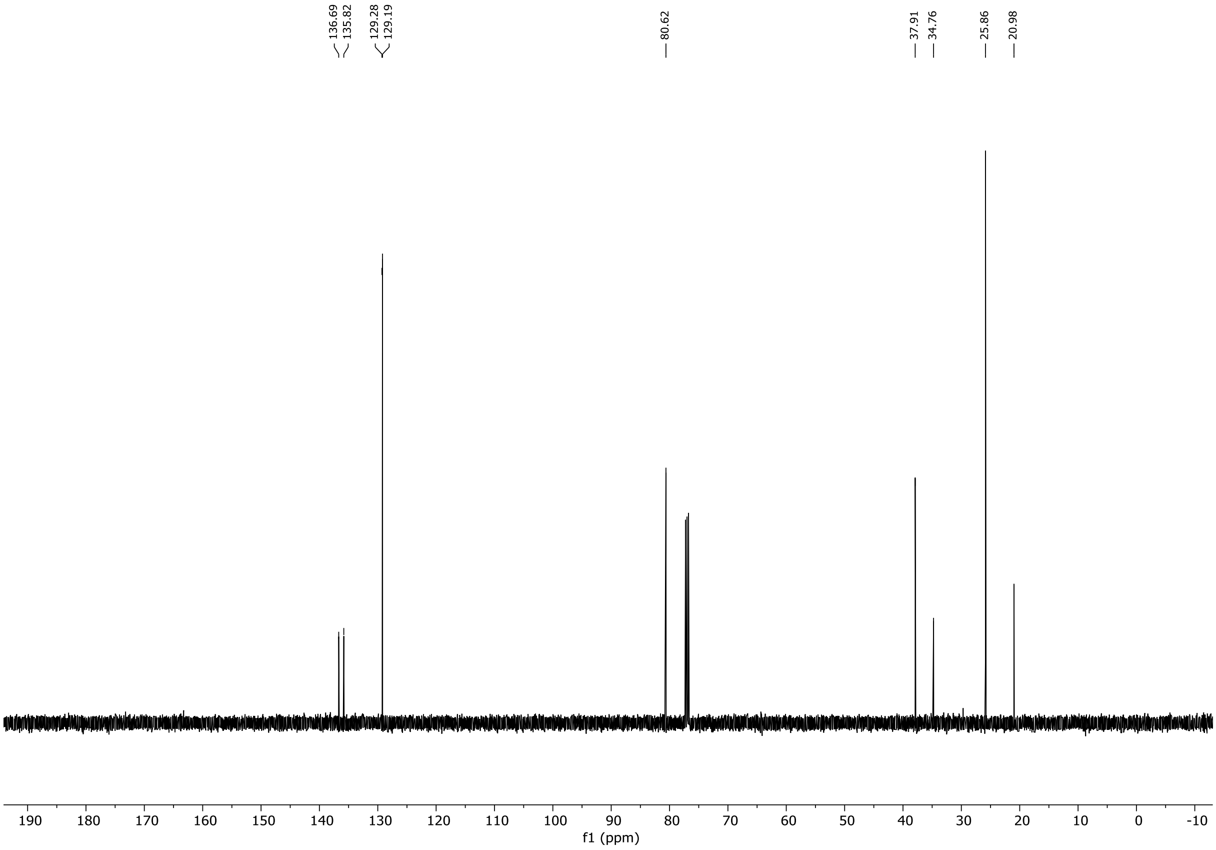

1-(4-methylbenzyl)cyclohexan-1-ol (7ma)

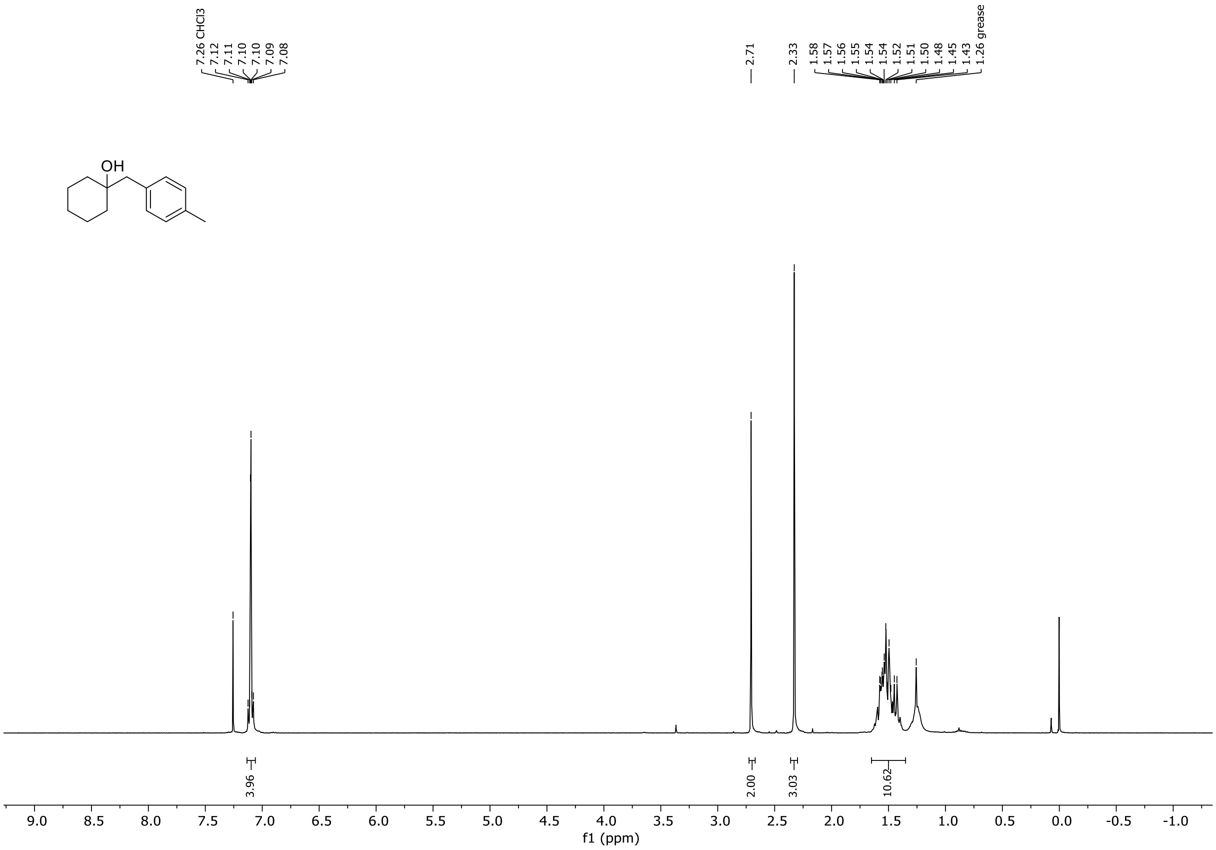

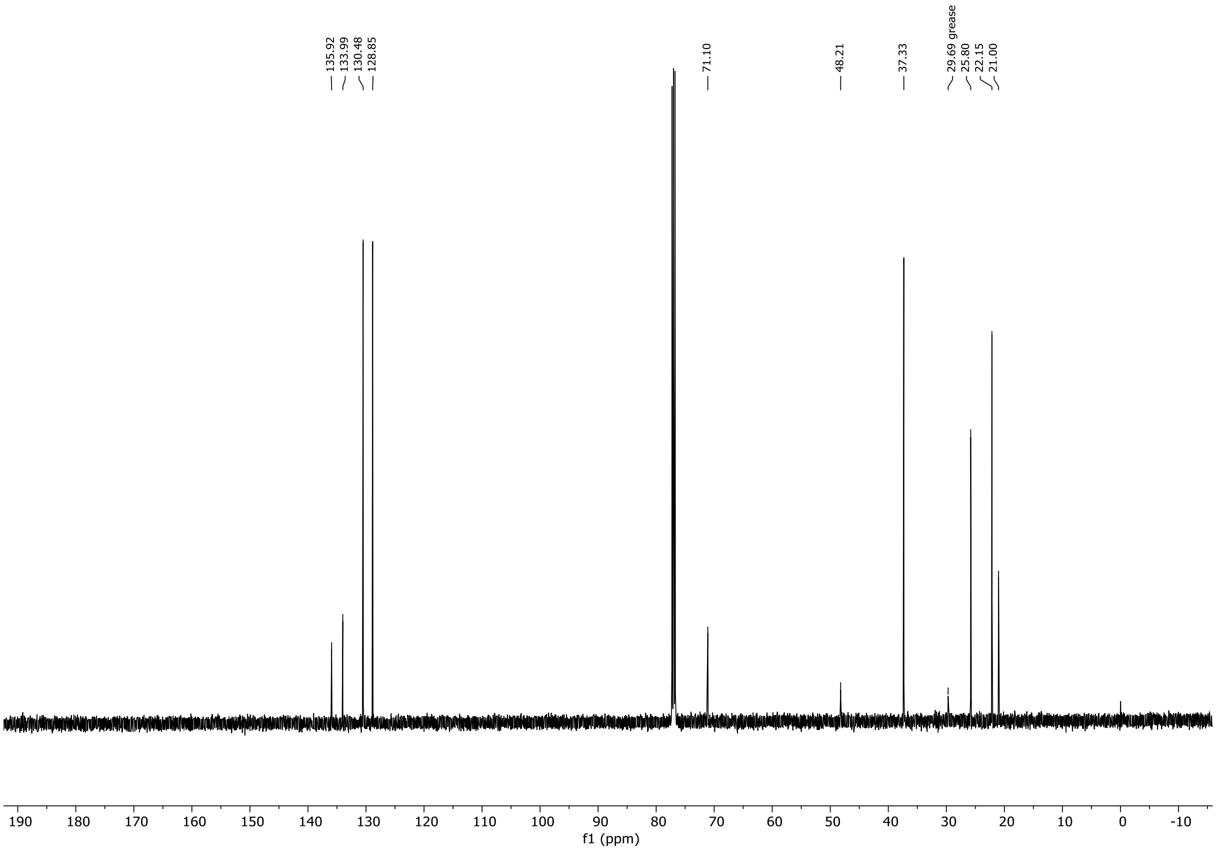

(1*S*,2*R*)-2-(4-tolyl)cyclopentan-1-ol (7na)

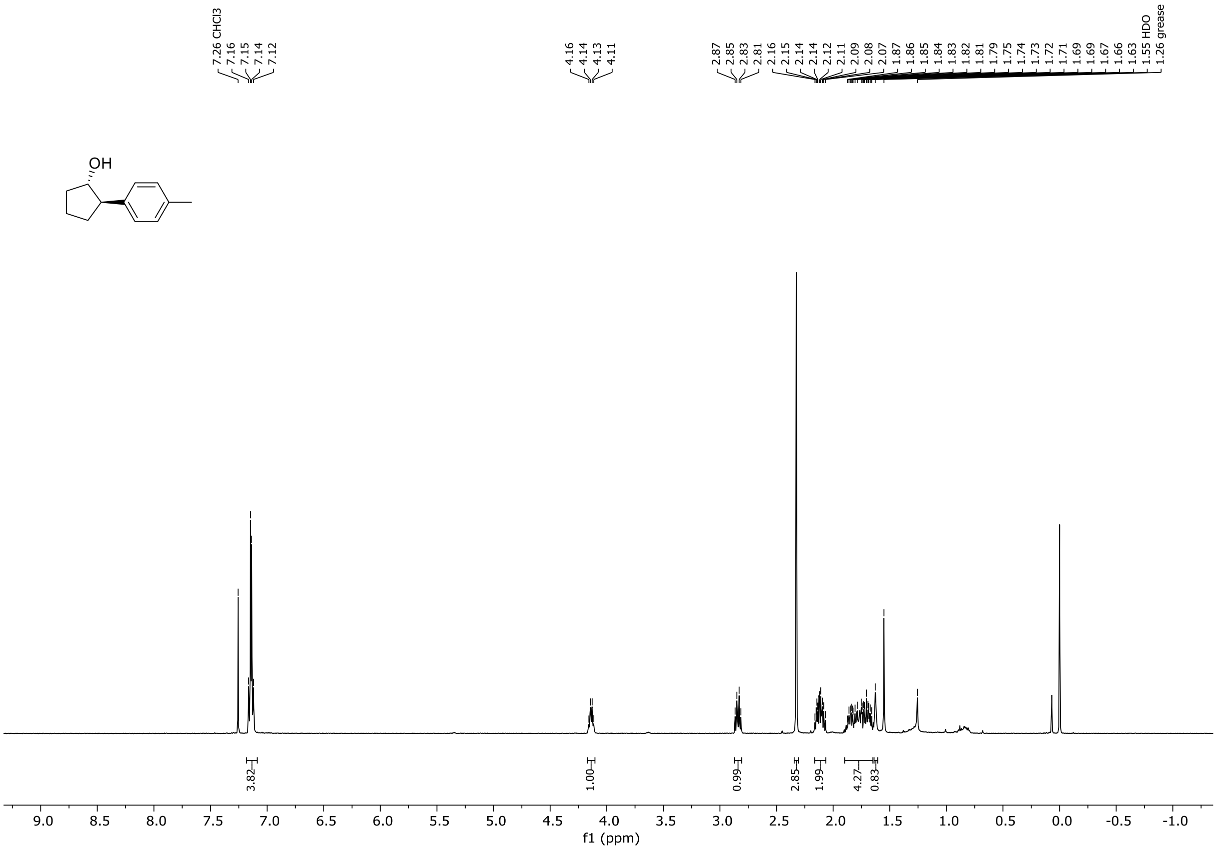

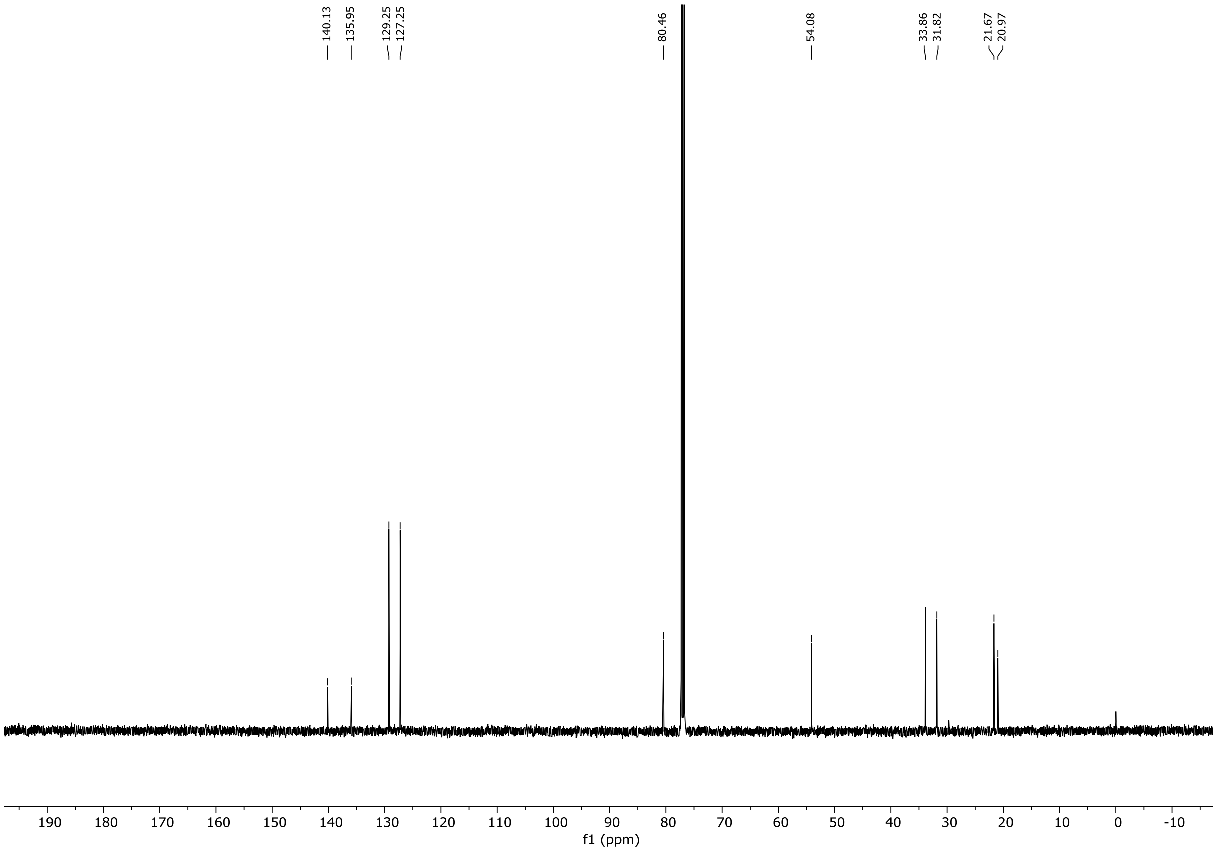

(1*S*,2*R*)-2-(4-tolyl)cyclohexan-1-ol (7oa)

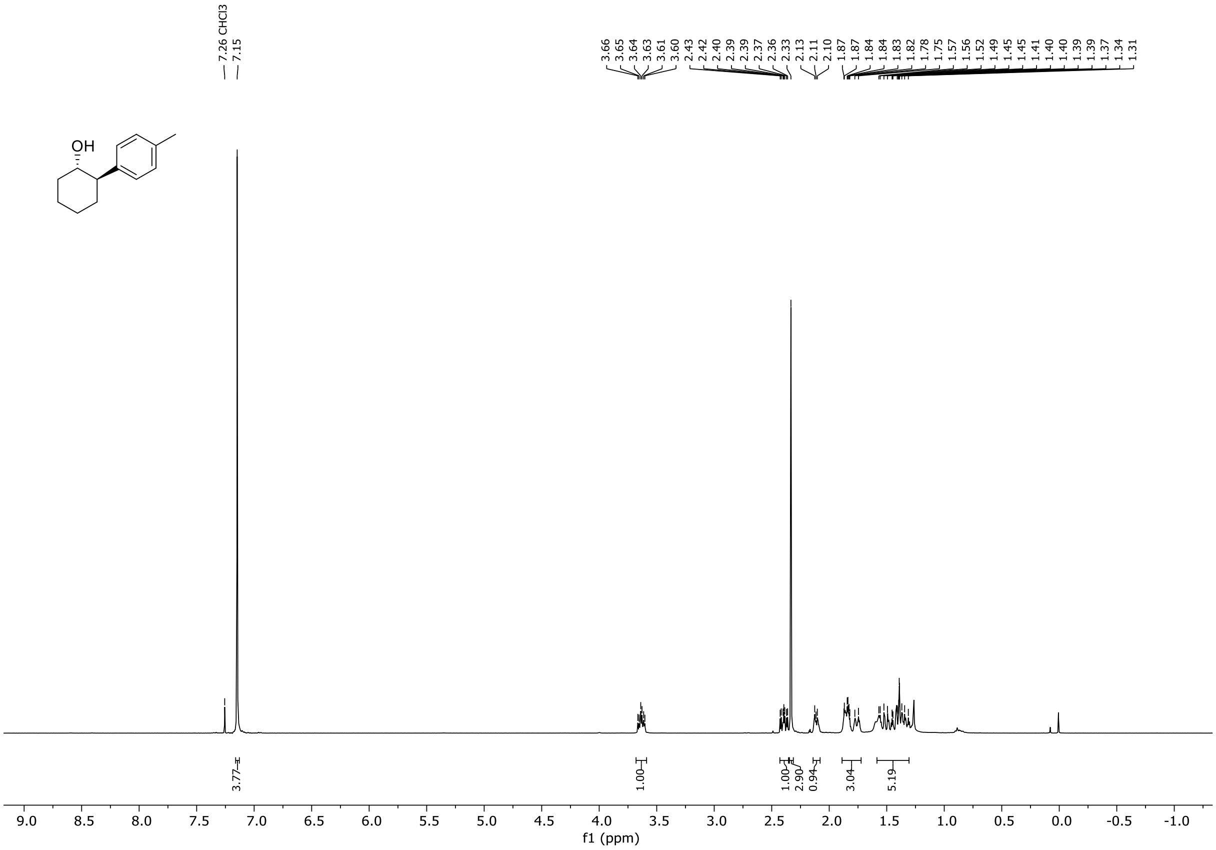

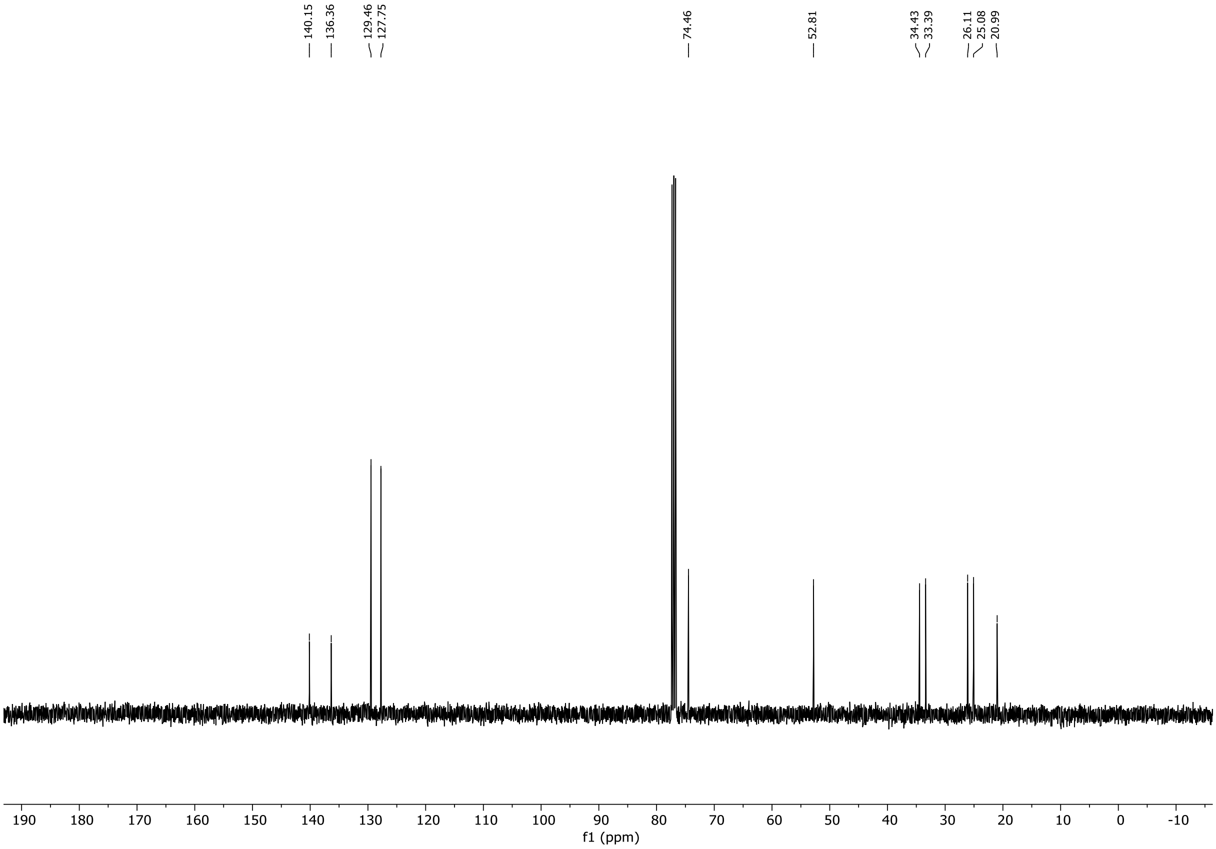

1-(4-chlorophenyl)hexan-2-ol (7hd)

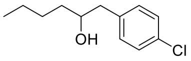

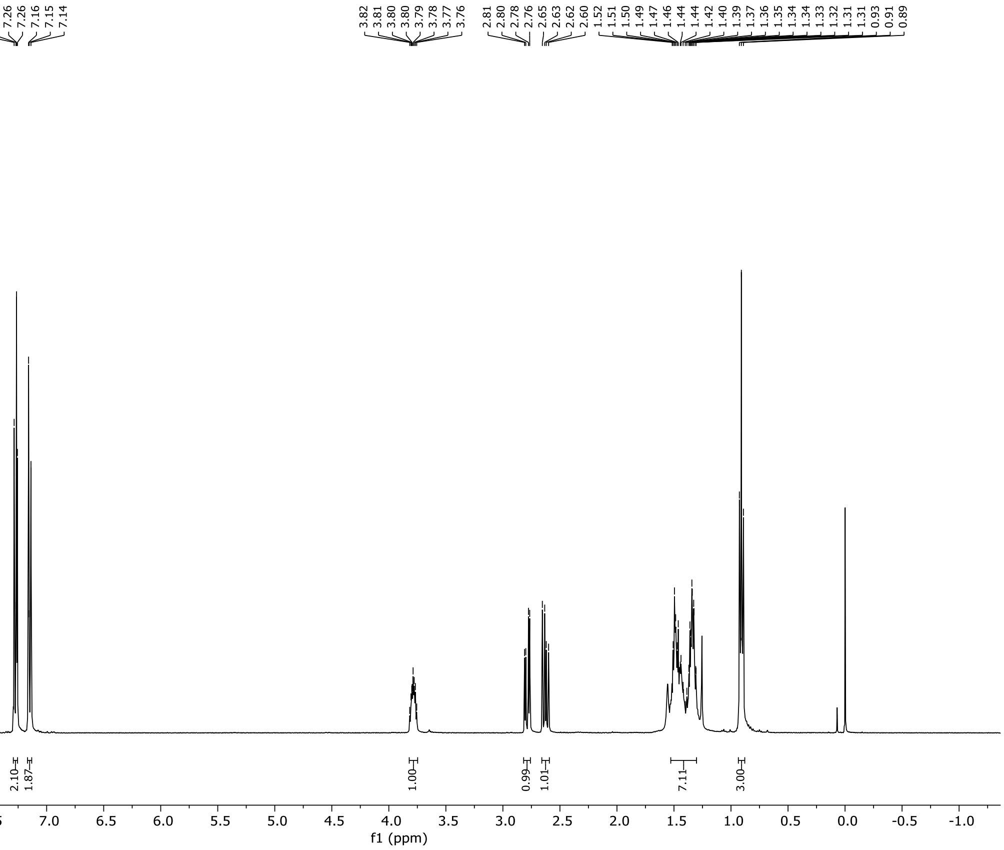

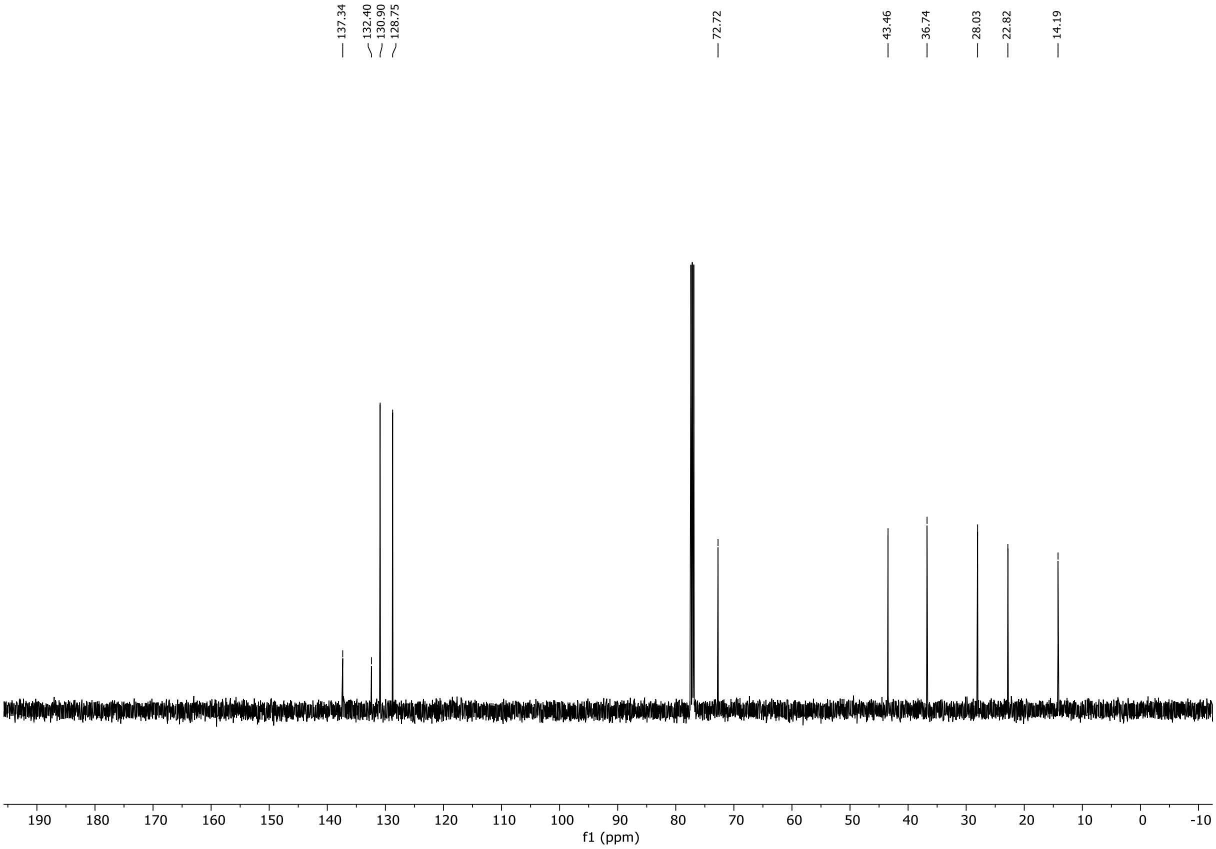

1-(benzo[*d*][1,3]dioxol-5-yl)hexan-2-ol (7he)

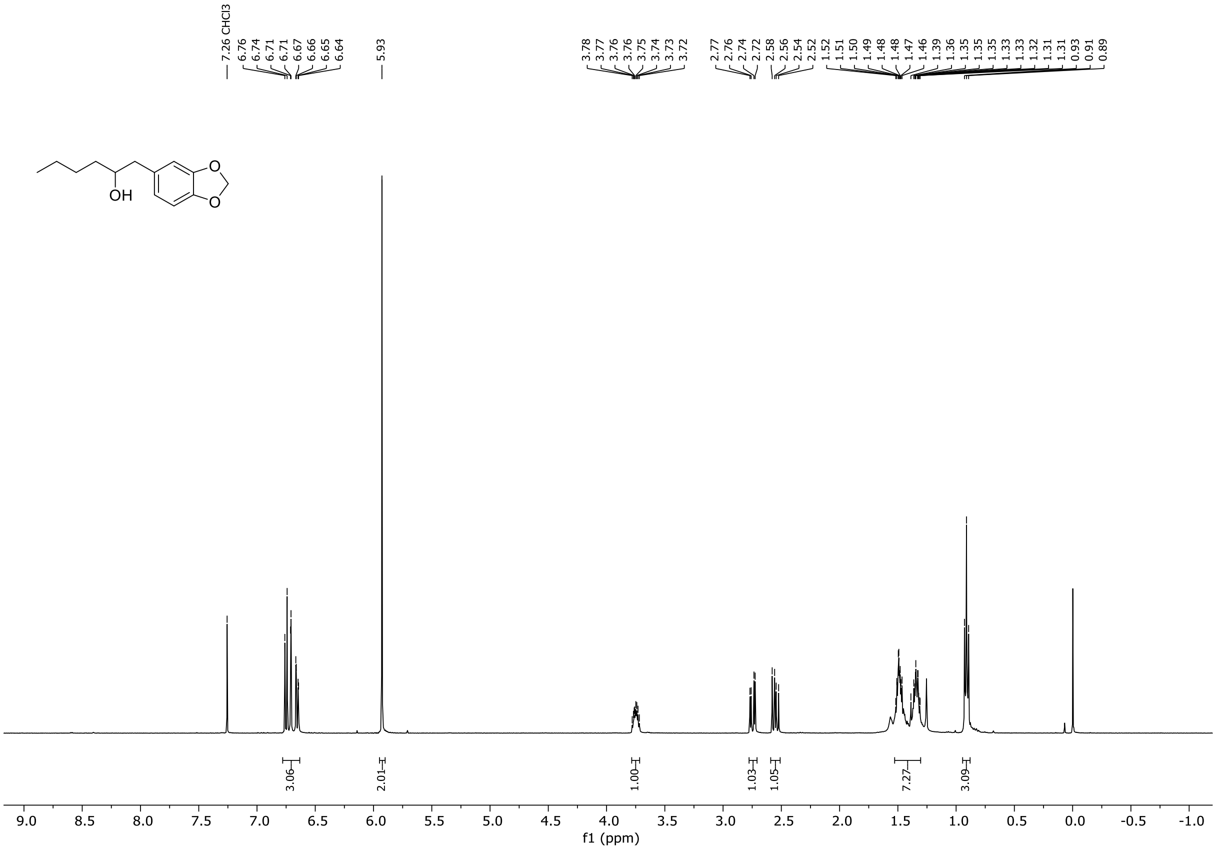

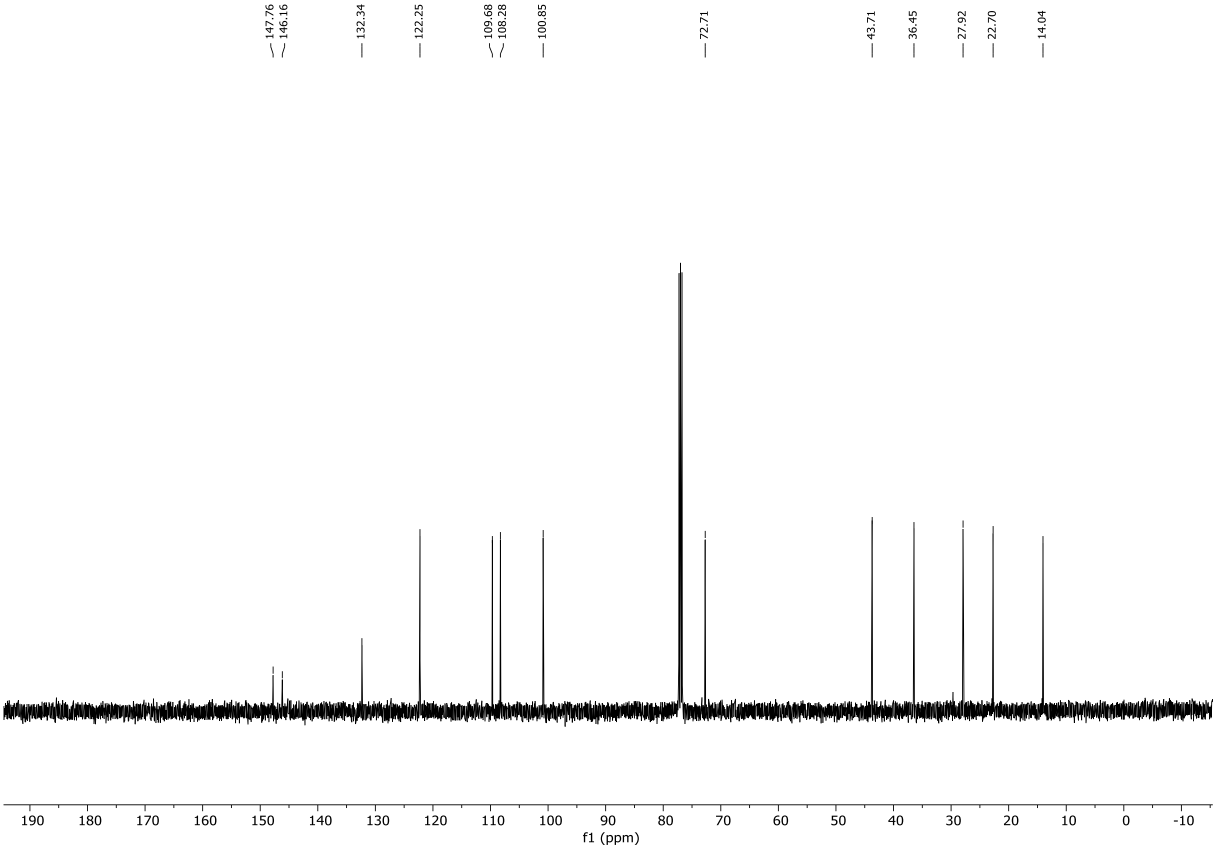

1-(4-(2-hydroxyhexyl)phenyl)ethan-1-one (7hg)

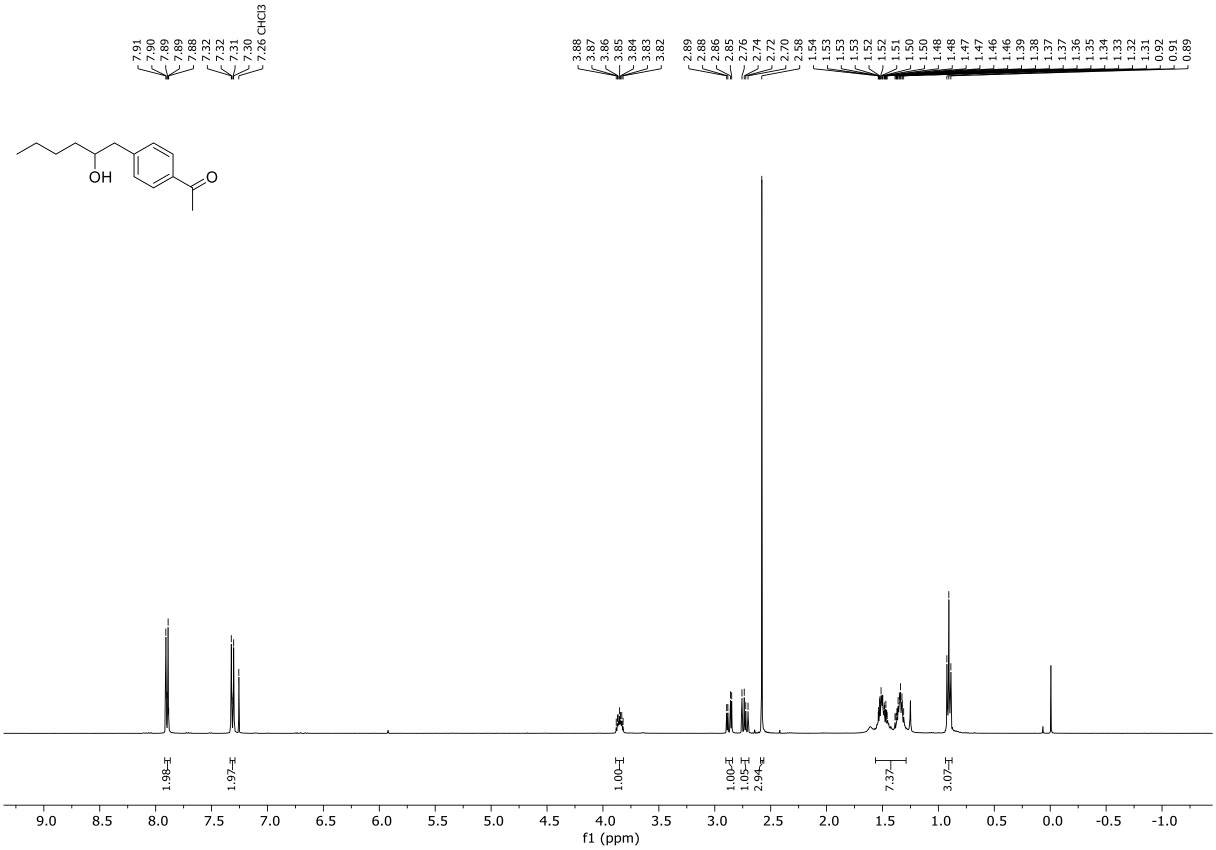

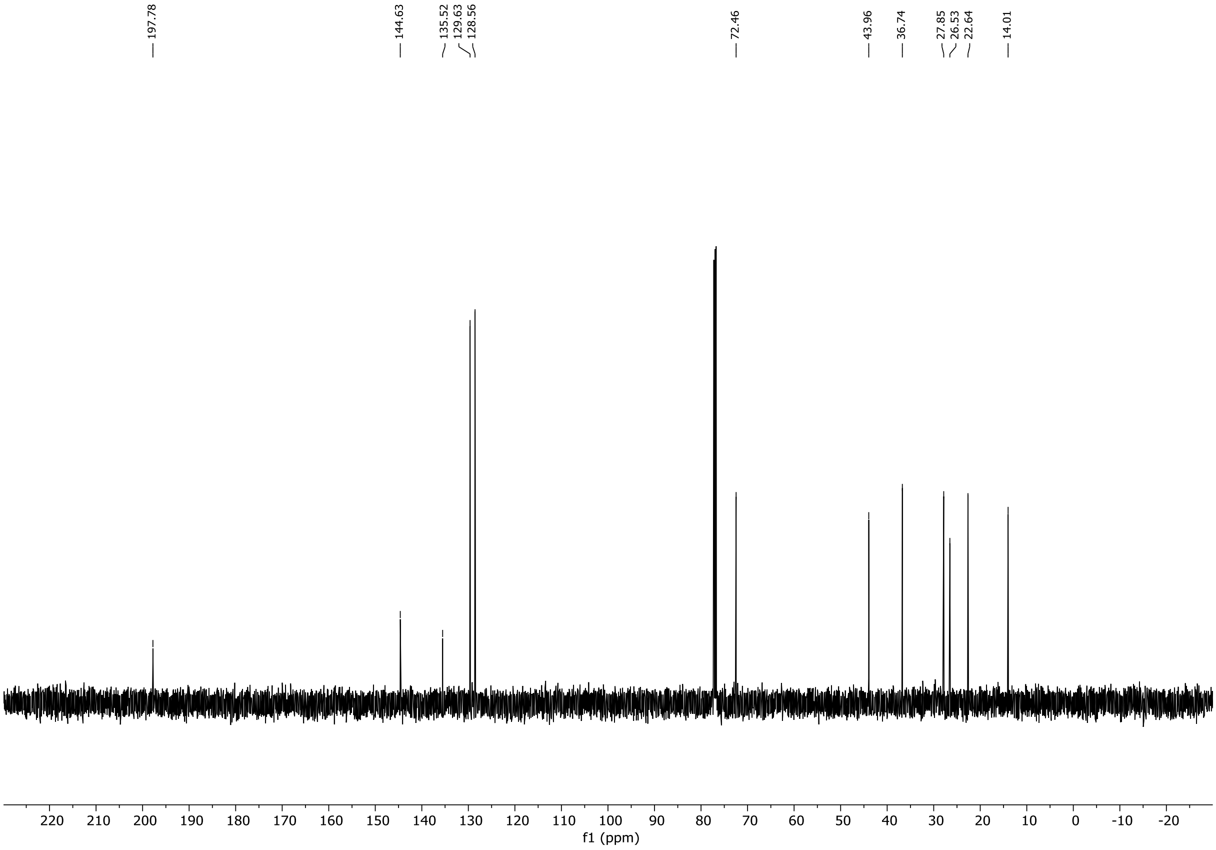

tert-butyl (4-(2-hydroxyhexyl)phenyl)carbamate (7hn)

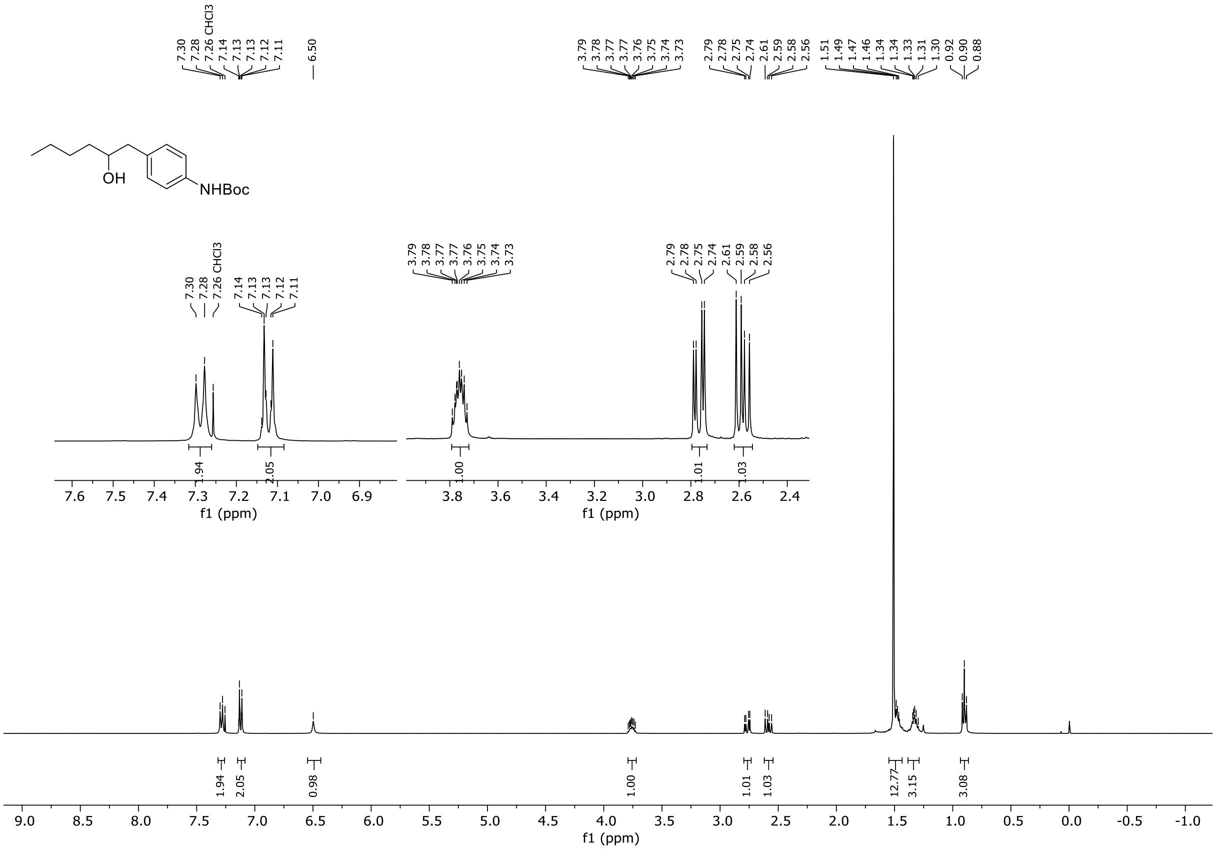

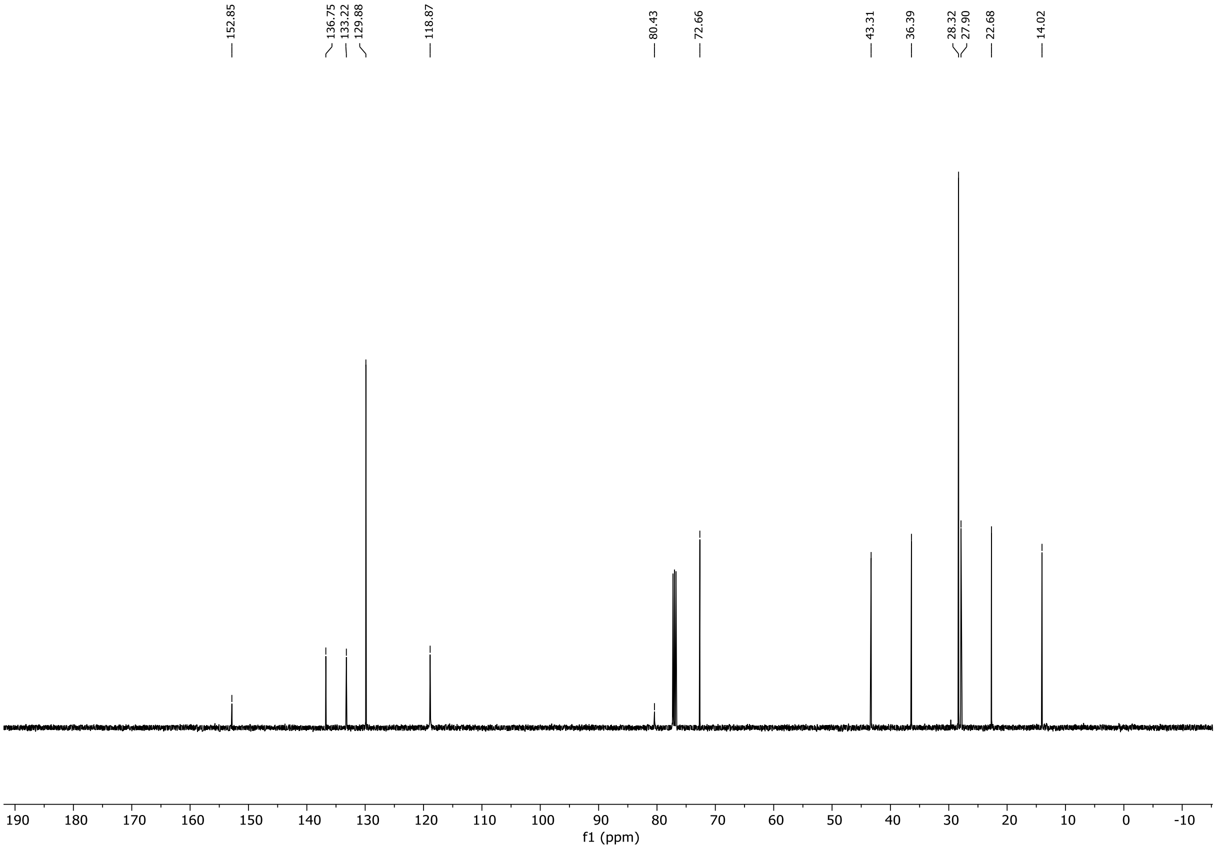

1-(1-tosyl-1*H*-indol-5-yl)hexan-2-ol (7hl)

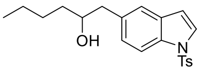

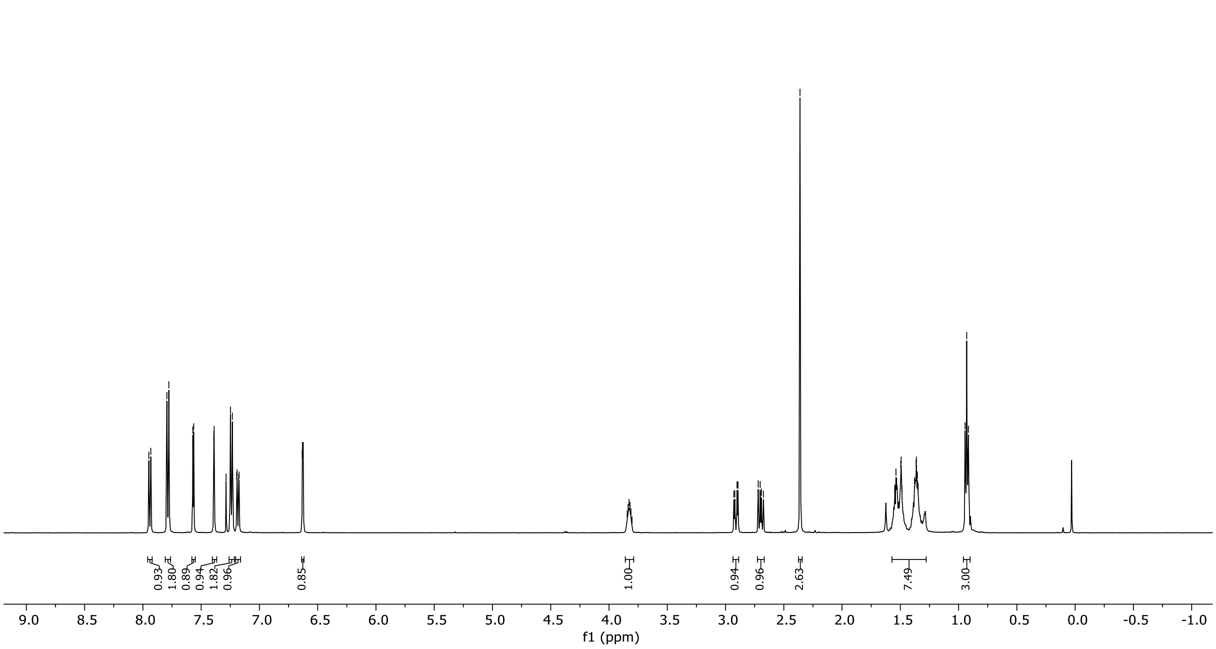

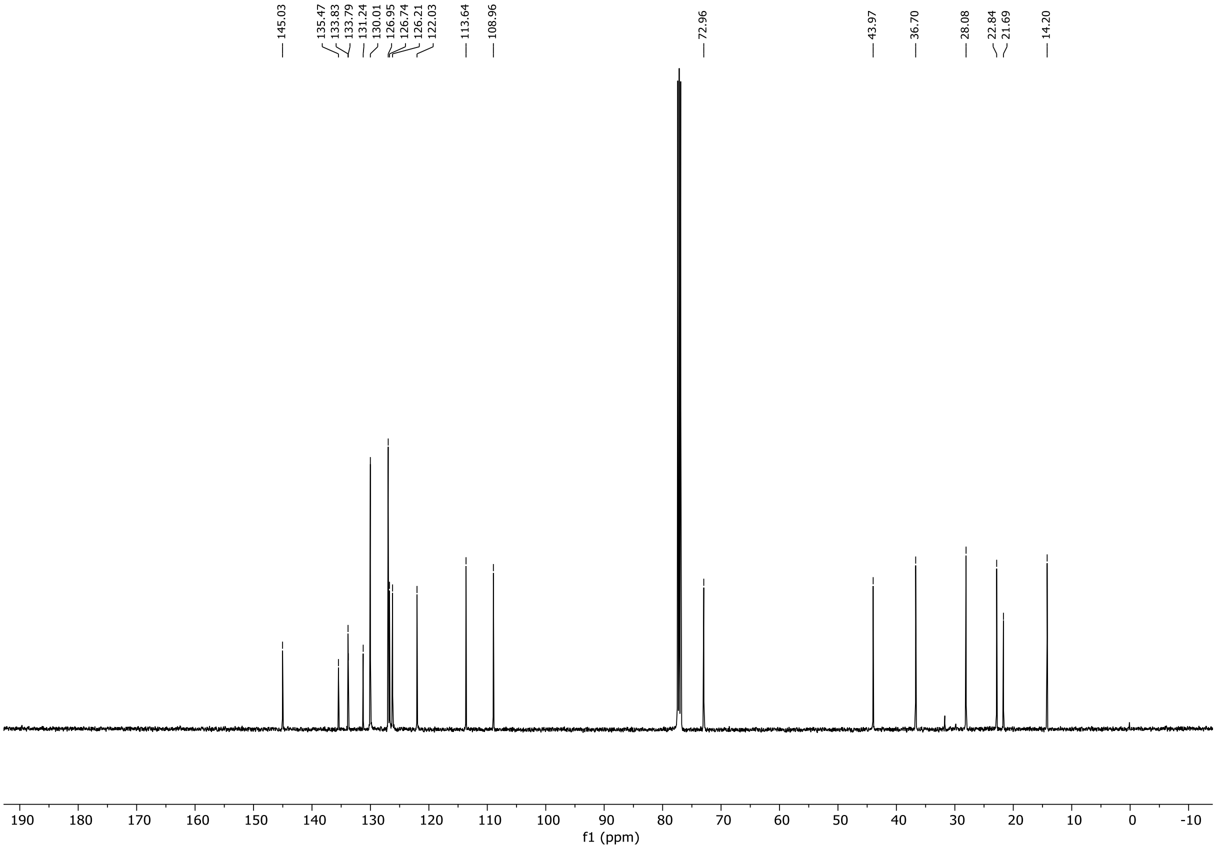

4-methyl-N-(2-phenyl-(*p*-tolyl)ethyl)benzenosulphonamide (S23) and 4-methyl-N-(1-phenyl-2-(*p*-tolyl)ethyl)benzenosulfonamide (S24)

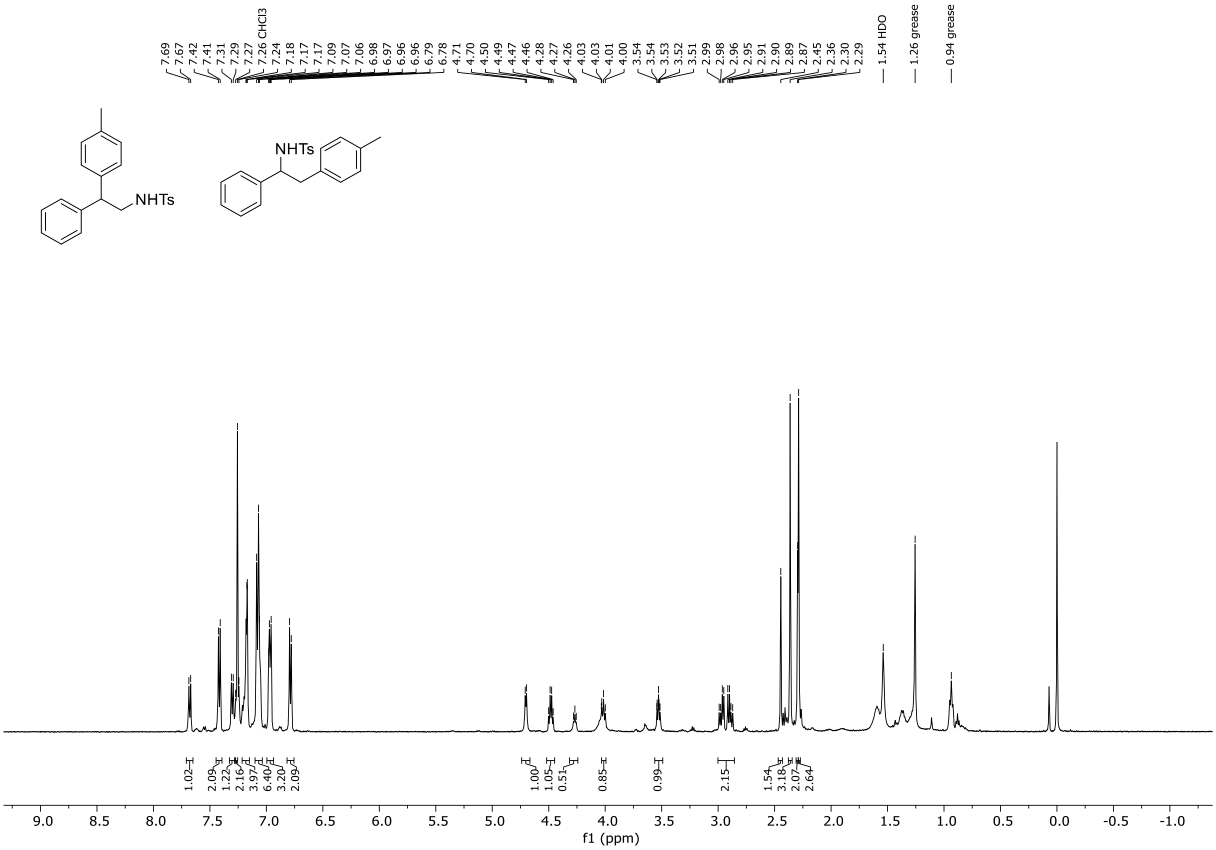

4-methyl-N-(2-(*p*-tolyl)cyclopentyl)benzenosulfonamide (S25)

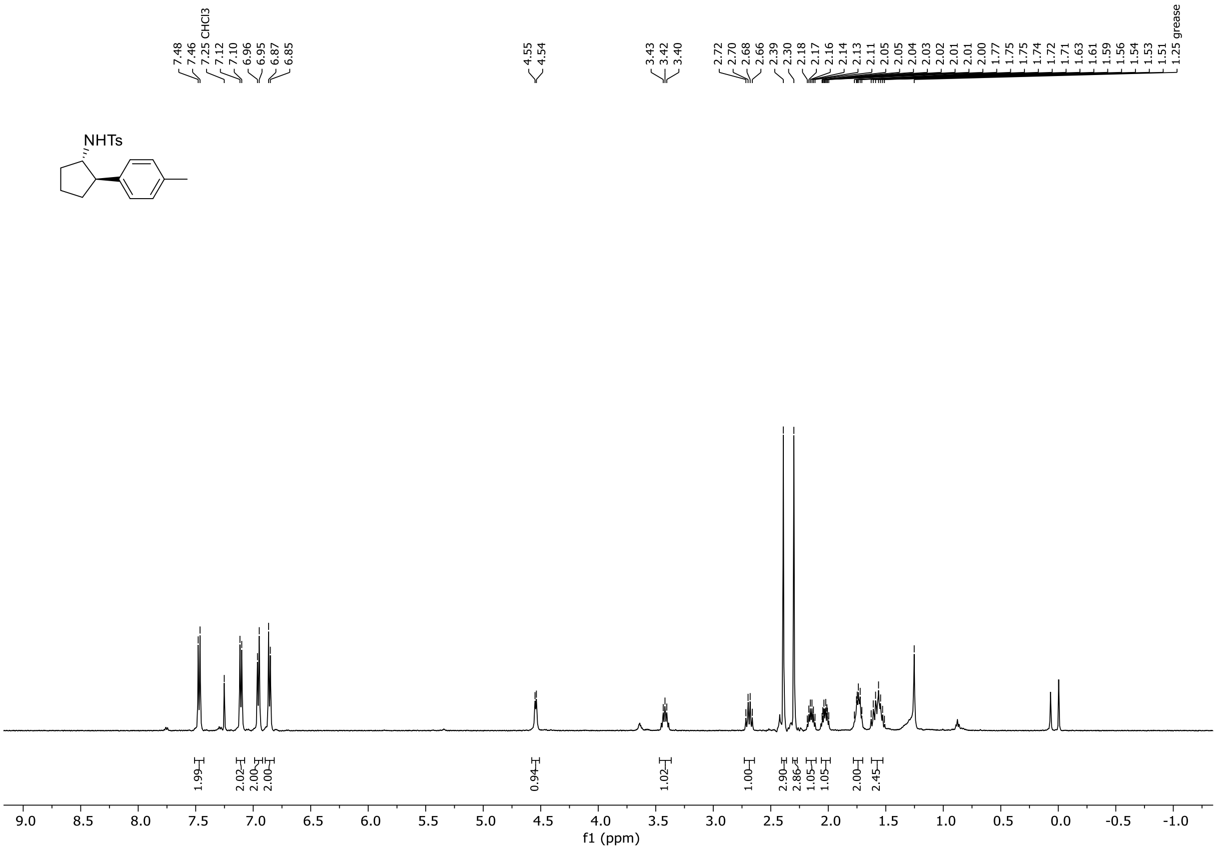

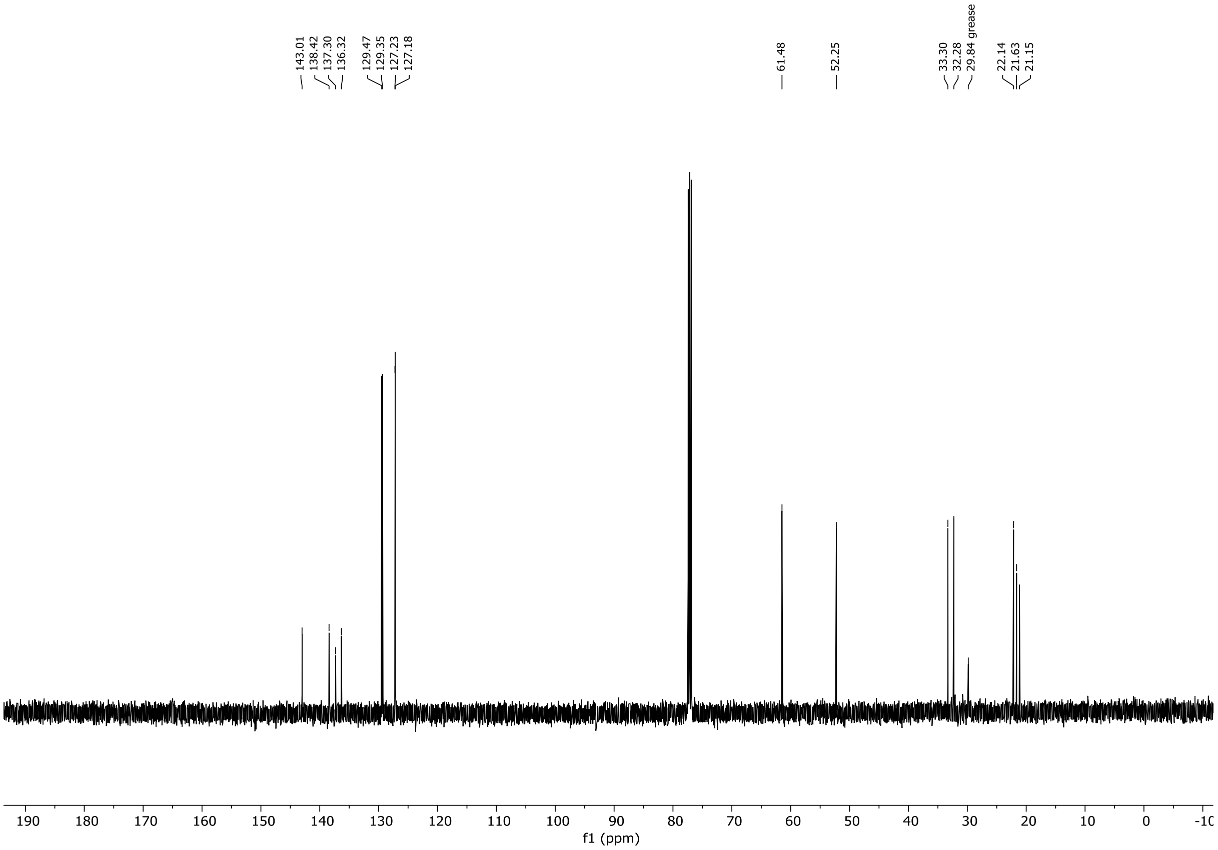

4-methyl-N-(1-(*p*-tolyl)hexan-2-yl)benzenosulfonamide (S26)

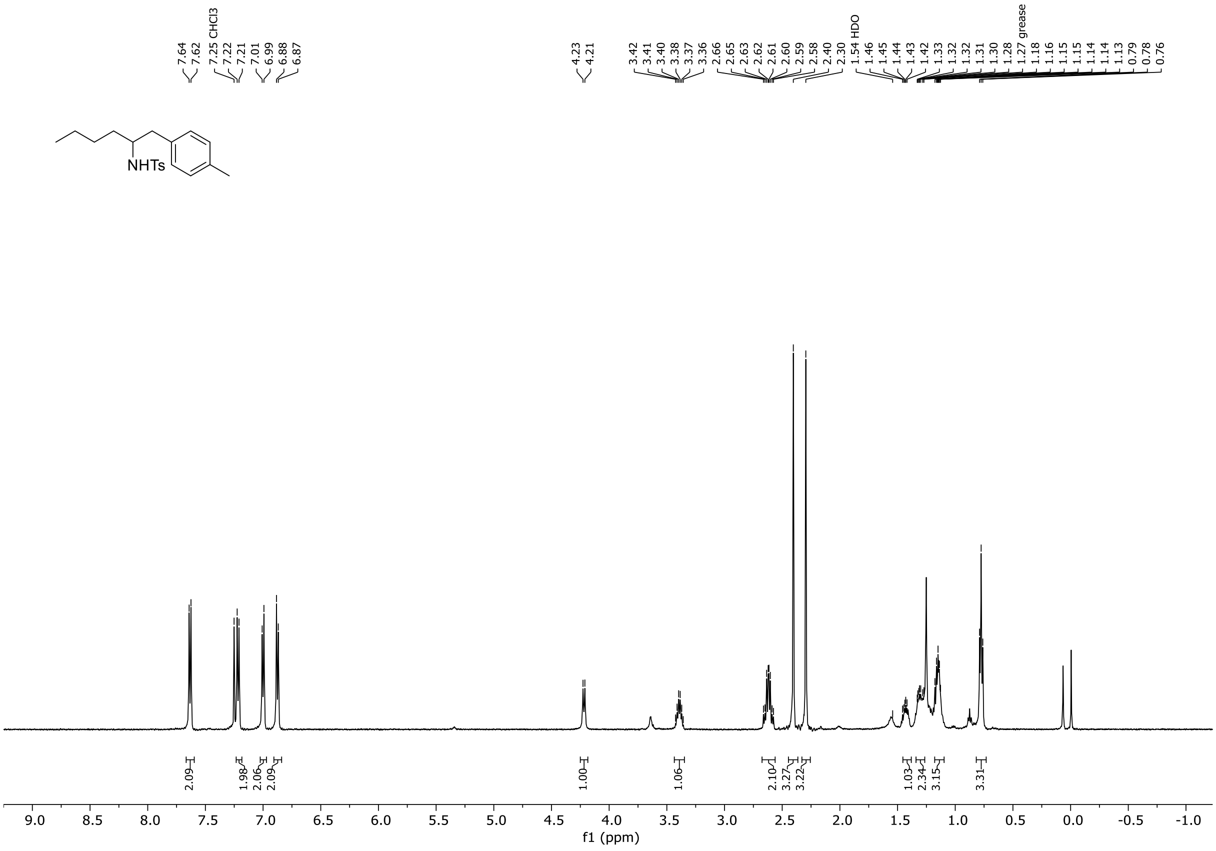

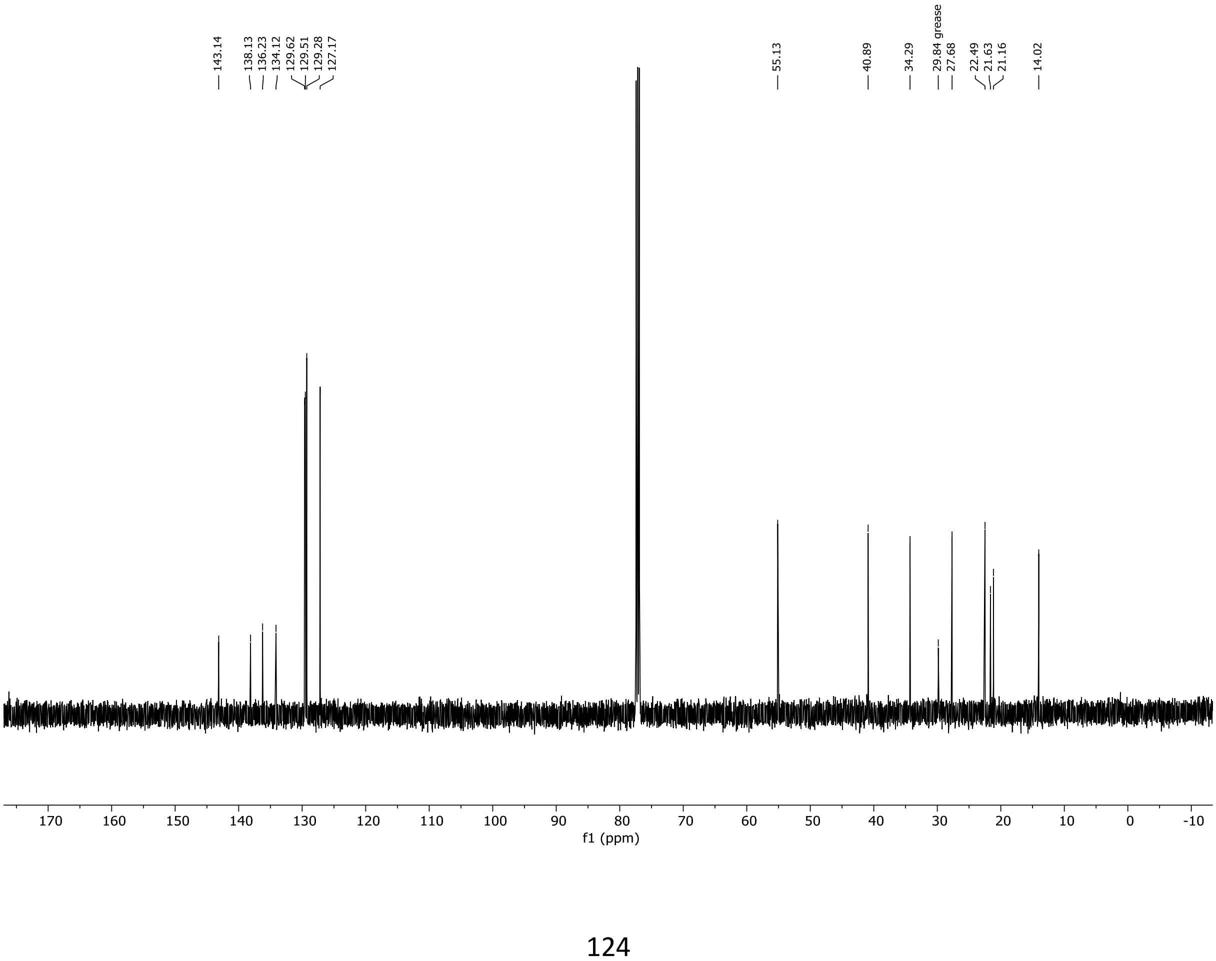

Supplement: Supplementary file 1 — ja1c00659_si_001.pdf [file ja1c00659_si_001.pdf]
